# *SGK1* repression by *WT1* may confer a survival advantage to leukemic cells under stress conditions

**DOI:** 10.1007/s00277-025-06458-z

**Published:** 2025-07-04

**Authors:** Miguel A. Rubio, Sabina Cisa-Wieczorek, Ana Mozos, Helena Castellet, Elena Bussaglia, Maite Carricondo, María Isabel Hernández-Alvarez, Jorge Sierra, Josep F. Nomdedéu

**Affiliations:** 1 Departament de Medicina, Universitat Autònoma de Barcelona, Barcelona, Spain; 2 Department of Pathology, Hospital de La Santa Creu I Sant Pau, Barcelona, Spain; 3 Departament de Bioquímica I Biomedicina Molecular, Facultat de Biologia, Universitat de Barcelona, Barcelona, Spain

**Keywords:** Leukemia, WT1, SGK1, Cell viability

## Abstract

Increased *WT1* mRNA levels are pervasive in acute myeloid leukemia (AML), and this marker has been used to assess the leukemic compartment size after chemotherapy or hematopoietic cell transplants. Little is known about the effects of *WT1* on the leukemic cells and their targets. This work used data obtained from gene expression arrays performed on AML samples with high and low *WT1* mRNA levels to pinpoint genes that WT1 can regulate. We singled out *SGK1*, which showed an inverse correlation between its mRNA levels and *WT1* in leukemic cell lines and AML samples. In cellular models, forced expression of *WT1* reduced mRNA and protein levels of *SGK1*. Furthermore, *WT1* repressed the SGK1 promoter activity, and accordingly, WT1 knockdown showed an increased expression of *SGK1*. We also detected an inverse correlation between *WT1* and *SGK1* during leukemic cell-line differentiation. *WT1* genetic knockdown displayed decreased cell viability under nutrient deprivation. By contrast, *SGK1* knockdown or pharmacologic inhibition increased resistance to apoptosis in response to serum starvation, acting on cell cycle progression. The effects of *SGK1* targeting in hematologic conditions merit further investigation.

**Supplementary Information:**

The online version contains supplementary material available at 10.1007/s00277-025-06458-z.

## Introduction

Acute myeloid leukemia (AML) is a heterogeneous group of neoplastic disorders that acquire driver mutations in the myeloid precursors. AML is more common in adults, and cytogenetic and molecular abnormalities influence the outcome. Current efforts in basic and clinical research focus on the genetic and epigenetic characterization of AML to better understand molecular pathogenesis and foster the discovery of new targeted therapies that would increase the cure rate [1].

The Wilms'tumor 1 (*WT1*) gene encodes a tumor suppressor gene identified initially by its inactivation in the Wilms tumor, a pediatric kidney cancer. *WT1* encodes a transcriptional regulator that can act as an activator or repressor. *WT1* is overexpressed in some solid tumors (i.e., mesothelioma and high-grade serous ovarian carcinoma) and most AML [2]. *WT1* levels in bone marrow provide relevant prognostic information in *de novo* AML treated with chemotherapy or hematopoietic cell transplantation [3]. *WT1* is upregulated in committed myeloid precursors in the healthy bone marrow, whereas stem cells and differentiated myeloid cells show low expression levels of *WT1*. Overexpression of *WT1* promotes leukemic cell proliferation [4] and impairs differentiation [5] and apoptosis [6, 7]. WT1 up-regulation is crucial in allowing neoplastic cell lines to adapt to hypoxia, upregulating the endothelial growth factor VEGF and increasing angiogenesis [8].

Both *WT1* overexpression and inactivating mutations can be found in clinical AML samples [9, 10]. In human leukemias, WT1 is mutated in 10% [3]. Earlier findings suggested that patients with *WT1* mutations had a dire prognosis, but this has not been confirmed. The leukemogenic capacity of high *WT1* mRNA levels has been linked to the DNA binding of *WT1* [2]. This binding was associated with regulating the expression of hundreds of genes [4, 11–13].

In this study, we look for new oncogenic targets of *WT1* by microarray analyses of AML patients with low or high *WT1* levels. Finding direct targets of *WT1* could guide us to the discovery of new leukemic pathways and novel therapeutic options for AML.

## Patients and methods

### Leukemia samples

Primary samples from AML patients were obtained from the Department of Hematology at the Hospital de la Santa Creu i Sant Pau (Barcelona, Spain) with full informed consent and the approval of the Institutional Review Boards. Peripheral blood or bone marrow aspirates were collected from 64 individual AML patients at diagnosis. Also, two potential donors for allogeneic bone marrow transplantation were used as controls. Clinical details of AML patients are given in Supplementary Tables 1 and 4. Mononuclear cells were isolated by density gradient centrifugation using Lymphoprep® (Alere Technologies, Oslo, Norway).

*WT1* copies were determined by RT-qPCR analysis using *ABL1* as internal control and shown as [*WT1/ABL1*] × 10^4^ copies (1). The cut-off point to establish low *vs*. high expression levels was 200 copies. As previously reported, analyses of *WT1* mutational status in exons 7 and 9 were determined (1).

### RNA preparation, labeling, microarray hybridization, and scanning

Total RNA was quantified with Nanodrop, and its quality was analyzed with a Bioanalyzer 2100 RNA chip (Agilent, Santa Clara, CA, USA). One hundred ng of RNA was retrotranscribed into sense strand cDNA using Ambion® WT Expression Kit for Affymetrix® GeneChip® WholeTranscript (WT) Expression Arrays (Applied Biosystems, Foster City, CA, USA), according to the manufacturer's procedure. 5.5 µg of the cDNA was then fragmented and labeled using Affymetrix ® GeneChip® WT Terminal Labeling Kit (Affymetrix, Santa Clara, CA, USA) following the manufacturer's procedure. 4.7 µg of the fragmented and labeled DNA was then hybridized on Affymetrix GeneChip® HG-U133 Plus 2.0 or GeneChip® Human Exon 1.0 ST Array cartridges using GeneChip Hybridization, Wash and Stain Kit, following the manufacturer's procedure. Arrays were hybridized in a 640 hybridization oven (Affymetrix) at 45ºC and 60 rpm for 17 h. Arrays were then washed, stained, and scanned according to the GeneChip® Expression Wash, Stain, and Scan User Manual.

### Cell starvation

For nutrient deprivation experiments, cells were seeded/plated at the indicated concentrations in a medium with FBS and grown without medium change for long periods, sometimes until 11 days. Cells were washed three times with PBS 1X for the serum withdrawal experiments, resuspended in a medium without FBS, counted, and seeded/plated at the indicated concentrations. These cells were also used in fasting-refeeding experiments; FBS was added to 10% after 72 h of withdrawal.

### *SGK1* luciferase reporter assay and *in silico SGK1* promoter analysis

HEK293T cells were plated in triplicate and transfected when exponentially growing (cell confluence below 50%) using Lipofectamine 2000 (Invitrogen) with 50 ng *pLightSwitch_Prom* (empty vector) or *pLightSwitch_SGK1* (#32001-S714486) containing 1471 bp of the *SGK1* isoform 1 promoter (SwitchGear Genomics, Carlsbad, CA, USA) plus 50 ng *pCMV-CB6* + + empty vector or *pCMV-CB6-WT1B, pCMV-CB6-WT1D* or *pCMV-CB6-WT1delZ* expressing *WT1* isoforms (±), (+/+) or *delZ* mutant, respectively. It is essential to provide the cells with a fresh medium before transfection. At 24 h post-transfection, cells were assayed for Renilla luciferase with LightSwitch Luciferase Assay Reagent (SwitchGear Genomics) and a Tecan Infinite M200 Pro luminometer (Tecan, Männedorf, Switzerland).

We used Ensembl genome browser release 98 (www.ensembl.org) to obtain 3000 bp of the *SGK1* isoform 1 promoter of seven mammals, taking as −1 the first base downstream of the + 1 start of the isoform 1 cDNA. The reference genome assembly and the orthologous *SGK1* isoform 1 transcript for each different organism is Mouse (GRCm38.p6, ENSMUST00000020145.11); Rat (Rnor_6.0, ENSRNOT00000016121.5); Human(GRCh38.p13, ENST00000237305.11); Dog (CanFam3.1, ENSCAFT00000046374.3); Pig (Sscrofa11.1, ENSSSCT00000027054.3); Goat (ARS1, ENSCHIT00000014208.1); and Cow (ARS-UCD1.2, ENSBTAT00000005592.4). Sequence alignment was performed with Clustal Omega (https://www.ebi.ac.uk/Tools/msa/clustalo/). *In silico* analysis of putative WT1 binding sites was performed with PROMO (http://alggen.lsi.upc.es/cgi-bin/promo_v3/promo/promoinit.cgi?dirDB=TF_8.3).

### Stable *WT1* overexpression

Overexpression of *WT1* was achieved by retrovirus-mediated expression of ORF-containing plasmids *pMWIG-WT1(* ±*)* and control plasmid *pMIG*, all with constitutive expression of GFP. Briefly, Phoenix-AMPHO cells were transfected with the plasmids plus packaging plasmids with Lipofectamine 2000. At 48, 60, and 72 h post-transfection, the supernatant containing viral particles was used to infect K562 and NB4 cell lines in the presence of 8 μg/ml polybrene. The infection efficiency was 40–50%. Cells were FACS sorted to > 99% GFP positive with BD FACSAria I (Becton Dickinson), and RNA was collected and analyzed after at least seven days post-infection. GFP and *WT1* expression in the cells was assessed for every experiment.

### Generation of stable *WT1* and *SGK1* knockdown cell pools

Human *WT1* and *SGK1* shRNA plasmids were previously described and validated target sequences:

- pLKO.1-shWT1-1 (shWT1 1), sequence:
- 5'-CCGGGCATCTGAGACCAGTGAGAAACTCGAGTTTCTCACTGGTCTCAGATGCTTTTTG-3'
- pLKO.1-shWT1-2 (shWT1 #2), sequence:
- CCGGGGTGAATCTTGTCTAACATTCCTCGAGGAATGTTAGACAAGATTCACCTTTTTG pLKO.1-shSGK1-1 (shSGK1 D), sequence:
- CCGGGCAATCTTATTGCACACTGTTCTCGAGAACAGTGTGCAATAAGATTGCTTTTTG pLKO.1-shSGK1-2 (shSGK1 A), sequence:
- CCGGCGGAATGTTCTGTTGAAGAATCTCGAGATTCTTCAACAGAACATTCCGTTTTTG

Lentiviral plasmid *pLKO.1-shWT1-1* was generated by the Broad Institute RNAi Consortium and purchased from Sigma-Aldrich (MISSION shRNA Bacterial Glycerol Stock SHCLNG-NM_024426, TRCN0000040067). *pLKO.1-shWT1-2, pLKO.1-shSGK1-1,* and *pLKO.1-shSGK1-2* were constructed by cloning the target sequence between the *AgeI* and *EcoRI* sites of *pLKO.1-TRC* (Addgene plasmid 10878). *pLKO.1-TRC* and control vectors *pLKO.1-scramble* shRNA and *pLKO.1-GFP shRNA*) were kindly provided by David Root and David M. Sabatini (Addgene plasmids 1864 and 30323). The production of lentiviral particles was performed by transient cotransfection (with Lipofectamine 2000) of 293FT cells with packaging plasmids *psPAX2* and *pCMV-VSVG*, gifts from D. Trono and R. Weinberg (Addgene plasmids 12260 and 8454). Viral supernatants were harvested 48, 60, and 72 h post-transfection and applied to K562 and NB4 cells for infection in the presence of 8 μg/ml polybrene. Two days after infection, cells were selected for at least three days with puromycin at 1 μg/ml. The infection efficiency was 70–90%, as assessed by counting the number of cells growing with or without puromycin. The knockdown of *WT1* or *SGK1* alone did not affect the proliferation or viability of the AML cell lines. In some transductions (especially in NB4 cells with *shWT1*), we observed the loss of the knockdown after growing the cells in culture for two or more weeks, probably because of silencing the shRNA. The cells were still puromycin-resistant. For this reason, we used cells that were recently infected (6–15 days) for all experiments, and the knockdown efficiency was checked at the mRNA level in every experiment.

For complete methods see suppl material.

## Results

### Global gene expression profiles differ between AML patients with high and low *WT1* levels

We compared three microarray experiments to find the most representative genes whose expression depends on *WT1* levels in adult AML patients to search for common genes. None of the selected patients had *WT1* mutations. The first experiment analyzed differentially expressed genes in 14 diagnostic AML samples (Supplementary Table 1) with low versus high *WT1* levels, using Affymetrix GeneChip® HG-U133 Plus 2.0 microarrays. The second experiment, a similar analysis using bone marrow samples from 5 AML cases, used the Affymetrix GeneChip® Human Exon 1.0 ST microarray (Supplementary Table 1). We used The Cancer Genome Atlas (TCGA) gene expression dataset for the third analysis in AML samples [10]. From 183 microarrays, we selected seven patients with the highest and the lowest levels of *WT1* (Supplementary Table 2).

**Fig. 1:**
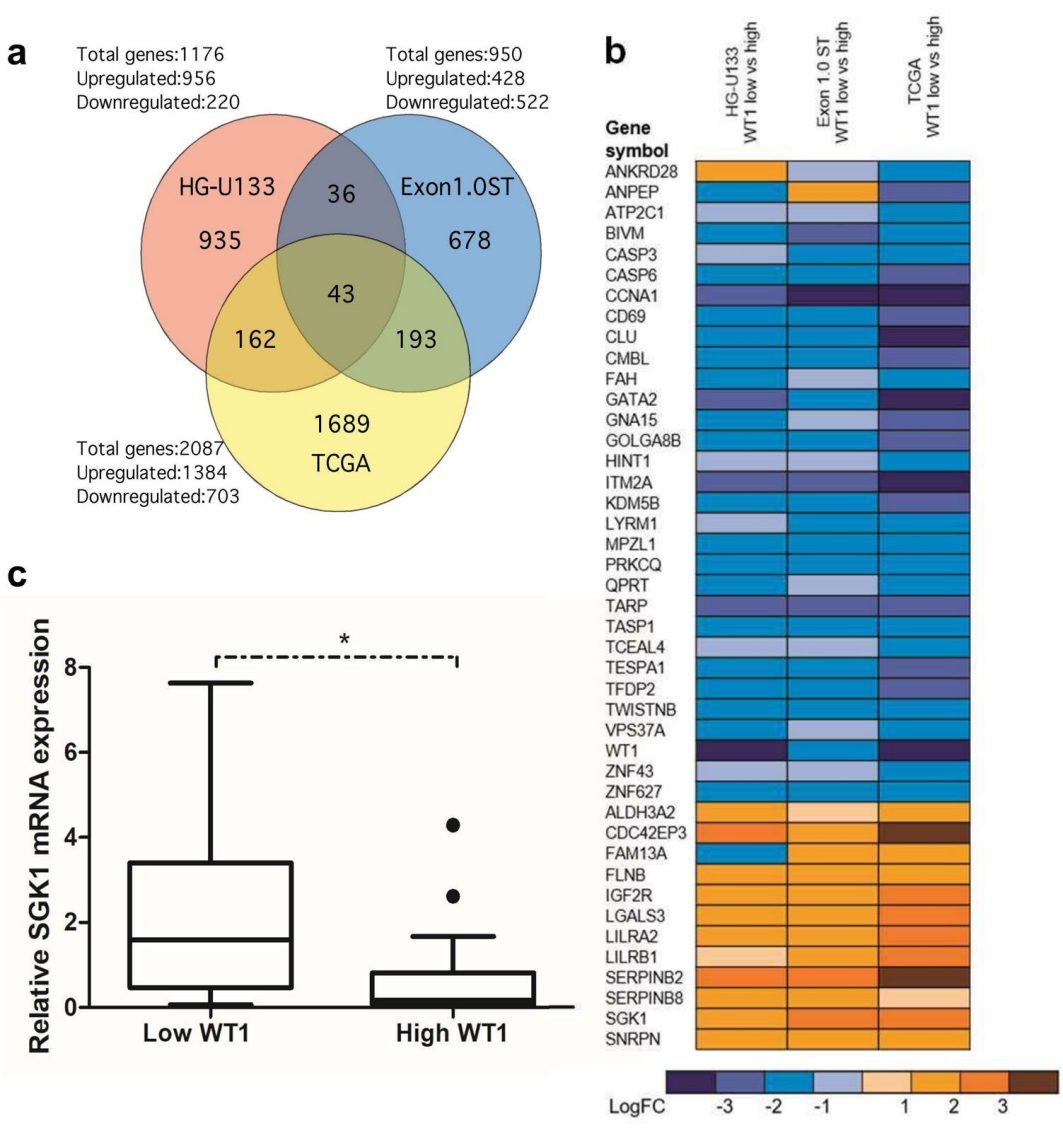
Comparison of microarray experiments. (**a**) Venn diagram of differentially expressed genes (*P* < 0.05) with the number of shared genes in the three microarray analyses of low vs. high *WT1* patients. Up/downregulated number is in patients with high levels of *WT1*. (**b**) Heat map showing relative expression level (log of fold change) of the 43 genes common to the three analyses. (**c**) *WT1* expression is associated with reduced levels of *SGK1* mRNA. The box-and-whisker plots illustrate the distribution of *SGK1* expression values in bone marrow samples of patients with low (*n* = 22) or high (*n* = 23) *WT1* mRNA levels. Student’s *t*-test, *P < 0.05

We obtained a set of differentially induced or repressed genes for each analysis. Significative genes for each experiment are detailed in Supplementary Table 3. Comparison of these lists showed a group of 43 genes present in the three analyses (Fig. 1a), with twelve genes, overexpressed and 31 underexpressed in patients with low *WT1* levels (Fig. 1b). The gene with the most extreme fold change values in Affymetrix GeneChip® HG-U133 Plus 2.0 microarray and TCGA Atlas was *WT1*, confirming the validity of the analysis (Fig. 1b).

In this group of 43 genes, we found known regulators of *WT1*, like *CCNA1* [13] and *GATA2*1 [14, 15]. Other genes in this set were regulated by *WT1*, such as *ANPEP* [5] and *QPRT* [16].

### Inverse correlation between *WT1 *and *SGK1* levels in AML patients

There is no reported connection with WT1 for some differentially expressed genes. One is serum- and glucocorticoid-regulated kinase 1 (*SGK1*), a stress-induced gene overexpressed in patients with low *WT1* levels. SGK1 was a good choice because its abnormal expression has significant cellular effects and is strongly linked to human cancer. *RT-qPCR analyzed SGK1* expression in 45 AML patients without a mutation in WT1 (Supplementary Table 4). When comparing the 22 cases with low and the 23 patients with high *WT1* expression, the mean of SGK1 expression was higher in the low WT1 vs high WT1 groups. (*P* < 0.05, Fig. 1c).

We also studied the expression of WT1 and SGK1 at the protein level by immunohistochemistry (IHC). We found that WT1 mRNA levels correlated with its protein expression (*p* = 0.028). Moreover, there was a strong correlation between WT1 mutation and SGK1 IHC expression (*p* = 0.02). We also found SGK1 protein expression tended to be higher in cases with low expression mRNA.

### Forced expression of *WT1* decreases *SGK1* mRNA and protein levels.

**Fig. 2:**
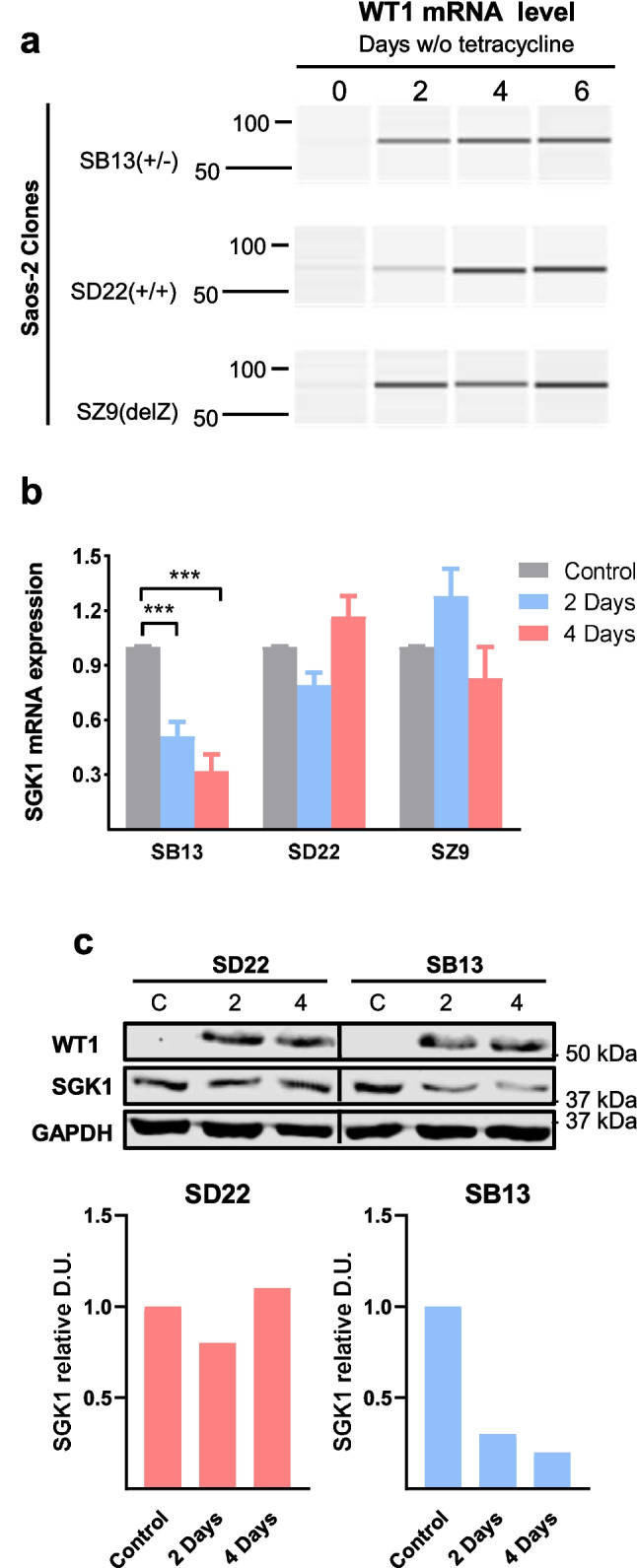
*WT1* represses the expression of *SGK1*. (**a**) RT-PCR analysis of inducible *WT1* mRNA levels in Saos-2 tetracycline-repressible clones (SB13: *WT1* (±), SD22: *WT1* (+/+), SZ9: *WT1-delZ* mutant) at different times after tetracycline removal. One representative experiment of three is shown. (**b**) RT-qPCR analysis of *SGK1* mRNA levels in the same clones 2 or 4 days after tetracycline removal and control with tetracycline. *ABL1* expression was used as an internal control. The results are the mean (SD) of three independent experiments. Student's *t*-test, ***P < 0.001. (**c**) Immunoblot analysis of WT1 and SGK1 expression in Saos-2 tetracycline-repressible clones grown with tetracycline or after removal for 2 or 4 days. One representative experiment of three is shown. GAPDH protein was used as an internal control. Relative levels of SGK1 (controls set to 1), as determined by densitometry units (D.U.) and normalization to GAPDH, are shown. The positions of prestained molecular mass markers are indicated to the right

The first alternative splice comprises exon 5, encoding 17 amino acids (51 nucleotides) inserted between the transactivation and DNA binding domains. The second alternative splice includes exon 9, encoding a tripeptide KTS, between the third and fourth zinc fingers of the *WT1* protein. So, an alternative splice is indicated with + and – for the absence. We used clones of human osteosarcoma cell lines Saos-2 and U2OS with tetracycline-repressible isoforms of *WT1* [20]. The WT1 gene is alternatively spliced at two sites. As previously described [21], the culture of Saos-2 clones SB13 (expressing inducible isoform *WT1*(±)), SD22 (*WT1*(+/+)), and SZ9 (*WT1-delZ* mutant) in the absence of tetracycline induced high levels of exogenous *WT1* mRNA (Fig. 2a). Expression of *SGK1* mRNA was reduced by 70% only in the SB13 clone expressing the *WT1(* ±*)* isoform (Fig. 2b). So, the repressive effect of *WT1* on *SGK1* expression was specific for *WT1* isoforms*,* at least in these non-leukemia cell lines.

WT1 protein was also induced in SB13 and SD22 after tetracycline removal (Fig. 2c). SB13 showed a 20% decrease in SGK1 protein levels compared to controls (Fig. 2c). There was no SGK1 decrease in clone SD22.

### *WT1* knockdown induces *SGK1* expression

**Fig. 3:**
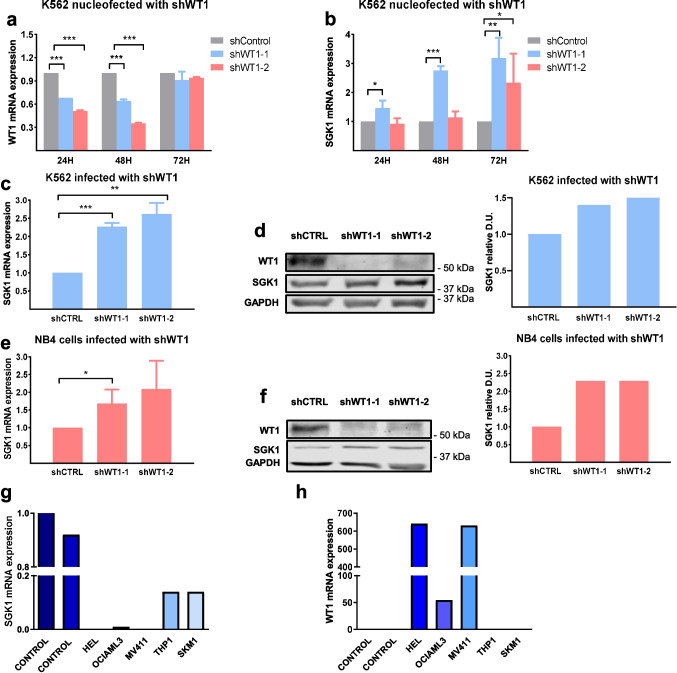
*WT1* knockdown induces *SGK1* expression. (**a**, **b**) K562 cells, nucleofected with two different shRNAs against *WT1* (shWT1) or shSCR (shControl), were incubated for the indicated times and analyzed for *WT1* knockdown (**a**) or *SGK1* mRNA levels (**b**) by RT-qPCR. (**c**, **d**) K562 (**c**) or NB4 (**d**) cells were infected with lentivirus containing two different shRNAs against *WT1* or shSCR as control; puromycin was selected for 11 days and analyzed for *SGK1* levels by RT-qPCR. Results are the mean (SD) of three independent experiments with a control set to 1. The *ABL1* gene was used as an internal control. Student's t-test, *P < 0.05; **P < 0.01; ***P < 0.001. (**e**, **f**) Immunoblot of WT1 and SGK1 expression in K562 (**e**) or NB4 (**f**) cells infected with lentiviral control (shSCR) or knockdowns for *WT1* (sh*WT1-1* or -*2)*. Relative levels of SGK1 protein are shown (control is set to 1). One representative experiment of three is shown. (**g**, **h**) RT-qPCR analysis of WT1 (**g**) and SGK1 (**h**) levels in leukemia cell lines. *ABL1* expression was used as an internal control, and the control human samples normalized the results

Many leukemia cell lines express high levels of wild-type *WT1* mRNA [14], for example, NB4 and K562 (Fig. 3a, Fig. Suppl 2). To test whether a reduction of *WT1* levels by RNA interference could induce SGK1, we transiently transfected K562 cells with plasmids expressing two small hairpin RNAs (shRNAs) against *WT1* (Fig. 3a). We determined *SGK1* expression at three different time points. *WT1* knockdown induced *SGK1* mRNA expression to a maximum of 3.18 fold of that observed in control cells (Fig. 3b). The induction of *SGK1* was later than *WT1* induction, which is compatible with *SGK1* being a downstream target of *WT1* (Fig. 3a, b).

A similar reduction in *SGK1* mRNA was seen when K562 (Fig. 3c) or NB4 cells (Fig. 3e) were infected with lentivirus containing two different shRNAs against *WT1* and puromycin selected(Fig. Suppl 1). The immunoblot confirmed WT1 knockdown by stable infection at the protein level with an anti-WT1 antibody (Fig. 3d-f). There was an induction of SGK1 protein levels in K562 (Fig. 3e) and NB4 cells (Fig. 3f). We concluded that *WT1* represses *SGK1* expression in leukemic cells and that, accordingly, *WT1* reduction results in increased *SGK1* levels. We have measured WT1 and SGK1 mRNA levels in leukemic cell lines (Fig. 3g, h). We have found that this inverse relationship between WT1 and SGK1.

### *WT1 *represses *SGK1* promoter activity

**Fig. 4:**
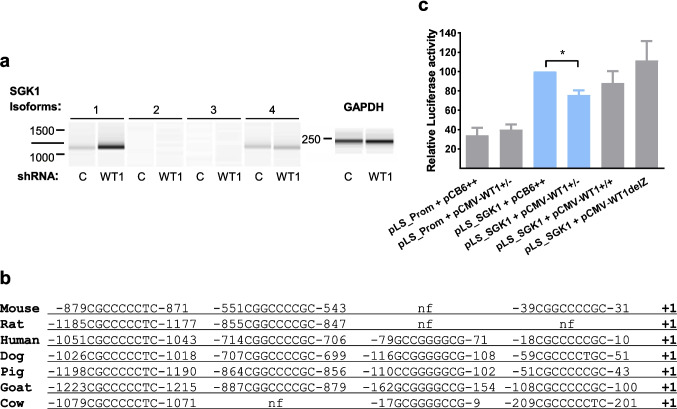
*WT1* acts on the promoter of *SGK1* isoform 1. A Semiquantitative PCR analysis of *SGK1* mRNA isoforms 1–4 in K562 cells infected with control shSCR (C) or *WT1* knockdown lentivirus *shWT1-1* (WT1). Internal control is *GAPDH* mRNA. (**b**) Four WT1 binding sites in the human promoter of *SGK1* isoform one are conserved in another six mammal species. Numbers indicate base pairs downstream of the + 1 start site of the isoform 1 cDNA, nf: not found **c***WT1* represses transcription from the *SGK1* isoform 1 promoter. 293 T cells were transfected with the SGK1 promoter-luciferase reporter *pLightSwitch_SGK1* (*pLS_SGK1*) or the empty reporter *pLightSwitch_Prom* (*pLS_Prom*) and cotransfected with empty vector (pCB6 + +) or pCMV plasmids expressing *WT1* isoforms (±), (+/+) or *delZ*. After 24 h, luciferase activity was analyzed. Results expressed as mean (SD) (*n* = 3). The *pLS_SGK1* plus empty vector activity is set to 100%, and other measurements are presented relative to this. Student's t-test, **P < 0.01

Next, we investigated the mechanism by which WT1 affects SGK1 expression. There are four primary alternatively spliced forms of *SGK1*, isoforms 1 to 4, arising from at least three promoters [22]. We first used semiquantitative PCR to determine the expression levels of the four *SGK1* isoforms in K562 cells (Fig. 4a). We also tested *WT1* knockdown after shRNA infection of K562 affected the expression of each isoform. In K562 cells, the levels of isoforms 2 and 3 were undetectable, even after 40 cycles of qPCR. Isoform 4 was detected, but levels remained constant after *the WT1* knockdown. The primary *SGK1* isoform levels in most human cells, *SGK1* isoform 1, were highly induced after *WT1* knockdown (Fig. 4a).

Next, we performed a comparative genomic analysis of 3kb downstream the 5’ region of the *SGK1* isoform 1 gene across seven mammalian species. *In silico* analysis revealed the presence of nine putative WT1 binding sites in the human promoter (similar to the consensus site of EGR1 (ref. 2)). Four of them were located in highly conserved regions present in most or all of the compared species (Fig. 4b).

We performed luciferase assays with a plasmid containing 1471 *SGK1* isoform 1 promoter base pairs, including the four conserved regions (Fig. 4b), cloned into the luciferase reporter *pLightSwitch_Prom* or a control vector. Reporter plasmids were cotransfected with *WT1* expressing plasmids in 293 T cells, and the activity of the promoter in response to the overexpressed *WT1* was determined. Cotransfection with *WT1*(±) resulted in significant repression of the *SGK1* promoter activity (*p* = 0.001048) (Fig. 4c). This outcome was not seen with the overexpression of *WT1*(+/+) or the *delZ* mutant. We concluded that *WT1*(±) could repress the main *SGK1* isoform (isoform 1), probably by binding to the promoter in one or more conserved *WT1* consensus sites. This result suggests the role of *WT1* as a critical transcriptional regulator of *SGK1*.

### Inverse correlation between *WT1* and *SGK1* levels during leukemic cell-line differentiation.

*WT1* is involved in leukemia cell differentiation [5, 9]. There are few reports of *SGK1* acting in leukemia, most of them describing an *SGK1* loss of function [19, 20], as could be expected of a tumor suppressor. Also, multiple reports note the induction of *SGK1* during blood cell differentiation and activation [17, 22, 23], but direct involvement of *SGK1* in leukemic cell differentiation has yet to be reported. *WT1* could alter differentiation, at least partially, through *SGK1* modulation. In agreement with our hypothesis, a search in the Bloodspot database (www.bloodspot.eu) showed a broad but consistent inverse relationship between the levels of *WT1* and *SGK1* in normal human hematopoiesis (Fig. Suppl 2 A) and in different types of leukemia (Fig. Suppl 2B).

**Fig. 5:**
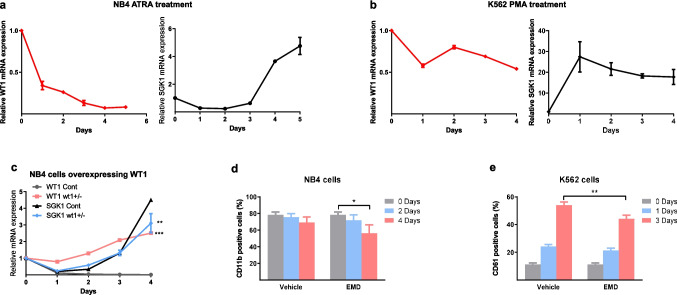
*WT1* and *SGK1* changes during leukemic cell differentiation. (**a**) RT-qPCR analysis of *WT1* and *SGK1* mRNA levels in NB4 cells after ATRA treatment. Internal control is *ABL1,* and the day 0 value is set to 1. (**b**) RT-qPCR analysis of *WT1* and *SGK1* mRNA levels in K562 cells after PMA treatment. Internal control is *ABL1*. (**c**) RT-qPCR analysis of *WT1* and *SGK1* mRNA levels in NB4 cells overexpressing *WT1*(±) or control (Cont), after ATRA treatment. Internal control is *GUSB*. P values were calculated vs the control (**d**) Expression of CD11b was analyzed by flow cytometry in NB4 cells treated with vehicle or 50 μM EMD638683 for the indicated times. (**e**) Expression of CD61 was analyzed by flow cytometry in K562 cells treated with PMA and vehicle or 50 μM EMD638683 for the indicated times. Mean (SD) from three different experiments. Student's *t*-test, *P < 0.05, **P < 0.01

As previously reported [24], we observed a reduction in *WT1* mRNA levels during the differentiation of NB4 cells with all-trans retinoic acid (ATRA) (Fig. 5a). This reduction preceded a high induction of *SGK1* mRNA (Fig. 5a). The differentiation of NB4 cells was confirmed by increased granulocyte markers CD11b and CD11c (Fig. Suppl 2 C).

In the K562 model of differentiation with phorbol-myristate acetate (PMA), we also observed a reduction of *WT1* mRNA levels (Fig. 5b), as has been previously reported [25], and concomitant induction of *SGK1* mRNA (Fig. 5b). Megakaryocytic differentiation was confirmed by CD61 induction (see below). Thus, the reduction of *WT1* and induction of *SGK1* levels appear to be a common phenomenon during differentiation in the hematopoietic and leukemia models.

To see if the downregulation of *WT1* affects the induction of *SGK1* during differentiation, we used a retrovirus to obtain NB4 cells constitutively overexpressing the *WT1(* ±*)* isoform. We differentiated these cells with ATRA in parallel with NB4 control cells. After four days, we observed a blunted induction of *SGK1* in the *WT1(* ±*)* overexpressing cells (Fig. 5c). The inhibitory effect of *WT1* on *SGK1* was lost at later time points (7 days.

Similarly, we obtained K562 cells constitutively overexpressing the *WT1*(±) isoform. After the differentiation of these and control cells with PMA, we did not see a blunted induction of *SGK1* (Fig Suppl 2D), which is consistent with the fact that *WT1* downregulation is not a prerequisite for K562 differentiation [25].

### Effect of *SGK1* inhibition on leukemic cell differentiation

The involvement of SGK1 in the differentiation process in leukemic cells could suggest a tumor suppressor behavior. To examine the role of SGK1 in differentiation, we treated NB4 cells with the *SGK1* inhibitor EMD638683 [26]. We observed a reduction of the basal level of CD11b (Fig. 5d), which indicated a dedifferentiation process.

We saw that EMD638683 could partially inhibit the induction of CD61 in K562 after PMA treatment (Fig. 5e). Again, probably due to a different level of *SGK1* inhibition, we could not replicate this result in K562-*shSGK1* cells, and CD61 was induced with the same kinetics as in control cells (Fig. Suppl 2E).

### *WT1* or *SGK1* downregulation changes leukemic cell viability during nutrient deprivation

**Fig. 6:**
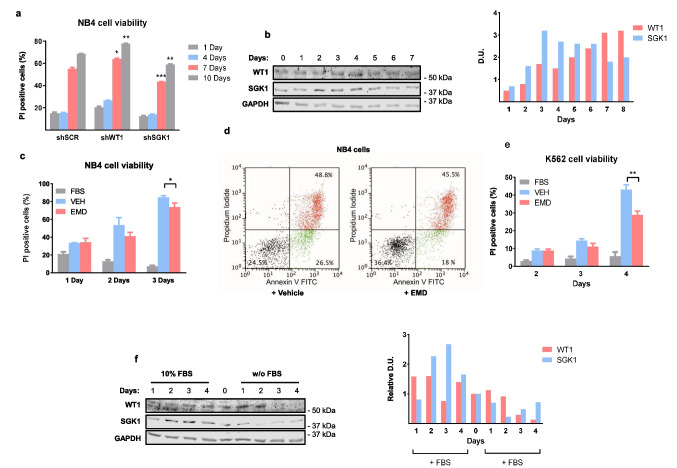
*SGK1* effects on nutrient deprivation and apoptosis. (**a**) Effect of *WT1* or *SGK1* knockdown on cell viability during starvation. NB4 cells infected with control lentivirus (*shSCR*) or knockdowns (*shWT1-2* or *shSGK1-1*) were plated at 300000 cells/ml, and the medium was not changed for the indicated times. Viable cells were determined by flow cytometry after staining with propidium iodide (PI). The fraction of PI-positive death cells is expressed as a percentage of total cells (mean (SD)). (**b**) Immunoblot shows WT1 and SGK1 protein levels in NB4 cells in culture for the indicated times without medium change. Internal control GAPDH. (**c**) NB4 cells plated at 150000 cel/ml were treated with vehicle (VEH), EMD 638683 (EMD), and cultured in medium without FBS for the indicated days. Control (FBS) cells grow in a medium with 10% FBS. Calculations as in (**a**). (**d**) NB4 cells were treated with vehicle (left panel) or EMD638683 (right panel), cultured without FBS for three days, and flow cytometry analysis was done after labeling with annexin V-FITC and PI. Percentage (mean of biological triplicates) of viable (FITC-/PE-), early (FITC +/PE-), and late (FITC +/PE +) apoptotic cells is shown. (**e**) K562 cells plated at 300000 cells/ml were treated with DMSO (VEH) or EMD638683 (EMD) and cultured in a medium without FBS for the indicated days. Control (FBS) in medium with 10% FBS. Calculations (*n* = 3) as in (**a**). Student's t-test, * *P* < 0.05, ** *P* < 0.01. **f** WT1 and SGK1 protein levels in K562 cells after FBS withdrawal for 0–4 days (w/o FBS) and controls in 10% FBS. GAPDH: internal control

Due to the limited effect of *SGK1* on leukemic cell differentiation, we examined if the kinase could be involved in another tumor-suppressive process. During the NB4 differentiation experiments with ATRA, we observed that untreated NB4 cells infected with *shSGK1* (Fig. 3f) and left in culture without medium change were more resistant to nutrient deprivation than control cells as shown with PI staining to measure cell viability (Fig. 6a). Similarly, NB4 cells treated with the *SGK1* inhibitor EMD638683 were more resistant to nutrient deprivation than untreated cells (Fig. Suppl 3 A).

SGK1 and WT1 protein levels in NB4 cells showed an oscillatory pattern in standard culture conditions but remained relatively constant even under cell starvation (Fig. 6b). However, the oscillatory pattern of *WT1* and *SGK1* mRNA levels was more pronounced (Fig. Suppl 3B). In K562 cells, *SGK1* mRNA and protein levels increased in culture after 3–4 days (Fig. Suppl 3 C).

*SGK1* is transcriptionally induced by serum [23], which makes SGK1 induction during nutrient deprivation a counterintuitive effect. Fasting-refeeding experiments in serum-starved NB4 and K562 cells showed a fast *SGK1* mRNA induction hours after FBS refeeding, with a concomitant reduction in *WT1* mRNA (Fig. Suppl 3D, E). These results agree with IGF-1 inhibition of WT1 when added to serum-starved Saos-2 cells [27]. These data indicate that a fast (in hours) *SGK1* induction by serum, as seen in other cell types [23], is conserved in leukemic cells. We speculate that a different mechanism is responsible for the slow (in days) induction of *SGK1* during K562 starvation (Fig. Suppl 3 C).

### EMD638683 induces resistance to apoptosis caused by FBS withdrawal in leukemic cells

We cultured cells in a medium without FBS to respond to nutrient deprivation faster. NB4 cells rapidly enter into apoptosis (Fig. Suppl 3 F). Interestingly, treatment with 50 μM EMD638683 reduced cell death compared with vehicle-treated controls (Fig. 6c). There was a reduction in both early and late apoptosis (Fig. 6d). After two days in a culture without FBS, NB4 cells showed a reduction in *WT1* and induction of *SGK1* mRNA levels (Fig. Suppl 3G).

Similarly, 50 μM EMD638683 increased cell viability in serum-starved K562 cells (Fig. 6e). The cells died by apoptosis, as shown by annexin V and PI staining (Fig. Suppl 3 F). Surprisingly, WT1 and SGK1 protein levels showed an unusual behavior in K562 cells after FBS deprivation (Fig. 6f). For the first 24 h, WT1 was induced, and SGK1 was reduced. After that, *WT1* levels started to decrease, and as the cells underwent massive apoptosis, there was a slight induction of SGK1. *SGK1* mRNA levels increased without a reduction of *WT1* mRNA levels (Fig. Suppl 3H).

We concluded that nutrient-deprived leukemic cells maintain or even upregulate *SGK1* levels in response to an increase in apoptotic cell death, usually preceded by a reduction in *WT1*. The death of starved cells can be partially abrogated by SGK1 inhibition, pointing to a tumor suppressor role of *SGK1* in this cellular context.

### *WT1* and *SGK1* knockdowns change leukemic cell resistance to serum deprivation

**Fig. 7:**
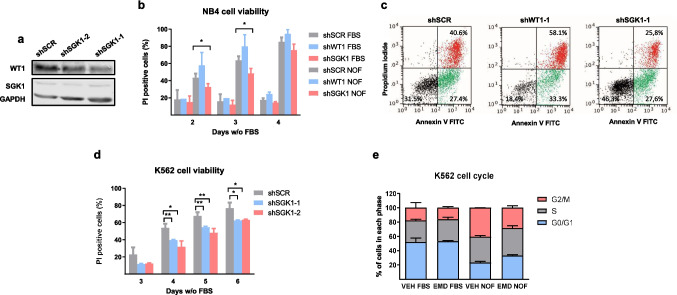
Effect of *SGK1* inhibition on cell cycle and apoptosis during serum withdrawal. (**a**) Immunoblot of WT1 and SGK1 expression in K562 NB4 cells infected with lentiviral controls (shSCR, or knockdowns for *SGK1* (*shSGK1-1* or 2). Relative levels of SGK1 protein are shown (control is set to 1). (**b**) NB4 control cells (*shSCR*) and knockdowns (*shWT1-2* and *shSGK1-1*) were plated at 300000 cel/ml and cultured in a medium without FBS (NOF) for the indicated days. Control (FBS) cells grow in 10% FBS. Viable cells were determined by PI staining and flow cytometry (*n* = 3). The fraction of PI-positive dead cells is expressed as a percentage of total cells (mean (SD)). (**c**) Changes in apoptosis. NB4 cells were infected with control lentivirus *shSCR*, *shWT1*, or *shSGK1-1* and cultured without FBS for three days. FACS analysis was done after labeling with annexin V-FITC and PI. The percentage (mean of biological triplicates) of viable, early, and late apoptotic cells is shown. (**d**) Effect of *SGK1* knockdown on K562 viability. Control cells (*shSCR*) and knockdowns (*shSGK1-1* and *shSGK1-2*) were plated at 300000 cel/ml and cultured without FBS for the indicated days. Calculations as in (**b**). (**e**) Cell cycle analysis of K562 cells treated with vehicle (VEH) or *SGK1* inhibitor (EMD), and growing for 48 h in the presence (FBS) or absence (NOF) of FBS. We determined the percentage of cells in each cell cycle phase through PI staining and FACS analysis of the DNA content (mean (SD)). Student's *t*-test, * *P* < 0.05, ** *P* < 0.01

Next, we used WT1 and SGK1 knockdowns to assess their effects on the viability of leukemic cells under starvation stress using PI staining. After shSGK1 infections, we did not detect changes in *WT1* mRNA and protein levels (Fig. 7a), suggesting that *WT1* inhibits *SGK1*, but *SGK1* does not regulate *WT1*.

*WT1* knockdown made NB4 cells more sensitive to serum starvation (Fig. 7b). However, we cannot rule out a deleterious effect of *WT1* depletion in these cells. *SGK1* knockdown made NB4 more resistant to FBS deprivation (Fig. 7b). This effect was also detected in counting viable cells with a hemocytometer, in the subG1 fraction in the cell cycle (see below) and in annexin V-FITC/PI experiments (Fig. 7c), where we saw that the main difference between NB4-*shSGK1* and control cells was in late apoptosis, with minor changes in the number of early apoptotic cells.

In K562 cells (Fig. 7d), the *SGK1* knockdowns (Fig. Suppl 4 A) also reduced apoptotic levels after FBS removal.

We only found increased viability by *SGK1* inhibition on starved cell lines (serum and nutrient deprivation stress) and not in NB4 cells treated with ATRA or K562 cells treated with imatinib.

### *SGK1* inhibition changes the cell cycle parameters of leukemic cells grown without serum

To understand how *SGK1* inhibition increased cell survival, we performed cell cycle analysis on K562 cells grown in medium with or without FBS in the presence of vehicle or 50 μM EMD638683. Serum withdrawal increased the number of cells in the G2/M phase (Fig. 7e) when the cells started to die (Fig. 6e). *SGK1* inhibition partially blocked cell accumulation in the G2/M phase (Fig. 7e) and increased cell survival. This effect was specific to nutrient-deprived cells, as cells growing in 10% FBS did not change their cell cycle parameters in the presence of EMD638683 (Fig. 7e).

Serum-starved NB4 cells were mainly found in the G1 phase (Fig. Suppl 4B), and there was a concomitant reduction in S-phase cells. *SGK1* inhibition partially blocked the increase in G1 phase cells but did not affect the number of S-phase cells. We saw no effect of EMD638683 in cells grown in a standard medium.

## Discussion

*WT1* mRNA levels are increased in most AML at diagnosis and can be used to follow up the efficacy of standard antileukemic therapies [3]. The effects of raised *WT1* on the leukemic cells are poorly understood. In this work, we used microarray analyses to look for new oncogenic targets of *WT1* and focused on *SGK1*, as there is no reported function in AML. *SGK1* is a serine/threonine kinase that shares structural and functional similarities with *AKT, PKC,* and *S6K*. *SGK1* is a master regulator of viability processes regulating cell survival and apoptosis transitions. It is induced in neurons after cardiac arrest and plays a role in the ischemia–reperfusion cardiac insult [17, 18]. There are no reports about *SGK1* involvement in AML, but it has been described as mutated in B-cell lymphoma [19, 20]. *S*tress-related molecules [23] induce SGK1 expression, which regulates various physiological functions, including epithelial transport, excitability, cell proliferation, and apoptosis [23, 28]. *WT1* is also involved in apoptosis [6, 7]. We found an inverse correlation between *SGK1* and *WT1* mRNA levels in clinical AML samples.

*WT1* and *SGK1* can act as oncogenes or tumor suppressors, depending on the context [2, 18]. *SGK1* may be upregulated in some cancers, but its expression could also be down-regulated in prostate, ovarian, and hepatocellular carcinoma [28]. Like *AKT* isoforms, *SGK1* does not appear to be mutated in human AML, as expected if the gene acts as a tumor suppressor. However, a recent study has found frequent *SGK1* mutations in NLPHL [19]. Mutations of *SGK1* were also detected in DLBCL and follicular lymphoma [20]. The new classifications of DLBCL have included the mutational status of *SGK1* as characteristic of some histogenetic categories [29, 30].

In AML, *WT1* acts predominantly as an oncogene. We show that forced expression of *WT1* reduced mRNA and protein levels of *SGK1*, and *WT1* knockdown induced the expression of *SGK1* in leukemic cells. Since *WT1* represses the *SGK1* promoter, *SGK1* might act as a tumor suppressor in this context by inducing the differentiation of leukemic cells. It is known that both *WT1* and *SGK1* act on hematopoietic differentiation [5, 17]. Indeed, we see that *WT1* overexpression partially blocks *SGK1* induction during NB4 differentiation with ATRA and that *SGK1* inhibition reduces NB4 and K562 differentiation markers. Our findings are in line with those observed in the colon. *SGK1* is expressed in more differentiated cells at the top of the colonic crypt. Lower *SGK1* levels have been associated with a maturation defect and aggressive behavior in colon carcinoma [31].

The mild phenotype observed in SGK1 knockout mice suggests that the action of this kinase is not critically important for maintaining housekeeping functions. However, following appropriate challenges (serum starvation as it could be in the highly proliferative leukemic niche), the lack of *SGK1* could be advantageous for the leukemic cells [23, 28]. In fasting-refeeding experiments, we first report the classic [23] fast *SGK1* mRNA induction in leukemic cells. However, at the same time, we find a slower response to nutrient deprivation, with *SGK1* mRNA and protein upregulation that correlates with massive apoptosis and usually with *WT1* downregulation. *SGK1* inhibition in this context increases cell viability, at least in part affecting cell cycle progression, giving a selective advantage to leukemic cells. Cell growth in serum-free or serum-reduced conditions indirectly measures cancer cells'adaptive mechanism to environmental stress. We speculate that *SGK1* could be involved in a nutrient deprivation-specific checkpoint in leukemic cells, and SGK1 inhibition would result in increased cell survival due to lower activation of cell cycle checkpoints and subsequent death. Our findings suggest that *WT1* renders leukemic cells more resistant to metabolic stress, and SGK1 activation or WT1 targeting could contribute to eradicating leukemic cells.

We used a pharmacologic inhibitor to block the SGK1 function, previously used with other drugs in solid neoplasms. In these systems, SGK1 inhibition sensitized tumoral cells to specific therapies [26]. There is no information regarding the use of SGK1 inhibitors in AML. However, these combinations could also be effective given that *WT1* is only expressed in committed myeloid precursors and is typically absent in healthy and leukemic stem cells. We intend to investigate *SGK1* targeting as a treatment for malignancies with increased *WT1* levels. Due to *SGK1* action during hematopoietic differentiation, SGK1 inhibitors could also be therapeutically valuable for diseases with defective hematopoiesis.

In conclusion, *WT1* represses *SGK1* in AML cells. Consequently, leukemic cells are more resistant to apoptosis under stress conditions. Future studies are warranted to assess if the therapeutic targeting of the WT1/SGK1 pair could help induce leukemia cell maturation or apoptosis.

## Supplementary Information

Below is the link to the electronic supplementary material.

Supplementary file1 (DOCX 515 KB)

Supplementary file2 (PDF 390 KB)

Supplementary file3 (PDF 32 KB)

Supplementary file4 (PDF 62 KB)

Supplementary file5 (PDF 427 KB)

Supplementary file6 (PDF 30 KB)

## Data Availability

No datasets were generated or analysed during the current study.
