# Supplementary figures and images for: SGK1 repression by WT1 may confer a survival advantage to leukemic cells under stress conditions
Source: Ann Hematol. 2025 Jul 4;104(7):3655–67. doi: 10.1007/s00277-025-06458-z (PMC12334445; doi:10.1007/s00277-025-06458-z)

**Figure 2c**


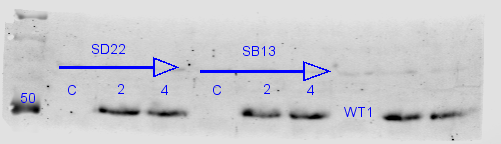


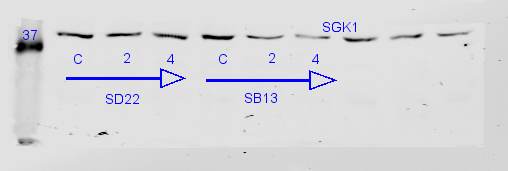


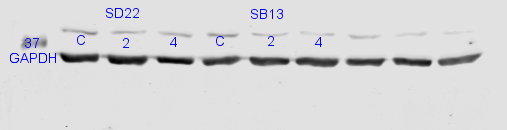


**Figure 3e**


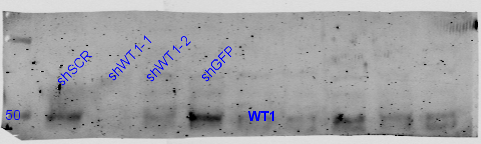


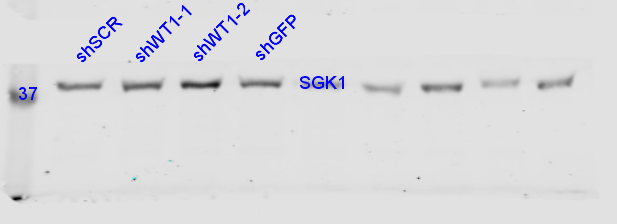


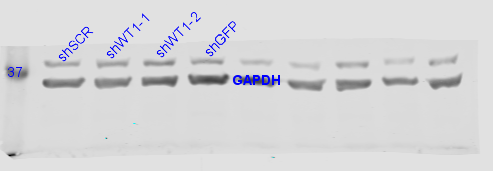


**Figure 3f**


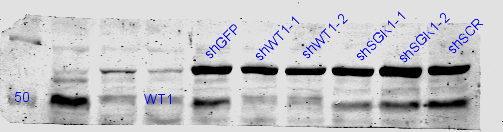


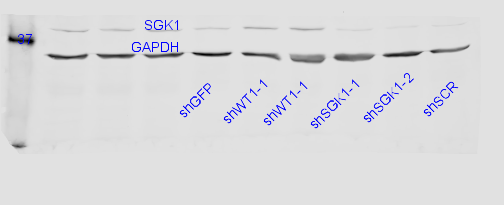


**Figure 6 b**


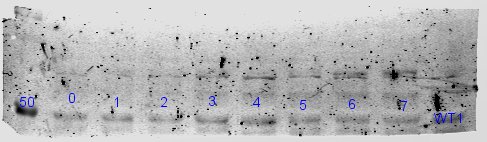


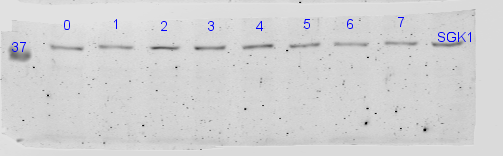


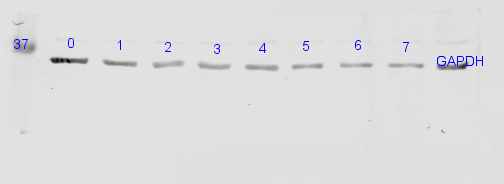


**Figure 6f**


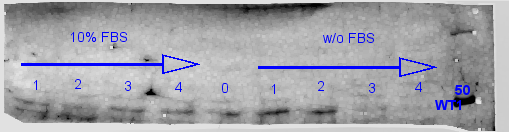


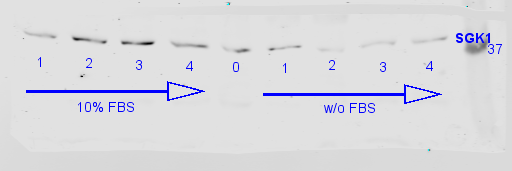


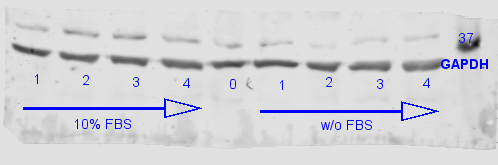


**Supplementary Figure 5a**


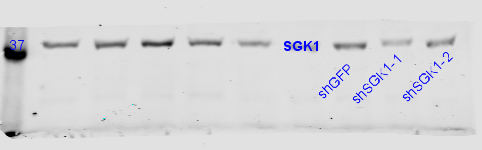


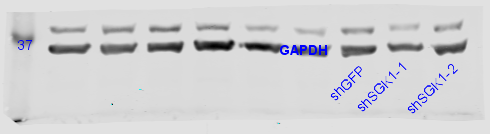

Supplement: Supplementary file 1 — Supplementary file1 (DOCX 515 KB) [file 277_2025_6458_MOESM1_ESM.docx]
