# Supplementary material for: SGK1 repression by WT1 may confer a survival advantage to leukemic cells under stress conditions
Source: Ann Hematol. 2025 Jul 4;104(7):3655–67. doi: 10.1007/s00277-025-06458-z (PMC12334445; doi:10.1007/s00277-025-06458-z)

SGK1 repression by *WT1* may confer a survival advantage to leukemic cells under stress conditions.

Authors: Miguel A. Rubio[1], Sabina Cisa[1], Ana Mozos[2], Helena Castellet[1], Elena Bussaglia[1], Maite Carricondo[1], María Isabel Hernández-Alvarez[2], Jorge Sierra[1], Josep F. Nomdedéu[1].

From the Laboratory of Hematology, Department of Hematology Hospital de la Santa Creu i Sant Pau. Institut Josep Carreras. Universitat Autònoma de Barcelona/IIB Sant Pau[1] and Departament de Bioquímica i Biomedicina Molecular, Facultat de Biologia, Universitat de Barcelona [2].SPAIN

Supplementary Information

Suppl Methods

Analysis of Global Gene Expression Profiles and TCGA Database Analysis

Microarray statistical analysis was performed in the R/Bioconductor. CEL files were read with an oligo package (2). The RMA algorithm performed preprocessing and summarization at the core transcript cluster level of annotation. For differential gene expression, we used the limma package (3). See Supplementary Tables 3 for a list of significant differentially expressed genes.

CEL files from 183 AML patient samples were obtained from The Cancer Genome Atlas (TCGA) database (TCGA Research Network: http://cancergenome.nih.gov/), and the 7 CEL files of patients with higher and lower wild-type *WT1* expression were selected (Supplementary Table 2) and analyzed as above.

Statistical methods

Statistical analyses were performed using GraphPad Prism Software 6.0 (San Diego, CA, USA). Unless stated otherwise, experimental data are represented as the mean (SD) of one representative experiment of three biological replicates. For comparisons, Student t-tests were performed as appropriate. Level of statistical significance: $*P < 0.05$; $**P < 0.01$; $***P < 0.001$.

Plasmids and chemicals

Reagents: Tetracycline hydrochloride, stock 1mg/ml in water, used one µg/ml; puromycin dihydrochloride, stock 10 mg/ml in water, used one µg/ml; all-trans retinoic acid (ATRA), stock five mM in ethanol, used one µM; phorbol 12-myristate 13-acetate (PMA), stock 1mM in DMSO, used 100 nM,

were purchased from Sigma (Sigma-Aldrich, St. Louis, MO, USA). EMD638683 SGK1 inhibitor was dissolved at ten mM in DMSO and used 10-50 µM (HY-15193 MedChem Express, Monmouth Junction, NJ, USA).

WT1 cDNA expressing plasmids *pCMV-CB6-WT1B* and *pCMV-CB6-WT1D* expressing the *WT1* isoforms (+/-) and (+/+), respectively, were kindly provided by Dr. Rene Bernards (NKI, Amsterdam, Netherlands). The retroviral plasmids pMIG, pMWIG(+/-), and pMWIG(delZ). were obtained by a generous gift from Dr. Urban Gullberg (Lund University, Sweden) (4). Vector *pCMV-CB6*++ excised *WT1*(+/+) from vector *pCMV-CB6-WT1D* with *HindIII* and *AflII,* plus blunting and religation. Vector *pCMV-CB6-WT1delZ* was constructed by cutting vector *pCMV-CB6-WT1D (EcoRI/ HindIII)* and introducing an *EcoRI/ HindIII* fragment with *WT1(delZ)* mutant from pMWIG(delZ). All constructs were verified by sequencing.

Cell culture and transfection

All cell lines used in this study were obtained from the American Type Culture Collection (Manassas, VA, USA) or the Leibniz Institute DSMZ-German Collection of Microorganisms and Cell Cultures (Leibniz, Germany). K562, NB4, and THP-1 cells were cultured in RPMI 1640 medium (Gibco, Karlsruhe, Germany) supplemented with 10% FBS, 100 mg/ml streptomycin, 100 units/ml penicillin, and two mM L-glutamine (all from Gibco). HEK293T, 293FT, Phoenix-AMPHO, U2OS, Saos-2 cells, and the WT1 tetracycline-inducible clones (a generous gift of Dr. Cristoph Englert) were maintained in Dulbecco's Modified Eagle's Medium (DMEM) (Gibco) supplemented with 10% FBS in the presence of penicillin, streptomycin, and L-glutamine. 293FT cells (Thermo Fisher Scientific, Darmstadt, Germany) were supplemented with 1X nonessential amino acids, one mM sodium pyruvate, and 2 mg/ml G418 (all from Gibco). The *WT1* tetracycline-inducible clones were supplemented with 1 mg/ml tetracycline and, for *WT1* induction, were washed three times with 1X PBS and resuspended in a medium without tetracycline. HEL, SKM-1 and MV-4-11 cells were cultured in RPMI 1640 medium supplemented with 10% or 20% of FBS in the presence of penicillin and streptomycin. OCI-AML-3 cells were maintained in 80-90% alpha-MEM (with ribs- and deoxyribonucleosides) medium with 20% FBS, penicillin, and streptomycin. MV-4-11 cells were cultured in a DMEM medium supplemented with 20% FBS, penicillin, and streptomycin.

For transient transfection, K562 cells were nucleofected (Nucleofector I Device) with the Lonza Nucleofector Kits V (Lonza, Basel, Switzerland), following the manufacturer's instructions. HEK293T, 293FT, and Phoenix-AMPHO cells were transfected using Lipofectamine 2000 (Invitrogen).

Immunohistochemical studies

We analyzed the expression of WT1 and SGK1 in 44 AML patients by immunohistochemistry on formalin-fixed paraffin-embedded tissue sections with a primary monoclonal antibody against WT1 (WT-1 MxH 6FH2 clone, DAKO) and SGK1 (D27C11; Cell Signalling Technology, Danvers, MA, USA). The staining intensity was graded on a 0 to 3 scale (0, negative; 1, weak; 2, moderate; and 3, intense staining), and the percentage of stained cells was scored. Both values (staining intensity and percentage of positive cells) were multiplied to obtain the final score. Cases with a score under ten were considered negative. The relationship between quantitative variables was evaluated by non-parametric correlation analysis (Spearman correlation test). A two-sided p-value under 0.05 was considered significant.

RNA isolation, reverse transcription, semiquantitative PCR and RT-qPCR

Total RNA was isolated with the QIAamp RNA Blood Mini Kit (Qiagen, Hilden, Germany). For semi-quantitative PCR and RT-qPCR, one µg RNA was reverse-transcribed in a 20 µl reaction using random hexamers and MuLV Reverse Transcriptase (Applied Biosystems). Subsequent PCR reactions were performed with 0.4 µl of the reverse transcription reaction in a 25 µl volume. Inducible mouse *WT1*-transcript was amplified using the forward 5′- AATGCGCCCTACCTGCCCA -3′ and reverse 5′-CCGTCGAAAGTGACCGTGCTGTAT -3′ primers (5) and GoTaq G2 Flexi DNA Polymerase (Promega, San Luis Obispo, CA) for 25 cycles (denaturation 95°C, 15 s; annealing 60°C, 30 s; extension 72°C, 30 s) on a GeneAmp PCR System 9700 (Applied Biosystems)

For the detection of *SGK1* 1 to 4 isoforms, we used the five primers described elsewhere (6). Cells were infected with shRNA lentivirus and puromycin selected for seven days before RNA isolation. PCR was performed for 40 cycles (denaturation 95°C, 30 s; annealing and extension 60°C, 40 s). Primers for *GAPDH* were forward (266-282) 5'-ATGCTGGCGCTGAGTAC-3' and reverse (523-506) 5'-TGAGTCCTTCCACGATAC-3' (258 nt band) for 20 cycles. The PCR products were analyzed on

a QIAxcel device (Qiagen). Cell- and reverse transcriptase-free samples were used as negative controls.

Real-time quantitative PCR (RT-qPCR) reactions were performed in triplicate with 0.4 µl of the reverse transcription reaction in a 10 µl volume in a 7500 Real-Time PCR System (Applied Biosystems). *WT1* and *ABL1* expression levels and *WT1* copy-number titration were determined as previously reported (1). The probes for the *SGK1* (Hs00985033_g1) and *GUSB* (Hs00939627_m1) were purchased as Assay-on-Demand (Applied Biosystems). Gene expression data were analyzed by the *ΔΔCt me*thod, using *ABL1*, *GUSB*, or both as internal controls. The efficacy of the PCR amplifications of controls and tests was identical; parallelism of the control and test standard curves was confirmed.

Western blot analysis

Protein lysates were prepared, and immunoblotting was performed as previously described (7). Briefly, cell lysates were prepared by extraction with RIPA buffer (Sigma). 35 µg of protein were analyzed by SDS-PAGE and transferred onto nitrocellulose membranes. The following primary antibodies were used: anti-*WT1* 1/100 (F-6; Santa Cruz Biotechnology, Santa Cruz, CA, USA), anti-*SGK1* 1/1000 (D27C11; Cell Signalling Technology, Danvers, MA, USA), and anti-GAPDH 1/24000 (MAB374; Millipore, Burlington, MA, USA), followed by IRDye Fluorescent Secondary Antibodies, washing and Odyssey CLx Imaging System scanning (LI-COR Biotechnology, Lincoln, NE, USA), following the manufacturer's instructions. For densitometry, Image Studio Lite 4.0.21 software was used. Quantification was performed by densitometry, and expression values were normalized to corresponding GAPDH. Normalized values of proteins, relative to the amount of control (set to 1), are shown.

Flow-cytometric analysis of cell surface antigens, apoptosis, and cell cycle distribution

Cells were seeded in 6-well plates at the indicated concentrations, treated with ATRA and PMA, and assayed by flow cytometry at the indicated times.

To analyze cell surface antigens, cells were washed in PBS and resuspended to 500,000 cells/ml. At room temperature, 100µl of the cell suspension was incubated with 10 µl of mAb for 15 min. The cells were washed and resuspended in BD FACSFlow Sheath fluid before flow cytometric analysis using a BD FACSCanto II flow cytometer and the FACSDiva Software v6.1.2 (BD Biosciences, San

Jose, CA). The following monoclonal antibodies (mAb) were used for labeling cell samples: control IgG1-FITC and IgG1-PE, CD11b-PE, CD11c-PE, and CD61-FITC (Becton Dickinson, San Jose, CA). Ten thousand viable events were acquired from each tube. Dead cells and debris were excluded from analysis by gating before calculating the percentage of positive cells. All of the samples were assayed in triplicate.

For apoptosis studies, cells were harvested and double-stained with FITC-annexin V and Propidium iodide (PI), using FITC Annexin V Apoptosis Detection Kit I (BD Pharmingen) following the manufacturer's protocol. Cells were classified into viable cells, dead cells, early apoptotic cells, and apoptotic cells. Alternatively, to assay total apoptosis, 100 µl cells were incubated in cytometer tubes with 2 µl of 250 µg/ml PI (Sigma) in the dark and 15 min later assayed directly by flow cytometry. The fraction of PI-positive apoptotic cells was expressed as a percentage of total cells. Measurements were performed in triplicate.

Staining with PI was used for cell-cycle analysis. Briefly, 300 µl cells were washed in PBS and resuspended in 100 µl of cell cycle buffer (NaCitrate 0.1%, NP-40 0.03%, PI 50 µg/ml). Samples were incubated in the dark for 15 min at room temperature, and flow cytometric analysis was performed. Cell cycle phase distribution, i.e., the G0 + G1, S, and G2 + M nuclei, percentages of the analyzed cell population, was determined using ModFit LT v3.3 software.

Supplemental References

1. Nomdedéu JF, Hoyos M, Carricondo M, Bussaglia E, Estivill C, Esteve J *et al.* Bone marrow WT1 levels at diagnosis, post-induction and post-intensification in adult de novo AML. *Leukemia* 2013; **27**(11): 2157-64.

2. Carvalho BS, Irizarry, RA. A framework for Oligonucleotide Microarray Preprocessing. *Bioinformatics* 2010, **26**(19): 2363-7.

3. Ritchie ME, Phipson B, Wu D, Hu Y, Law CW, Shi W *et al.* limma powers differential expression analyses for RNA-sequencing and microarray studies. *Nucleic Acids Res* 2015; **43**(7): e47

4. Svedberg H, Richter J, Gullberg U. Forced expression of the Wilms tumor 1 (WT1) gene inhibits proliferation of human hematopoietic CD34(+) progenitor cells. *Leukemia* 2001; **15**(12): 1914-22.

5. Dallosso AR, Hancock AL, Malik S, Salpekar A, King-Underwood L, Pritchard-Jones K *et al* Alternately spliced WT1 antisense transcripts interact with WT1 sense RNA and show epigenetic and splicing defects in cancer. *RNA* 2007; **13**(12): 2287-99.

6. Burgon J, Robertson AL, Sadiku P, Wang X, Hooper-Greenhill E, Prince LR *et al.* Serum and glucocorticoid-regulated kinase 1 regulates neutrophil clearance during inflammation resolution. *J Immunol* 2014; **192**(4): 1796-805.

7. Menendez JA, Rubio MA, Campisi J, Lupu R. Heregulin, a new regulator of telomere length in human cells. *Oncotarget* 2015; **6**(37): 39422-36.

8. Busch M, Schwindt H, Brandt A, Beier M, Görldt N, Romaniuk P *et al.* Classification of a frameshift/extended and a stop mutation in WT1 as gain-of-function mutations that activate cell cycle genes and promote Wilms tumour cell proliferation. *Hum Mol Genet* 2014; **23**(15): 3958-74.

9. Wang Y, Xiao M, Chen X, Chen L, Xu Y, Lv L *et al.* WT1 recruits TET2 to regulate its target gene expression and suppress leukemia cell proliferation. *Mol Cell* 2015; **57**(4): 662-73.

10. Sommer EM, Dry H, Cross D, Guichard S, Davies BR, Alessi DR. Elevated SGK1 predicts resistance of breast cancer cells to Akt inhibitors. *Biochem J* 2013; **452**(3): 499-508.

Supplementary Figures

Supplementary Figure 1. WT1 mRNA knockdown levels, as determined by RT-qPCR, after lentiviral infection of control shSCR or shWT1(hairpins 1 or 2) and puromycin selection in K562 cells (left, see Figure 3c) and NB4 cells (right, see Figure 3d). ABL1 mRNA expression was used as a control. One representative experiment out of three. The results are mean (SD).

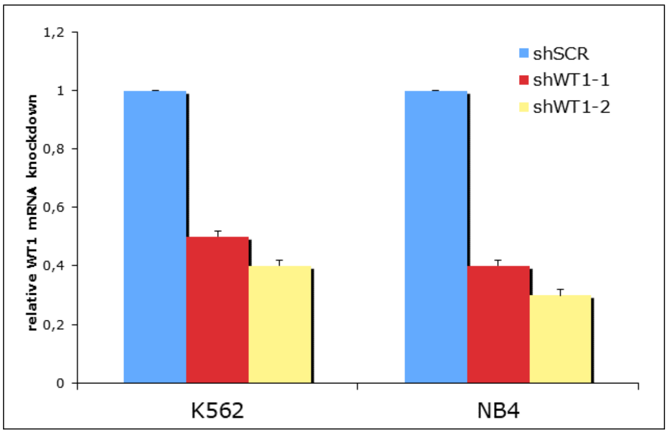

Supplementary Figure 2. (**A**) Bloodspot analysis of WT1 and SGK1 expression in normal human hematopoietic lineages (dataset: Normal hematopoiesis with AMLs). (**B**) Bloodspot analysis of WT1 (right) and SGK1 expression (left) in human AML subtypes (dataset: BloodPool: AML samples with normal cells). (**C**) Flow cytometric analysis of CD11c expression (or an isotype-matched control) in NB4 cells after treatment with 1 mM ATRA for 4 days and untreated control. The histogram shows representative fluorescence values of one in three different experiments performed. (**D**) RT-qPCR analysis of *SGK1* mRNA levels in K562 cells infected with control retrovirus (C) or cells overexpressing WT1(+/-) after PMA treatment (mean (SD)). Internal control is ABL1 and day 0 value is set to 1. (**E**) Flow cytometric analysis of CD61 expression in K562 cells infected with control (shSCR) or *WT1* (shWT1) or *SGK1* (shSGK1) knockdowns and treated with PMA for the indicated times. Data is the mean (SD) of three distinct experiments.

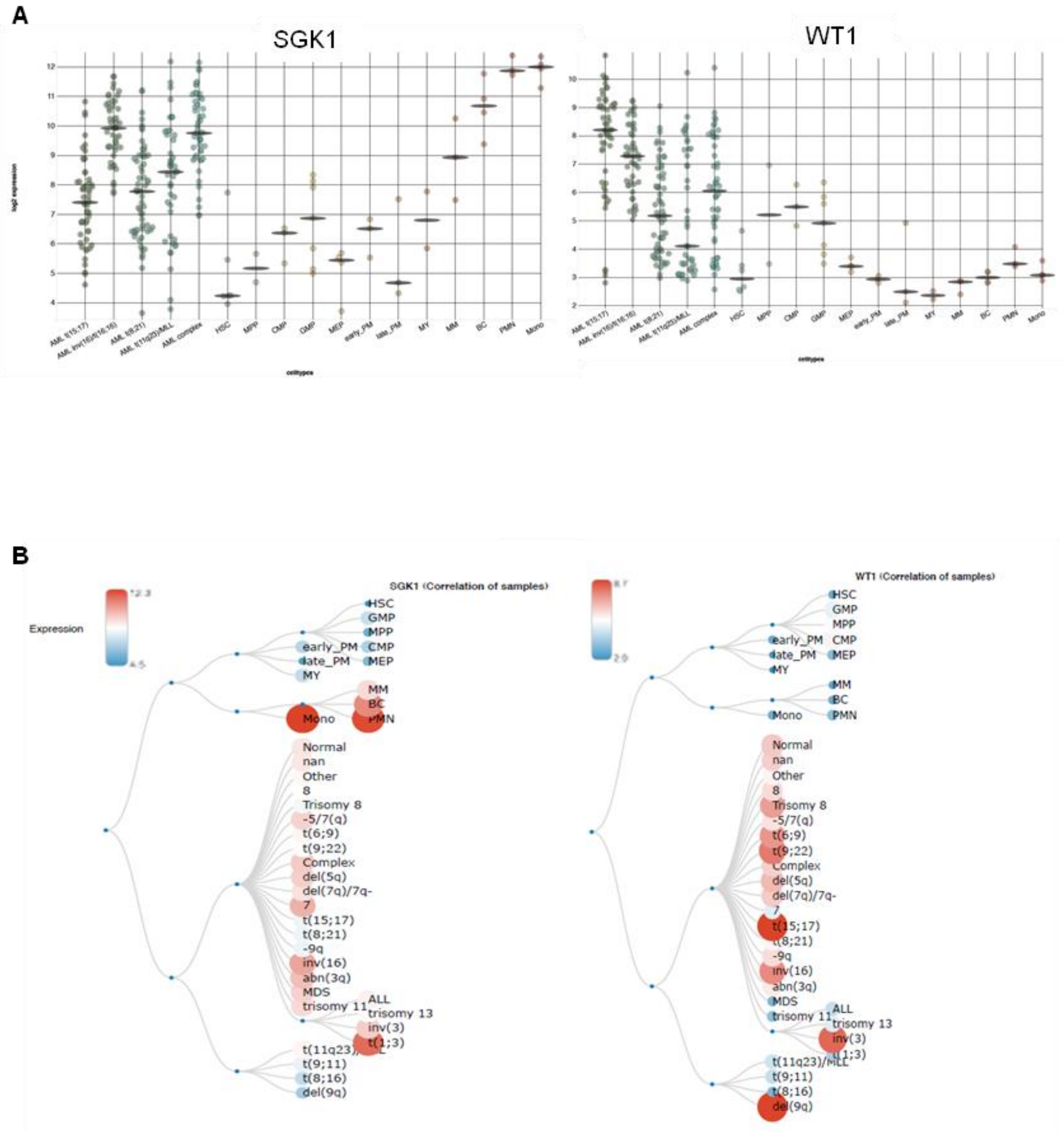
A
SGK1
WT1
log2 expression
AML t(15;17)
AML inv(16)/t(16;16)
AML t(8;21)
AML t(11q23)/MLL
AML complex
HSC
MPP
CMP
GMP
MEP
early_PM
late_PM
MY
MM
BC
PMN
Mono
celltypes
B
Expression
SGK1 (Correlation of samples)
WT1 (Correlation of samples)
HSC
GMP
MPP
early_PM
CMP
late_PM
MEP
MY
MM
BC
Mono
PMN
Normal
nan
Other
8
Trisomy 8
-5/7(q)
t(6;9)
t(9;22)
Complex
del(5q)
del(7q)/7q-
7
t(15;17)
t(8;21)
-9q
inv(16)
abn(3q)
MDS
trisomy 11
ALL
trisomy 13
inv(3)
t(1;3)
t(11q23)/MLL
t(9;11)
t(8;16)
del(9q)

C

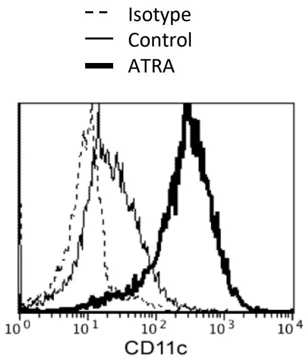
Isotype
Control
ATRA
Event count
10^0
10^1
10^2
10^3
10^4
CD11c

D

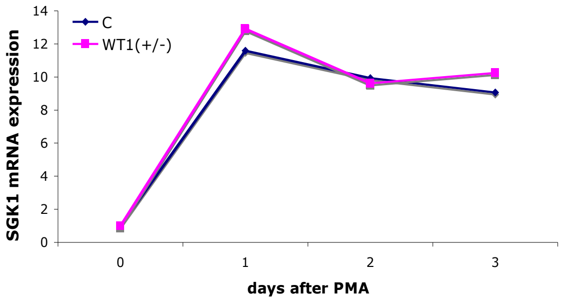
C
WT1(+/-)
SGK1 mRNA expression
14
12
10
8
6
4
2
0
0
1
2
3
days after PMA

E

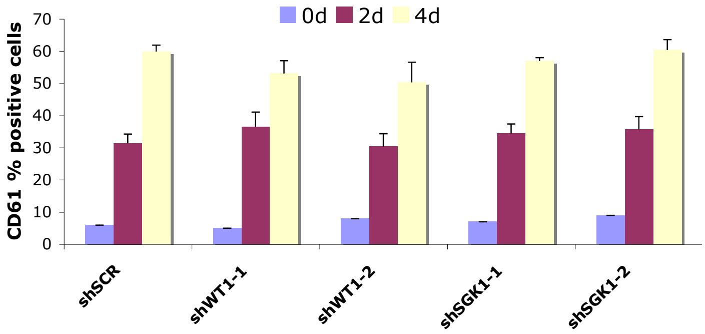
0d
2d
4d
CD61 % positive cells
70
60
50
40
30
20
10
0
shSCR
shWT1-1
shWT1-2
shSGK1-1
shSGK1-2

Supplementary Figure 3. (**A**) Effect of SGK1 inhibition on NB4 cell viability during starvation. Cells were plated at 150000 cel/ml, treated, and left undisturbed for the indicated times. Treatment: Vehicle (Veh), EMD 638683 (EMD), non-starving, medium changed at day 3 (NoSt). Viable cells were determined by flow cytometry after staining with propidium iodide (PI). (**B**) RT-qPCR analysis of *WT1* and *SGK1* mRNA levels in NB4 cells without medium change. Internal control is ABL1, and the day 0 value is set to 1. One representative experiment out of two is shown. (**C**) RT-qPCR analysis of *WT1* and *SGK1* mRNA levels in K562 cells without medium change. Cells were seeded at 300000 cel/ml and grown for the indicated times (mean (SD)). Internal control is ABL1. (**D-E**) Fasting-refeeding experiments. RT-qPCR analysis of *WT1* and *SGK1* mRNA levels (mean(SD)) of NB4 (**D**) or K562 cells (**E**) cultured without FBS for three days. Then, an FBS of 10% was added, and cells were collected at the indicated times. Internal control is ABL1, and the time 0 value is set to 1. One representative experiment out of two is shown. (**F**) Induction of apoptosis in NB4 (up) or K562 (down) cells, serum starved for 3 or 4 days, respectively (right panels). Control cells were grown with 10% FBS (left panels). FACS analysis was done after labeling with annexin V-FITC (FITC-A) and PI (PE-A). One representative experiment out of three is shown. (**G-H**) RT-qPCR analysis (mean (SD)) of *WT1* and *SGK1* mRNA levels in NB4 (**G**) or K562 cells (**H**) cultured in medium without FBS for the indicated times (n=2). Internal control is ABL1 for NB4 and GUSB for K562, and day 0 value is set to 1.

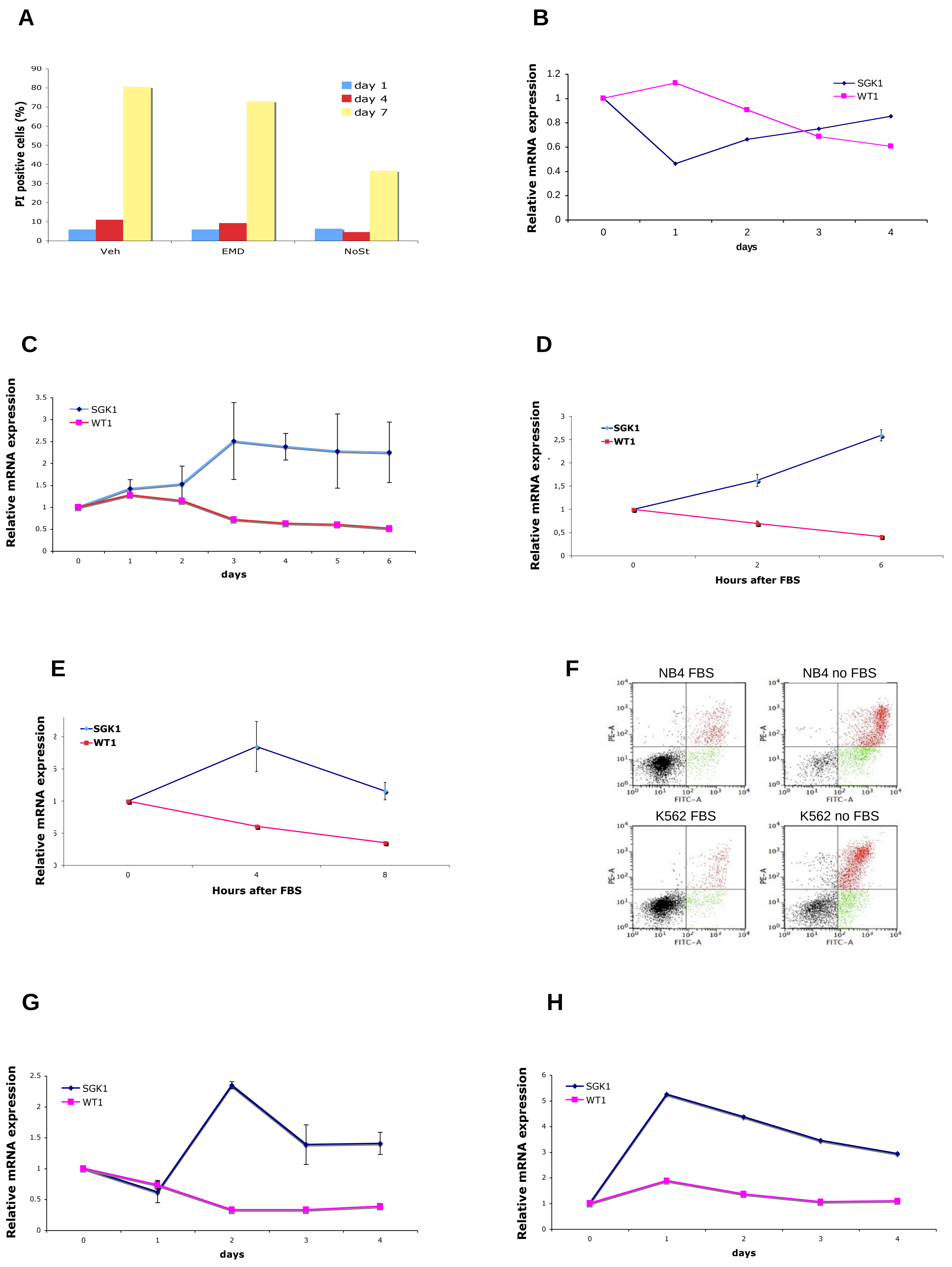
A
PI positive cells (%)
day 1
day 4
day 7
Veh
EMD
NoSt
B
Relative mRNA expression
SGK1
WT1
days
C
Relative mRNA expression
SGK1
WT1
days
D
Relative mRNA expression
SGK1
WT1
Hours after FBS
E
Relative mRNA expression
SGK1
WT1
Hours after FBS
F
NB4 FBS
NB4 no FBS
K562 FBS
K562 no FBS
PE-A
FITC-A
G
Relative mRNA expression
SGK1
WT1
days
H
Relative mRNA expression
SGK1
WT1
days

Supplementary Figure 4. (**A**) Immunoblot of SGK1 protein levels in K562 cells infected with lentiviral control (*shGFP*) or knockdowns for *SGK1* (*shSGK1-1* or *-2*). GAPDH protein was used as an internal control. The positions of prestained molecular mass markers are indicated to the right. (**B**) Cell cycle analysis of NB4 cells plated at 150000 cel/ml, treated with vehicle (VEH) or SGK1 inhibitor (EMD), and growing for 24 hours in the presence (FBS) or absence (NOF) of FBS. The percentage of cells in each phase of the cell cycle was determined through PI staining and FACS analysis of the DNA content. One representative experiment out of two is shown.

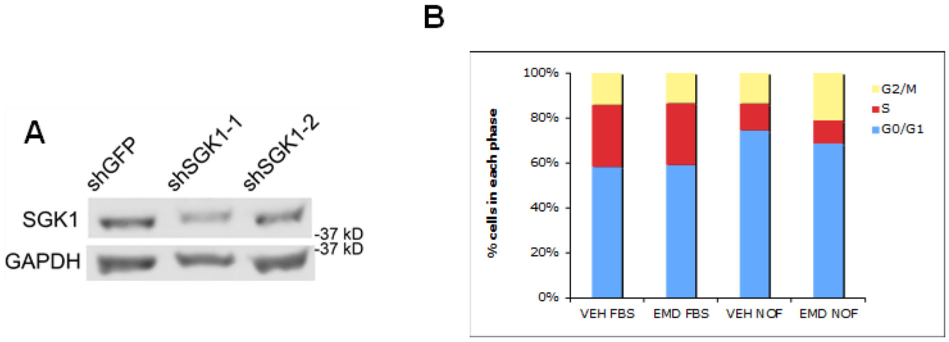

Supplement: Supplementary file 2 — Supplementary file2 (PDF 390 KB) [file 277_2025_6458_MOESM2_ESM.pdf]
