# Supplementary material for: SGK1 repression by WT1 may confer a survival advantage to leukemic cells under stress conditions
Source: Ann Hematol. 2025 Jul 4;104(7):3655–67. doi: 10.1007/s00277-025-06458-z (PMC12334445; doi:10.1007/s00277-025-06458-z)
Supplement: Supplementary file 3 — Supplementary file3 (PDF 32 KB) [file 277_2025_6458_MOESM3_ESM.pdf]

Supplementary Table 1: Clinical and biological characteristics of the AML patients included in the microarray studies.

Affymetrix GeneChip® HG-U133 Plus 2.0 microarrays

Patient #	WT1 status	Sex	Age (years)	⌈WT1/ABL1⌉ x 10^4 WT1 copies	Diagnosis	Cytogenetics	Molecular
1	WT1 low	M		137	AML	NA	NA
2	WT1 low	M	63	76.6	AML	46,XY / 46,XY,9q-	FLT3- CEBPA- MLL-
3	WT1 low	M	30	5	AML	46,XY	FLT3- NPM- CEBPA- MLL-
4	WT1 low	F		168	AML	46,XX	FLT3- NPM+ CEBPA- MLL-
5	WT1 low	F		3	AML	47,XX,+8,t(9;11)[18]/46,XX	FLT3- NPM- CEBPA-
6	WT1 high	F	25	43750	AML	46,XX	FLT3+ NPM+CEBPA - MLL-
7	WT1 high	F	47	6545	AML	46,XX/46,XX,t(8;21)(q22;q22),t(10;19)(q[illegible]	FLT3- NPM- CEBPA- MLL-
8	WT1 high	M	28	3750	AML	NA	FLT3- NPM- CEBPA- MLL-
9	WT1 high	M		32432	AML	47,XY,+11,i(11)(q10)[18]/46,XY	FLT3- NPM- CEBPA- MLL+
10	WT1 high	M		25161	AML	46,XY	FLT3- NPM- CEBPA- MLL+
11	WT1 high	M	45	15365	AML	nd	FLT3- NPM- CEBPA- MLL+
12	WT1 high	M		8750	AML	normal	FLT3+ NPM- CEBPA - MLL-
13	WT1 high	M	55	6000	AML	46,XY	FLT3+ NPM+CEBPA - MLL-
14	WT1 high	F		8333	AML	normal	FLT3+ NPM+CEBPA - MLL-

Affymetrix GeneChip® Human Exon 1.0 ST microarrays

Patient #	WT1 status	Sex	Age (years)	⌈WT1/ABL1⌉ x 10^4 WT1 copies	Diagnosis	Cytogenetics	Molecular
1	WT1 low	F	60	23.8	AML	normal	FLT3+ NPM- CEBPA - MLL-
2	WT1 low	F	51	83.3	AML	normal	FLT3- NPM+ CEBPA- MLL-
3	WT1 low	M	67	20	AML	normal	FLT3- NPM- CEBPA- MLL-
4	WT1 high	M		8750	AML	normal	FLT3+ NPM- CEBPA- MLL-
5	WT1 high	F	44	28461	AML	normal	FLT3+ NPM+ CEBPA- MLL-
