# Supplementary material for: SGK1 repression by WT1 may confer a survival advantage to leukemic cells under stress conditions
Source: Ann Hematol. 2025 Jul 4;104(7):3655–67. doi: 10.1007/s00277-025-06458-z (PMC12334445; doi:10.1007/s00277-025-06458-z)
Supplement: Supplementary file 4 — Supplementary file4 (PDF 62 KB) [file 277_2025_6458_MOESM4_ESM.pdf]

Supplementary Table 2. Selected The Cancer Genome Atlas microarrays of AML patients for the analysis of WT1 (low level) vs WT1 (high level) differentially expressed genes.

Microarray number	WT1 level	TCGA Patient ID	WT1 mutation	Arrays used in the analysis
TCGA-AB-2910-03A-01R-0757-21.CEL	2.495049912	2910		WT1 low
TCGA-AB-2829-03A-01R-0757-21.CEL	2.676324805	2829		WT1 low
TCGA-AB-2805-03A-01R-0757-21.CEL	2.72087013	2805		WT1 low
TCGA-AB-2833-03A-01R-0757-21.CEL	2.752092503	2833		WT1 low
TCGA-AB-2866-03A-01R-0757-21.CEL	2.897146326	2866		WT1 low
TCGA-AB-2861-03A-01R-0757-21.CEL	2.940984167	2861		WT1 low
TCGA-AB-2883-03A-01R-0757-21.CEL	2.964480388	2883		WT1 low
TCGA-AB-2987-03A-01R-0757-21.CEL	3.074790764	2987		
TCGA-AB-2873-03A-01R-0757-21.CEL	3.076773015	2873		
TCGA-AB-2857-03A-01R-0757-21.CEL	3.104579585	2857		
TCGA-AB-2916-03A-01R-0757-21.CEL	3.179898982	2916		
TCGA-AB-2807-03A-01R-0757-21.CEL	3.197135767	2807		
TCGA-AB-2974-03A-01R-0757-21.CEL	3.329199054	2974		
TCGA-AB-2995-03A-01R-0757-21.CEL	3.446724305	2995		
TCGA-AB-2933-03A-01R-0757-21.CEL	3.518560861	2933		
TCGA-AB-2969-03A-01R-0757-21.CEL	3.525436113	2969		
TCGA-AB-2978-03A-01R-0757-21.CEL	3.552545819	2978		
TCGA-AB-2894-03A-01R-0757-21.CEL	3.573732821	2894		
TCGA-AB-2825-03A-01R-0757-21.CEL	3.61291341	2825		
TCGA-AB-2948-03A-01R-0757-21.CEL	3.622301851	2948		
TCGA-AB-2813-03A-01R-0757-21.CEL	3.64057269	2813		
TCGA-AB-2832-03A-01R-0757-21.CEL	3.744078497	2832		
TCGA-AB-2891-03A-01R-0757-21.CEL	3.861325207	2891		
TCGA-AB-2934-03A-01R-0757-21.CEL	3.901561391	2934		
TCGA-AB-3005-03A-01R-0757-21.CEL	3.901690474	3005		
TCGA-AB-2893-03A-01R-0757-21.CEL	3.904199499	2893		
TCGA-AB-2887-03A-01R-0757-21.CEL	3.90698397	2887		
TCGA-AB-2928-03A-01R-0757-21.CEL	3.907480216	2928		
TCGA-AB-2835-03A-01R-0757-21.CEL	3.910061403	2835		
TCGA-AB-2847-03A-01R-0757-21.CEL	3.986338636	2847		
TCGA-AB-2973-03A-01R-0757-21.CEL	4.042916026	2973		
TCGA-AB-2865-03A-01R-0757-21.CEL	4.106256047	2865		
TCGA-AB-2981-03B-01R-0757-21.CEL	4.467493003	2981		
TCGA-AB-2968-03A-01R-0757-21.CEL	4.491455371	2968		
TCGA-AB-2854-03A-01R-0757-21.CEL	4.543941863	2854		
TCGA-AB-2814-03A-01R-0757-21.CEL	4.576963888	2814		
TCGA-AB-2927-03A-01R-0757-21.CEL	4.599088088	2927		
TCGA-AB-2888-03B-01R-0757-21.CEL	4.683422079	2888		
TCGA-AB-3002-03A-01R-0757-21.CEL	4.73315654	3002		
TCGA-AB-2925-03A-01R-0757-21.CEL	4.911464481	2925		
TCGA-AB-2985-03A-01R-0757-21.CEL	4.962187892	2985		
TCGA-AB-2917-03A-01R-0757-21.CEL	4.984347584	2917		
TCGA-AB-2959-03A-01R-0757-21.CEL	5.165917795	2959		
TCGA-AB-2922-03A-01R-0757-21.CEL	5.176215107	2922		
TCGA-AB-2920-03A-01R-0757-21.CEL	5.272634316	2920		
TCGA-AB-2872-03A-01R-0757-21.CEL	5.309435764	2872	yes	
TCGA-AB-2848-03A-01R-0757-21.CEL	5.317542961	2848		
TCGA-AB-2932-03A-01R-0757-21.CEL	5.383406188	2932		
TCGA-AB-2817-03A-01R-0757-21.CEL	5.408603339	2817		
TCGA-AB-2858-03A-01R-0757-21.CEL	5.48858721	2858		
TCGA-AB-2950-03A-01R-0757-21.CEL	5.530663549	2950		
TCGA-AB-2890-03A-01R-0757-21.CEL	5.564647923	2890		
TCGA-AB-2967-03A-01R-0757-21.CEL	5.592017195	2967		
TCGA-AB-3008-03A-01R-0757-21.CEL	5.597434941	3008		
TCGA-AB-2937-03A-01R-0757-21.CEL	5.670548203	2937		
TCGA-AB-2808-03A-01R-0757-21.CEL	5.745728078	2808	yes	
TCGA-AB-2837-03A-01R-0757-21.CEL	5.76761983	2837		
TCGA-AB-2946-03A-01R-0757-21.CEL	5.830964051	2946		
TCGA-AB-2875-03A-01R-0757-21.CEL	5.878731431	2875		
TCGA-AB-2939-03A-01R-0757-21.CEL	5.896484615	2939		
TCGA-AB-3000-03A-01R-0757-21.CEL	5.919173068	3000		

TCGA-AB-2955-03A-01R-0757-21.CEL	5.93949921	2955	
TCGA-AB-2908-03A-01R-0757-21.CEL	5.943005147	2908	
TCGA-AB-2997-03B-01R-0757-21.CEL	6.013761324	2997	
TCGA-AB-2898-03A-01R-0757-21.CEL	6.015831571	2898	
TCGA-AB-2824-03A-01R-0757-21.CEL	6.044427364	2824	
TCGA-AB-2900-03A-01R-0757-21.CEL	6.062187968	2900	
TCGA-AB-2912-03A-01R-0757-21.CEL	6.066602055	2912	
TCGA-AB-2956-03A-01R-0757-21.CEL	6.144429102	2956	
TCGA-AB-2851-03A-01R-0757-21.CEL	6.177955967	2851	
TCGA-AB-2836-03A-01R-0757-21.CEL	6.207614081	2836	
TCGA-AB-2816-03A-01R-0757-21.CEL	6.255688066	2816	
TCGA-AB-2945-03A-01R-0757-21.CEL	6.276989699	2945	
TCGA-AB-2977-03A-01R-0757-21.CEL	6.287536733	2977	
TCGA-AB-2952-03A-01R-0757-21.CEL	6.294931877	2952	
TCGA-AB-2996-03A-01R-0757-21.CEL	6.295524344	2996	
TCGA-AB-2947-03A-01R-0757-21.CEL	6.306582936	2947	
TCGA-AB-2845-03B-01R-0757-21.CEL	6.322184363	2845	
TCGA-AB-2806-03A-01R-0757-21.CEL	6.359223093	2806	
TCGA-AB-2924-03A-01R-0757-21.CEL	6.40820918	2924	
TCGA-AB-2905-03A-01R-0757-21.CEL	6.466201214	2905	p.T377fs
TCGA-AB-2966-03A-01R-0757-21.CEL	6.480178996	2966	
TCGA-AB-2941-03A-01R-0757-21.CEL	6.481781772	2941	
TCGA-AB-2843-03A-01R-0757-21.CEL	6.499770229	2843	
TCGA-AB-2826-03A-01R-0757-21.CEL	6.520731775	2826	
TCGA-AB-2880-03A-01R-0757-21.CEL	6.521641626	2880	
TCGA-AB-2938-03A-01R-0757-21.CEL	6.535747798	2938	
TCGA-AB-2867-03A-01R-0757-21.CEL	6.548911856	2867	
TCGA-AB-2949-03B-01R-0757-21.CEL	6.560647561	2949	
TCGA-AB-2936-03A-01R-0757-21.CEL	6.613318547	2936	
TCGA-AB-2828-03A-01R-0757-21.CEL	6.630934407	2828	
TCGA-AB-2856-03A-01R-0757-21.CEL	6.654475729	2856	
TCGA-AB-2818-03A-01R-0757-21.CEL	6.663186121	2818	
TCGA-AB-2846-03A-01R-0757-21.CEL	6.694803281	2846	p.A382fs
TCGA-AB-2971-03A-01R-0757-21.CEL	6.770541689	2971	
TCGA-AB-2944-03A-01R-0757-21.CEL	6.780411993	2944	
TCGA-AB-2957-03A-01R-0757-21.CEL	6.807952074	2957	
TCGA-AB-2860-03A-01R-0757-21.CEL	6.812630759	2860	
TCGA-AB-2859-03A-01R-0757-21.CEL	6.840392326	2859	
TCGA-AB-2812-03A-01R-0757-21.CEL	6.843348688	2812	
TCGA-AB-2855-03A-01R-0757-21.CEL	6.845646637	2855	
TCGA-AB-2899-03A-01R-0757-21.CEL	6.850289807	2899	
TCGA-AB-2965-03A-01R-0757-21.CEL	6.865414667	2965	
TCGA-AB-2884-03A-01R-0757-21.CEL	6.873746561	2884	
TCGA-AB-2878-03A-01R-0757-21.CEL	6.886060193	2878	
TCGA-AB-2864-03A-01R-0757-21.CEL	6.892691012	2864	
TCGA-AB-2844-03A-01R-0757-21.CEL	6.942834153	2844	p.S381fs
TCGA-AB-2930-03A-01R-0757-21.CEL	6.950767777	2930	p.V370fs,p.R370fs
TCGA-AB-2810-03A-01R-0757-21.CEL	6.9919691	2810	
TCGA-AB-2921-03A-01R-0757-21.CEL	7.02028891	2921	
TCGA-AB-2911-03A-01R-0757-21.CEL	7.020633346	2911	
TCGA-AB-2868-03A-01R-0757-21.CEL	7.02775213	2868	
TCGA-AB-2963-03A-01R-0757-21.CEL	7.04740498	2963	
TCGA-AB-2943-03A-01R-0757-21.CEL	7.054697536	2943	yes
TCGA-AB-2882-03A-01R-0757-21.CEL	7.102205022	2882	
TCGA-AB-2983-03A-01R-0757-21.CEL	7.105425695	2983	yes
TCGA-AB-2940-03A-01R-0757-21.CEL	7.140549349	2940	
TCGA-AB-2819-03A-01R-0757-21.CEL	7.147398849	2819	
TCGA-AB-2881-03A-01R-0757-21.CEL	7.150493599	2881	
TCGA-AB-2989-03A-01R-0757-21.CEL	7.19681943	2989	p.Y402fs
TCGA-AB-2970-03A-01R-0757-21.CEL	7.197584231	2970	e9-1
TCGA-AB-2935-03A-01R-0757-21.CEL	7.239450999	2935	
TCGA-AB-2901-03A-01R-0757-21.CEL	7.246028107	2901	
TCGA-AB-2838-03A-01R-0757-21.CEL	7.280599112	2838	yes
TCGA-AB-2827-03A-01R-0757-21.CEL	7.30486835	2827	
TCGA-AB-2885-03A-01R-0757-21.CEL	7.315464322	2885	
TCGA-AB-2821-03A-01R-0757-21.CEL	7.332125547	2821	
TCGA-AB-2853-03A-01R-0757-21.CEL	7.395909815	2853	
TCGA-AB-2915-03A-01R-0757-21.CEL	7.403417683	2915	
TCGA-AB-3009-03A-01R-0757-21.CEL	7.413759306	3009	p.A382fs

TCGA-AB-2926-03A-01R-0757-21.CEL	7.436955656	2926		
TCGA-AB-2886-03A-01R-0757-21.CEL	7.445190246	2886		
TCGA-AB-2876-03A-01R-0757-21.CEL	7.453130684	2876		
TCGA-AB-2964-03A-01R-0757-21.CEL	7.475083605	2964	yes	
TCGA-AB-2954-03A-01R-0757-21.CEL	7.507632518	2954		
TCGA-AB-2820-03A-01R-0757-21.CEL	7.520362822	2820	yes	
TCGA-AB-2923-03A-01R-0757-21.CEL	7.533154085	2923		
TCGA-AB-2975-03A-01R-0757-21.CEL	7.533870576	2975		
TCGA-AB-2841-03A-01R-0757-21.CEL	7.567104283	2841		
TCGA-AB-2863-03A-01R-0757-21.CEL	7.575488637	2863		
TCGA-AB-2892-03A-01R-0757-21.CEL	7.596633936	2892		
TCGA-AB-2822-03A-01R-0757-21.CEL	7.610527692	2822		
TCGA-AB-2830-03A-01R-0757-21.CEL	7.646822041	2830		
TCGA-AB-2942-03A-01R-0757-21.CEL	7.653523526	2942		
TCGA-AB-2909-03A-01R-0757-21.CEL	7.656375531	2909		
TCGA-AB-2918-03A-01R-0757-21.CEL	7.688902343	2918		
TCGA-AB-2999-03B-01R-0757-21.CEL	7.72273985	2999		
TCGA-AB-2889-03A-01R-0757-21.CEL	7.741569544	2889		
TCGA-AB-2913-03A-01R-0757-21.CEL	7.758189781	2913	e7+1	
TCGA-AB-2979-03A-01R-0757-21.CEL	7.75945657	2979		
TCGA-AB-2903-03A-01R-0757-21.CEL	7.763414664	2903		
TCGA-AB-2840-03A-01R-0757-21.CEL	7.763795585	2840		
TCGA-AB-2990-03B-01R-0757-21.CEL	7.806135825	2990		
TCGA-AB-2870-03A-01R-0757-21.CEL	7.856386545	2870		
TCGA-AB-2976-03A-01R-0757-21.CEL	7.856630572	2976	p.A382fs	
TCGA-AB-2874-03A-01R-0757-21.CEL	7.894402691	2874	p.R462W	
TCGA-AB-2998-03A-01R-0757-21.CEL	7.938254271	2998		
TCGA-AB-2972-03A-01R-0757-21.CEL	8.003876187	2972		
TCGA-AB-2988-03B-01R-0757-21.CEL	8.01074333	2988		
TCGA-AB-3011-03A-01R-0757-21.CEL	8.038190398	3011		
TCGA-AB-2823-03A-01R-0757-21.CEL	8.039408763	2823		
TCGA-AB-2904-03A-01R-0757-21.CEL	8.048159587	2904		
TCGA-AB-2914-03A-01R-0757-21.CEL	8.064420689	2914		
TCGA-AB-2896-03B-01R-0757-21.CEL	8.091881806	2896		
TCGA-AB-2879-03A-01R-0757-21.CEL	8.139060787	2879		
TCGA-AB-2877-03A-01R-0757-21.CEL	8.14600729	2877		
TCGA-AB-2919-03A-01R-0757-21.CEL	8.16553403	2919	p.H465Y	
TCGA-AB-3007-03A-01R-0757-21.CEL	8.190505398	3007		
TCGA-AB-2984-03A-01R-0757-21.CEL	8.233481846	2984		
TCGA-AB-2871-03A-01R-0757-21.CEL	8.235653449	2871		
TCGA-AB-2803-03A-01R-0757-21.CEL	8.239011167	2803		
TCGA-AB-3006-03A-01R-0757-21.CEL	8.251232547	3006		
TCGA-AB-2986-03A-01R-0757-21.CEL	8.326936249	2986		
TCGA-AB-2897-03A-01R-0757-21.CEL	8.395656766	2897		
TCGA-AB-3001-03A-01R-0757-21.CEL	8.402605368	3001		
TCGA-AB-2931-03A-01R-0757-21.CEL	8.404044383	2931		
TCGA-AB-2869-03A-01R-0757-21.CEL	8.406731307	2869		WT1 high
TCGA-AB-2982-03B-01R-0757-21.CEL	8.514429149	2982		WT1 high
TCGA-AB-2895-03A-01R-0757-21.CEL	8.636543274	2895		WT1 high
TCGA-AB-3012-03A-01R-0757-21.CEL	8.642937078	3012		WT1 high
TCGA-AB-2980-03A-01R-0757-21.CEL	8.657852429	2980		WT1 high
TCGA-AB-2994-03A-01R-0757-21.CEL	8.690526373	2994		WT1 high
TCGA-AB-2862-03A-01R-0757-21.CEL	8.753294105	2862		WT1 high
