# Supplementary material for: SGK1 repression by WT1 may confer a survival advantage to leukemic cells under stress conditions
Source: Ann Hematol. 2025 Jul 4;104(7):3655–67. doi: 10.1007/s00277-025-06458-z (PMC12334445; doi:10.1007/s00277-025-06458-z)
Supplement: Supplementary file 5 — Supplementary file5 (PDF 427 KB) [file 277_2025_6458_MOESM5_ESM.pdf]

Supplementary Table 3A. Affymetrix GeneChip® Human Genome U133 Plus 2.0 Arrays. Top upregulated and top downregulated genes
Analysis of AML patients with low WT1 expression vs. high WT1 expression, ranked by significance (P Value < 0.05).

PROBEID	ENTREZID	SYMBOL	GENENAME	logFC	P.Value
206067_s_at	7490	WT1	Wilms tumor 1	-4.15749319	1.4028E-07
205349_at	2769	GNA15	guanine nucleotide binding protein (G protein), alpha 15 (Gq class)	-1.95746717	5.5071E-05
207830_s_at	5511	PPP1R8	protein phosphatase 1, regulatory subunit 8	-1.26842621	6.7577E-05
225685_at	10602	CDC42EP3	CDC42 effector protein (Rho GTPase binding) 3	2.18023904	6.7796E-05
209710_at	2624	GATA2	GATA binding protein 2	-2.6574335	9.1263E-05
207857_at	11027	LILRA2	leukocyte immunoglobulin-like receptor, subfamily A (with TM domain), member 2	1.77940206	0.00025034
209286_at	10602	CDC42EP3	CDC42 effector protein (Rho GTPase binding) 3	1.94482336	0.00029439
1555279_at	25852	ARMC8	armadillo repeat containing 8	-1.61714935	0.00031137
225314_at	132299	OCIAD2	OCIA domain containing 2	-1.39486193	0.00036143
211240_x_at	528016 | 1500	TMX2-CTNND1 | CTNN	TMX2-CTNND1 readthrough (NMD candidate) | catenin (cadherin-associated protein),	-1.27012842	0.00061807
222763_s_at	55339 | 84826	WDR33 | SFT2D3	WD repeat domain 33 | SFT2 domain containing 3	-1.40082478	0.00062156
205048_s_at	8781 | 5723	PSPHP1 | PSPH	phosphoserine phosphatase pseudogene 1 | phosphoserine phosphatase	3.16665932	0.00064346
202703_at	8446	DUSP11	dual specificity phosphatase 11 (RNA/RNP complex 1-interacting)	-1.22696754	0.00075868
218314_s_at	55216	C11orf57	chromosome 11 open reading frame 57	-0.95993073	0.00077277
228330_at	221302	ZUFSP	zinc finger with UFM1-specific peptidase domain	-1.11523499	0.00082924
223289_s_at	84640	USP38	ubiquitin specific peptidase 38	-1.47536316	0.00087082
212837_at	23172	FAM175B	family with sequence similarity 175, member B	-1.27768559	0.00087567
209774_x_at	2920	CXCL2	chemokine (C-X-C motif) ligand 2	-2.89497971	0.00091315
1553808_a_at	159296	NKX2-3	NK2 homeobox 3	-3.53886395	0.00096314
216338_s_at	25844	YIPF3	Yip1 domain family, member 3	-1.11950436	0.00096676
209259_s_at	9126	SMC3	structural maintenance of chromosomes 3	-1.22321165	0.00099605
224598_at	11282	MGAT4B	mannosyl (alpha-1,3-)-glycoprotein beta-1,4-N-acetylglucosaminyltransferase, isozyr	-1.25406611	0.0010526
222467_s_at	55291	PPP6R3	protein phosphatase 6, regulatory subunit 3	-1.05206479	0.00106349
220112_at	79722	ANKRD55	ankyrin repeat domain 55	1.92702327	0.0011515
203334_at	1659	DHX8	DEAH (Asp-Glu-Ala-His) box polypeptide 8	-0.89449495	0.00119318
222344_at	NA	NA	NA	1.90728951	0.00121247
209085_x_at	5981	RFC1	replication factor C (activator 1) 1, 145kDa	-0.86186615	0.00121859
205453_at	3212	HOXB2	homeobox B2	-2.76848295	0.00123792
210999_s_at	2887	GRB10	growth factor receptor-bound protein 10	-2.18545883	0.00137569
202227_s_at	10902	BRD8	bromodomain containing 8	-1.22878079	0.0013926
209160_at	8644	AKR1C3	aldo-keto reductase family 1, member C3	-3.08845022	0.00146973
235019_at	1368	CPM	carboxypeptidase M	1.70446839	0.00149442
237747_at	100507281	LOC100507281	uncharacterized LOC100507281	1.81823811	0.00150485
207850_at	2921	CXCL3	chemokine (C-X-C motif) ligand 3	-2.43781003	0.00151492
209288_s_at	10602	CDC42EP3	CDC42 effector protein (Rho GTPase binding) 3	1.6551313	0.00151845
241681_at	NA	NA	NA	1.73980636	0.0015397
229174_at	285237	C3orf38	chromosome 3 open reading frame 38	-1.33328595	0.00160088
200985_s_at	966	CD59	CD59 molecule, complement regulatory protein	-2.59485427	0.00161084
212015_x_at	5 | 100616459	PTBP1 | MIR4745	polypyrimidine tract binding protein 1 | microRNA 4745	-0.92966965	0.0016214
203593_at	23607	CD2AP	CD2-associated protein	-1.82857224	0.0016954
214086_s_at	10038	PARP2	poly (ADP-ribose) polymerase 2	-1.11869543	0.00176804
201830_s_at	10276	NET1	neuroepithelial cell transforming 1	-1.68220184	0.0018493
210732_s_at	3964	LGALS8	lectin, galactoside-binding, soluble, 8	-1.56525117	0.00188064
202521_at	10664	CTCF	CCCTC-binding factor (zinc finger protein)	-0.86753941	0.00189209
201938_at	8099	CDK2AP1	cyclin-dependent kinase 2 associated protein 1	-1.00996512	0.00191059
211270_x_at	5725	PTBP1	polypyrimidine tract binding protein 1	-0.8852749	0.00197314
212440_at	11017	SNRNP27	small nuclear ribonucleoprotein 27kDa (U4/U6.U5)	-0.96314205	0.00202259
223221_at	6341	SCO1	SCO1 cytochrome c oxidase assembly protein	-0.77088211	0.00202605
1564154_at	NA	NA	NA	1.23784238	0.00202946
203299_s_at	8905	AP1S2	adaptor-related protein complex 1, sigma 2 subunit	1.36061847	0.00205004
209167_at	2824	GPM6B	glycoprotein M6B	3.2031664	0.00205859
209033_s_at	1859	DYRK1A	dual-specificity tyrosine-(Y)-phosphorylation regulated kinase 1A	-0.83221561	0.00209647
210944_s_at	825	CAPN3	calpain 3, (p94)	1.62236158	0.00212268
1568768_s_at	100302650	BRE-AS1	BRE antisense RNA 1	1.78961259	0.00212707
203688_at	5311	PKD2	polycystic kidney disease 2 (autosomal dominant)	-1.65338155	0.0021414
229971_at	221188	ADGRG5	adhesion G protein-coupled receptor G5	-1.98655014	0.00216676
200049_at	11143	KAT7	K(lysine) acetyltransferase 7	-1.09883929	0.00225567
202832_at	9648	GCC2	GRIP and coiled-coil domain containing 2	-1.04335015	0.00225916
203588_s_at	7029	TFDP2	transcription factor Dp-2 (E2F dimerization partner 2)	-1.64139983	0.0022765
209030_s_at	23705	CADM1	cell adhesion molecule 1	1.95168262	0.00233798
239106_at	340591	CA5BP1	carbonic anhydrase VB pseudogene 1	0.93437892	0.00237276
215248_at	2887	GRB10	growth factor receptor-bound protein 10	-2.0107194	0.00242252
212229_s_at	23014	FBXO21	F-box protein 21	-1.91177168	0.00243243
226323_at	91603	ZNF830	zinc finger protein 830	-1.15338744	0.00243714
211063_s_at	4690	NCK1	NCK adaptor protein 1	-1.50565403	0.00249105
225121_at	55773	TBC1D23	TBC1 domain family, member 23	-1.08096783	0.00252386
203589_s_at	7029	TFDP2	transcription factor Dp-2 (E2F dimerization partner 2)	-1.53737901	0.00254738
212072_s_at	1457	CSNK2A1	casein kinase 2, alpha 1 polypeptide	-0.76579239	0.00261678
219443_at	55617	TASP1	taspase, threonine aspartase, 1	-1.35543064	0.00263047
206310_at	6691	SPINK2	serine peptidase inhibitor, Kazal type 2 (acrosin-trypsin inhibitor)	-3.58770663	0.00268007
202819_s_at	6924	TCEB3	transcription elongation factor B (SIII), polypeptide 3 (110kDa, elongin A)	-0.75049891	0.00269213
204568_at	22863	ATG14	autophagy related 14	-1.24829191	0.00269757
220367_s_at	79595	SAP130	Sin3A-associated protein, 130kDa	-0.77176993	0.0027058
1555274_a_at	85465	EPT1	ethanolaminephosphotransferase 1	-1.12513917	0.00273045
224151_s_at	50808	AK3	adenylate kinase 3	-1.06351441	0.00274068
207483_s_at	55832	CAND1	cullin-associated and neddylation-dissociated 1	-0.86006173	0.00280077
202623_at	55837	EAPP	E2F-associated phosphoprotein	-0.93031056	0.00285486
204739_at	1060	CENPC	centromere protein C	-1.12057079	0.00289225
226524_at	285237	C3orf38	chromosome 3 open reading frame 38	-1.34560868	0.00289882
204401_at	3783	KCNN4	potassium channel, calcium activated intermediate/small conductance subfamily N al	-1.21692431	0.00293593
202377_at	54741	LEPROT	leptin receptor overlapping transcript	-1.42999511	0.00293931
223298_s_at	51251	NT5C3A	5'-nucleotidase, cytosolic IIIA	-1.25496376	0.00295889
209128_s_at	9733	SART3	squamous cell carcinoma antigen recognized by T cells 3	-1.00799102	0.00298292
204809_at	10845	CLPX	caseinolytic mitochondrial matrix peptidase chaperone subunit	-0.85238515	0.00298748
208021_s_at	5981	RFC1	replication factor C (activator 1) 1, 145kDa	-0.951486	0.00302803
205883_at	7704	ZBTB16	zinc finger and BTB domain containing 16	-2.21662261	0.00308445
222203_s_at	5 | 100526794	RDH14 | NT5C1B-RDH	retinol dehydrogenase 14 (all-trans/9-cis/11-cis) | NT5C1B-RDH14 readthrough	-0.86376325	0.00311032
239237_at	100506776	TRG-AS1	T cell receptor gamma locus antisense RNA 1	-1.72578209	0.003131
212438_at	11017	SNRNP27	small nuclear ribonucleoprotein 27kDa (U4/U6.U5)	-1.12789434	0.00314416
227141_at	127253	TYW3	tRNA-yW synthesizing protein 3 homolog (S. cerevisiae)	-0.92962558	0.00321686
210664_s_at	7035	TFPI	tissue factor pathway inhibitor (lipoprotein-associated coagulation inhibitor)	-2.51366246	0.00324932
210358_x_at	2624	GATA2	GATA binding protein 2	-0.95718239	0.0033465
212421_at	23313	KIAA0930	KIAA0930	1.09242369	0.00343184
201392_s_at	3482	IGF2R	insulin-like growth factor 2 receptor	1.08439569	0.00343795
209930_s_at	4778	NFE2	nuclear factor, erythroid 2	-2.39187472	0.00347015
209112_at	1027	CDKN1B	cyclin-dependent kinase inhibitor 1B (p27, Kip1)	-1.13081007	0.00352362
218535_s_at	55781	RIOK2	RIO kinase 2	-0.84011595	0.00355599
243509_at	NA	NA	NA	1.35075198	0.00362163

202732_at	11142	PKIG	protein kinase (cAMP-dependent, catalytic) inhibitor gamma	-1.37392141	0.00363779
236558_at	NA	NA	NA	1.84099449	0.00364073
221432_s_at	81894	SLC25A28	solute carrier family 25 (mitochondrial iron transporter), member 28	-0.86012378	0.0036823
225799_at	1471 | 112597	MIR4435-1HG | LINC0	MIR4435-1 host gene | long intergenic non-protein coding RNA 152	-1.53074902	0.00372705
201700_at	896	CCND3	cyclin D3	-1.12112193	0.00372873
214039_s_at	55353	LAPTM4B	lysosomal protein transmembrane 4 beta	-2.5372402	0.00377937
226102_at	7745	ZKSCAN8	zinc finger with KRAB and SCAN domains 8	-1.42149716	0.00381227
221007_s_at	81608	FIP1L1	factor interacting with PAPOLA and CPSF1	-0.95431164	0.00381968
224655_at	50808	AK3	adenylate kinase 3	-0.81619615	0.00388372
209282_at	25865	PRKD2	protein kinase D2	-1.05547108	0.0038842
226888_at	53944	CSNK1G1	casein kinase 1, gamma 1	-1.02942282	0.00389736
204071_s_at	10210	TOPORS	topoisomerase I binding, arginine/serine-rich, E3 ubiquitin protein ligase	-1.09032863	0.00393989
204689_at	3087	HHEX	hematopoietically expressed homeobox	-1.54002245	0.00404193
202798_at	10427	SEC24B	SEC24 family member B	-0.96873019	0.00409468
215411_s_at	10758	TRAF3IP2	TRAF3 interacting protein 2	-1.41361659	0.00413199
209255_at	23008	KLHDC10	kelch domain containing 10	-1.35305763	0.00415156
208029_s_at	55353	LAPTM4B	lysosomal protein transmembrane 4 beta	-1.88228759	0.00415302
230048_at	3475	IFRD1	interferon-related developmental regulator 1	1.2262264	0.00420167
221704_s_at	79720	VPS37B	vacuolar protein sorting 37 homolog B (S. cerevisiae)	-0.99246173	0.00420488
215424_s_at	22938	SNW1	SNW domain containing 1	-0.90080852	0.00421312
224671_at	124995	MRPL10	mitochondrial ribosomal protein L10	-0.84714827	0.00425519
204929_s_at	10791	VAMP5	vesicle-associated membrane protein 5	1.20648895	0.00429549
202189_x_at	5 | 100616459	PTBP1 | MIR4745	polypyrimidine tract binding protein 1 | microRNA 4745	-0.76842754	0.00430563
221535_at	55341	LSG1	large 60S subunit nuclear export GTPase 1	-0.91967047	0.00445577
205664_at	22944	KIN	Kin17 DNA and RNA binding protein	-0.9947991	0.00448197
218806_s_at	10451	VAV3	vav 3 guanine nucleotide exchange factor	-1.20960589	0.00449975
212463_at	966	CD59	CD59 molecule, complement regulatory protein	-1.7123317	0.00450893
201084_s_at	9774	BCLAF1	BCL2-associated transcription factor 1	-0.80117476	0.00453372
219634_at	50515	CHST11	carbohydrate (chondroitin 4) sulfotransferase 11	-1.16861967	0.00464802
201519_at	9868	TOMM70A	translocase of outer mitochondrial membrane 70 homolog A (S. cerevisiae)	-0.67057775	0.00469655
227762_at	NA	NA	NA	-2.06757184	0.00473732
203300_x_at	8905	AP1S2	adaptor-related protein complex 1, sigma 2 subunit	1.17914071	0.00474558
218929_at	55602	CDKN2AIP	CDKN2A interacting protein	-0.8716391	0.00493076
203640_at	10150	MBNL2	muscleblind-like splicing regulator 2	-1.54958555	0.00494568
1562194_at	NA	NA	NA	1.40156099	0.00499701
226683_at	112574	SNX18	sorting nexin 18	1.08339254	0.00506274
213110_s_at	1287	COL4A5	collagen, type IV, alpha 5	-3.06443213	0.00507933
223117_s_at	55031	USP47	ubiquitin specific peptidase 47	-0.75634839	0.00509869
224819_at	90843	TCEAL8	transcription elongation factor A (SII)-like 8	-1.29364692	0.005141
208656_s_at	10983	CCNI	cyclin I	-0.65458416	0.0051578
208854_s_at	8428	STK24	serine/threonine kinase 24	-1.26879596	0.00519264
217952_x_at	23469	PHF3	PHD finger protein 3	-0.89874981	0.00525518
206493_at	3674	ITGA2B	integrin, alpha 2b (platelet glycoprotein IIb of IIb/IIIa complex, antigen CD41)	-1.55499291	0.00528627
212835_at	23172	FAM175B	family with sequence similarity 175, member B	-1.0486289	0.00531156
221675_s_at	56994	CHPT1	choline phosphotransferase 1	-1.02652452	0.00536628
222623_s_at	51193	ZNF639	zinc finger protein 639	-0.97830683	0.00537978
202537_s_at	25978	CHMP2B	charged multivesicular body protein 2B	-1.10966018	0.00543238
242343_x_at	NA	NA	NA	1.49972412	0.00545779
203016_s_at	117178	SSX2IP	synovial sarcoma, X breakpoint 2 interacting protein	-1.54785894	0.00546102
1559882_at	25939	SAMHD1	SAM domain and HD domain 1	2.40736595	0.00546778
206494_s_at	3674	ITGA2B	integrin, alpha 2b (platelet glycoprotein IIb of IIb/IIIa complex, antigen CD41)	-1.82278817	0.00548075
204301_at	9920	KBTBD11	kelch repeat and BTB (POZ) domain containing 11	2.1449786	0.00552841
214259_s_at	8574	AKR7A2	aldo-keto reductase family 7, member A2 (aflatoxin aldehyde reductase)	1.30373569	0.00554751
1556657_at	NA	NA	NA	1.43069308	0.00560588
215933_s_at	3087	HHEX	hematopoietically expressed homeobox	-1.93561939	0.00560753
229695_at	NA	NA	NA	1.99568355	0.00564188
202356_s_at	2962	GTF2F1	general transcription factor IIF, polypeptide 1, 74kDa	-0.82936304	0.00568081
202218_s_at	9415	FADS2	fatty acid desaturase 2	-1.05213435	0.00568585
217821_s_at	51729	WBP11	WW domain binding protein 11	-1.09493836	0.00570136
209333_at	8408	ULK1	unc-51 like autophagy activating kinase 1	-1.01257646	0.00579336
200828_s_at	7756	ZNF207	zinc finger protein 207	-0.86491348	0.00582984
226976_at	23633	KPNA6	karyopherin alpha 6 (importin alpha 7)	-0.67359904	0.00586787
1556352_at	NA	NA	NA	1.3584516	0.00587204
201717_at	740	MRPL49	mitochondrial ribosomal protein L49	-0.91344142	0.00589657
206100_at	1368	CPM	carboxypeptidase M	1.61498339	0.00590126
219433_at	54880	BCOR	BCL6 corepressor	-1.02743511	0.00590437
203085_s_at	7040	TGFB1	transforming growth factor, beta 1	-1.3061012	0.00592677
212246_at	90411	MCFD2	multiple coagulation factor deficiency 2	-0.98131544	0.00594104
219276_x_at	79886	CAAP1	caspase activity and apoptosis inhibitor 1	-1.14153938	0.00598079
235811_at	NA	NA	NA	1.74246736	0.00598336
226157_at	7029	TFDP2	transcription factor Dp-2 (E2F dimerization partner 2)	-1.33365424	0.00601094
223059_s_at	83641	FAM107B	family with sequence similarity 107, member B	1.78548484	0.00602429
209174_s_at	54870	QRICH1	glutamine-rich 1	-0.72003045	0.00602825
209362_at	2 | 101928625	MED21 | LOC1019286	mediator complex subunit 21 | uncharacterized LOC101928625	-1.52603644	0.00605658
1556543_at	NA	NA	NA	1.54370332	0.006133
219378_at	79612	NAA16	N(alpha)-acetyltransferase 16, NatA auxiliary subunit	-0.62165055	0.00613965
211297_s_at	1022	CDK7	cyclin-dependent kinase 7	-1.05102688	0.00614193
228854_at	NA	NA	NA	-2.26295758	0.00621563
204150_at	23166	STAB1	stabilin 1	-1.87249318	0.00623295
217906_at	23588	KLHDC2	kelch domain containing 2	-0.86506771	0.006258
210665_at	7035	TFPI	tissue factor pathway inhibitor (lipoprotein-associated coagulation inhibitor)	-2.18649103	0.00628442
201874_at	9019	MPZL1	myelin protein zero-like 1	-1.16447296	0.00632515
200000_s_at	10594	PRPF8	pre-mRNA processing factor 8	-0.83826674	0.00633115
1557810_at	NA	NA	NA	1.35820228	0.00637436
226503_at	55183	RIF1	replication timing regulatory factor 1	-1.16168076	0.00643154
201596_x_at	3875	KRT18	keratin 18, type I	-2.70358222	0.00643523
1552755_at	157983	C9orf66	chromosome 9 open reading frame 66	1.43726623	0.00647403
217987_at	54529	ASNSD1	asparagine synthetase domain containing 1	-0.79694351	0.00650445
212386_at	6925	TCF4	transcription factor 4	-2.73211527	0.00652733
209363_s_at	2 | 101928625	MED21 | LOC1019286	mediator complex subunit 21 | uncharacterized LOC101928625	-1.16874173	0.00655112
223247_at	84246	MED10	mediator complex subunit 10	-0.67926766	0.00665227
227400_at	4784	NFIX	nuclear factor I/X (CCAAT-binding transcription factor)	-1.41914074	0.00669566
1555281_x_at	25852	ARMC8	armadillo repeat containing 8	-1.42236678	0.00675937
200984_s_at	966	CD59	CD59 molecule, complement regulatory protein	-2.05504162	0.0067618
219492_at	26511	CHIC2	cysteine-rich hydrophobic domain 2	-1.12019723	0.00680243
203635_at	10311	DSCR3	Down syndrome critical region 3	-1.0264068	0.0068277
223058_at	83641	FAM107B	family with sequence similarity 107, member B	1.61448945	0.00684119
208948_s_at	6780	STAU1	staufen double-stranded RNA binding protein 1	-0.88868302	0.00686606
204367_at	6668	SP2	Sp2 transcription factor	-1.04272372	0.00690113
204011_at	10253	SPRY2	sprouty homolog 2 (Drosophila)	-1.85237037	0.00696256
1559362_at	NA	NA	NA	1.49431349	0.00696776
223263_s_at	26127	FGFR1OP2	FGFR1 oncogene partner 2	-1.4498794	0.00697663
224968_at	112942	CFAP36	cilia and flagella associated protein 36	-0.79940158	0.00708093
214511_x_at	2210	FCGR1B	Fc fragment of IgG, high affinity Ib, receptor (CD64)	1.92881669	0.0070985

234594_at	319085	ITPK1-AS1	ITPK1 antisense RNA 1	1.51402993	0.00710532
209102_s_at	26959	HBP1	HMG-box transcription factor 1	-1.09934238	0.00712536
200058_s_at	) | 101929240	SNRNP200 | LOC1019	small nuclear ribonucleoprotein 200kDa (U5) | U5 small nuclear ribonucleoprotein 20	-0.59830371	0.00724079
225532_at	91768	CABLES1	Cdk5 and Abl enzyme substrate 1	-1.29790294	0.00727171
221664_s_at	50848	F11R	F11 receptor	-1.13233581	0.00729077
211271_x_at	5 | 100616459	PTBP1 | MIR4745	polypyrimidine tract binding protein 1 | microRNA 4745	-0.89035628	0.00743178
205677_s_at	10301	DLEU1	deleted in lymphocytic leukemia 1 (non-protein coding)	-1.08462738	0.00744691
206034_at	5271	SERPINB8	serpin peptidase inhibitor, clade B (ovalbumin), member 8	1.76347944	0.00747454
218345_at	55365	TMEM176A	transmembrane protein 176A	1.73904697	0.00748026
212101_at	23633	KPNA6	karyopherin alpha 6 (importin alpha 7)	-0.72768699	0.00748592
202946_s_at	22903	BTBD3	BTB (POZ) domain containing 3	-0.93562858	0.00753487
209682_at	868	CBLB	Cbl proto-oncogene B, E3 ubiquitin protein ligase	-1.00917597	0.00753776
226194_at	283489	CHAMP1	chromosome alignment maintaining phosphoprotein 1	-1.19506316	0.00753847
231848_x_at	7756	ZNF207	zinc finger protein 207	0.85684534	0.00761616
224850_at	84896	ATAD1	ATPase family, AAA domain containing 1	-0.68356694	0.00772117
239598_s_at	54947	LPCAT2	lysophosphatidylcholine acyltransferase 2	-1.31686988	0.00773289
202538_s_at	25978	CHMP2B	charged multivesicular body protein 2B	-0.92284055	0.00773734
225378_at	137492	VPS37A	vacuolar protein sorting 37 homolog A (S. cerevisiae)	-1.32133086	0.00774459
205407_at	8434	RECK	reversion-inducing-cysteine-rich protein with kazal motifs	1.72659821	0.00786624
203162_s_at	10300	KATNB1	katanin p80 (WD repeat containing) subunit B 1	-0.71328062	0.00788783
208655_at	10983	CCNI	cyclin I	-0.57786178	0.00790328
213772_s_at	23062	GGA2	golgi-associated, gamma adaptin ear containing, ARF binding protein 2	0.86043811	0.00796149
204957_at	5001	ORC5	origin recognition complex, subunit 5	-1.20558671	0.00801354
216620_s_at	9639	ARHGEF10	Rho guanine nucleotide exchange factor (GEF) 10	1.18171719	0.00803077
201157_s_at	4836	NMT1	N-myristoyltransferase 1	-0.93861312	0.00810888
202527_s_at	4089	SMAD4	SMAD family member 4	-1.27893291	0.00811985
211505_s_at	6780	STAU1	staufen double-stranded RNA binding protein 1	-0.99151415	0.00817684
227522_at	134147	CMBL	carboxymethylenebutenolidase homolog (Pseudomonas)	-1.87701433	0.00818507
216241_s_at	6917	TCEA1	transcription elongation factor A (SII), 1	-0.68397674	0.00820708
203364_s_at	9776	ATG13	autophagy related 13	-0.85801583	0.00832407
202753_at	9861	PSMD6	proteasome (prosome, macropain) 26S subunit, non-ATPase, 6	-0.84978727	0.0084069
226531_at	84876	ORAI1	ORAI calcium release-activated calcium modulator 1	-1.2808506	0.00842404
223566_s_at	54880	BCOR	BCL6 corepressor	-1.37480322	0.00853888
227255_at	149420	PDIK1L	PDLIM1 interacting kinase 1 like	-1.17879865	0.00853958
222163_s_at	79029	SPATA5L1	spermatogenesis associated 5-like 1	-0.91360013	0.00856823
235603_at	3192	HNRNPU	heterogeneous nuclear ribonucleoprotein U (scaffold attachment factor A)	1.40550902	0.00858465
204297_at	5289	PIK3C3	phosphatidylinositol 3-kinase, catalytic subunit type 3	-1.1108931	0.00864793
224435_at	84293	FAM213A	family with sequence similarity 213, member A	1.7799977	0.00866604
226257_x_at	56945	MRPS22	mitochondrial ribosomal protein S22	-0.57276017	0.00869279
218395_at	64431	ACTR6	ARP6 actin-related protein 6 homolog (yeast)	-1.18332394	0.00875274
230006_s_at	258010	SVIP	small VCP/p97-interacting protein	-1.61681912	0.00878924
232693_s_at	5893 | 157574	ZNF395 | FBXO16	zinc finger protein 395 | F-box protein 16	-1.061026	0.00888885
222399_s_at	56889	TM9SF3	transmembrane 9 superfamily member 3	-0.68803152	0.00890916
228904_at	3213	HOXB3	homeobox B3	-3.84195942	0.00897088
202804_at	4363	ABCC1	ATP-binding cassette, sub-family C (CFTR/MRP), member 1	-1.53145505	0.00898467
230720_at	221687	RNF182	ring finger protein 182	-3.03083816	0.00903352
203247_s_at	7572	ZNF24	zinc finger protein 24	-1.02062906	0.00909025
208767_s_at	55353	LAPTM4B	lysosomal protein transmembrane 4 beta	-1.39584764	0.0091264
221265_s_at	81556	VWA9	von Willebrand factor A domain containing 9	-0.72456227	0.00912788
227433_at	205717	KIAA2018	KIAA2018	-1.25330596	0.00917357
224835_at	56261	GPCPD1	glycerophosphocholine phosphodiesterase GDE1 homolog (S. cerevisiae)	0.99108544	0.0091761
201696_at	6429	SRSF4	serine/arginine-rich splicing factor 4	-0.6625282	0.00919289
231205_at	NA	NA	NA	1.49036673	0.00919428
209479_at	25901	CCDC28A	coiled-coil domain containing 28A	-1.06368334	0.00919806
202501_at	10982	MAPRE2	microtubule-associated protein, RP/EB family, member 2	-1.1614551	0.00920247
212131_at	26065	LSM14A	LSM14A, SCD6 homolog A (S. cerevisiae)	-0.77295536	0.0092374
212057_at	23199	GSE1	Gse1 coiled-coil protein	-0.94004817	0.00928827
220189_s_at	11282	MGAT4B	mannosyl (alpha-1,3-)-glycoprotein beta-1,4-N-acetylglucosaminyltransferase, isozyr	-1.13447604	0.0093329
243473_at	NA	NA	NA	1.5024764	0.00934888
216306_x_at	5 | 100616459	PTBP1 | MIR4745	polypyrimidine tract binding protein 1 | microRNA 4745	-0.8555989	0.00938217
202145_at	4061	LY6E	lymphocyte antigen 6 complex, locus E	-1.01504978	0.00939896
217843_s_at	29079	MED4	mediator complex subunit 4	-0.63170018	0.00946291
205600_x_at	3215	HOXB5	homeobox B5	-1.79886017	0.00952116
212696_s_at	6047	RNF4	ring finger protein 4	-0.7915426	0.0095819
212485_at	23131	GPATCH8	G patch domain containing 8	-0.92690247	0.00960636
212351_at	8893	EIF2B5	eukaryotic translation initiation factor 2B, subunit 5 epsilon, 82kDa	-0.61175156	0.00961548
203351_s_at	5000	ORC4	origin recognition complex, subunit 4	-0.97859812	0.00963066
236265_at	6671	SP4	Sp4 transcription factor	-0.89502696	0.00976678
225390_s_at	51621	KLF13	Kruppel-like factor 13	-1.07469795	0.00976991
225296_at	57693	ZNF317	zinc finger protein 317	-0.88788017	0.00986633
242414_at	23475	QPRT	quinolinate phosphoribosyltransferase	-1.25530348	0.00992705
200619_at	10992	SF3B2	splicing factor 3b, subunit 2, 145kDa	-0.59610295	0.00996746
218108_at	55148	UBR7	ubiquitin protein ligase E3 component n-recognin 7 (putative)	-1.2381875	0.00998378
222607_s_at	22894	DIS3	DIS3 exosome endoribonuclease and 3'-5' exoribonuclease	-0.97218972	0.01002345
228655_at	NA	NA	NA	1.41222465	0.01016871
221123_x_at	5893 | 157574	ZNF395 | FBXO16	zinc finger protein 395 | F-box protein 16	-1.16377728	0.01017314
201794_s_at	9887	SMG7	SMG7 nonsense mediated mRNA decay factor	-1.05085367	0.01017343
206207_at	1178	CLC	Charcot-Leyden crystal galectin	-3.22623056	0.01022129
216973_s_at	3217	HOXB7	homeobox B7	-1.94753615	0.01023238
206075_s_at	1457	CSNK2A1	casein kinase 2, alpha 1 polypeptide	-0.8949511	0.01026462
203787_at	23635	SSBP2	single-stranded DNA binding protein 2	-1.79542034	0.01027447
212199_at	114932	MRFAP1L1	Morf4 family associated protein 1-like 1	-1.11289962	0.01027596
228155_at	84293	FAM213A	family with sequence similarity 213, member A	1.79674655	0.01028252
228652_at	284309	ZNF776	zinc finger protein 776	-1.24387709	0.0103504
202890_at	9053	MAP7	microtubule-associated protein 7	-1.95320998	0.01037033
201390_s_at	1460	CSNK2B	casein kinase 2, beta polypeptide	-0.67869146	0.01039887
212145_at	23107	MRPS27	mitochondrial ribosomal protein S27	-0.62633643	0.01040051
201225_s_at	10250	SRRM1	serine/arginine repetitive matrix 1	-0.76456781	0.01040561
226784_at	221830	TWISTNB	TWIST neighbor	-1.26123903	0.0104798
223288_at	84640	USP38	ubiquitin specific peptidase 38	-0.98593675	0.01048114
207320_x_at	6780	STAU1	staufen double-stranded RNA binding protein 1	-0.78042305	0.01051846
225098_at	10152	ABI2	abl-interactor 2	-1.33264867	0.01066336
201997_s_at	23013	SPEN	spen family transcriptional repressor	-0.69183177	0.01072671
217887_s_at	2060	EPS15	epidermal growth factor receptor pathway substrate 15	-0.8379403	0.01076381
202888_s_at	290	ANPEP	alanyl (membrane) aminopeptidase	-1.96521774	0.01085029
209790_s_at	839	CASP6	caspase 6, apoptosis-related cysteine peptidase	-1.45399195	0.01089663
203347_s_at	22823	MTF2	metal response element binding transcription factor 2	-1.12700038	0.01089923
208934_s_at	3964	LGALS8	lectin, galactoside-binding, soluble, 8	-0.8410641	0.01098271
201386_s_at	1665	DHX15	DEAH (Asp-Glu-Ala-His) box helicase 15	-0.95983168	0.0109845
229211_at	285193	DUSP28	dual specificity phosphatase 28	0.59886216	0.01100653
214697_s_at	9991	PTBP3	polypyrimidine tract binding protein 3	-1.17646091	0.01114022
203680_at	5577	PRKAR2B	protein kinase, cAMP-dependent, regulatory, type II, beta	-2.45288482	0.0111426
217954_s_at	23469	PHF3	PHD finger protein 3	-0.80394057	0.01115277
215038_s_at	29072	SETD2	SET domain containing 2	-0.77950356	0.0111684

228348_at	55180	LINS	lines homolog (Drosophila)	-0.79455136	0.01118674
203753_at	6925	TCF4	transcription factor 4	-2.18103439	0.01121544
218558_s_at	54148	MRPL39	mitochondrial ribosomal protein L39	-0.87060631	0.01123106
204752_x_at	10038	PARP2	poly (ADP-ribose) polymerase 2	-0.79685083	0.0112526
222996_s_at	51523	CXXC5	CXXC finger protein 5	-1.35767367	0.01127146
218577_at	55631	LRRC40	leucine rich repeat containing 40	-1.04839512	0.01128862
39248_at	360	AQP3	aquaporin 3 (Gill blood group)	-2.05361318	0.01140204
226638_at	57636	ARHGAP23	Rho GTPase activating protein 23	-0.89996629	0.01145593
209533_s_at	9373	PLAA	phospholipase A2-activating protein	-0.71308609	0.0114802
227936_at	137695	TMEM68	transmembrane protein 68	-1.01732995	0.01150651
222204_s_at	54700	RRN3	RRN3 RNA polymerase I transcription factor homolog (S. cerevisiae)	-1.05435962	0.01154632
208766_s_at	10236	HNRNPR	heterogeneous nuclear ribonucleoprotein R	-0.56296622	0.01158701
213168_at	6670	SP3	Sp3 transcription factor	-0.75738492	0.01162748
209580_s_at	8930	MBD4	methyl-CpG binding domain protein 4	-0.87363814	0.01171563
218277_s_at	79665	DHX40	DEAH (Asp-Glu-Ala-His) box polypeptide 40	-0.76843909	0.01173979
223018_at	28987	NOB1	NIN1/RPN12 binding protein 1 homolog (S. cerevisiae)	-0.70857873	0.01177784
202697_at	11051	NUDT21	nudix (nucleoside diphosphate linked moiety X)-type motif 21	-0.90796741	0.01186003
202718_at	3485	IGFBP2	insulin-like growth factor binding protein 2, 36kDa	-2.01358686	0.01189027
238558_at	NA	NA	NA	1.35104693	0.01197371
201377_at	9898	UBAP2L	ubiquitin associated protein 2-like	-0.72819135	0.01203314
201829_at	10276	NET1	neuroepithelial cell transforming 1	-0.99952753	0.01213762
212689_s_at	55818	KDM3A	lysine (K)-specific demethylase 3A	-0.92563474	0.01215449
212243_at	1488 | 145781	POLR2M | GCOM1	polymerase (RNA) II (DNA directed) polypeptide M | GRINL1A complex locus 1	-0.74048417	0.01217057
222761_at	54841	BIVM	basic, immunoglobulin-like variable motif containing	-1.47241078	0.01217215
201369_s_at	678	ZFP36L2	ZFP36 ring finger protein-like 2	-1.39717852	0.01230554
228278_at	4784	NFIX	nuclear factor I/X (CCAAT-binding transcription factor)	-1.01793361	0.01238497
221482_s_at	10776	ARPP19	cAMP-regulated phosphoprotein, 19kDa	-0.91388629	0.01247067
212187_x_at	5730	PTGDS	prostaglandin D2 synthase 21kDa (brain)	-0.83867124	0.0124713
223396_at	85025	TMEM60	transmembrane protein 60	-0.95610408	0.01251904
1554660_a_at	163882	CNST	consortin, connexin sorting protein	-1.31916705	0.0125389
206875_s_at	9748	SLK	STE20-like kinase	-0.84386171	0.01260881
209099_x_at	182	JAG1	jagged 1	-1.90027276	0.012611
236168_at	NA	NA	NA	1.32288214	0.01262474
227018_at	54878	DPP8	dipeptidyl-peptidase 8	-0.79960195	0.01276605
202918_s_at	3 | 100529241	MOB4 | HSPE1-MOB4	MOB family member 4, phocein | HSPE1-MOB4 readthrough	-0.93729856	0.01278816
226541_at	84085	FBXO30	F-box protein 30	-1.41903512	0.01283206
228495_at	253635	GPATCH11	G patch domain containing 11	-0.85085824	0.01295891
217980_s_at	54948	MRPL16	mitochondrial ribosomal protein L16	-0.66729221	0.01296376
200983_x_at	966	CD59	CD59 molecule, complement regulatory protein	-2.16028544	0.01304041
224643_at	133619	PRRC1	proline-rich coiled-coil 1	-0.92726429	0.01313164
226472_at	85313	PPIL4	peptidylprolyl isomerase (cyclophilin)-like 4	-0.94494409	0.01317317
203302_at	1633	DCK	deoxycytidine kinase	-1.1324722	0.0133251
202536_at	25978	CHMP2B	charged multivesicular body protein 2B	-1.01275718	0.01336135
244548_at	NA	NA	NA	1.25891685	0.01337412
211746_x_at	5682	PSMA1	proteasome (prosome, macropain) subunit, alpha type, 1	-0.58763877	0.01342178
218846_at	9439	MED23	mediator complex subunit 23	-0.74245384	0.01346779
212244_at	1488 | 145781	POLR2M | GCOM1	polymerase (RNA) II (DNA directed) polypeptide M | GRINL1A complex locus 1	-0.70222928	0.01349101
224878_at	56061	UBFD1	ubiquitin family domain containing 1	-0.71507414	0.01350832
209410_s_at	2887	GRB10	growth factor receptor-bound protein 10	-1.2188929	0.01361744
210137_s_at	1635	DCTD	dCMP deaminase	-0.58047725	0.01362403
41577_at	26051	PPP1R16B	protein phosphatase 1, regulatory subunit 16B	-1.55511628	0.01362724
203017_s_at	117178	SSX2IP	synovial sarcoma, X breakpoint 2 interacting protein	-1.41045645	0.01366985
203250_at	22828	SCAF8	SR-related CTD-associated factor 8	-0.77995337	0.0138266
242008_at	NA	NA	NA	1.3806045	0.01383073
227134_at	84958	SYTL1	synaptotagmin-like 1	-1.66755368	0.01389981
200614_at	1213	CLTC	clathrin, heavy chain (Hc)	-0.92202379	0.01390305
200056_s_at	10438	C1D	C1D nuclear receptor corepressor	-0.73799134	0.01396167
221229_s_at	55006	TRMT61B	tRNA methyltransferase 61B	-0.85887226	0.01397409
241985_at	133746	JMY	junction mediating and regulatory protein, p53 cofactor	-1.4304196	0.01398465
233271_at	NA	NA	NA	-1.1679658	0.01406113
202053_s_at	224	ALDH3A2	aldehyde dehydrogenase 3 family, member A2	1.00314808	0.01408243
223401_at	56985	ADPRM	ADP-ribose/CDP-alcohol diphosphatase, manganese-dependent	-0.83522257	0.01409539
209657_s_at	3298	HSF2	heat shock transcription factor 2	-1.1276397	0.01412118
204560_at	2289	FKBP5	FK506 binding protein 5	-1.57639951	0.01415543
203292_s_at	55823	VPS11	vacuolar protein sorting 11 homolog (S. cerevisiae)	-0.5857308	0.01415849
223077_at	29766	TMOD3	tropomodulin 3 (ubiquitous)	-1.02765836	0.01416552
217730_at	4 | 102465256	TMBIM1 | MIR6513	transmembrane BAX inhibitor motif containing 1 | microRNA 6513	-0.98121527	0.01421403
223370_at	65977	PLEKHA3	pleckstrin homology domain containing, family A (phosphoinositide binding specific) r	-0.79724302	0.01424141
205609_at	284	ANGPT1	angiopoietin 1	-2.81047807	0.01431938
38269_at	25865	PRKD2	protein kinase D2	-0.85372648	0.01439462
227455_at	221545	C6orf136	chromosome 6 open reading frame 136	-0.85890276	0.01445792
223216_x_at	5893 | 157574	ZNF395 | FBXO16	zinc finger protein 395 | F-box protein 16	-1.14757597	0.01447204
214700_x_at	55183	RIF1	replication timing regulatory factor 1	-0.71579068	0.01451224
228793_at	221037	JMJD1C	jumonji domain containing 1C	1.27640835	0.01458327
224076_s_at	54904	WHSC1L1	Wolf-Hirschhorn syndrome candidate 1-like 1	-0.78481371	0.01459333
1554679_a_at	55353	LAPTM4B	lysosomal protein transmembrane 4 beta	-1.5033226	0.0146035
233936_s_at	79893	GGNBP2	gametogenetin binding protein 2	-1.06878455	0.01465561
223140_s_at	170506	DHX36	DEAH (Asp-Glu-Ala-His) box polypeptide 36	-0.74282545	0.01467824
203414_at	23531	MMD	monocyte to macrophage differentiation-associated	-1.03240351	0.01469003
1553693_s_at	84869	CBR4	carbonyl reductase 4	0.77827338	0.01469407
212928_at	23270	TSPYL4	TSPY-like 4	-1.04763166	0.01475853
1561079_at	23243	ANKRD28	ankyrin repeat domain 28	1.16514821	0.01483759
208654_s_at	8763	CD164	CD164 molecule, sialomucin	-1.20043094	0.01494656
203406_at	4236	MFAP1	microfibrillar-associated protein 1	-0.9738072	0.01499943
212756_s_at	23304	UBR2	ubiquitin protein ligase E3 component n-recognin 2	-0.67167528	0.01500363
241788_x_at	NA	NA	NA	1.23704339	0.01501593
226199_at	139596	UPRT	uracil phosphoribosyltransferase (FUR1) homolog (S. cerevisiae)	-0.73123132	0.01502059
223519_at	51776	ZAK	sterile alpha motif and leucine zipper containing kinase AZK	-1.76570144	0.01508362
209409_at	2887	GRB10	growth factor receptor-bound protein 10	-1.87182302	0.01518375
204779_s_at	3217	HOXB7	homeobox B7	-1.84585581	0.01529781
220668_s_at	1789	DNMT3B	DNA (cytosine-5-)-methyltransferase 3 beta	-0.890631	0.01530403
213891_s_at	6925	TCF4	transcription factor 4	-2.01530948	0.01542177
201783_s_at	5970	RELA	v-rel avian reticuloendotheliosis viral oncogene homolog A	-0.75660456	0.0154481
203725_at	1647	GADD45A	growth arrest and DNA-damage-inducible, alpha	-1.1956976	0.01548017
218520_at	29110	TBK1	TANK-binding kinase 1	-0.63796655	0.01550509
200927_s_at	51552	RAB14	RAB14, member RAS oncogene family	-1.27948444	0.0155141
201143_s_at	1965	EIF2S1	eukaryotic translation initiation factor 2, subunit 1 alpha, 35kDa	-0.88680196	0.01553142
220603_s_at	55784	MCTP2	multiple C2 domains, transmembrane 2	-1.40325063	0.01556432
1556059_s_at	23013	SPEN	spen family transcriptional repressor	-0.71370204	0.01559341
232291_at	5982 | 407048	MIR17HG | MIR17 | M	miR-17-92 cluster host gene | microRNA 17 | microRNA 18a | microRNA 19a | microR	1.7074042	0.01561605
242931_at	NA	NA	NA	1.74840123	0.01561616
219220_x_at	56945	MRPS22	mitochondrial ribosomal protein S22	-0.54746469	0.0156345
200923_at	3959	LGALS3BP	lectin, galactoside-binding, soluble, 3 binding protein	-2.21602353	0.01566926
1557797_a_at	NA	NA	NA	1.19479824	0.01573629

208671_at	57515	SERINC1	serine incorporator 1	-0.93325977	0.0157627
203447_at	5711	PSMD5	proteasome (prosome, macropain) 26S subunit, non-ATPase, 5	-0.83355865	0.01577624
209311_at	599	BCL2L2	BCL2-like 2	-0.69608941	0.01583844
210104_at	10001	MED6	mediator complex subunit 6	-0.69217781	0.01583915
216950_s_at	) | 100132417	FCGR1A | FCGR1B | F(	Fc fragment of IgG, high affinity Ia, receptor (CD64) | Fc fragment of IgG, high affinit	1.59729419	0.0159118
230787_at	101927886	LOC101927886	uncharacterized LOC101927886	1.05816291	0.01595335
205366_s_at	3216	HOXB6	homeobox B6	-2.70119259	0.01597609
202773_s_at	6433	SFSWAP	splicing factor, suppressor of white-apricot family	-0.64682267	0.01600721
219176_at	79568	C2orf47	chromosome 2 open reading frame 47	-0.56258532	0.01602122
238079_at	7170	TPM3	tropomyosin 3	1.10078851	0.01603707
220606_s_at	56985	ADPRM	ADP-ribose/CDP-alcohol diphosphatase, manganese-dependent	-1.13035613	0.01613657
205997_at	10863	ADAM28	ADAM metallopeptidase domain 28	-2.09481454	0.01614559
202763_at	836	CASP3	caspase 3, apoptosis-related cysteine peptidase	-0.98081312	0.01633271
218593_at	55131	RBM28	RNA binding motif protein 28	-0.86385847	0.0163393
230097_at	2618	GART	phosphoribosylglycinamide formyltransferase, phosphoribosylglycinamide synthetase,	0.90531123	0.01642129
223383_at	84937	ZNRF1	zinc and ring finger 1, E3 ubiquitin protein ligase	-0.96731969	0.01642702
230264_s_at	8905	AP1S2	adaptor-related protein complex 1, sigma 2 subunit	1.00177958	0.01645379
208831_x_at	6830	SUPT6H	suppressor of Ty 6 homolog (S. cerevisiae)	-0.86598062	0.01651117
211729_x_at	644	BLVRA	biliverdin reductase A	1.05107345	0.01668794
207845_s_at	10393	ANAPC10	anaphase promoting complex subunit 10	-1.05444476	0.0167084
216766_at	NA	NA	NA	1.71493697	0.01675993
224856_at	2289	FKBP5	FK506 binding protein 5	-1.60927065	0.01680998
220134_x_at	55194	EVA1B	eva-1 homolog B (C. elegans)	-0.76386631	0.01685056
208091_s_at	81552	VOPP1	vesicular, overexpressed in cancer, prosurvival protein 1	-0.94554216	0.01686717
236738_at	401097	C3orf80	chromosome 3 open reading frame 80	-2.32463047	0.01689047
1556658_a_at	NA	NA	NA	1.52787661	0.01689702
212168_at	10137	RBM12	RNA binding motif protein 12	-0.97172616	0.01689838
217822_at	51729	WBP11	WW domain binding protein 11	-0.54184134	0.01700626
205042_at	10020	GNE	glucosamine (UDP-N-acetyl)-2-epimerase/N-acetylmannosamine kinase	-0.54009293	0.01702602
203403_s_at	6049	RNF6	ring finger protein (C3H2C3 type) 6	-0.66454784	0.01703289
226732_at	155435	RBM33	RNA binding motif protein 33	-0.83935497	0.01704832
240347_at	NA	NA	NA	0.95945028	0.0170531
232478_at	100379345	MIR181A2HG	MIR181A2 host gene	1.04142695	0.01705435
203432_at	7112	TMPO	thymopoietin	-1.01894961	0.01712149
213225_at	5495	PPM1B	protein phosphatase, Mg2+/Mn2+ dependent, 1B	-0.67120968	0.01720318
210907_s_at	11235	PDCD10	programmed cell death 10	-0.7747755	0.01725756
225550_at	163882	CNST	consortin, connexin sorting protein	-1.24489838	0.0173672
208653_s_at	8763	CD164	CD164 molecule, sialomucin	-1.00636844	0.01738841
227948_at	121512	FGD4	FYVE, RhoGEF and PH domain containing 4	1.21911736	0.01739532
235490_at	84314	TMEM107	transmembrane protein 107	1.91550427	0.01740466
203562_at	9638	FEZ1	fasciculation and elongation protein zeta 1 (zygin I)	1.62923472	0.01747903
204614_at	5055	SERPINB2	serpin peptidase inhibitor, clade B (ovalbumin), member 2	2.75244937	0.01752559
214771_x_at	23164	MPRIP	myosin phosphatase Rho interacting protein	-0.70575501	0.01755539
209592_s_at	10238	DCAF7	DDB1 and CUL4 associated factor 7	-0.71305158	0.01756362
212749_s_at	25898	RCHY1	ring finger and CHY zinc finger domain containing 1, E3 ubiquitin protein ligase	-1.14819129	0.01758853
238127_at	650669	GAS6-AS1	GAS6 antisense RNA 1	1.41168457	0.01759084
208789_at	284119	PTRF	polymerase I and transcript release factor	-0.83096087	0.01764454
212060_at	23350	U2SURP	U2 snRNP-associated SURP domain containing	-0.88870399	0.01765943
217883_at	27249	MMADHC	methylmalonic aciduria (cobalamin deficiency) cblD type, with homocystinuria	-0.59168279	0.01766738
211954_s_at	3843	IPO5	importin 5	-0.69119031	0.01770026
218514_at	55181	SMG8	SMG8 nonsense mediated mRNA decay factor	-0.84342749	0.01770633
240165_at	NA	NA	NA	1.14629744	0.01772011
203015_s_at	117178	SSX2IP	synovial sarcoma, X breakpoint 2 interacting protein	-1.40202022	0.01774313
216621_at	NA	NA	NA	0.94039978	0.01775901
223000_s_at	50848	F11R	F11 receptor	-1.00030582	0.01777071
218478_s_at	55596	ZCCHC8	zinc finger, CCHC domain containing 8	-0.70667473	0.0178193
209330_s_at	3184	HNRNPD	heterogeneous nuclear ribonucleoprotein D (AU-rich element RNA binding protein 1,	-0.51145746	0.01782289
205899_at	8900	CCNA1	cyclin A1	-2.91843028	0.01782924
201161_s_at	8531	YBX3	Y box binding protein 3	-1.00201418	0.01789204
222624_s_at	51193	ZNF639	zinc finger protein 639	-0.90542527	0.01800049
202171_at	7716	VEZF1	vascular endothelial zinc finger 1	-0.83086404	0.0180687
203985_at	7988	ZNF212	zinc finger protein 212	-0.56986004	0.01807398
239647_at	166012	CHST13	carbohydrate (chondroitin 4) sulfotransferase 13	1.24092464	0.01812268
221536_s_at	55341	LSG1	large 60S subunit nuclear export GTPase 1	-0.79063745	0.01815452
209031_at	23705	CADM1	cell adhesion molecule 1	1.71066203	0.01823163
209374_s_at	3507	IGHM	immunoglobulin heavy constant mu	-1.76498894	0.01824626
201799_s_at	5007	OSBP	oxysterol binding protein	-0.72507519	0.01831916
228059_x_at	56945	MRPS22	mitochondrial ribosomal protein S22	-0.46439934	0.01837551
228910_at	3732	CD82	CD82 molecule	-0.75670991	0.01841741
204440_at	9308	CD83	CD83 molecule	1.38585113	0.01847166
203341_at	10153	CEBPZ	CCAAT/enhancer binding protein (C/EBP), zeta	-0.63539948	0.01848655
219218_at	57597	BAHCC1	BAH domain and coiled-coil containing 1	-1.12129359	0.01856608
212676_at	4763	NF1	neurofibromin 1	-0.90678741	0.01863208
209813_x_at	5967 | 445347	TRGV9 | TRGC2 | TARI	T cell receptor gamma variable 9 | T cell receptor gamma constant 2 | TCR gamma a	-2.19569138	0.01870438
212055_at	25941	TPGS2	tubulin polyglutamylase complex subunit 2	-1.26515534	0.01870545
212286_at	23253	ANKRD12	ankyrin repeat domain 12	-0.88588177	0.01873996
201068_s_at	5701	PSMC2	proteasome (prosome, macropain) 26S subunit, ATPase, 2	-0.7425243	0.01881536
218028_at	4 | 102466723	ELOVL1 | MIR6734	ELOVL fatty acid elongase 1 | microRNA 6734	-0.82339838	0.01882997
203965_at	10868	USP20	ubiquitin specific peptidase 20	-0.97116147	0.01889145
243395_at	NA	NA	NA	1.34468615	0.01890102
212597_s_at	10042	HMGXB4	HMG box domain containing 4	-0.79036035	0.01895078
221190_s_at	29919	C18orf8	chromosome 18 open reading frame 8	-0.82727127	0.01899377
225827_at	27161	AGO2	argonaute RISC catalytic component 2	-0.87611327	0.01906387
221725_at	10163	WASF2	WAS protein family, member 2	-0.8231581	0.01908598
212584_at	9716	AQR	aquarius intron-binding spliceosomal factor	-0.67466692	0.01909524
211318_s_at	8480	RAE1	ribonucleic acid export 1	-0.64892223	0.01910395
225406_at	57045	TWSG1	twisted gastrulation BMP signaling modulator 1	-1.24833136	0.01918379
209717_at	7813	EVI5	ecotropic viral integration site 5	0.96167063	0.01920647
209382_at	10623	POLR3C	polymerase (RNA) III (DNA directed) polypeptide C (62kD)	-0.79161444	0.01924844
209647_s_at	9655	SOCS5	suppressor of cytokine signaling 5	-1.14213928	0.01926806
211144_x_at	| 6967 | 6983	TARP | TRGC2 | TRGV	TCR gamma alternate reading frame protein | T cell receptor gamma constant 2 | T c	-2.10411886	0.01934595
212301_at	23168	RTF1	Rtf1, Paf1/RNA polymerase II complex component, homolog (S. cerevisiae)	-0.61215517	0.01935811
205361_s_at	5203	PFDN4	prefoldin subunit 4	-0.66646079	0.01939866
230424_at	9315	NREP	neuronal regeneration related protein	1.46575	0.01941598
203087_s_at	3796	KIF2A	kinesin heavy chain member 2A	-0.96737321	0.01941933
201163_s_at	3490	IGFBP7	insulin-like growth factor binding protein 7	1.49474857	0.01944604
217975_at	51186	WBP5	WW domain binding protein 5	-1.96145768	0.01946838
203519_s_at	26019	UPF2	UPF2 regulator of nonsense transcripts homolog (yeast)	-0.92389776	0.01949696
215160_x_at	5 | 100289097	LOC642236 | LOC100	FSHD region gene 1 pseudogene | protein FRG1-like	-0.70399919	0.01954974
1554543_at	9043	SPAG9	sperm associated antigen 9	1.40940718	0.01956749
228152_s_at	91351	DDX60L	DEAD (Asp-Glu-Ala-Asp) box polypeptide 60-like	1.16633434	0.01957561
236251_at	NA	NA	NA	-1.51037042	0.01961455
202043_s_at	6611	SMS	spermine synthase	-0.68745569	0.01963466
219869_s_at	64116	SLC39A8	solute carrier family 39 (zinc transporter), member 8	-1.08771669	0.01964453

202042_at	3035	HARS	histidyl-tRNA synthetase	-0.70102177	0.01976076
200747_s_at	4926	NUMA1	nuclear mitotic apparatus protein 1	-0.64218686	0.01981797
233955_x_at	51523	CXXC5	CXXC finger protein 5	-1.25956973	0.01991533
236338_at	NA	NA	NA	1.31619355	0.01999833
209170_s_at	2824	GPM6B	glycoprotein M6B	2.9138018	0.02002532
202760_s_at	45815 | 11217	PALM2-AKAP2 | AKAP2	PALM2-AKAP2 readthrough | A kinase (PRKA) anchor protein 2	2.16543635	0.02004656
227394_at	4684	NCAM1	neural cell adhesion molecule 1	1.53109348	0.02005159
212648_at	54505	DHX29	DEAH (Asp-Glu-Ala-His) box polypeptide 29	-0.90681228	0.02016427
228106_at	54876	DCAF16	DDB1 and CUL4 associated factor 16	-0.83791882	0.02017967
210949_s_at	3663 | 728689	EIF3C | EIF3CL	eukaryotic translation initiation factor 3, subunit C | eukaryotic translation initiation fa	-0.52358111	0.02023674
202020_s_at	10314	LANCL1	LanC lantibiotic synthetase component C-like 1 (bacterial)	-0.90324527	0.02026402
212458_at	200734	SPRED2	sprouty-related, EVH1 domain containing 2	-1.59126629	0.02040554
203203_s_at	11103	KRR1	KRR1, small subunit (SSU) processome component, homolog (yeast)	-0.81800786	0.02043415
212387_at	6925	TCF4	transcription factor 4	-1.79886938	0.02043785
202337_at	11243	PMF1	polyamine-modulated factor 1	-0.76567872	0.02046087
213118_at	23074	UHRF1BP1L	UHRF1 binding protein 1-like	-0.83536499	0.02048233
202054_s_at	224	ALDH3A2	aldehyde dehydrogenase 3 family, member A2	0.9573969	0.02048634
223947_s_at	9439	MED23	mediator complex subunit 23	-0.64887869	0.02062182
215806_x_at	6983 | 445347	TRGC2 | TRGV9 | TARI	T cell receptor gamma constant 2 | T cell receptor gamma variable 9 | TCR gamma a	-2.16844069	0.02067144
223211_at	26061	HACL1	2-hydroxyacyl-CoA lyase 1	-0.67735562	0.02068225
206478_at	9834	KIAA0125	KIAA0125	-2.92483777	0.02109904
220377_at	9834	KIAA0125	KIAA0125	-1.90001108	0.02122547
211137_s_at	27032	ATP2C1	ATPase, Ca++ transporting, type 2C, member 1	-0.96307351	0.02128172
201663_s_at	10051	SMC4	structural maintenance of chromosomes 4	-1.20411297	0.02134607
225974_at	169200	TMEM64	transmembrane protein 64	-1.90448992	0.02136532
204725_s_at	4690	NCK1	NCK adaptor protein 1	-0.91787952	0.02141379
226264_at	64420	SUSD1	sushi domain containing 1	-0.77682093	0.02144378
203044_at	22856	CHSY1	chondroitin sulfate synthase 1	0.91454914	0.02144713
226128_at	148362	BROX	BRO1 domain and CAAX motif containing	-0.84430619	0.0214666
212568_s_at	1737	DLAT	dihydrolipoamide S-acetyltransferase	-0.98427557	0.02149438
1561690_at	NA	NA	NA	-1.46812209	0.0214981
1554948_at	NA	NA	NA	1.55954803	0.02150544
1554365_a_at	5527	PPP2R5C	protein phosphatase 2, regulatory subunit B', gamma	-0.78355325	0.02153169
223192_at	81894	SLC25A28	solute carrier family 25 (mitochondrial iron transporter), member 28	-0.60735303	0.02153436
218528_s_at	152006	RNF38	ring finger protein 38	-0.69544575	0.02158252
204044_at	23475	QPRT	quinolinate phosphoribosyltransferase	-1.02412974	0.02162186
233223_at	NA	NA	NA	1.17573321	0.02167651
203818_s_at	10946	SF3A3	splicing factor 3a, subunit 3, 60kDa	-0.76717303	0.02174225
208735_s_at	10106	CTDSP2	CTD (carboxy-terminal domain, RNA polymerase II, polypeptide A) small phosphatase	-0.98357702	0.02175827
207629_s_at	9181	ARHGEF2	Rho/Rac guanine nucleotide exchange factor (GEF) 2	-0.81715967	0.02179651
1554661_s_at	163882	CNST	consortin, connexin sorting protein	-1.04567928	0.02183108
229510_at	84689	MS4A14	membrane-spanning 4-domains, subfamily A, member 14	1.60628936	0.0218937
209486_at	57050	UTP3	UTP3, small subunit (SSU) processome component, homolog (S. cerevisiae)	-0.86334282	0.02192015
202693_s_at	9263	STK17A	serine/threonine kinase 17a	-1.14649523	0.02192416
224516_s_at	51523	CXXC5	CXXC finger protein 5	-1.23143164	0.02196573
223513_at	55835	CENPJ	centromere protein J	-1.04829875	0.02197649
203773_x_at	644	BLVRA	biliverdin reductase A	1.00110276	0.02200958
1556203_a_at	23380	SRGAP2	SLIT-ROBO Rho GTPase activating protein 2	1.20749605	0.02201934
203437_at	8834	TMEM11	transmembrane protein 11	-0.61997925	0.02209543
218807_at	10451	VAV3	vav 3 guanine nucleotide exchange factor	-1.39356674	0.02216723
236840_at	115749	C12orf56	chromosome 12 open reading frame 56	1.47660331	0.0222306
224636_at	1270 | 80829	CNTF | ZFP91	ciliary neurotrophic factor | ZFP91 zinc finger protein	-0.77740422	0.02225604
208713_at	11100	HNRNPUL1	heterogeneous nuclear ribonucleoprotein U-like 1	-0.50427027	0.02238728
221970_s_at	25926	NOL11	nucleolar protein 11	-0.79917171	0.02241685
225133_at	51274	KLF3	Kruppel-like factor 3 (basic)	-1.7684018	0.02250947
200815_s_at	5048	PAFAH1B1	platelet-activating factor acetylhydrolase 1b, regulatory subunit 1 (45kDa)	-0.65979365	0.02251737
235407_at	27342	RABGEF1	RAB guanine nucleotide exchange factor (GEF) 1	1.11942174	0.02251752
212388_at	23358	USP24	ubiquitin specific peptidase 24	-0.88094081	0.02286697
222602_at	55236	UBA6	ubiquitin-like modifier activating enzyme 6	-0.6004935	0.02307429
218911_at	8089	YEATS4	YEATS domain containing 4	-0.85597628	0.02317621
222533_at	51185	CRBN	cereblon	-0.87284261	0.02318377
235181_at	129450	TYW5	tRNA-yW synthesizing protein 5	-0.80339935	0.02318601
214430_at	2717	GLA	galactosidase, alpha	0.75152095	0.02322266
211734_s_at	2205	FCER1A	Fc fragment of IgE, high affinity I, receptor for; alpha polypeptide	-2.29356607	0.02330804
202658_at	8799	PEX11B	peroxisomal biogenesis factor 11 beta	-1.20386664	0.023312
205928_at	10224	ZNF443	zinc finger protein 443	-0.97454953	0.02334919
226091_s_at	93621	MRFAP1	Morf4 family associated protein 1	-0.48894061	0.0234995
212636_at	9444	QKI	QKI, KH domain containing, RNA binding	1.20271036	0.02351486
210549_s_at	6368	CCL23	chemokine (C-C motif) ligand 23	2.07699792	0.02353053
210759_s_at	5682	PSMA1	proteasome (prosome, macropain) subunit, alpha type, 1	-0.64499563	0.02354164
222920_s_at	9840	TESPA1	thymocyte expressed, positive selection associated 1	-1.27654538	0.0236105
215285_s_at	10745	PHTF1	putative homeodomain transcription factor 1	-0.88395143	0.02375532
209022_at	10735	STAG2	stromal antigen 2	-0.67713981	0.02376806
227447_at	23517	SKIV2L2	superkiller viralicidic activity 2-like 2 (S. cerevisiae)	-0.68257239	0.02380562
232798_at	85479	DNAJC5B	DnaJ (Hsp40) homolog, subfamily C, member 5 beta	0.93545617	0.0238415
200780_x_at	2778	GNAS	GNAS complex locus	-0.50221715	0.0239132
202654_x_at	64844	MARCH7	membrane-associated ring finger (C3HC4) 7, E3 ubiquitin protein ligase	-1.271388	0.02392136
221732_at	124583	CANT1	calcium activated nucleotidase 1	-0.63445977	0.02392295
227185_at	643988	C1orf233	chromosome 1 open reading frame 233	0.76342264	0.0239254
216920_s_at	| 6967 | 6983	TARP | TRGC2 | TRGV	TCR gamma alternate reading frame protein | T cell receptor gamma constant 2 | T c	-2.03377854	0.02393578
206342_x_at	3423	IDS	iduronate 2-sulfatase	-1.15488319	0.02399496
217978_s_at	55585	UBE2Q1	ubiquitin-conjugating enzyme E2Q family member 1	-0.55740477	0.02401221
232797_at	NA	NA	NA	-1.64012003	0.02403782
200833_s_at	5908	RAP1B	RAP1B, member of RAS oncogene family	-0.70540717	0.02413745
201687_s_at	8539	API5	apoptosis inhibitor 5	-1.05498227	0.02413879
215392_at	NA	NA	NA	1.24191559	0.02419595
226134_s_at	124540	MSI2	musashi RNA-binding protein 2	-1.49906762	0.02422325
225887_at	80209	PROSER1	proline and serine rich 1	-1.35863834	0.02431843
222417_s_at	27131	SNX5	sorting nexin 5	-0.55649175	0.02437566
222146_s_at	6925	TCF4	transcription factor 4	-1.93634963	0.02438988
201845_s_at	23429	RYBP	RING1 and YY1 binding protein	-0.9805548	0.02441215
215017_s_at	54874	FNBP1L	formin binding protein 1-like	-1.53579532	0.02447204
218562_s_at	55219	TMEM57	transmembrane protein 57	-0.63330935	0.02450185
219141_s_at	55626	AMBRA1	autophagy/beclin-1 regulator 1	-0.55838746	0.02450265
201176_s_at	372	ARCN1	archain 1	-1.02026776	0.02452067
233642_s_at	54497	HEATR5B	HEAT repeat containing 5B	-1.12641648	0.02454988
209825_s_at	l | 100500832	UCK2 | MIR3658	uridine-cytidine kinase 2 | microRNA 3658	-0.49579462	0.02461077
208619_at	1642	DDB1	damage-specific DNA binding protein 1, 127kDa	-0.66210748	0.02473176
202346_at	3093	UBE2K	ubiquitin-conjugating enzyme E2K	-0.82197693	0.02474058
223110_at	25962	KIAA1429	KIAA1429	-0.6725607	0.02474684
202495_at	6903	TBCC	tubulin folding cofactor C	-0.5944117	0.02477237
217963_s_at	27018	NGFRAP1	nerve growth factor receptor (TNFRSF16) associated protein 1	-2.05729271	0.02482442
212546_s_at	285527	FRYL	FRY-like	-0.95264329	0.02486653
200981_x_at	2778	GNAS	GNAS complex locus	-0.49561365	0.02497603

201934_at	80335	WDR82	WD repeat domain 82	-0.55571078	0.02506196
225114_at	8540	AGPS	alkylglycerone phosphate synthase	-1.1067822	0.02514805
1559739_at	56994	CHPT1	choline phosphotransferase 1	-1.18642002	0.02523564
203490_at	2000	ELF4	E74-like factor 4 (ets domain transcription factor)	-0.80328033	0.02526337
218014_at	79902	NUP85	nucleoporin 85kDa	-0.75657458	0.02532658
202116_at	5977	DPF2	D4, zinc and double PHD fingers family 2	-0.74621446	0.02547124
225461_at	79813	EHMT1	euchromatic histone-lysine N-methyltransferase 1	-0.90400479	0.0254831
202653_s_at	64844	MARCH7	membrane-associated ring finger (C3HC4) 7, E3 ubiquitin protein ligase	-0.82899451	0.02559609
218079_s_at	79893	GGNBP2	gametogenetin binding protein 2	-0.81612295	0.02571064
201364_s_at	4947	OAZ2	ornithine decarboxylase antizyme 2	-0.76995232	0.02571308
218398_at	10884	MRPS30	mitochondrial ribosomal protein S30	-0.59547997	0.02582134
203097_s_at	9693	RAPGEF2	Rap guanine nucleotide exchange factor (GEF) 2	-1.08704706	0.0259368
218732_at	51651	PTRH2	peptidyl-tRNA hydrolase 2	-0.70530856	0.02595956
210548_at	6368	CCL23	chemokine (C-C motif) ligand 23	1.98604247	0.02598381
217928_s_at	55291	PPP6R3	protein phosphatase 6, regulatory subunit 3	-0.5552241	0.02606118
217942_at	60488	MRPS35	mitochondrial ribosomal protein S35	-0.47193612	0.02608307
222531_s_at	55745	AP5M1	adaptor-related protein complex 5, mu 1 subunit	-0.71318755	0.0261848
206015_s_at	22887	FOXJ3	forkhead box J3	-0.6294298	0.02622949
225267_at	3840	KPNA4	karyopherin alpha 4 (importin alpha 3)	-0.57515988	0.02630601
208855_s_at	8428	STK24	serine/threonine kinase 24	-0.90427385	0.02632176
212893_at	26009	ZZZ3	zinc finger, ZZ-type containing 3	-0.81971154	0.0263409
213037_x_at	6780	STAU1	staufen double-stranded RNA binding protein 1	-0.67907929	0.02641455
224358_s_at	58475	MS4A7	membrane-spanning 4-domains, subfamily A, member 7	1.47437124	0.02641493
209091_s_at	51100	SH3GLB1	SH3-domain GRB2-like endophilin B1	-0.60196052	0.02646957
205214_at	9262	STK17B	serine/threonine kinase 17b	-1.40476646	0.02648256
211988_at	6605	SMARCE1	SWI/SNF related, matrix associated, actin dependent regulator of chromatin, subfami	-0.62177012	0.02649348
220532_s_at	28959	TMEM176B	transmembrane protein 176B	1.93488542	0.02661004
200023_s_at	8665	EIF3F	eukaryotic translation initiation factor 3, subunit F	-0.48337613	0.02668386
223343_at	58475	MS4A7	membrane-spanning 4-domains, subfamily A, member 7	1.59615209	0.02670629
201727_s_at	1994	ELAVL1	ELAV like RNA binding protein 1	-0.59846012	0.026735
215773_x_at	10038	PARP2	poly (ADP-ribose) polymerase 2	-0.65964266	0.02679012
218896_s_at	55421	C17orf85	chromosome 17 open reading frame 85	0.53844992	0.02680744
211953_s_at	3843	IPO5	importin 5	-0.96232745	0.0268214
202770_s_at	901	CCNG2	cyclin G2	-0.79258066	0.02686854
235652_at	NA	NA	NA	1.38578472	0.02693339
236621_at	6232	RPS27	ribosomal protein S27	1.50652364	0.02697086
201955_at	892	CCNC	cyclin C	-0.65364424	0.02697955
218626_at	56478	EIF4ENIF1	eukaryotic translation initiation factor 4E nuclear import factor 1	-0.8608515	0.02703605
202241_at	10221	TRIB1	tribbles pseudokinase 1	1.53432921	0.02707394
200673_at	9741	LAPTM4A	lysosomal protein transmembrane 4 alpha	-0.524396	0.02710607
200986_at	710	SERPING1	serpin peptidase inhibitor, clade G (C1 inhibitor), member 1	-1.51604093	0.02714341
223215_s_at	51528	JKAMP	JNK1/MAPK8-associated membrane protein	-0.79331178	0.02714716
201356_at	10291	SF3A1	splicing factor 3a, subunit 1, 120kDa	-0.56098695	0.0271477
201719_s_at	2037	EPB41L2	erythrocyte membrane protein band 4.1-like 2	-1.59287905	0.0271797
218823_s_at	54793	KCTD9	potassium channel tetramerization domain containing 9	-0.86866452	0.02720475
204799_at	9889	ZBED4	zinc finger, BED-type containing 4	-1.01796774	0.02730399
217742_s_at	51322	WAC	WW domain containing adaptor with coiled-coil	-0.74437869	0.02732077
202774_s_at	6433	SFSWAP	splicing factor, suppressor of white-apricot family	-0.67555405	0.02740447
219161_s_at	2 | 100529251	CKLF | CKLF-CMTM1	chemokine-like factor | CKLF-CMTM1 readthrough	-0.72792274	0.02746468
212380_at	23070	CMTR1	cap methyltransferase 1	-0.50386255	0.02749012
211423_s_at	6309	SC5D	sterol-C5-desaturase	-1.01765982	0.0275239
206238_s_at	10138	YAF2	YY1 associated factor 2	-1.04578591	0.0276267
201493_s_at	23369	PUM2	pumilio RNA-binding family member 2	-0.71770395	0.0276693
209056_s_at	988	CDC5L	cell division cycle 5-like	-0.67062903	0.02769067
230078_at	51735	RAPGEF6	Rap guanine nucleotide exchange factor (GEF) 6	-0.89414965	0.02769307
202531_at	3659	IRF1	interferon regulatory factor 1	-0.80239552	0.02775514
208915_s_at	23062	GGA2	golgi-associated, gamma adaptin ear containing, ARF binding protein 2	0.65367256	0.02776256
227787_s_at	90390	MED30	mediator complex subunit 30	-0.97028695	0.02776592
226861_at	140461	ASB8	ankyrin repeat and SOCS box containing 8	-0.62154529	0.02777055
212264_s_at	23063	WAPAL	wings apart-like homolog (Drosophila)	-0.61585298	0.02786096
225578_at	440145	MZT1	mitotic spindle organizing protein 1	-0.87703054	0.0278793
210658_s_at	23062	GGA2	golgi-associated, gamma adaptin ear containing, ARF binding protein 2	0.80798827	0.0278868
218991_at	63897	HEATR6	HEAT repeat containing 6	-0.64428625	0.02788847
222497_x_at	51068	NMD3	NMD3 ribosome export adaptor	-0.45151341	0.02797882
201476_s_at	6240	RRM1	ribonucleotide reductase M1	-1.12333463	0.0280569
220937_s_at	27090	ST6GALNAC4	ST6 (alpha-N-acetyl-neuraminyl-2,3-beta-galactosyl-1,3)-N-acetylgalactosaminide al	-0.89138184	0.02806957
204834_at	10875	FGL2	fibrinogen-like 2	1.90391223	0.02808297
209435_s_at	9181	ARHGEF2	Rho/Rac guanine nucleotide exchange factor (GEF) 2	-0.62143604	0.02820722
230285_at	258010	SVIP	small VCP/p97-interacting protein	-1.38612607	0.02824888
207095_at	6555	SLC10A2	solute carrier family 10 (sodium/bile acid cotransporter), member 2	1.65740641	0.02829439
227945_at	23216	TBC1D1	TBC1 (tre-2/USP6, BUB2, cdc16) domain family, member 1	1.08454778	0.0282977
204381_at	4037	LRP3	low density lipoprotein receptor-related protein 3	0.74963368	0.0283619
234984_at	121441	NEDD1	neural precursor cell expressed, developmentally down-regulated 1	-0.64303235	0.02836329
210676_x_at	7851 | 729540	RGPD5 | RGPD4 | RGF	RANBP2-like and GRIP domain containing 5 | RANBP2-like and GRIP domain containir	-1.09408786	0.02839557
205105_at	4124	MAN2A1	mannosidase, alpha, class 2A, member 1	-0.67924005	0.02842119
223526_at	83608	C18orf21	chromosome 18 open reading frame 21	-0.60596486	0.02843731
209878_s_at	5970	RELA	v-rel avian reticuloendotheliosis viral oncogene homolog A	-0.83881387	0.0284537
243051_at	29097	CNIH4	cornichon family AMPA receptor auxiliary protein 4	1.2296554	0.02860448
202140_s_at	1198	CLK3	CDC-like kinase 3	-0.64081706	0.02865218
208420_x_at	6830	SUPT6H	suppressor of Ty 6 homolog (S. cerevisiae)	-0.68955565	0.02876014
226391_at	673	BRAF	B-Raf proto-oncogene, serine/threonine kinase	-0.7422839	0.02883621
201532_at	5684	PSMA3	proteasome (prosome, macropain) subunit, alpha type, 3	-0.57046896	0.02885363
216899_s_at	8935	SKAP2	src kinase associated phosphoprotein 2	-1.49840355	0.0288541
200647_x_at	3663 | 728689	EIF3C | EIF3CL	eukaryotic translation initiation factor 3, subunit C | eukaryotic translation initiation fa	-0.45314645	0.02885657
203428_s_at	25842	ASF1A	anti-silencing function 1A histone chaperone	-1.17706085	0.02887804
239243_at	2 | 100507113	ZNF638 | ZNF638-IT1	zinc finger protein 638 | ZNF638 intronic transcript 1	-0.90046106	0.02890582
212114_at	552889	ATXN7L3B	ataxin 7-like 3B	-0.81336046	0.02890804
202382_s_at	10007	GNPDA1	glucosamine-6-phosphate deaminase 1	1.04108414	0.02896601
205061_s_at	5393	EXOSC9	exosome component 9	-0.69515502	0.02897664
226663_at	100505494	ANKRD10-IT1	ANKRD10 intronic transcript 1	1.27215122	0.02904248
207856_s_at	5627 | 150776	SMPD4 | LOC150776	sphingomyelin phosphodiesterase 4, neutral membrane (neutral sphingomyelinase-3)	-0.73419082	0.0291475
218405_at	29777	ABT1	activator of basal transcription 1	-0.58848027	0.02918678
218339_at	29093	MRPL22	mitochondrial ribosomal protein L22	-0.6049412	0.02924231
206695_x_at	7594	ZNF43	zinc finger protein 43	-0.81450671	0.02925013
201604_s_at	4659	PPP1R12A	protein phosphatase 1, regulatory subunit 12A	-0.67416951	0.0292797
211594_s_at	65005	MRPL9	mitochondrial ribosomal protein L9	-0.50011258	0.02932492
207196_s_at	10318	TNIP1	TNFAIP3 interacting protein 1	-0.6227039	0.02937368
222099_s_at	26065	LSM14A	LSM14A, SCD6 homolog A (S. cerevisiae)	-0.48937506	0.02942587
224473_x_at	84445	LZTS2	leucine zipper, putative tumor suppressor 2	-0.5901965	0.02945274
209055_s_at	988	CDC5L	cell division cycle 5-like	-0.85044049	0.02948929
205474_at	51379	CRLF3	cytokine receptor-like factor 3	-0.8643955	0.02959264
203261_at	10671	DCTN6	dynactin 6	-0.64991648	0.02960038
209654_at	23379	ICE1	interactor of little elongation complex ELL subunit 1	-0.70624507	0.02960355
226278_at	258010	SVIP	small VCP/p97-interacting protein	-1.30473998	0.02965388

1562307_at	NA	NA	NA	1.23651633	0.02970808
210208_x_at	7917	BAG6	BCL2-associated athanogene 6	-0.55706031	0.02973557
231896_s_at	8562	DENR	density-regulated protein	-0.61639475	0.0297798
227062_at	3131 | 693197	NEAT1 | MIR612	nuclear paraspeckle assembly transcript 1 (non-protein coding) | microRNA 612	1.4001485	0.02986795
225614_at	113174	SAAL1	serum amyloid A-like 1	-0.55611457	0.02992364
235142_at	653121	ZBTB8A	zinc finger and BTB domain containing 8A	-2.14840824	0.02996437
209861_s_at	10988	METAP2	methionyl aminopeptidase 2	-0.84074031	0.02999077
225297_at	115106	HAUS1	HAUS augmin-like complex, subunit 1	-0.88466649	0.0299967
226838_at	130502	TTC32	tetratricopeptide repeat domain 32	-0.80975943	0.03000127
208095_s_at	818 | 6731	CAMK2G | SRP72	calcium/calmodulin-dependent protein kinase II gamma | signal recognition particle 7	-0.63142243	0.03002592
204630_s_at	9527	GOSR1	golgi SNAP receptor complex member 1	-0.54550674	0.03003185
222646_s_at	30001	ERO1L	ERO1-like (S. cerevisiae)	-0.8699009	0.0300715
237176_at	NA	NA	NA	0.96786204	0.03008233
203612_at	705	BYSL	bystin-like	-0.62244937	0.03011565
220252_x_at	80231	CXorf21	chromosome X open reading frame 21	1.28227534	0.03013182
215460_x_at	23774	BRD1	bromodomain containing 1	-0.71618034	0.0302823
209799_at	5562	PRKAA1	protein kinase, AMP-activated, alpha 1 catalytic subunit	-1.0447086	0.03030102
210829_s_at	23635	SSBP2	single-stranded DNA binding protein 2	-1.35684522	0.03030641
222371_at	NA	NA	NA	0.62352874	0.03032023
221518_s_at	55031	USP47	ubiquitin specific peptidase 47	-0.61850364	0.03032754
203544_s_at	8027	STAM	signal transducing adaptor molecule (SH3 domain and ITAM motif) 1	-1.0984176	0.03039072
1554462_a_at	4189	DNAJB9	DnaJ (Hsp40) homolog, subfamily B, member 9	-0.88064318	0.03040403
202371_at	79921	TCEAL4	transcription elongation factor A (SII)-like 4	-0.94383783	0.03043408
225545_at	4 | 101930123	EEF2K | LOC10193012	eukaryotic elongation factor 2 kinase	-1.02460716	0.03044922
229428_at	2 | 100652748	TIMM23 | TIMM23B	translocase of inner mitochondrial membrane 23 homolog (yeast) | translocase of inn	-0.74969083	0.03049043
222984_at	51247	PAIP2	poly(A) binding protein interacting protein 2	-0.49784541	0.03052324
1554999_at	153020	RASGEF1B	RasGEF domain family, member 1B	1.37838415	0.03054835
201343_at	7322	UBE2D2	ubiquitin-conjugating enzyme E2D 2	-0.50824784	0.03058152
213762_x_at	7 | 101928747	RBMX | SNORD61 | LC	RNA binding motif protein, X-linked | small nucleolar RNA, C/D box 61 | uncharacteri:	-0.45486283	0.03063487
226048_at	5599	MAPK8	mitogen-activated protein kinase 8	-0.56426069	0.03067591
223136_at	51390	AIG1	androgen-induced 1	-1.55752671	0.0307128
224705_s_at	27327	TNRC6A	trinucleotide repeat containing 6A	-0.89074948	0.03075249
216988_s_at	8073	PTP4A2	protein tyrosine phosphatase type IVA, member 2	-0.56160956	0.03081021
219679_s_at	51322	WAC	WW domain containing adaptor with coiled-coil	-0.94040651	0.03086914
208933_s_at	3964	LGALS8	lectin, galactoside-binding, soluble, 8	-0.97130822	0.03100415
209620_s_at	22	ABCB7	ATP-binding cassette, sub-family B (MDR/TAP), member 7	-0.50856538	0.03113647
217839_at	10342	TFG	TRK-fused gene	-0.60166207	0.0311458
212132_at	26065	LSM14A	LSM14A, SCD6 homolog A (S. cerevisiae)	-0.50742512	0.03114581
226867_at	55667	DENND4C	DENN/MADD domain containing 4C	-1.29032671	0.03115187
200768_s_at	4144	MAT2A	methionine adenosyltransferase II, alpha	0.85647906	0.03119847
209518_at	6602	SMARCD1	SWI/SNF related, matrix associated, actin dependent regulator of chromatin, subfami	-0.58264272	0.03122193
225177_at	80223	RAB11FIP1	RAB11 family interacting protein 1 (class I)	0.87755876	0.03128854
1554616_at	5271	SERPINB8	serpin peptidase inhibitor, clade B (ovalbumin), member 8	0.76842462	0.03129153
231780_at	26301	GBGT1	globoside alpha-1,3-N-acetylgalactosaminyltransferase 1	-1.06251761	0.03143477
235174_s_at	100128822	LINC01003	long intergenic non-protein coding RNA 1003	0.89312255	0.03152878
212687_at	5 | 100288695	LIMS1 | LIMS3 | LIMS	LIM and senescent cell antigen-like domains 1 | LIM and senescent cell antigen-like d	-1.03401322	0.03154937
217118_s_at	23313	KIAA0930	KIAA0930	0.95440202	0.03155527
201795_at	3930	LBR	lamin B receptor	-1.072131	0.03162731
201310_s_at	9315	NREP	neuronal regeneration related protein	1.17368525	0.031646
211559_s_at	901	CCNG2	cyclin G2	-1.00764778	0.03168596
202883_s_at	5519	PPP2R1B	protein phosphatase 2, regulatory subunit A, beta	-1.06550887	0.03180447
223271_s_at	51496	CTDSPL2	CTD (carboxy-terminal domain, RNA polymerase II, polypeptide A) small phosphatase	-0.87641115	0.0318453
232979_at	406902	MIR10A	microRNA 10a	-1.60642458	0.03187096
218582_at	54708	MARCH5	membrane-associated ring finger (C3HC4) 5	-0.7110445	0.03199323
211858_x_at	2778	GNAS	GNAS complex locus	-0.53178463	0.0320744
207104_x_at	10859	LILRB1	leukocyte immunoglobulin-like receptor, subfamily B (with TM and ITIM domains), me	0.7919064	0.03211029
225010_at	8030	CCDC6	coiled-coil domain containing 6	-0.70006932	0.03215367
202272_s_at	23219	FBXO28	F-box protein 28	-0.72308541	0.03220302
203209_at	5985	RFC5	replication factor C (activator 1) 5, 36.5kDa	-0.81681305	0.03230133
212273_x_at	2778	GNAS	GNAS complex locus	-0.4707823	0.03230477
208914_at	23062	GGA2	golgi-associated, gamma adaptin ear containing, ARF binding protein 2	0.69952686	0.03231124
223538_at	3293 | 728492	SERF1A | SERF1B	small EDRK-rich factor 1A (telomeric) | small EDRK-rich factor 1B (centromeric)	-0.72476023	0.03245219
224492_s_at	199692	ZNF627	zinc finger protein 627	-1.16728503	0.03248286
235233_s_at	10691	GMEB1	glucocorticoid modulatory element binding protein 1	-0.77682097	0.03254524
218149_s_at	55893	ZNF395	zinc finger protein 395	-0.9529715	0.03254534
221517_s_at	9440	MED17	mediator complex subunit 17	-1.02195724	0.03256347
209704_at	22823	MTF2	metal response element binding transcription factor 2	-1.06010153	0.03258925
229790_at	7014	TERF2	telomeric repeat binding factor 2	-0.7388569	0.0325918
204497_at	115	ADCY9	adenylate cyclase 9	2.10073086	0.03264387
209921_at	23657	SLC7A11	solute carrier family 7 (anionic amino acid transporter light chain, xc- system), memt	-1.23000824	0.03265808
201926_s_at	1604	CD55	CD55 molecule, decay accelerating factor for complement (Cromer blood group)	0.62581952	0.03267431
207983_s_at	10735	STAG2	stromal antigen 2	-0.67952332	0.0326756
210706_s_at	11237	RNF24	ring finger protein 24	-0.76948815	0.03271467
203775_at	10165	SLC25A13	solute carrier family 25 (aspartate/glutamate carrier), member 13	-0.74639907	0.03275589
228642_at	100506311	HOTAIRM1	HOXA transcript antisense RNA, myeloid-specific 1	-1.24370416	0.0327616
227508_at	NA	NA	NA	-0.86595146	0.03285626
244811_at	55023	PHIP	pleckstrin homology domain interacting protein	-0.85547228	0.03286022
222846_at	51762	RAB8B	RAB8B, member RAS oncogene family	-1.32384017	0.03288529
208614_s_at	2317	FLNB	filamin B, beta	1.38057155	0.03292051
213282_at	139322	APOOL	apolipoprotein O-like	-0.73851437	0.03300074
205550_s_at	9577	BRE	brain and reproductive organ-expressed (TNFRSF1A modulator)	0.84161788	0.03307458
1560741_at	6638 | 8926	SNRPN | SNURF	small nuclear ribonucleoprotein polypeptide N | SNRPN upstream reading frame	1.63096144	0.03308928
202251_at	9129	PRPF3	pre-mRNA processing factor 3	-0.45917176	0.03313815
218486_at	8462	KLF11	Kruppel-like factor 11	1.25909941	0.03324278
204520_x_at	23774	BRD1	bromodomain containing 1	-0.71349328	0.03324854
208707_at	1983	EIF5	eukaryotic translation initiation factor 5	0.74237306	0.03338194
215001_s_at	2752	GLUL	glutamate-ammonia ligase	-0.54013976	0.03348183
211748_x_at	5730	PTGDS	prostaglandin D2 synthase 21kDa (brain)	-0.75718917	0.03348425
223344_s_at	58475	MS4A7	membrane-spanning 4-domains, subfamily A, member 7	1.30242397	0.03348824
44111_at	26276	VPS33B	vacuolar protein sorting 33 homolog B (yeast)	-0.58653057	0.033506
201800_s_at	5007	OSBP	oxysterol binding protein	-0.65390928	0.03356391
208146_s_at	54504	CPVL	carboxypeptidase, vitellogenic-like	-1.93637925	0.03364976
208936_x_at	3964	LGALS8	lectin, galactoside-binding, soluble, 8	-0.56990828	0.0337567
214097_at	6227	RPS21	ribosomal protein S21	0.78606477	0.0337706
201664_at	10051	SMC4	structural maintenance of chromosomes 4	-1.28482631	0.03380196
212666_at	57154	SMURF1	SMAD specific E3 ubiquitin protein ligase 1	-0.9749068	0.03382874
203135_at	6908	TBP	TATA box binding protein	-0.61717574	0.03388537
214442_s_at	9063	PIAS2	protein inhibitor of activated STAT, 2	-0.59905216	0.03389673
207643_s_at	7132	TNFRSF1A	tumor necrosis factor receptor superfamily, member 1A	-1.06297319	0.03404368
204299_at	10772	SRSF10	serine/arginine-rich splicing factor 10	-0.67280267	0.03417183
231090_s_at	196528	ARID2	AT rich interactive domain 2 (ARID, RFX-like)	-0.72223441	0.0342118
205608_s_at	284	ANGPT1	angiopoietin 1	-2.18819126	0.03421945
209095_at	1738	DLD	dihydrolipoamide dehydrogenase	-0.68846156	0.034257
204622_x_at	4929	NR4A2	nuclear receptor subfamily 4, group A, member 2	1.03962365	0.03426473

202941_at	4729	NDUFV2	NADH dehydrogenase (ubiquinone) flavoprotein 2, 24kDa	-0.43832242	0.03449293
209451_at	10010	TANK	TRAF family member-associated NFKB activator	-1.11619675	0.03453276
227861_at	153396	TMEM161B	transmembrane protein 161B	-0.81910213	0.03467419
218268_at	64786	TBC1D15	TBC1 domain family, member 15	-0.58337162	0.03473938
224934_at	81555	YIPF5	Yip1 domain family, member 5	-0.77193402	0.03483742
212673_at	23173	METAP1	methionyl aminopeptidase 1	-0.58197966	0.03484205
225456_at	5469	MED1	mediator complex subunit 1	-0.94309351	0.03486572
214450_at	1521	CTSW	cathepsin W	-1.24980299	0.03487361
204090_at	8859	STK19	serine/threonine kinase 19	-0.44543362	0.03492694
201560_at	25932	CLIC4	chloride intracellular channel 4	-1.13976744	0.03499194
210438_x_at	6738	TROVE2	TROVE domain family, member 2	-0.87293721	0.03503854
211955_at	3843	IPO5	importin 5	-0.52795519	0.03504741
1557278_s_at	3842	TNPO1	transportin 1	1.39970267	0.03511465
224786_at	60592	SCOC	short coiled-coil protein	-1.13268342	0.03513487
232095_at	NA	NA	NA	0.96103954	0.03514164
213361_at	23424	TDRD7	tudor domain containing 7	0.82653642	0.03515777
208698_s_at	4841	NONO	non-POU domain containing, octamer-binding	-0.8004832	0.03523778
202629_at	10513	APPBP2	amyloid beta precursor protein (cytoplasmic tail) binding protein 2	-0.98990837	0.03526943
200693_at	10971	YWHAQ	tyrosine 3-monooxygenase/tryptophan 5-monooxygenase activation protein, theta	-0.53951179	0.03527158
208800_at	6731	SRP72	signal recognition particle 72kDa	-0.57551767	0.03527604
200632_s_at	10397	NDRG1	N-myc downstream regulated 1	0.70433928	0.03537097
201603_at	4659	PPP1R12A	protein phosphatase 1, regulatory subunit 12A	-0.72300152	0.03539283
213795_s_at	5786	PTPRA	protein tyrosine phosphatase, receptor type, A	-0.64069144	0.03540465
225278_at	5565	PRKAB2	protein kinase, AMP-activated, beta 2 non-catalytic subunit	-1.07180595	0.035427
200626_s_at	9782 | 724102	MATR3 | SNHG4	matrin 3 | small nucleolar RNA host gene 4	-0.47252851	0.03543206
225074_at	84932	RAB2B	RAB2B, member RAS oncogene family	-0.81808442	0.03552982
201394_s_at	10181	RBM5	RNA binding motif protein 5	-0.62503386	0.03554095
218165_at	64769	MEAF6	MYST/Esa1-associated factor 6	-0.61188254	0.03554853
236685_at	NA	NA	NA	-1.13483887	0.03556705
234942_s_at	9967 | 116092	THRAP3 | DNTTIP1	thyroid hormone receptor associated protein 3 | deoxynucleotidyltransferase, termina	-0.90006937	0.03561367
208842_s_at	26003	GORASP2	golgi reassembly stacking protein 2, 55kDa	-0.59285565	0.03562378
205639_at	313	AOAH	acyloxyacyl hydrolase (neutrophil)	1.12755146	0.0357445
200880_at	3301	DNAJA1	DnaJ (Hsp40) homolog, subfamily A, member 1	0.65411553	0.03575924
222574_s_at	79665	DHX40	DEAH (Asp-Glu-Ala-His) box polypeptide 40	-0.72539623	0.03577721
239151_at	399761	BMS1P5	BMS1 pseudogene 5	-0.88348058	0.03579431
224049_at	89822	KCNK17	potassium channel, two pore domain subfamily K, member 17	-0.96103795	0.03581488
222430_s_at	51441	YTHDF2	YTH N(6)-methyladenosine RNA binding protein 2	-0.72325532	0.03581754
219055_at	55133	SRBD1	S1 RNA binding domain 1	-1.04898982	0.03582
225477_s_at	7182	NR2C2	nuclear receptor subfamily 2, group C, member 2	-0.88445762	0.03582609
211015_s_at	3308	HSPA4	heat shock 70kDa protein 4	-0.71789389	0.03595381
38149_at	9938	ARHGAP25	Rho GTPase activating protein 25	-0.98873021	0.035959
208407_s_at	) | 100528016	CTNND1 | TMX2-CTNN	catenin (cadherin-associated protein), delta 1 | TMX2-CTNND1 readthrough (NMD ca	-1.49253837	0.03595916
202530_at	1432	MAPK14	mitogen-activated protein kinase 14	-0.85374286	0.03598753
215758_x_at	81931	ZNF93	zinc finger protein 93	-0.66378455	0.03602662
200948_at	8079	MLF2	myeloid leukemia factor 2	-0.687591	0.03613708
209267_s_at	64116	SLC39A8	solute carrier family 39 (zinc transporter), member 8	-0.9164941	0.03614341
217959_s_at	) | 100500840	TRAPPC4 | MIR3656	trafficking protein particle complex 4 | microRNA 3656	-0.88995515	0.0362696
211133_x_at	11025	LILRB3	leukocyte immunoglobulin-like receptor, subfamily B (with TM and ITIM domains), me	0.46121683	0.0362844
200721_s_at	10121	ACTR1A	ARP1 actin-related protein 1 homolog A, centractin alpha (yeast)	-0.59735717	0.0363529
227860_at	56265	CPXM1	carboxypeptidase X (M14 family), member 1	-1.40238378	0.03643034
201146_at	4780	NFE2L2	nuclear factor, erythroid 2-like 2	-0.83977877	0.03646684
204128_s_at	5983	RFC3	replication factor C (activator 1) 3, 38kDa	-0.95517255	0.03647226
202642_s_at	8295	TRRAP	transformation/transcription domain-associated protein	-0.95462653	0.03654095
1557813_at	NA	NA	NA	-1.45271043	0.03657884
211450_s_at	2956	MSH6	mutS homolog 6	-1.29065332	0.03667571
244860_at	NA	NA	NA	1.28782646	0.03668829
214882_s_at	5427 | 693221	SRSF2 | MIR636	serine/arginine-rich splicing factor 2 | microRNA 636	-0.46212702	0.03671066
230110_at	255231	MCOLN2	mucolipin 2	1.65936974	0.03671239
243888_at	NA	NA	NA	-0.99665712	0.03684121
208498_s_at	280 | 648740	AMY1A | AMY1B | AMY	amylase, alpha 1A (salivary) | amylase, alpha 1B (salivary) | amylase, alpha 1C (sali	1.26089351	0.03684999
238534_at	9208	LRRFIP1	leucine rich repeat (in FLII) interacting protein 1	1.00333095	0.03689091
203745_at	3052	HCCS	holocytochrome c synthase	-0.87359273	0.03691428
222367_at	9005 | 440253	WHAMMP3 | WHAMMP	WAS protein homolog associated with actin, golgi membranes and microtubules pseu	1.17980741	0.03708174
232053_x_at	57414	RHBDD2	rhomboid domain containing 2	-0.93381593	0.03709336
204900_x_at	8819	SAP30	Sin3A-associated protein, 30kDa	-1.99007786	0.0371568
212381_at	23358	USP24	ubiquitin specific peptidase 24	-0.59739681	0.03728342
233878_s_at	22803	XRN2	5'-3' exoribonuclease 2	-1.07195206	0.0373002
201676_x_at	5682	PSMA1	proteasome (prosome, macropain) subunit, alpha type, 1	-0.51084562	0.03735694
209287_s_at	10602	CDC42EP3	CDC42 effector protein (Rho GTPase binding) 3	0.84105872	0.03737244
227187_at	79872	CBLL1	Cbl proto-oncogene-like 1, E3 ubiquitin protein ligase	-1.10192933	0.03739573
238714_at	NA	NA	NA	-0.91609295	0.03748909
223296_at	84275	SLC25A33	solute carrier family 25 (pyrimidine nucleotide carrier), member 33	0.96504984	0.03755787
222630_at	64864	RFX7	regulatory factor X, 7	-0.86589073	0.03758843
226763_at	91404	SESTD1	SEC14 and spectrin domains 1	-1.32016698	0.03762208
226692_at	4 | 100302254	SERF2 | HYPK | MIR12	small EDRK-rich factor 2 | huntingtin interacting protein K | microRNA 1282	0.68990032	0.03763581
1558836_at	NA	NA	NA	-0.83106386	0.03763792
216705_s_at	100	ADA	adenosine deaminase	-1.17632483	0.03764432
218308_at	10460	TACC3	transforming, acidic coiled-coil containing protein 3	-0.92148248	0.03765731
204391_x_at	8805	TRIM24	tripartite motif containing 24	-0.54537924	0.03766577
228415_at	8905	AP1S2	adaptor-related protein complex 1, sigma 2 subunit	0.71158408	0.03776478
202365_at	84747	UNC119B	unc-119 homolog B (C. elegans)	-0.84567203	0.03778268
213857_s_at	961	CD47	CD47 molecule	-0.75475151	0.03785065
214548_x_at	2778	GNAS	GNAS complex locus	-0.50998699	0.03785923
217969_at	738	VPS51	vacuolar protein sorting 51 homolog (S. cerevisiae)	-0.67891006	0.03796111
210425_x_at	3015 | 440270	GOLGA8A | GOLGA8B	golgin A8 family, member A | golgin A8 family, member B	-1.48463527	0.03800087
212652_s_at	8723	SNX4	sorting nexin 4	-0.58232956	0.03808725
212505_s_at	23383	MAU2	MAU2 sister chromatid cohesion factor	-0.56759124	0.03810878
1555950_a_at	1604	CD55	CD55 molecule, decay accelerating factor for complement (Cromer blood group)	0.60570795	0.03813208
221551_x_at	27090	ST6GALNAC4	ST6 (alpha-N-acetyl-neuraminyl-2,3-beta-galactosyl-1,3)-N-acetylgalactosaminide al	-0.9441763	0.03821676
225468_at	219988	PATL1	protein associated with topoisomerase II homolog 1 (yeast)	-0.73928893	0.03821826
244508_at	989	SEPT7	septin 7	1.01143565	0.03828574
226399_at	79982	DNAJB14	DnaJ (Hsp40) homolog, subfamily B, member 14	-1.13886371	0.03842371
212180_at	1399	CRKL	v-crk avian sarcoma virus CT10 oncogene homolog-like	-0.62059512	0.03842443
209252_at	23438	HARS2	histidyl-tRNA synthetase 2, mitochondrial	-0.76212975	0.03845276
219457_s_at	79890	RIN3	Ras and Rab interactor 3	-0.91489824	0.03856315
224865_at	84188	FAR1	fatty acyl CoA reductase 1	0.74203443	0.03866343
244753_at	NA	NA	NA	1.23623016	0.03866681
238909_at	6281	S100A10	S100 calcium binding protein A10	1.81358094	0.03866854
236892_s_at	404266	HOXB-AS3	HOXB cluster antisense RNA 3	-3.07745	0.0388139
201263_at	6897	TARS	threonyl-tRNA synthetase	-0.70602572	0.03904023
228325_at	23514	SPIDR	scaffolding protein involved in DNA repair	1.34429272	0.0390817
212382_at	6925	TCF4	transcription factor 4	-1.69383496	0.03908419
202084_s_at	6397	SEC14L1	SEC14-like 1 (S. cerevisiae)	-0.70496816	0.03914138
200072_s_at	4670	HNRNPM	heterogeneous nuclear ribonucleoprotein M	-0.70537552	0.03915489

223262_s_at	26127	FGFR1OP2	FGFR1 oncogene partner 2	-0.61794266	0.03916512
203248_at	7572	ZNF24	zinc finger protein 24	-0.80165562	0.03919676
202253_s_at	1785	DNM2	dynamin 2	-0.63112664	0.03923745
1557197_a_at	3958	LGALS3	lectin, galactoside-binding, soluble, 3	1.23076939	0.03924638
229309_at	153	ADRB1	adrenoceptor beta 1	-1.98416959	0.03933904
212986_s_at	11011	TLK2	tousled-like kinase 2	-0.76680445	0.03940544
218119_at	2 | 100652748	TIMM23 | TIMM23B	translocase of inner mitochondrial membrane 23 homolog (yeast) | translocase of inn	-0.77830461	0.03941387
216996_s_at	22868	FASTKD2	FAST kinase domains 2	-0.56470295	0.0394525
217885_at	55705	IPO9	importin 9	-0.60595541	0.03946139
226775_at	56943	ENY2	enhancer of yellow 2 homolog (Drosophila)	-0.74522216	0.03946313
218348_s_at	29066	ZC3H7A	zinc finger CCCH-type containing 7A	-0.62860689	0.03954334
201718_s_at	2037	EPB41L2	erythrocyte membrane protein band 4.1-like 2	-1.36395741	0.03954353
222697_s_at	55347	ABHD10	abhydrolase domain containing 10	-0.9700292	0.03960519
215236_s_at	8301	PICALM	phosphatidylinositol binding clathrin assembly protein	-1.14412077	0.03964171
201755_at	4174	MCM5	minichromosome maintenance complex component 5	-0.90715843	0.03965975
221255_s_at	83460	EMC6	ER membrane protein complex subunit 6	-0.43944693	0.03982045
207945_s_at	1453	CSNK1D	casein kinase 1, delta	-0.80477414	0.03982394
203633_at	1374	CPT1A	carnitine palmitoyltransferase 1A (liver)	-0.66217154	0.03984815
223044_at	30061	SLC40A1	solute carrier family 40 (iron-regulated transporter), member 1	-1.52055097	0.0398691
228123_s_at	26090	ABHD12	abhydrolase domain containing 12	-0.72219328	0.0398778
242946_at	963	CD53	CD53 molecule	0.92252087	0.03988206
242390_at	NA	NA	NA	1.52358173	0.03993782
228980_at	4 | 100529207	RFFL | RAD51L3-RFFL	ring finger and FYVE-like domain containing E3 ubiquitin protein ligase | RAD51L3-RF	-0.49607278	0.03994179
227577_at	149371	EXOC8	exocyst complex component 8	-0.83369237	0.04001781
202558_s_at	6782	HSPA13	heat shock protein 70kDa family, member 13	-0.78731754	0.04011066
224800_at	57590	WDFY1	WD repeat and FYVE domain containing 1	0.6290145	0.04015968
212871_at	8550	MAPKAPK5	mitogen-activated protein kinase-activated protein kinase 5	-0.58431666	0.04018052
207541_s_at	5394	EXOSC10	exosome component 10	-0.60818948	0.04022075
223021_x_at	51534	VTA1	vesicle (multivesicular body) trafficking 1	-1.02709303	0.04023769
203738_at	55322	C5orf22	chromosome 5 open reading frame 22	-0.69148961	0.04028362
226776_at	56943	ENY2	enhancer of yellow 2 homolog (Drosophila)	-0.74608766	0.04037424
202846_s_at	5279	PIGC	phosphatidylinositol glycan anchor biosynthesis, class C	-0.52648279	0.04039821
223988_x_at	64745	METTL17	methyltransferase like 17	-0.60395065	0.04049566
224990_at	201895	SMIM14	small integral membrane protein 14	1.35961715	0.04057642
201549_x_at	10765	KDM5B	lysine (K)-specific demethylase 5B	-1.11029519	0.04065034
202406_s_at	7073	TIAL1	TIA1 cytotoxic granule-associated RNA binding protein-like 1	-0.46962625	0.04068428
201093_x_at	6389	SDHA	succinate dehydrogenase complex, subunit A, flavoprotein (Fp)	-0.41582015	0.04070427
226684_at	55102	ATG2B	autophagy related 2B	-0.83971319	0.04079052
201200_at	8804	CREG1	cellular repressor of E1A-stimulated genes 1	-0.56095941	0.04080009
214949_at	NA	NA	NA	-0.91320643	0.04081758
217971_at	8649	LAMTOR3	late endosomal/lysosomal adaptor, MAPK and MTOR activator 3	-0.72094096	0.04081946
203189_s_at	3 | 102465669	NDUFS8 | MIR4691 | I	NADH dehydrogenase (ubiquinone) Fe-S protein 8, 23kDa (NADH-coenzyme Q reduct	-0.59634352	0.04092171
224862_at	2776	GNAQ	guanine nucleotide binding protein (G protein), q polypeptide	-0.80153465	0.04102517
225111_s_at	63908	NAPB	N-ethylmaleimide-sensitive factor attachment protein, beta	-0.81830596	0.04107207
210296_s_at	5828	PEX2	peroxisomal biogenesis factor 2	-0.86424358	0.04107594
223067_at	51503	CWC15	CWC15 spliceosome-associated protein	-0.66120588	0.04113652
202862_at	2184	FAH	fumarylacetoacetate hydrolase (fumarylacetoacetase)	-1.03731144	0.04129504
225501_at	84295	PHF6	PHD finger protein 6	-0.73120655	0.04134036
204885_s_at	10232	MSLN	mesothelin	2.28805122	0.04140221
212519_at	7324	UBE2E1	ubiquitin-conjugating enzyme E2E 1	-0.50158814	0.04147191
225140_at	51274	KLF3	Kruppel-like factor 3 (basic)	-1.18785981	0.04162804
202126_at	8899	PRPF4B	pre-mRNA processing factor 4B	-0.59060439	0.04166934
211653_x_at	1646	AKR1C2	aldo-keto reductase family 1, member C2	-1.34090849	0.04173451
225653_at	9392	TGFBRAP1	transforming growth factor, beta receptor associated protein 1	-0.73037787	0.04178527
211034_s_at	283450	HECTD4	HECT domain containing E3 ubiquitin protein ligase 4	-0.69993541	0.0418494
202810_at	4733	DRG1	developmentally regulated GTP binding protein 1	-0.43257755	0.04188198
221827_at	10616	RBCK1	RanBP-type and C3HC4-type zinc finger containing 1	-0.42500479	0.04194065
203529_at	5537	PPP6C	protein phosphatase 6, catalytic subunit	-0.48379819	0.04196514
210365_at	3 | 101928269	RUNX1 | LOC1005064	runt-related transcription factor 1 | uncharacterized LOC100506403 | uncharacterizec	-1.09521334	0.04199912
1553587_a_at	56655	POLE4	polymerase (DNA-directed), epsilon 4, accessory subunit	0.81686347	0.04203404
212008_at	23190	UBXN4	UBX domain protein 4	-0.98849724	0.0421331
226331_at	56987	BBX	bobby sox homolog (Drosophila)	-1.23242803	0.04219336
206508_at	970	CD70	CD70 molecule	0.89132552	0.04221323
224640_at	121665	SPPL3	signal peptide peptidase like 3	-0.48863079	0.04227761
218467_at	56984	PSMG2	proteasome (prosome, macropain) assembly chaperone 2	-0.41396939	0.04238463
201595_s_at	55854	ZC3H15	zinc finger CCCH-type containing 15	-0.47501328	0.04243148
209053_s_at	7468	WHSC1	Wolf-Hirschhorn syndrome candidate 1	-1.1030272	0.04247219
208759_at	23385	NCSTN	nicastrin	-0.58857792	0.04251792
212764_at	6935	ZEB1	zinc finger E-box binding homeobox 1	-1.66854125	0.04252614
208051_s_at	10605	PAIP1	poly(A) binding protein interacting protein 1	-0.93360461	0.0425734
208821_at	6628	SNRPB	small nuclear ribonucleoprotein polypeptides B and B1	-0.54988384	0.04257528
242506_at	NA	NA	NA	0.56479635	0.04258508
211727_s_at	1353	COX11	COX11 cytochrome c oxidase copper chaperone	-0.76692219	0.0425897
202560_s_at	26097	CHTOP	chromatin target of PRMT1	-0.66447219	0.04260562
212721_at	140890	SREK1	splicing regulatory glutamine/lysine-rich protein 1	-0.63107533	0.04264724
208649_s_at	7415	VCP	valosin containing protein	-0.66527244	0.04268692
201440_at	9416	DDX23	DEAD (Asp-Glu-Ala-Asp) box polypeptide 23	-0.61463467	0.04270623
208913_at	23062	GGA2	golgi-associated, gamma adaptin ear containing, ARF binding protein 2	0.92040432	0.04275363
208969_at	4704	NDUFA9	NADH dehydrogenase (ubiquinone) 1 alpha subcomplex, 9, 39kDa	-0.47245969	0.04277266
209256_s_at	23008	KLHDC10	kelch domain containing 10	-0.68527532	0.04278075
225466_at	219988	PATL1	protein associated with topoisomerase II homolog 1 (yeast)	-0.61582238	0.04279433
237018_at	NA	NA	NA	1.10676431	0.04290234
218414_s_at	54820	NDE1	nudE neurodevelopment protein 1	-0.43094114	0.04293789
227265_at	10875	FGL2	fibrinogen-like 2	1.92911022	0.04294968
219711_at	54807	ZNF586	zinc finger protein 586	-1.0522792	0.04298748
219236_at	79957	PAQR6	progestin and adipoQ receptor family member VI	-0.62237869	0.04300899
204776_at	7060	THBS4	thrombospondin 4	0.99759059	0.04301962
213016_at	56987	BBX	bobby sox homolog (Drosophila)	-1.11416561	0.04303977
37966_at	29780	PARVB	parvin, beta	-0.91261455	0.04305445
226117_at	92610	TIFA	TRAF-interacting protein with forkhead-associated domain	-1.17738525	0.04307348
212885_at	10199	MPHOSPH10	M-phase phosphoprotein 10 (U3 small nucleolar ribonucleoprotein)	-0.54787456	0.0430788
209143_s_at	1207	CLNS1A	chloride channel, nucleotide-sensitive, 1A	-0.5932319	0.04313129
200764_s_at	1495	CTNNA1	catenin (cadherin-associated protein), alpha 1, 102kDa	-0.95562733	0.04313931
208826_x_at	3094	HINT1	histidine triad nucleotide binding protein 1	-0.55954287	0.04313946
219575_s_at	54146 | 84342	PDF | COG8	peptide deformylase (mitochondrial) | component of oligomeric golgi complex 8	-0.57578603	0.04314942
218409_s_at	64215	DNAJC1	DnaJ (Hsp40) homolog, subfamily C, member 1	-0.76473865	0.04316209
201608_s_at	11137	PWP1	PWP1 homolog (S. cerevisiae)	-0.44341078	0.04318439
228654_at	139886	SPIN4	spindlin family, member 4	-1.22041177	0.04318885
213153_at	23067	SETD1B	SET domain containing 1B	-0.42358943	0.04320367
212628_at	5586	PKN2	protein kinase N2	-0.75091371	0.04321446
238156_at	NA	NA	NA	0.84902751	0.04330679
218219_s_at	55915	LANCL2	LanC lantibiotic synthetase component C-like 2 (bacterial)	-0.77527489	0.04332466
228442_at	4773	NFATC2	nuclear factor of activated T-cells, cytoplasmic, calcineurin-dependent 2	-0.90590568	0.04334348
213000_at	23515	MORC3	MORC family CW-type zinc finger 3	-0.51535334	0.04337679

219694_at	54491	FAM105A	family with sequence similarity 105, member A	1.45476239	0.04339347
202729_s_at	4052	LTBP1	latent transforming growth factor beta binding protein 1	-1.67347326	0.04340615
205229_s_at	1690	COCH	cochlin	-1.59427458	0.04343248
225535_s_at	2 | 100652748	TIMM23 | TIMM23B	translocase of inner mitochondrial membrane 23 homolog (yeast) | translocase of inn	-0.63712809	0.0434557
208666_s_at	6767	ST13	suppression of tumorigenicity 13 (colon carcinoma) (Hsp70 interacting protein)	-0.61453093	0.0434997
209186_at	488	ATP2A2	ATPase, Ca++ transporting, cardiac muscle, slow twitch 2	-0.69528551	0.04355502
204132_s_at	2309 | 2310	FOXO3 | FOXO3B	forkhead box O3 | forkhead box O3B pseudogene	-1.23264124	0.04358651
201368_at	678	ZFP36L2	ZFP36 ring finger protein-like 2	-0.94385667	0.0436047
212688_at	5291	PIK3CB	phosphatidylinositol-4,5-bisphosphate 3-kinase, catalytic subunit beta	-0.96903467	0.04362538
238880_at	2971	GTF3A	general transcription factor IIIA	0.72417238	0.0436565
1555241_at	401466	C8orf59	chromosome 8 open reading frame 59	-0.78777815	0.04383827
225848_at	155061	ZNF746	zinc finger protein 746	-0.61544563	0.04389049
234157_at	NA	NA	NA	1.29793689	0.04389959
226710_at	414919	C8orf82	chromosome 8 open reading frame 82	0.58516956	0.04390771
225671_at	124976	SPNS2	spinster homolog 2 (Drosophila)	-0.97875698	0.04398003
231109_at	NA	NA	NA	1.47410782	0.04400266
213473_at	8315	BRAP	BRCA1 associated protein	-0.63064256	0.04410641
221235_s_at	9392	TGFBRAP1	transforming growth factor, beta receptor associated protein 1	-0.96954306	0.04416666
213258_at	7035	TFPI	tissue factor pathway inhibitor (lipoprotein-associated coagulation inhibitor)	-1.73159995	0.04420612
205087_at	25950	RWDD3	RWD domain containing 3	-0.64601376	0.04427446
218025_s_at	10455	ECI2	enoyl-CoA delta isomerase 2	-1.15802465	0.0442844
201023_at	6879	TAF7	TAF7 RNA polymerase II, TATA box binding protein (TBP)-associated factor, 55kDa	-0.63578214	0.04429054
201273_s_at	6726	SRP9	signal recognition particle 9kDa	-0.69539905	0.04429481
210038_at	5588	PRKCQ	protein kinase C, theta	-1.1993749	0.0442984
212750_at	26051	PPP1R16B	protein phosphatase 1, regulatory subunit 16B	-1.1883823	0.04435239
239405_at	NA	NA	NA	1.15290582	0.04435269
216457_s_at	10291	SF3A1	splicing factor 3a, subunit 1, 120kDa	-0.48660103	0.04445426
213864_s_at	4673	NAP1L1	nucleosome assembly protein 1-like 1	-0.4918776	0.04447995
203345_s_at	22823	MTF2	metal response element binding transcription factor 2	-0.67175789	0.04450004
224927_at	170954	PPP1R18	protein phosphatase 1, regulatory subunit 18	-0.74791355	0.04453162
225470_at	129401	NUP35	nucleoporin 35kDa	-0.88964196	0.04455316
210184_at	3687	ITGAX	integrin, alpha X (complement component 3 receptor 4 subunit)	0.75046915	0.04456165
201773_at	23394	ADNP	activity-dependent neuroprotector homeobox	-0.68955098	0.04456179
223130_s_at	29116	MYLIP	myosin regulatory light chain interacting protein	0.86099467	0.04457216
218111_s_at	55907	CMAS	cytidine monophosphate N-acetylneuraminic acid synthetase	-1.21720282	0.04457931
234915_s_at	8562	DENR	density-regulated protein	-0.65218814	0.04463071
202583_s_at	10048	RANBP9	RAN binding protein 9	-0.75011344	0.04467187
210943_s_at	1130	LYST	lysosomal trafficking regulator	1.15496411	0.04468184
208268_at	10863	ADAM28	ADAM metallopeptidase domain 28	-1.56867823	0.044721
1557263_s_at	102724814	LOC102724814	uncharacterized LOC102724814	0.5804921	0.04477102
201701_s_at	10424	PGRMC2	progesterone receptor membrane component 2	-0.99020875	0.04477492
218106_s_at	55173	MRPS10	mitochondrial ribosomal protein S10	-0.50153202	0.04479888
208642_s_at	7520	XRCC5	X-ray repair complementing defective repair in Chinese hamster cells 5 (double-stran	-0.6791182	0.04480936
224754_at	6667	SP1	Sp1 transcription factor	-0.52057341	0.04482429
225052_at	94107	TMEM203	transmembrane protein 203	-0.66944897	0.04487848
201784_s_at	10944	C11orf58	chromosome 11 open reading frame 58	-0.60028648	0.04493772
1559593_a_at	9441	MED26	mediator complex subunit 26	0.98921436	0.04495189
55093_at	54480	CHPF2	chondroitin polymerizing factor 2	-0.66783301	0.04497275
227284_at	90321	ZNF766	zinc finger protein 766	-0.67866834	0.04499989
226963_at	91408	BTF3L4	basic transcription factor 3-like 4	-0.47631717	0.04506657
203493_s_at	9702	CEP57	centrosomal protein 57kDa	-0.7244479	0.04514771
204767_s_at	2237	FEN1	flap structure-specific endonuclease 1	-1.18205984	0.04515289
211101_x_at	11027	LILRA2	leukocyte immunoglobulin-like receptor, subfamily A (with TM domain), member 2	0.9672859	0.04515291
1567913_at	NA	NA	NA	0.90681705	0.04530219
213161_at	7111 | 158427	TMOD1 | TSTD2	tropomodulin 1 | thiosulfate sulfurtransferase (rhodanese)-like domain containing 2	-0.80751852	0.04530235
222708_s_at	55014	STX17	syntaxin 17	-0.82737221	0.04532816
203211_s_at	8898	MTMR2	myotubularin related protein 2	-0.84772347	0.04549999
206120_at	945	CD33	CD33 molecule	-0.90657549	0.04557514
220952_s_at	54477	PLEKHA5	pleckstrin homology domain containing, family A member 5	-0.98535218	0.04562421
235879_at	NA	NA	NA	0.87114072	0.04563504
225639_at	8935	SKAP2	src kinase associated phosphoprotein 2	-1.74018953	0.04571189
233898_s_at	26127	FGFR1OP2	FGFR1 oncogene partner 2	-1.24018932	0.04575147
236248_x_at	93624	TADA2B	transcriptional adaptor 2B	0.68895619	0.04582192
239893_at	NA	NA	NA	-1.45234864	0.04585731
227249_at	54820	NDE1	nudE neurodevelopment protein 1	-0.6033675	0.04595739
232544_at	NA	NA	NA	1.29573656	0.04598214
210101_x_at	51100	SH3GLB1	SH3-domain GRB2-like endophilin B1	-0.50458013	0.04601904
229444_at	101929243	LOC101929243	uncharacterized LOC101929243	0.66651821	0.04604939
202746_at	9452	ITM2A	integral membrane protein 2A	-2.21380429	0.04610634
219200_at	79072	FASTKD3	FAST kinase domains 3	-0.81821516	0.04612137
212646_at	23180	RFTN1	raftlin, lipid raft linker 1	-0.86490718	0.04612775
213052_at	5576	PRKAR2A	protein kinase, cAMP-dependent, regulatory, type II, alpha	0.47117528	0.04617705
228145_s_at	57541	ZNF398	zinc finger protein 398	-0.75843837	0.04619309
231990_at	3 | 102465133	USP15 | MIR6125	ubiquitin specific peptidase 15 | microRNA 6125	-1.27238867	0.04624044
202469_s_at	11052	CPSF6	cleavage and polyadenylation specific factor 6, 68kDa	-0.57767979	0.0462937
221652_s_at	55726	ASUN	asunder spermatogenesis regulator	-0.88810432	0.04631026
232931_at	23020	SNRNP200	small nuclear ribonucleoprotein 200kDa (U5)	-0.64106916	0.04632826
201353_s_at	11176	BAZ2A	bromodomain adjacent to zinc finger domain, 2A	-0.79631365	0.0463448
209795_at	969	CD69	CD69 molecule	-1.49766625	0.04635723
235113_at	122769	LRR1	leucine rich repeat protein 1	-0.99570529	0.04639002
206148_at	3563	IL3RA	interleukin 3 receptor, alpha (low affinity)	-0.76087304	0.04643663
221918_at	5128	CDK17	cyclin-dependent kinase 17	-0.96641301	0.04654343
203897_at	57149	LYRM1	LYR motif containing 1	-0.73786277	0.04673232
216202_s_at	9517	SPTLC2	serine palmitoyltransferase, long chain base subunit 2	-0.88941012	0.04679629
226547_at	7994	KAT6A	K(lysine) acetyltransferase 6A	-0.85877611	0.04679791
222527_s_at	55696	RBM22	RNA binding motif protein 22	-0.65087729	0.04683696
220199_s_at	64853	AIDA	axin interactor, dorsalization associated	-1.3709563	0.04691893
203984_s_at	842	CASP9	caspase 9, apoptosis-related cysteine peptidase	0.6251349	0.04694401
203696_s_at	5982	RFC2	replication factor C (activator 1) 2, 40kDa	-0.62786893	0.04695639
222186_at	NA	NA	NA	0.93412273	0.04706711
212644_s_at	93487	MAPK1IP1L	mitogen-activated protein kinase 1 interacting protein 1-like	-0.51597267	0.04716839
235692_at	30011	SH3KBP1	SH3-domain kinase binding protein 1	0.93103042	0.04717121
212056_at	23199	GSE1	Gse1 coiled-coil protein	-0.77443431	0.04717823
233931_at	NA	NA	NA	1.12351946	0.04738489
201834_at	5564	PRKAB1	protein kinase, AMP-activated, beta 1 non-catalytic subunit	-0.63272112	0.04742163
1554239_s_at	284273	ZADH2	zinc binding alcohol dehydrogenase domain containing 2	-0.67421385	0.04743987
209728_at	3126	HLA-DRB4	major histocompatibility complex, class II, DR beta 4	2.6245177	0.04750871
215512_at	10299	MARCH6	membrane-associated ring finger (C3HC4) 6, E3 ubiquitin protein ligase	-1.24520077	0.04756768
227002_at	286336	FAM78A	family with sequence similarity 78, member A	-0.82255385	0.04761264
218142_s_at	51185	CRBN	cereblon	-0.70091798	0.04768929
223382_s_at	84937	ZNRF1	zinc and ring finger 1, E3 ubiquitin protein ligase	-0.81399252	0.04772131
212080_at	4297	KMT2A	lysine (K)-specific methyltransferase 2A	-0.95595327	0.04779436
201024_x_at	9669	EIF5B	eukaryotic translation initiation factor 5B	-0.67367282	0.04780923
218633_x_at	55347	ABHD10	abhydrolase domain containing 10	-0.55594157	0.04781713

203320_at	10019	SH2B3	SH2B adaptor protein 3	-0.67252778	0.04782149
201162_at	3490	IGFBP7	insulin-like growth factor binding protein 7	1.11333252	0.04783998
219631_at	29967	LRP12	low density lipoprotein receptor-related protein 12	-1.24772458	0.04787084
225460_at	9117	SEC22C	SEC22 vesicle trafficking protein homolog C (S. cerevisiae)	-0.56007008	0.04789782
219456_s_at	79890	RIN3	Ras and Rab interactor 3	-0.99910927	0.04790968
229391_s_at	441168	FAM26F	family with sequence similarity 26, member F	-1.46432583	0.04791157
226461_at	3219	HOXB9	homeobox B9	-1.12873771	0.04793843
225788_at	88745	RRP36	ribosomal RNA processing 36 homolog (S. cerevisiae)	-0.58755903	0.04797174
236223_s_at	6016	RIT1	Ras-like without CAAX 1	-1.23826907	0.04809153
218049_s_at	28998	MRPL13	mitochondrial ribosomal protein L13	-1.11757743	0.04818084
219690_at	79713	IGFLR1	IGF-like family receptor 1	0.6551861	0.04839326
218118_s_at	2 | 100652748	TIMM23 | TIMM23B	translocase of inner mitochondrial membrane 23 homolog (yeast) | translocase of inn	-0.70157106	0.04842333
201920_at	6574	SLC20A1	solute carrier family 20 (phosphate transporter), member 1	-0.66056807	0.04846205
38487_at	23166	STAB1	stabilin 1	-1.45701573	0.04850123
208792_s_at	1191	CLU	clusterin	-1.73897747	0.04851083
201588_at	9352	TXNL1	thioredoxin-like 1	-0.48025299	0.04860306
57163_at	4 | 102466723	ELOVL1 | MIR6734	ELOVL fatty acid elongase 1 | microRNA 6734	-0.61780116	0.04864978
213617_s_at	25941	TPGS2	tubulin polyglutamylase complex subunit 2	-0.694593	0.0486528
216336_x_at	4493	MT1E	metallothionein 1E	0.79110343	0.04868119
218570_at	5709 | 114971	KBTBD4 | PTPMT1	kelch repeat and BTB (POZ) domain containing 4 | protein tyrosine phosphatase, mito	-0.6969684	0.04868854
216237_s_at	4174	MCM5	minichromosome maintenance complex component 5	-0.88617162	0.04883677
201739_at	6446	SGK1	serum/glucocorticoid regulated kinase 1	1.33670453	0.04893392
223486_at	29083	GTPBP8	GTP-binding protein 8 (putative)	-0.62930593	0.04900452
225551_at	163882	CNST	consortin, connexin sorting protein	-1.0328364	0.04900712
217047_s_at	10144	FAM13A	family with sequence similarity 13, member A	-1.09632939	0.04903995
205210_at	9392	TGFBRAP1	transforming growth factor, beta receptor associated protein 1	-0.76764834	0.04906057
212156_at	23339	VPS39	vacuolar protein sorting 39 homolog (S. cerevisiae)	-0.57828258	0.04910509
227456_s_at	221545	C6orf136	chromosome 6 open reading frame 136	-0.48318798	0.04917568
235170_at	168374	ZNF92	zinc finger protein 92	-0.71293981	0.04919331
218036_x_at	51068	NMD3	NMD3 ribosome export adaptor	-0.81238226	0.04923623
212276_at	23175	LPIN1	lipin 1	-0.93150489	0.04924329
209188_x_at	1810	DR1	down-regulator of transcription 1, TBP-binding (negative cofactor 2)	-0.52746442	0.0494393
223309_x_at	50640	PNPLA8	patatin-like phospholipase domain containing 8	0.61302433	0.04949426
212752_at	23332	CLASP1	cytoplasmic linker associated protein 1	-0.55039346	0.04958885
207911_s_at	9333	TGM5	transglutaminase 5	1.08810644	0.04964696
217732_s_at	9445	ITM2B	integral membrane protein 2B	-0.66102336	0.04976344
201725_at	8872	CDC123	cell division cycle 123	-0.73001118	0.0498618
200950_at	10552	ARPC1A	actin related protein 2/3 complex, subunit 1A, 41kDa	-0.80550131	0.04996528

Supplementary Table 3B. Affymetrix GeneChip® Human Exon 1.0 ST. Top upregulated and top downregulated genes
Analysis of AML patients with low WT1 expression vs. high WT1 expression, ranked by significance (P Value < 0.05).

transcriptclusterid	logFC	P.Value	Gene Symbol
4048241	5.95066654	1.2728E-09	HLA-DRB5
4048265	6.7985275	2.89039E-09	HLA-DRB1
3837257	4.25925529	5.20958E-07	C5AR1
3887210	3.22585211	1.3275E-06	MMP9
3403773	2.80636483	3.4256E-06	CLEC4D
3742285	2.74826196	4.29453E-06	CXCL16
3443183	2.64356906	4.94595E-06	CLEC4E
3869237	2.74438461	6.3724E-06	FPR1
2497161	2.72324536	8.89077E-06	IL18RAP
3992304	-3.32458607	1.03081E-05	SAGE1
3870611	2.56995876	1.52018E-05	LILRA3
2956563	3.39171896	1.73337E-05	CRISP3
2829947	3.00075832	1.97579E-05	TGFBI
2878437	3.69150061	2.172E-05	CD14
3823304	2.32629297	2.49961E-05	CYP4F3
2701071	2.31549486	3.42845E-05	P2RY13
3894727	2.46282732	3.53584E-05	SIRPB1
2527747	2.4121305	3.75824E-05	SLC11A1
2958325	-2.10252972	3.79655E-05	DST
3028011	2.76253192	4.24559E-05	MGAM
3445544	3.3278614	4.26203E-05	PLBD1
2496907	2.95592773	5.23847E-05	IL1R2
2974635	2.56647324	5.27072E-05	VNN2
2372858	2.79347188	6.33765E-05	RGS2
2742224	2.12943883	6.8289E-05	SPRY1
3325634	-2.27418387	7.17985E-05	WT1-AS
2443370	2.47225355	7.45182E-05	F5
2599303	2.32623315	8.47001E-05	CXCR1
3945515	2.45632484	8.97242E-05	APOBEC3A_B
3839910	1.93891461	0.000103268	FPR2
2701109	-2.42643767	0.000126532	IGSF10
3671935	2.22213397	0.000127997	CRISPLD2
2911372	-2.04188609	0.00017057	BAG2
2778273	-2.07583683	0.000175172	HPGDS
2648677	2.4822021	0.000179368	MME
4013549	-2.80173916	0.000181129	ITM2A
2820925	-2.35528633	0.000194776	RHOBTB3
3229338	2.47801094	0.000204579	FCN1
3982612	-2.5262659	0.000215902	GPR174
3373811	2.61442156	0.00022098	PRG2
3223551	1.75027641	0.000221998	MEGF9
3317071	2.0095036	0.00023437	LSP1
3437500	2.47500186	0.000259637	GLT1D1
2477438	1.86955437	0.000275862	QPCT
3466206	1.7311667	0.000292135	TMCC3
3523855	-1.91008549	0.000316632	TEX30
3905875	2.10919725	0.000317371	MAFB
3148463	-2.28965408	0.000332624	ANGPT1
3617574	-2.07672921	0.000337581	GOLGA8B
3244622	1.76734706	0.00037999	ALOX5
3667858	2.30869227	0.00040735	HP
3974019	2.57015805	0.000409246	TSPAN7
2934308	1.79486904	0.000419692	IGF2R
2435981	2.66371347	0.000455656	S100A12
3499585	-2.22056419	0.000465259	BIVM
3556816	2.91960425	0.000468801	SLC7A7
2398820	1.95399704	0.000479217	PADI2
2799030	1.78532499	0.000503966	SLC6A19

3870733	2.01520871	0.000507894	LILRB2
3870361	1.75995943	0.000510495	NLRP12
2322848	1.92579136	0.000511243	PADI4
3291151	-1.76502551	0.000513671	RHOBTB1
3518418	1.99115471	0.000544363	KCTD12
2363689	2.24034137	0.000633941	FCGR2A
3841357	1.78340714	0.000640998	LILRA2
2947095	1.91528351	0.000643552	HIST1H3J
3633699	-2.90642504	0.000661361	NRG4
3569754	1.80580688	0.000717663	ZFP36L1
3071459	1.49764082	0.00073427	LRRC4
3417842	2.54633103	0.000740925	LRP1
3444043	1.86533231	0.000750308	OLR1
3547375	1.76625647	0.000836981	GPR65
3651478	-2.08666464	0.00087774	ACSM3
3762473	1.65301205	0.000894087	TOB1
3351166	2.21022912	0.000894283	IL10RA
3535628	1.53285161	0.000896742	GNG2
2974610	2.26344998	0.00090043	VNN3
3852832	1.78221045	0.000943974	EMR3
3375091	1.73758512	0.000977874	SLC15A3
2731542	2.10133882	0.001006461	AREG
3238528	-2.77983317	0.001011134	SPAG6
3257204	-1.97456697	0.001038349	IFIT3
3869158	1.4327185	0.001063948	SIGLEC5
2411173	1.73979943	0.001077661	PDZK1IP1
3046556	-2.60753496	0.001105335	TARP
3617458	-1.82234832	0.001106002	GOLGA8B
3927226	2.88579846	0.001110218	APP
2979871	1.40670547	0.001134285	SYNE1
3894699	1.47818122	0.00114702	SIRPD
3126504	1.47755561	0.001179152	CSGALNACT1
2434031	-1.74202906	0.001183566	HIST2H2BF
2945882	-1.6100633	0.001192223	CMAHP
3031624	1.87498921	0.001198881	TMEM176A
3445723	2.17248305	0.001209459	ART4
2662331	1.48451392	0.001216325	CAMK1
3661684	-2.26012348	0.001229528	MMP2
3656990	1.74306673	0.001244949	ITGAM
2324084	2.57993304	0.001290272	CDA
3527662	2.86944442	0.001305092	RNASE6
2548699	1.9893599	0.001330224	CYP1B1
2578028	1.43594416	0.001348337	CXCR4
3962401	1.55543007	0.001421148	NFAM1
2878809	1.42080437	0.001427845	ARAP3
2600068	2.14259981	0.0014369	TUBA4A
2761842	2.13532831	0.001476369	PROM1
4047185	1.65576549	0.001479031	CCRL2
3821301	-1.64825266	0.001481863	ZNF627
2734421	1.83736338	0.001498537	ARHGAP24
3149528	2.16060033	0.001500697	TRPS1
2364677	1.45636696	0.001507801	PBX1
3222144	1.56790565	0.001571731	TNFSF8
2363562	2.1368425	0.001607274	FCER1G
3089816	2.05414075	0.001619739	TNFRSF10C
2910236	-1.46022646	0.00164126	EFHC1
2357193	-2.03535341	0.001676699	
3577612	1.84201389	0.001680555	SERPINA1
3824874	1.62912461	0.001689043	IFI30
3371003	1.39525284	0.00174792	TP53I11
3346548	-1.53157721	0.001771052	BIRC3
3368304	-1.45162754	0.001776674	WT1
3288013	-1.4752359	0.001789496	AGAP6
3477967	1.42995209	0.001809358	
2878273	1.88715934	0.001821577	HBEGF
2608309	1.58319727	0.00182332	LRRN1
2867836	1.32430511	0.001829401	GLRX
3863669	2.22835824	0.001868719	CEACAM1
2975014	2.85317636	0.001930704	SGK1
3703885	1.34213005	0.001984855	SLC7A5
3259253	1.89774158	0.002196242	ENTPD1
3869078	-1.86913346	0.00222354	SIGLEC12
3544562	1.41393375	0.002226136	JDP2
3857171	-1.82511531	0.002313165	ZNF675
3388751	2.47288175	0.002352021	MMP8
3076076	1.46823552	0.002359628	SLC37A3
2440943	1.55053303	0.00237307	FCGR3A
3890597	1.568824	0.002412871	RBM38
3742783	1.58451225	0.002442043	NLRP1
3043264	1.98960378	0.002523458	JAZF1
2663396	1.43072802	0.002563936	IQSEC1
3732793	1.34802732	0.002593407	ARSG
3595594	2.33853155	0.002600141	AQP9
3727510	-1.48437894	0.002726256	STXBP4
2694314	-1.55716989	0.002792906	GATA2
3012019	-1.52510096	0.002864033	CLDN12
3223967	2.28382851	0.00287453	GGTA1P
2792166	2.31649926	0.00287815	MARCH1
3677516	1.253848	0.002896231	MEFV
3863189	1.93480955	0.002912414	CEACAM4
2802696	2.41165324	0.002917041	FAM105A
3854954	1.4708896	0.002953999	LRRC25
3865853	2.30772236	0.002998399	PGLYRP1
3521484	-1.84326816	0.00300265	UGGT2
3955185	-1.37906819	0.003015102	GGT5
3329983	2.13709388	0.003158041	OR4B1
3870758	1.90204587	0.003194656	LILRA5
2731513	-2.5548448	0.003303404	EREG
3773340	1.2981072	0.003312969	SGSH
3484436	1.30006353	0.003350399	EEF1DP3
3325503	-2.2690203	0.00336391	RCN1
3589458	2.19531684	0.003400219	THBS1
3525234	1.69780916	0.003402266	IRS2
2709606	1.33836115	0.003446725	RPL39L

2940202	1.99793361	0.003496342	F13A1
3926138	-1.31781348	0.00351901	C21orf91
3789947	1.93462192	0.003612678	NEDD4L
3795942	-1.29629881	0.003660447	YES1
3107242	-1.34458818	0.003674442	TMEM67
3456700	1.50521029	0.003755663	ZNF385A
3869030	1.1772779	0.003781329	SIGLEC10
2697490	-2.2401902	0.003787897	CEP70
3756591	1.20986121	0.003831654	KRT23
2362333	1.37530926	0.003872907	MNDA
2672140	2.39490665	0.003886799	LTF
3193339	1.23159877	0.003911212	RXRA
2547454	1.62143879	0.003915946	NLRC4
2403215	1.4612664	0.00392983	FGR
3908358	1.3270342	0.00394437	SULF2
3186966	1.23557393	0.003945887	TLR4
4016045	1.36140569	0.003957881	TCEAL6
2943434	1.62902419	0.003987808	ATXN1
2766788	2.12883521	0.00399217	RBM47
3841862	2.44892413	0.004027686	FCAR
2702724	-1.54283621	0.004036317	LXN
3042919	-2.21128676	0.004057991	HOXA9
3816664	-1.3797784	0.004062578	ZNF555
3108489	-1.98173023	0.004071905	LAPTM4B
3844744	-1.96723121	0.00410453	PRSS57
2663504	1.34510019	0.004119315	LOC100128644
2374746	2.01972516	0.004148089	NAV1
2731381	1.79394711	0.004175376	CXCL1
2791419	1.93505205	0.004236836	FAM198B
2947889	1.67231718	0.004334854	GABBR1
2869096	1.56805921	0.004399984	SLCO4C1
3020222	1.30368512	0.004412819	
4011008	1.91147509	0.004419609	VSIG4
2874920	1.49230139	0.004424256	ACSL6
3839818	-1.41475066	0.004460544	ZNF175
3489138	-2.25087217	0.00451204	CYSLTR2
3761725	1.98225339	0.004577099	PHOSPHO1
3835372	-1.31298322	0.00463196	ZNF223
3005444	1.42413362	0.004753081	TPST1
2400518	1.40514061	0.004753316	ECE1
2377332	1.77551999	0.004758751	CR1
3839619	1.61794753	0.004821997	SIGLEC9
2580943	-1.17526377	0.004988818	RBM43
3820612	1.27608067	0.0050244	SLC44A2
3791935	2.27276526	0.005045313	SERPINB2
3444009	2.05337974	0.005078051	CLEC7A
3409127	-1.28371155	0.005128241	ARNTL2
2451309	-1.32143841	0.005158164	KDM5B
3260338	-1.80702119	0.005230026	NKX2-3
2359664	1.86198649	0.005250966	S100A9
3132016	1.29156104	0.00534143	FGFR1
3528895	1.25317057	0.005349833	LRP10
2731496	-1.84999271	0.005361321	EPGN
2886679	1.14886591	0.005374336	KCNMB1
2595042	-1.39843981	0.005401996	ALS2
2796484	-1.2601922	0.0054443	CASP3
3134013	1.87120004	0.005651526	CEBPD
3838624	1.5742417	0.005653022	FCGRT
2781693	-1.30674775	0.005677251	CASP6
2604390	1.19269227	0.005689654	ARL4C
3469844	-1.1474868	0.005713206	MTERF2
3025500	1.5521635	0.005791068	BPGM
3125001	-1.28495478	0.005829117	LONRF1
2620842	2.96748923	0.005845243	CCR2
3831588	-1.36432157	0.005892397	ZNF345
3778504	2.3756012	0.005911288	RAB31
3150579	-2.4600065	0.005933181	ENPP2
3837269	1.11265289	0.00610999	C5AR2
3442706	2.10830633	0.006156474	CD163
2361342	1.32233696	0.006365084	SEMA4A
4023242	-4.52720918	0.006368953	CT45A5
2452615	-1.33043669	0.006402252	SLC45A3
3484060	1.65416671	0.006411491	ALOX5AP
3827218	1.99366448	0.006429471	RPSA
3205488	-1.10757813	0.006459598	ZBTB5
4045643	2.78905967	0.006517375	S100A16
3684486	2.04316728	0.00672644	IGSF6
3393479	1.23178121	0.006751387	FXYD6
3941793	1.39441989	0.00683021	KREMEN1
2336456	1.09953243	0.006845301	FAM159A
2787703	1.18128019	0.00690329	
2975741	-1.44739245	0.006905592	MAP7
3062082	1.16192308	0.006932673	PDK4
3382861	1.33829987	0.006973567	PAK1
3826079	-1.66432838	0.006990516	ZNF93
2709778	2.11375481	0.00706569	BCL6
3389077	-1.63818061	0.007191715	PDGFD
2464909	-1.15483127	0.007210323	SMYD3
2672096	1.67055023	0.007234509	CCR1
3638607	1.89568149	0.007248573	ANPEP
2899194	-1.14226522	0.007309173	HIST1H2BE
2899176	-1.40187394	0.007310843	HIST1H2BD
2946194	1.44979304	0.007527293	HIST1H1A
3936009	1.53649853	0.007566849	CECR7
2325192	-1.09160666	0.007572707	RPL11
2327283	1.2083551	0.007705244	THEMIS2
3902489	1.31621244	0.00780664	BCL2L1
3901055	1.9074993	0.007823594	CD93
3469687	1.60666615	0.007834385	CKAP4
3354443	1.61007596	0.007893578	SLC37A2
2946714	-1.23304871	0.007952607	HIST1H2BK
3843180	-1.05681873	0.008129113	ZNF304
3373795	1.184353	0.008166706	PRG3
2941784	1.24822978	0.008215245	NEDD9

3480411	-1.19681183	0.008266427	IFT88
2422035	-1.24123993	0.008301941	GBP5
3876990	-2.53536914	0.008309445	SPTLC3
3190420	-1.32406703	0.008324739	CERCAM
2888485	1.37017774	0.008326868	HK3
2376376	1.88861556	0.008374177	TMCC2
3317352	1.03619806	0.008524842	KCNQ1
2565935	-2.02268118	0.008587462	ANKRD36B
3452818	1.02574065	0.008604034	VDR
3823583	-1.88054425	0.008614124	HSH2D
3167731	-1.32916606	0.008620006	UNC13B
3927903	-1.27125956	0.008635984	N6AMT1
2724235	-1.20597173	0.008655456	WDR19
3831260	-1.55490206	0.00866769	ZNF146
2320411	1.33861867	0.008693291	AGTRAP
3000984	2.07673788	0.00884957	ABCA13
2891341	1.16122701	0.008902863	IRF4
3327166	-1.05312015	0.008968859	C11orf74
2642543	1.22401598	0.00897012	NUDT16P1
3523881	-1.05003546	0.009062907	KDELC1
3833893	-1.15850887	0.009066758	CYP2B7P
3453732	-1.907542	0.009077763	TUBA1B
3142217	1.51837261	0.009090641	PAG1
2736259	-1.26419231	0.009119447	SMARCAD1
3770305	1.26738159	0.009119959	CD300C
3139580	1.32546589	0.009212322	SLCO5A1
3768474	1.22513747	0.009220396	WIPI1
3722917	1.15503842	0.009257981	GRN
3485674	-3.0962391	0.009285424	CCNA1
2690900	-1.31819548	0.009294769	CD80
2448073	1.08095318	0.009335336	IVNS1ABP
3645601	1.09279694	0.009363327	MMP25
2750594	-1.4055351	0.009442776	MSMO1
3456805	-3.75477311	0.009554207	GTSF1
3015519	1.7079581	0.009644543	PILRA
2818517	3.70464486	0.009713977	VCAN
2483016	-1.2782693	0.009787008	CCDC104
2461473	-1.18322522	0.009834652	TARBP1
3702547	1.24059051	0.009878953	COTL1
2319661	1.03614909	0.01014385	KIF1B
2548617	1.08936174	0.010199483	CDC42EP3
2674047	1.14808426	0.010377472	LAMB2
3627248	1.07328843	0.010412322	ANXA2
3015865	1.05587458	0.010478524	SLC12A9
2946845	-1.08113973	0.010583388	ZNF184
3598662	1.1848192	0.010604314	MAP2K1
2447414	1.92840728	0.010702222	NCF2
3250602	-1.44969182	0.010912235	H2AFY2
2899102	2.57903872	0.010951555	
3040454	-1.11413203	0.010988603	TWISTNB
3944404	-0.99894607	0.010994949	APOL1
3105430	-1.02314502	0.011130024	LRRCC1
3053380	-1.6200368	0.011160178	ERV3-1
3841621	2.03944459	0.0112064	LILRB4
3802254	-1.28122843	0.011259559	KCTD1
3268588	-1.12333624	0.011262127	ACADSB
2777564	1.05087661	0.011432802	FAM13A
3178545	1.20235444	0.011461395	C9orf47
2484422	-1.15059722	0.011462206	PEX13
3408573	-1.24483545	0.011475284	LYRM5
3165780	-1.14370363	0.011488905	IFT74
2635741	-1.52246601	0.011563614	CD96
2958670	-1.17026223	0.011614291	RAB23
3153428	1.27906325	0.011653723	ASAP1
2542651	-1.43990197	0.011707242	WDR35
2468622	1.82576877	0.01179173	ID2
3991650	-1.17705325	0.011809125	PHF6
3657367	-1.04675354	0.011891822	ZNF267
2351854	1.10886015	0.01195732	C1orf162
2401448	1.4115602	0.011978166	E2F2
2698565	-1.11976013	0.012031117	TFDP2
3815243	-1.11692899	0.012107015	CFD
2547716	-1.37996934	0.012111422	FAM98A
2777639	1.68460244	0.012125196	GPRIN3
3770345	1.5975123	0.012139009	CD300E
3634811	2.41470033	0.012149847	CTSH
2862696	1.02405461	0.012194422	ENC1
3860450	-1.24375392	0.012197049	ZNF566
2512930	1.00150755	0.012529265	GCA
3060332	1.43040325	0.012530767	STEAP4
3920850	1.01683319	0.012581327	KCNJ15
3868998	1.53053831	0.012767223	NKG7
3864551	1.26390634	0.012872681	PLAUR
3982534	-1.15223389	0.012898376	LPAR4
2476510	-1.35938326	0.01294462	LTBP1
3746574	1.48041452	0.012972636	PMP22
3816699	-1.22141584	0.012982088	ZNF57
4039752	1.37469029	0.013027614	DOC2B
2533670	1.08731601	0.013122399	AGAP1
2913277	-1.13933591	0.01314702	KCNQ5
2925841	1.98852505	0.013168796	ARG1
3683018	-1.19690765	0.013228792	RPS15A
2372211	-1.16342167	0.013242981	PLA2G4A
3512948	1.09541811	0.013261082	KIAA0226L
2898562	-0.96284379	0.013296581	ACOT13
3841545	1.46092307	0.013310584	LILRA1
3360456	-4.01632791	0.013333218	OR51B5
3450655	-1.12838521	0.013392088	CPNE8
3651588	-1.07428751	0.013413614	LYRM1
2460817	1.16301097	0.013426786	SIPA1L2
2353283	-1.8518432	0.013463649	VANGL1
3571727	-0.95916415	0.013500502	ALDH6A1
2704267	-1.01041152	0.013588979	GOLIM4
2803329	1.2388837	0.013602867	BASP1

3474372	0.9693703	0.013612292	PXN
4036196	1.06930164	0.013642757	ERVH-6
2401994	0.97641254	0.013656141	RUNX3
2499053	-1.04884105	0.013661561	LIMS1
3742627	2.23936461	0.013916334	SCIMP
3973839	1.5747148	0.014019141	CYBB
2580955	-1.1150047	0.014042721	NMI
2377094	1.7176729	0.014071043	PFKFB2
2650393	-0.96224474	0.014158193	PPM1L
2553970	-1.14542652	0.014223633	PNPT1
3849688	-1.16834825	0.014431133	ZNF266
3628832	1.05974372	0.014513701	DAPK2
3786868	1.86141681	0.014543767	SLC14A1
3443868	-1.81464096	0.014554605	CD69
3622386	-1.57527594	0.014683058	GATM
3636391	1.09115787	0.014684966	HOMER2
3768412	1.25213224	0.014695165	SLC16A6
2451593	1.94399345	0.014808775	CHI3L1
2435383	1.53651783	0.014842137	S100A10
3728588	1.80184291	0.014900661	EPX
3662774	-1.18841644	0.0149132	GPR114
2980258	1.24401785	0.01500055	MTRF1L
3066436	-1.28386022	0.015022987	PUS7
2905327	1.23644319	0.015051665	FGD2
2549565	1.95596818	0.015172588	SLC8A1
3185498	1.04347443	0.015397804	SLC31A2
2948547	0.9357565	0.015482674	NRM
2805635	-1.63058756	0.015508773	NPR3
2741083	-0.98504458	0.015518254	METTL14
3558145	0.98502053	0.015589833	CIDEB
2411575	-1.3293736	0.015656234	SPATA6
2980449	1.04121582	0.015832117	IPCEF1
3299255	-1.07617492	0.015867383	ATAD1
3730806	1.0462393	0.015988498	MAP3K3
3774906	1.29632132	0.016114379	SECTM1
2366753	-0.92285335	0.016160905	GORAB
3203855	1.16410053	0.016246901	DCAF12
3584443	1.04022874	0.01627916	SNRPN
3695867	1.00695718	0.016289419	RANBP10
3849267	-1.10252376	0.016295263	ZNF558
2362157	1.60016168	0.016338203	CD1D
2381309	1.39803597	0.016360415	MARC1
2922840	-1.09341133	0.016409655	KPNA5
2768396	-1.11237863	0.016492364	TEC
3737874	-1.20333692	0.016513107	BAHCC1
2727587	-1.6375491	0.016535156	KIT
3551120	1.29220602	0.016604145	ERVH-4
2378180	-1.19342348	0.016668399	DIEXF
3203636	-1.16739496	0.016693256	SUGT1P1
2476411	-1.36606111	0.016721784	TTC27
3602767	1.01664368	0.016747584	PSTPIP1
2345286	1.12351778	0.016758311	LMO4
3555675	1.17008499	0.016777805	RNASE1
3749010	1.1341425	0.016839006	ULK2
2853388	-1.07026172	0.016966992	NADK2
2882098	1.65197558	0.017006441	SPARC
4018080	1.18279545	0.017062737	CHRDL1
3504213	1.96075739	0.017107552	GJB6
3777470	1.970293	0.017127175	PTPRM
3015911	0.90806717	0.017128954	TRIP6
3431620	-1.14736021	0.017203516	TCTN1
3310725	-1.08540449	0.017247863	C10orf88
3011977	-1.10513062	0.017266688	GTPBP10
3858659	-0.99610036	0.017272909	ANKRD27
3442785	1.02203322	0.017303969	CLEC4C
3745525	-1.08307921	0.017344506	TMEM220
3474418	0.9003268	0.017361291	PXN
2421000	-1.53477023	0.017402136	COL24A1
3243078	-0.98296993	0.017461414	ZNF33A
3171865	-0.99507099	0.017493316	LOC100132167
2518583	-1.10149171	0.017597165	DNAJC10
2625907	1.1063157	0.017670095	FLNB
2642720	1.03303727	0.017850049	ACPP
3274758	-0.95193922	0.01785488	AKR1C2
2732844	1.47512012	0.017871642	ANXA3
2924619	-1.24036296	0.017987467	TRMT11
2362201	2.49760965	0.018115988	CD1C
3835035	1.10645884	0.018225088	CD177
3831917	-1.16161318	0.018279374	ZNF570
2389718	-1.17402569	0.018355178	CNST
3061456	-1.31826657	0.018357423	SAMD9L
3989826	-1.1911483	0.018480923	SH2D1A
2984655	1.02981253	0.018569185	RPS6KA2
2324416	0.98021745	0.018672018	ALPL
2879028	0.90904197	0.018721902	GNPDA1
2519756	-0.95474423	0.018732757	WDR75
3586834	-1.05884846	0.018809943	FAN1
3160175	1.30385086	0.018868996	VLDLR
3951768	1.55607526	0.018890765	CECR1
3967689	1.32961057	0.018911378	STS
3326950	1.54663013	0.019026177	LDLRAD3
3795680	-0.92203523	0.01903778	THOC1
2849469	1.21697914	0.019139201	ANKH
2939886	0.93843041	0.01925373	LYRM4
2478017	-0.96535657	0.019298907	MORN2
2421121	-1.08212567	0.019316213	ODF2L
2874686	-0.88961968	0.019372132	HINT1
2703133	-1.15486791	0.01948185	IFT80
2452405	1.042734	0.019493397	NUAK2
3731826	1.39808615	0.019586229	PRKCA
2598261	2.14683004	0.019688136	FN1
3995392	1.07791337	0.019692436	ZNF185
3333309	0.89182124	0.019869462	BEST1
3568080	-1.44396094	0.020002338	WDR89

3089102	1.62262564	0.020073275	DMTN
2470838	-1.9500408	0.020178983	MYCN
3826041	-1.40247578	0.020207557	ZNF253
2421843	-0.97527694	0.020312082	GBP3
2591837	-1.00399344	0.02031972	SLC40A1
3125571	1.99642665	0.020458169	MSR1
2900372	-0.93131029	0.020513478	ZSCAN9
2598606	1.02710232	0.02062656	PECR
2784027	2.11158911	0.02065847	ANXA5
3267036	1.06111558	0.020660719	GRK5
2527655	0.9003592	0.020661702	GPBAR1
2732611	-0.96556956	0.020662596	MRPL1
3415229	1.09912203	0.020671809	NR4A1
3683845	-1.11114527	0.020690088	DCUN1D3
3160735	-0.89367305	0.020707956	CDC37L1
2821347	-1.04272018	0.020717782	ERAP2
2792800	-1.44821135	0.020722059	DDX60
3666601	0.99803804	0.020753019	SNTB2
2871717	-0.92733024	0.020796913	CCDC112
3101893	-0.87757418	0.020823923	CSPP1
2591421	-0.88796608	0.020867249	TFPI
2420958	-1.35067682	0.020970136	ZNHIT6
3354227	1.00619788	0.02097092	NRGN
3840142	-1.1650092	0.021059492	ZNF480
2947100	-1.56455503	0.021223532	HIST1H2AM
3131819	-1.40392605	0.02123558	STAR
3898126	-1.08690805	0.021312199	TASP1
3442854	1.22619149	0.021518711	SLC2A3
3749332	-1.22315375	0.021583102	
3707095	1.18500874	0.021593893	ARRB2
3741456	-1.10988915	0.021691565	TRPV1
2977471	-1.05108697	0.022002554	ADAT2
3256074	-2.3991126	0.022096476	BMPR1A
2855285	-2.32557613	0.022123239	SEPP1
2766492	1.22877638	0.022124748	SMIM14
2895159	-0.92674516	0.022167584	HIVEP1
3827427	-0.92663654	0.022183029	ZNF254
3843742	0.92266737	0.022192529	ZNF135
3860824	-0.92971294	0.02234449	ZNF569
3463727	1.49603599	0.022425988	LIN7A
2775994	1.08916768	0.022527388	HPSE
3951719	0.91880017	0.022529542	CECR6
3103523	1.30980459	0.022564182	LY96
3520989	-1.09615212	0.022636344	TGDS
3790361	1.87325418	0.022784823	ZNF532
3959388	-1.08420796	0.022846524	APOL4
2954506	0.91161839	0.02289637	CRIP3
2949311	-0.86481008	0.023100309	DDAH2
3389878	-0.90853442	0.023186051	ALKBH8
2992814	0.95513749	0.023201397	GPNMB
3148582	-0.96784352	0.023252541	EIF3E
2948239	1.20250962	0.02330499	TRIM10
2848265	-1.09712746	0.023367301	CMBL
3181976	0.97391627	0.023473502	NR4A3
2515276	-0.95111579	0.023473852	DYNC1I2
2623568	0.99777419	0.023678846	PPM1M
3667890	1.32265158	0.023679811	HPR
2673312	0.87554221	0.023746804	PFKFB4
3821159	0.843789	0.02391408	LPPR2
3725602	0.90119747	0.023927547	ABI3
2705748	0.95609037	0.023937944	NCEH1
2392584	0.96883681	0.023944864	TNFRSF14
2927967	-0.90756616	0.023967181	ABRACL
3841574	1.39929887	0.023981322	LILRB1
2894573	1.0617641	0.024094203	GCNT2
3981735	-1.50784161	0.024101596	JPX
2900059	1.96470649	0.024142966	HIST1H2BM
3713951	0.91692833	0.024192651	SLC47A1
2720732	-1.11423454	0.024216257	PACRGL
3874636	0.99138847	0.024223031	SMOX
2389789	-0.8766056	0.024252403	SCCPDH
2905025	0.91403352	0.024270719	PNPLA1
2883440	0.86756819	0.024330432	ADAM19
2379399	-1.00603722	0.024435823	RPS6KC1
2633460	-0.89711523	0.02443951	CMSS1
3352948	1.01759845	0.024439949	SORL1
3439356	-1.11666189	0.024661482	ZNF140
2743085	-0.88220925	0.024680144	LARP1B
2527580	1.06005295	0.024914316	CXCR2
2452691	0.94575913	0.025075419	SLC41A1
3224591	-0.97414403	0.02515497	STRBP
3622934	1.82712418	0.025167894	MYEF2
2384401	1.01931911	0.025183166	RHOU
2474977	0.9119618	0.02521334	FOSL2
3746845	-0.99467955	0.025232139	TRIM16L
2929127	1.53430836	0.025235769	STX11
3574121	-1.71803399	0.025318779	STON2
3869650	-1.02299419	0.025325612	ZNF83
3130757	-1.18168887	0.02536806	FUT10
2469910	-1.01237799	0.025385681	LPIN1
2380055	-1.06726678	0.025450926	KCTD3
3040518	-2.4321332	0.025529258	MACC1
3308489	1.62539473	0.025639481	KIAA1598
4013224	-0.93936085	0.025701627	ATRX
3625761	-0.95694247	0.025880952	MNS1
2443235	-1.06201789	0.02591513	NME7
3894098	-0.8775181	0.026008301	C20orf96
2595560	1.22973224	0.026024704	RAPH1
3340665	0.99111485	0.026066417	DGAT2
2642325	-0.8967407	0.026208757	ATP2C1
3438061	0.90891074	0.026281374	GPR133
3746675	-0.86676701	0.026436376	CDRT4
2434124	-1.10287549	0.026474444	HIST2H2BE
3061319	-0.95964747	0.026654467	CDK6

3901041	0.89163006	0.026687831	THBD
2486178	-2.36180388	0.026693606	MEIS1
3403595	1.45915841	0.026775641	CLEC4A
2390180	0.97703517	0.026845693	OR2W3
2770193	-1.06322963	0.026956521	AASDH
3076868	1.68096616	0.026962902	CLEC5A
2595443	-0.86874385	0.02699792	WDR12
3655587	-0.90511766	0.027066303	QPRT
3985523	-1.44056838	0.027074763	WBP5
2451463	1.05287976	0.027108559	ADIPOR1
2365958	-1.04715433	0.027114611	MPZL1
2666478	-0.87646201	0.027133422	TOP2B
3948590	0.88245257	0.027201209	RIBC2
3129065	-1.70114631	0.02721907	CLU
3543756	-0.97468964	0.027286422	DNAL1
2435989	1.2553404	0.027311176	S100A8
2719617	1.07386805	0.027351965	BST1
3613338	-0.97629842	0.027385841	NIPA1
3543714	-0.94127342	0.027398353	ACOT4
2391687	1.67195445	0.027406694	NADK
3022814	-1.37758769	0.027504235	HILPDA
3973768	-0.89611426	0.027529071	LANCL3
3294576	-1.08798066	0.027631582	USP54
3771037	0.92218486	0.027686649	WBP2
2789266	-0.86645489	0.027744146	LRBA
3053229	-1.12941746	0.027745128	ZNF680
3791996	1.30708735	0.027814383	SERPINB8
3783723	-0.95980195	0.027938374	RNF125
4021508	-0.87257565	0.027946994	ZNF280C
3426502	1.00592667	0.027993601	PLXNC1
3666409	1.39970769	0.02806507	CDH1
2435005	1.90869611	0.028197694	SELENBP1
3645947	-1.13805965	0.028268858	CLUAP1
3828162	-0.88527431	0.028277616	URI1
3822122	-0.94338264	0.028284226	NFIX
3672059	0.86940501	0.028421886	KIAA0513
2994558	0.83122511	0.028430732	CREB5
2440385	-1.31680008	0.028448548	CD244
3419641	1.00919894	0.028523697	SRGAP1
2735027	0.8760038	0.028585427	SPP1
3493448	-0.9784805	0.02861544	PIBF1
2680571	0.84685115	0.028696122	LOC100508226
2731757	-0.98926474	0.028722654	THAP6
3244539	-1.10825478	0.028947023	ZNF22
2648098	-1.93693208	0.029055701	SUCNR1
3896257	1.28099033	0.029159073	PROKR2
2363248	1.06801107	0.029159378	LY9
3029475	0.81430687	0.029222681	OR6B1
2805695	-1.0415327	0.029327836	NPR3
3079172	1.14174044	0.029363613	TMEM176B
3168385	0.97341284	0.029404149	GLIPR2
2344393	-1.1447089	0.029505798	PRKACB
2966298	-1.04652802	0.029590513	USP45
3009198	0.88937398	0.029618539	RHBDD2
2439508	1.19575869	0.029653783	OR6N2
3838665	0.86202782	0.029763523	RCN3
3214749	-1.02021665	0.029792343	NOL8
3845681	0.81472016	0.029834428	MOB3A
3403841	-0.94953844	0.030031008	RIMKLB
2498977	-0.86358005	0.030192375	GCC2
3421177	-0.8340602	0.030272464	NUP107
2896545	1.34803496	0.03030039	GMPR
2440476	-0.81841414	0.030383386	TSTD1
2694123	-0.90814102	0.030411874	RUVBL1
2990043	-1.16173692	0.03043915	PHF14
3677612	-0.84617699	0.03049652	ZNF597
3142519	-0.84996859	0.030512426	ZFAND1
2427688	-1.2156752	0.030579005	LRIF1
3219215	2.04654236	0.030661636	KLF4
2841699	1.27030243	0.030661691	CPEB4
3503164	-0.84553158	0.030699112	CDC16
3840372	-1.17330015	0.030713374	ZNF701
3744150	1.05584313	0.03072916	PER1
2331679	0.91533853	0.030807042	MFSD2A
3839642	0.87468893	0.030823977	SIGLEC7
2493858	1.18181585	0.030948296	MAL
2597273	-0.98462045	0.031000963	KANSL1L
3356175	0.95029497	0.031084428	ST14
3014229	1.04317601	0.031251775	BRI3
3869847	-0.83639508	0.031255926	ZNF468
3439305	-1.18661086	0.031311958	ZNF84
3144235	0.85566403	0.031543902	TMEM55A
3156307	1.97069413	0.031561818	PTK2
3389529	-1.45809902	0.031574614	MSANTD4
3406589	-0.82919706	0.031707361	MGST1
2767295	0.94727613	0.031732668	BEND4
2517013	-0.95855353	0.031815564	MTX2
2558511	-1.1255871	0.031992868	TIA1
2902609	0.8304991	0.032002855	C6orf25
2764054	-0.84058447	0.032191405	SEPSECS
2419046	-0.89710165	0.032255626	ZZZ3
3603932	-0.90661975	0.032372004	FAH
2817793	0.91007391	0.032558249	FAM151B
2461999	0.85908568	0.032587488	LYST
3632492	0.87678143	0.032691215	NPTN
3456955	-1.35448999	0.032703156	TESPA1
3583541	0.79039261	0.032716502	GOLGA6L1
4022106	0.8850145	0.032772555	MBNL3
3264621	1.02901809	0.032783921	TCF7L2
3535186	-1.21900971	0.033022177	ATL1
2777044	-0.82917863	0.033049141	HSD17B13
2900750	0.82709483	0.033066978	OR2J3
3860596	-0.89680027	0.033195645	ZNF461
3509677	-1.13059032	0.033347453	CCDC169

2953570	1.61571276	0.033381543	TREM1
3576812	-1.18271012	0.033395352	TRIP11
3118818	-0.95799193	0.033397332	PTP4A3
3677913	1.22060028	0.033439307	ADCY9
3752437	-0.96639021	0.033479155	UTP6
2699623	0.85851947	0.033480179	PLSCR4
2766219	0.95078738	0.03362764	TLR1
3665049	0.80640718	0.03367823	CES4A
2924253	0.80798387	0.033901773	RNF217
2660029	-1.06106409	0.033936438	LMLN
3826803	0.80619508	0.033978328	ZNF729
3872733	-0.7907063	0.033979186	ZNF329
3849044	0.87173482	0.034012098	MYO1F
2780522	-0.78103502	0.034028898	PPA2
2897172	1.06438613	0.034040147	RNF144B
2809423	-0.83144796	0.034041808	NDUFS4
2734047	1.2486712	0.034077506	AGPAT9
3843233	-1.10652742	0.034308448	ZNF17
3464622	-1.06359156	0.034395302	CEP290
3534128	-0.99177022	0.034538908	FAM179B
3254521	0.82767725	0.034549444	TSPAN14
3039731	-0.78522444	0.03456524	ANKMY2
2808180	-1.08264236	0.03461451	LOC153684
3527340	1.00911036	0.034759342	OR4L1
3175274	0.85771967	0.034851238	PCSK5
3257246	-1.38849215	0.034889509	IFIT1
2808290	-0.94587524	0.034946823	ZNF131
3611744	0.87800369	0.035036358	LRRK1
3305017	-0.96222576	0.035085515	OBFC1
2998333	-1.3256573	0.035112569	YAE1D1
2595388	-0.9344568	0.035136766	ICA1L
2854327	0.91930981	0.035208176	FYB
2881187	1.58947564	0.035280656	CSF1R
3466499	-2.19591649	0.03543288	USP44
3299469	0.82511059	0.035447302	ANKRD22
3772719	-1.66300207	0.035507838	LGALS3BP
2988459	-0.82055553	0.035523254	RBAK
3320717	1.07633476	0.035585719	MICAL2
3750723	0.8147362	0.035617983	UNC119
3153235	-0.86192711	0.035658182	CCDC26
3536706	1.44769874	0.035720744	LGALS3
3541073	-0.78443805	0.035733003	MPP5
2351004	1.16706655	0.035733989	GSTM5
3686635	1.19083695	0.035865071	APOBR
2551905	0.97625469	0.035895385	C2orf61
2944025	-1.08692207	0.035919659	TPMT
4009849	1.74956447	0.036060936	ALAS2
3934729	1.04598694	0.036095045	ITGB2
2692909	0.8401134	0.036148205	HEG1
3638699	0.83245775	0.036151903	ARPIN
2389062	-0.96371821	0.036208795	COX20
3883941	-0.93031314	0.036255132	TGIF2
2686371	-0.81673993	0.036450574	TOMM70A
3992521	-0.78446594	0.036450843	HTATSF1
2348060	-0.77824958	0.036510091	PTBP2
3449760	-0.80701816	0.036542979	DENND5B
3065963	-0.93350887	0.036564612	ORC5
3168409	0.85982917	0.036647832	CCIN
3672489	2.33268371	0.036805094	IRF8
3985615	-0.86354459	0.036822396	TCEAL4
2383524	-0.98244836	0.03687561	ZNF678
2946324	-0.83055077	0.036950622	HIST1H3D
3105467	-1.50528847	0.037194704	E2F5
2904597	0.77760631	0.037217341	PPARD
2522247	0.86514028	0.037242953	AOX1
3886765	0.83158423	0.037291103	PI3
2957314	0.88174072	0.037438377	GSTA2
3432090	0.86741281	0.03752416	ALDH2
3811000	0.9510463	0.037538961	RNF152
3894668	0.93310513	0.037612965	SIRPB2
3770290	1.01086319	0.03762703	CD300LB
3185063	2.0781328	0.037688677	UGCG
3945349	-0.92565955	0.038008361	CBY1
2916246	-0.82391403	0.038022743	SMIM8
3144740	-1.13161481	0.038234822	FP6628
3196691	-0.88613363	0.03824002	KIAA0020
2903219	1.99947721	0.038249239	HLA-DQA1
3663181	-1.0476773	0.03837343	CCDC113
3831514	-0.91665992	0.03847787	ZNF567
3111375	-0.7856705	0.038539597	EMC2
3243908	0.84973649	0.038578875	CSGALNACT2
2442493	0.82793472	0.038603637	GPA33
3190190	1.75054894	0.038982923	LCN2
2511603	0.79457697	0.039006839	GALNT5
3256590	0.93994076	0.03906833	PAPSS2
3816815	-0.7945426	0.039119547	GNA15
3771543	0.80677259	0.03913177	UBE2O
3856594	-0.80713628	0.039149107	ZNF43
2830861	1.64181541	0.03914958	EGR1
3594986	-1.01273363	0.039150132	TEX9
3854982	-0.82607785	0.039194461	ISYNA1
3871459	1.07636341	0.039332299	SHISA7
3381925	-0.78452007	0.039512621	PGM2L1
2593352	-0.87057742	0.039525288	GTF3C3
3232979	-0.75191572	0.039641987	AKR1C1
4007164	0.9281164	0.03967677	CFP
2353988	0.85623524	0.039768963	FAM46C
2438657	0.94950527	0.03985464	ARHGEF11
3201242	0.97975337	0.03988819	IFNA17
2754937	-1.5654822	0.03990755	TLR3
3714068	0.93933894	0.039993411	ALDH3A2
2921374	-0.80149093	0.040265087	RPF2
3962219	0.97734575	0.040269727	NAGA
3942179	0.80161678	0.040284213	MTMR3

2952834	-0.91152413	0.04032089	KCNK5
3028956	0.93513065	0.040656272	TAS2R39
2780143	-0.89567156	0.040661754	BDH2
2479510	-0.77332928	0.040679618	DYNC2LI1
2592356	-1.00521101	0.040796633	STAT4
2522598	0.82963845	0.040886305	NDUFB3
3435980	-0.92708685	0.041004443	TCTN2
2520429	-1.23585658	0.041005365	MYO1B
3061484	1.42523162	0.041045325	HEPACAM2
2514563	-0.97416644	0.041096086	PHOSPHO2-KLHL23
3421630	-0.78862236	0.041155444	CCT2
3359751	-0.9216354	0.041175043	ZNF195
3564027	-0.77914673	0.041248962	SAV1
3941848	-0.82560575	0.041266275	EMID1
2362230	1.92341929	0.041290366	CD1E
3970642	0.78383472	0.041351638	CDKL5
3781124	-0.90149498	0.041446475	MIB1
2326049	0.8075468	0.041493869	MAN1C1
2879927	-0.85343637	0.041537819	LARS
2400655	1.34723915	0.041544298	RAP1GAP
3826504	-0.93712701	0.041549528	ZNF431
2443575	-0.89445498	0.041570319	KIFAP3
2814642	-0.88470877	0.041684314	MCCC2
3367965	-0.80006419	0.041717067	IMMP1L
3190061	-0.77790371	0.041787738	FPGS
2832297	-2.18611583	0.041829592	PCDHB2
3250019	-0.83281599	0.041858294	DDX50
3215570	1.35866213	0.041907412	FBP1
3759006	2.0409636	0.041999918	SLC4A1
2371346	0.76584166	0.042125932	RGL1
3839346	0.94911795	0.042207652	SPIB
3489212	-0.86015249	0.042353217	FNDC3A
3466318	-0.75848384	0.042396695	NR2C1
3167110	0.84273801	0.042415606	ANXA2P2
3360396	1.06966477	0.042565336	OR51V1
3275922	-1.05231527	0.042609304	PRKCQ
2613441	0.88021126	0.042611234	KAT2B
2664452	-0.85579515	0.042740724	ANKRD28
3138204	-0.98914406	0.042790553	CYP7B1
2462511	-0.7534055	0.042827863	HEATR1
4027769	1.28872181	0.042837346	CLIC2
3908631	0.83275139	0.042898938	PREX1
3091077	1.63118011	0.042925036	DPYSL2
3087555	-0.79597077	0.042945594	VPS37A
3305313	1.33576365	0.043034202	ITPRIP
3513549	1.02348229	0.043036456	RCBTB2
2722291	-0.89731061	0.04304462	TBC1D19
3147985	-1.17633615	0.043165021	LRP12
3687452	0.74136368	0.043208311	YPEL3
3355145	-0.74785758	0.043222432	ST3GAL4
2912980	1.06563174	0.043248254	OGFRL1
3094514	-0.75127334	0.043342369	DDHD2
3322251	-0.94322225	0.043387946	NUCB2
2626258	0.83145317	0.043393672	KCTD6
3728571	0.8242661	0.043563802	OR4D1
3859622	-1.18737916	0.043574014	ZNF792
3318153	0.81522419	0.043577673	OR52I2
3148796	-1.04475986	0.043578285	NUDCD1
2606348	0.75474453	0.043631108	MGC16025
3444472	-1.3166503	0.043655074	TAS2R50
3014159	0.75455594	0.043708234	LMTK2
3975762	-0.97358716	0.043882261	KRBOX4
2947283	-0.82911999	0.043917418	ZSCAN12
2473965	0.79550561	0.044011889	SLC35F6
3008220	-0.74385868	0.04401536	CLIP2
3545634	0.7537214	0.044036868	NRXN3
3608113	1.01965762	0.044057827	IQGAP1
2609904	0.75708085	0.044114173	OGG1
3466369	1.07112906	0.044138822	FGD6
3904508	-0.87504393	0.044156282	SLA2
2358693	-1.3126402	0.044185056	MLLT11
3143112	0.84547432	0.044293721	REXO1L2P
2833286	1.12006042	0.044302309	ARHGAP26
2436526	0.76395198	0.044395795	TPM3
3131741	0.85930892	0.044409399	RAB11FIP1
2413907	-0.92959777	0.044437481	DHCR24
3621029	1.81501925	0.04444994	EPB42
3521174	-0.8535891	0.044458539	ABCC4
3849773	-1.01566049	0.044556943	ZNF121
2462160	1.18449927	0.044645242	NID1
2926802	-0.81766786	0.044669606	MYB
2664209	0.85644549	0.044703094	SH3BP5
2748830	-1.60439816	0.044863772	GUCY1A3
3301011	-0.99099041	0.044868074	NOC3L
2406783	0.79122976	0.044945681	CSF3R
2982630	0.84645271	0.044967509	LPAL2
2939814	-0.99969268	0.045102792	RPP40
3374890	1.73016218	0.045186991	TCN1
3320819	1.12613871	0.045256642	MICALCL
2497301	-0.85941197	0.045381493	TMEM182
3681705	-0.75771044	0.045429978	RRN3
3748798	0.77824499	0.045519352	MFAP4
3260586	0.89045337	0.045543392	SCD
2448971	-0.82493468	0.045573439	UCHL5
3075531	-1.15616583	0.0455991	ZC3HAV1L
2585167	0.80864926	0.045752474	LOC102724230
3130161	0.89054343	0.045816111	GSR
2910364	-0.8276676	0.045895478	TMEM14A
3602116	0.76467685	0.045933187	C15orf39
3183238	-0.95669612	0.045947164	FSD1L
3293762	0.86180225	0.045974825	PSAP
3403754	0.78809289	0.04599054	CLEC6A
2390322	0.78763344	0.046037272	
3114111	1.03549727	0.046050186	FAM83A

3345593	-0.74556671	0.046242683	CEP57
3373453	0.77230791	0.046345728	OR5M10
2476671	-1.79786926	0.046451993	RASGRP3
2776088	-0.78748182	0.046536843	FAM175A
2936564	-0.73202464	0.046654357	FGFR1OP
3382061	-0.89835714	0.046760656	XRRA1
2354082	-1.04622721	0.046877317	WDR3
2660617	-0.76597539	0.046926405	IL5RA
3226493	-0.82032698	0.047091374	TRUB2
3531355	-0.81024867	0.047135772	NUBPL
2922972	-0.76427712	0.047173092	DCBLD1
3934111	0.76885625	0.047190641	SIK1
3969115	1.73934652	0.047218093	TLR8
3470193	0.99008524	0.047230112	CMKLR1
3677795	0.72364113	0.047232197	CREBBP
2649824	-1.4505067	0.047269124	IQCJ
3386814	-0.75003362	0.047294282	TAF1D
2592268	-0.78538306	0.047372295	STAT1
2874371	0.77437914	0.04741511	FBN2
2328273	1.05102184	0.047457478	SERINC2
3416943	-0.8228336	0.047471344	GDF11
3679533	1.14047989	0.047520749	CARHSP1
2972411	0.75752716	0.047534685	TRDN
3420151	-1.01786587	0.047576134	MSRB3
3055466	0.79093869	0.047628235	CALN1
3042756	-1.36215415	0.047655648	HOXA2
2492783	0.73109448	0.04766183	THNSL2
3186123	1.35895024	0.047668522	ORM1
2360506	0.75011203	0.047671886	ZBTB7B
2880679	-0.86674633	0.047683799	SH3TC2
2671968	-0.79898697	0.047723449	LZTFL1
2518889	-0.98217572	0.047834572	ZNF804A
2449693	-0.79123777	0.048009874	DENND1B
2996321	-0.90048092	0.048044828	BBS9
3714729	0.9620262	0.0481413	MAP2K3
2480992	-0.79796958	0.048318637	MSH2
3750767	0.93184988	0.048347407	ALDOC
3872310	-0.9364235	0.048373983	ZNF550
3448152	-0.92895198	0.048441227	ITPR2
3127579	0.83825053	0.048453825	BIN3-IT1
3918779	0.93799322	0.048457338	ITSN1
3043895	1.12961948	0.048601686	SCRN1
3019401	-0.8577958	0.048727704	ZNF277
3218067	-0.91726584	0.048789731	MRPL50
2758870	-1.19417111	0.048794038	CYTL1
3092663	-0.7365677	0.049051819	WRN
3835361	-0.85976206	0.049178746	ZNF222
3107151	-0.83138116	0.049217779	FAM92A1
2449619	-0.983398	0.049220271	ZBTB41
3285926	-1.13688091	0.049230279	ZNF33B
3444329	-1.16084176	0.049332013	TAS2R10
2418451	-1.29409985	0.049359588	CRYZ
3451670	-0.91050187	0.049359962	PUS7L
2953435	-0.73582068	0.049394497	OARD1
3610804	1.0633536	0.049412039	IGF1R
3066818	1.16445922	0.049509432	NAMPT
4013828	-0.82089638	0.049602567	HMGN5
2498951	-1.40235414	0.049624534	SULT1C4
3766480	0.71545416	0.049738117	CSH1
3332615	0.74939679	0.049860529	TMEM109

Supplementary Table 3C. The Cancer Genome Atlas AML samples 183 Microarrays. Top upregulated and top downregulated genes
Analysis of AML patients with the top low WT1 expression (7 microarrays) vs. the top high WT1 (7), ranked by significance (P Value < 0.05).

PROBEID	ENTREZID	SYMBOL	GENENAME	logFC	P.Value
206067_s_at	7490	WT1	Wilms tumor 1	-5.83648078	2.2564E-19
229629_at	NA	NA	NA	-4.28210598	2.3127E-11
201069_at	4313	MMP2	matrix metallopeptidase 2	-4.56479284	2.5932E-10
219837_s_at	54360	CYTL1	cytokine-like 1	-5.60636345	1.5883E-09
206761_at	10225	CD96	CD96 molecule	-4.56108661	2.0191E-09
216614_at	NA	NA	NA	-3.6033013	2.1513E-09
244457_at	NA	NA	NA	-2.99917608	1.0065E-08
205624_at	1359	CPA3	carboxypeptidase A3 (mast cell)	-7.17883044	1.0353E-08
231982_at	284422	SMIM24	small integral membrane protein 24	-3.60243086	1.0493E-08
205349_at	2769	GNA15	guanine nucleotide binding protein (G protein), alpha 15 (Gq class)	-2.49378934	1.5092E-08
1555120_at	10225	CD96	CD96 molecule	-3.48983507	1.5564E-08
209790_s_at	839	CASP6	caspase 6, apoptosis-related cysteine peptidase	-2.1993393	2.5464E-08
223075_s_at	83543	AIF1L	allograft inflammatory factor 1-like	-3.24449987	2.8676E-08
240458_at	NA	NA	NA	-3.19171199	3.2023E-08
202746_at	9452	ITM2A	integral membrane protein 2A	-5.60173636	3.3578E-08
225671_at	124976	SPNS2	spinster homolog 2 (Drosophila)	-1.81639522	4.5303E-08
223204_at	51313	FAM198B	family with sequence similarity 198, member B	4.55070368	4.6968E-08
202661_at	3709	ITPR2	inositol 1,4,5-trisphosphate receptor, type 2	-2.53902338	5.0384E-08
211071_s_at	10962	MLLT11	myeloid/lymphoid or mixed-lineage leukemia (trithorax homolog, Drosophila); translc	-2.60965232	5.6522E-08
201243_s_at	481	ATP1B1	ATPase, Na+/K+ transporting, beta 1 polypeptide	-3.22503289	6.1184E-08
228854_at	NA	NA	NA	-3.76186816	6.5719E-08
213395_at	23209	MLC1	megalencephalic leukoencephalopathy with subcortical cysts 1	-2.78280394	6.6099E-08
228654_at	139886	SPIN4	spindlin family, member 4	-2.62256427	9.3731E-08
233072_at	84628	NTNG2	netrin G2	-3.27726851	9.3894E-08
201549_x_at	10765	KDM5B	lysine (K)-specific demethylase 5B	-2.09293326	1.0007E-07
213348_at	1028	CDKN1C	cyclin-dependent kinase inhibitor 1C (p57, Kip2)	-3.016334	1.4731E-07
228550_at	65078	RTN4R	reticulon 4 receptor	-2.21899038	1.4896E-07
227556_at	481 | 29922	ATP1B1 | NME7	ATPase, Na+/K+ transporting, beta 1 polypeptide | NME/NM23 family member 7	-3.65794108	1.4975E-07
201596_x_at	3875	KRT18	keratin 18, type I	-3.51464201	1.944E-07
211202_s_at	10765	KDM5B	lysine (K)-specific demethylase 5B	-1.95944225	2.123E-07
223922_x_at	64231	MS4A6A	membrane-spanning 4-domains, subfamily A, member 6A	4.78295158	2.1509E-07
212657_s_at	3557	IL1RN	interleukin 1 receptor antagonist	3.06716407	2.2388E-07
219443_at	55617	TASP1	taspase, threonine aspartase, 1	-1.76791035	2.304E-07
209710_at	2624	GATA2	GATA binding protein 2	-3.60279811	2.469E-07
213182_x_at	1028	CDKN1C	cyclin-dependent kinase inhibitor 1C (p57, Kip2)	-1.9455896	2.5553E-07
225305_at	123096	SLC25A29	solute carrier family 25 (mitochondrial carnitine/acylcarnitine carrier), member 29	-1.73788848	3.0757E-07

218035_s_at	54502	RBM47	RNA binding motif protein 47	3.87641592	3.1144E-07
213624_at	10924	SMPDL3A	sphingomyelin phosphodiesterase, acid-like 3A	2.07845801	3.2037E-07
222477_s_at	51768	TM7SF3	transmembrane 7 superfamily member 3	-1.65776384	3.3772E-07
230550_at	64231	MS4A6A	membrane-spanning 4-domains, subfamily A, member 6A	5.76305974	3.3931E-07
226291_at	57679	ALS2	amyotrophic lateral sclerosis 2 (juvenile)	-2.00251427	3.4932E-07
226841_at	219972	MPEG1	macrophage expressed 1	4.86725032	3.668E-07
203151_at	4130	MAP1A	microtubule-associated protein 1A	-3.38196064	3.6861E-07
207735_at	54941	RNF125	ring finger protein 125, E3 ubiquitin protein ligase	-2.36358746	3.747E-07
57588_at	57419	SLC24A3	solute carrier family 24 (sodium/potassium/calcium exchanger), member 3	-3.30452579	3.8334E-07
228885_at	256691	MAMDC2	MAM domain containing 2	-4.42436817	4.0502E-07
232280_at	123096	SLC25A29	solute carrier family 25 (mitochondrial carnitine/acylcarnitine carrier), member 29	-2.30646351	4.1135E-07
223280_x_at	64231	MS4A6A	membrane-spanning 4-domains, subfamily A, member 6A	5.28565799	4.3458E-07
202660_at	3709	ITPR2	inositol 1,4,5-trisphosphate receptor, type 2	-2.95928684	5.2361E-07
205883_at	7704	ZBTB16	zinc finger and BTB domain containing 16	-2.45776934	5.3288E-07
225306_s_at	123096	SLC25A29	solute carrier family 25 (mitochondrial carnitine/acylcarnitine carrier), member 29	-1.88621607	5.5613E-07
206954_at	51352	WT1-AS	WT1 antisense RNA	-1.74385352	5.6314E-07
226602_s_at	613	BCR	breakpoint cluster region	-1.87357161	5.6414E-07
202888_s_at	290	ANPEP	alanyl (membrane) aminopeptidase	-2.51424538	7.3436E-07
232234_at	84174	SLA2	Src-like-adaptor 2	-2.08924202	7.3567E-07
1552908_at	148823	GCSAML	germinal center-associated, signaling and motility-like	-3.2073471	7.421E-07
225962_at	84937	ZNRF1	zinc and ring finger 1, E3 ubiquitin protein ligase	-2.53717212	7.6912E-07
240539_at	NA	NA	NA	-2.95762433	8.2821E-07
224356_x_at	64231	MS4A6A	membrane-spanning 4-domains, subfamily A, member 6A	5.68705676	8.4164E-07
204604_at	5218	CDK14	cyclin-dependent kinase 14	-2.18950939	9.0232E-07
208056_s_at	863	CBFA2T3	core-binding factor, runt domain, alpha subunit 2; translocated to, 3	-1.89020851	9.1912E-07
1561690_at	NA	NA	NA	-2.76056513	9.5595E-07
209332_s_at	4149	MAX	MYC associated factor X	-1.24563144	9.6793E-07
219534_x_at	1028	CDKN1C	cyclin-dependent kinase inhibitor 1C (p57, Kip2)	-2.39757508	1.0858E-06
205739_x_at	51427	ZNF107	zinc finger protein 107	-2.48870463	1.161E-06
230266_at	338382	RAB7B	RAB7B, member RAS oncogene family	-3.14961622	1.2255E-06
218966_at	55930	MYO5C	myosin VC	-2.65902401	1.2546E-06
226818_at	219972	MPEG1	macrophage expressed 1	5.34548459	1.2623E-06
226531_at	84876	ORAI1	ORAI calcium release-activated calcium modulator 1	-1.8998166	1.2869E-06
203547_at	920	CD4	CD4 molecule	2.36235246	1.2869E-06
202956_at	10565	ARFGEF1	ADP-ribosylation factor guanine nucleotide-exchange factor 1 (brefeldin A-inhibited)	-1.29045834	1.3276E-06
227649_s_at	23380 | 6471	SRGAP2 | SRGAP2B |	SLIT-ROBO Rho GTPase activating protein 2 | SLIT-ROBO Rho GTPase activating prote	1.49034159	1.3662E-06
236685_at	NA	NA	NA	-2.74753958	1.3772E-06
213541_s_at	2078	ERG	v-ets avian erythroblastosis virus E26 oncogene homolog	-3.56514439	1.3839E-06
203787_at	23635	SSBP2	single-stranded DNA binding protein 2	-2.84109486	1.3842E-06
202718_at	3485	IGFBP2	insulin-like growth factor binding protein 2, 36kDa	-3.94613613	1.4507E-06
204961_s_at	653361 | 6548	NCF1 | NCF1B | NCF1(	neutrophil cytosolic factor 1 | neutrophil cytosolic factor 1B pseudogene | neutrophil (	3.26499217	1.4963E-06
219090_at	57419	SLC24A3	solute carrier family 24 (sodium/potassium/calcium exchanger), member 3	-3.54500255	1.53E-06
210152_at	11006	LILRB4	leukocyte immunoglobulin-like receptor, subfamily B (with TM and ITIM domains), me	3.13151174	1.745E-06
202747_s_at	9452	ITM2A	integral membrane protein 2A	-4.35872183	1.828E-06
206710_s_at	23136	EPB41L3	erythrocyte membrane protein band 4.1-like 3	2.40530898	1.8458E-06
219666_at	64231	MS4A6A	membrane-spanning 4-domains, subfamily A, member 6A	5.10955328	1.8827E-06
209286_at	10602	CDC42EP3	CDC42 effector protein (Rho GTPase binding) 3	3.36679848	2.0255E-06
222496_s_at	54502	RBM47	RNA binding motif protein 47	3.43788328	2.2043E-06
202315_s_at	613	BCR	breakpoint cluster region	-1.69426923	2.2426E-06
213110_s_at	1287	COL4A5	collagen, type IV, alpha 5	-5.43053691	2.2501E-06
225485_at	95681	CEP41	centrosomal protein 41kDa	-2.01841452	2.3251E-06
235142_at	653121	ZBTB8A	zinc finger and BTB domain containing 8A	-3.50857177	2.4757E-06
215411_s_at	10758	TRAF3IP2	TRAF3 interacting protein 2	-1.7393162	2.6231E-06
226869_at	1953	MEGF6	multiple EGF-like-domains 6	-2.53034807	2.6365E-06
216268_s_at	182	JAG1	jagged 1	-4.13674118	2.6384E-06
209935_at	27032	ATP2C1	ATPase, Ca++ transporting, type 2C, member 1	-1.85724559	2.6384E-06
200986_at	710	SERPING1	serpin peptidase inhibitor, clade G (C1 inhibitor), member 1	-3.72110446	2.7231E-06
204502_at	25939	SAMHD1	SAM domain and HD domain 1	3.71408898	3.0032E-06
221522_at	84079	ANKRD27	ankyrin repeat domain 27 (VPS9 domain)	-1.76984351	3.1165E-06
205936_s_at	3101	HK3	hexokinase 3 (white cell)	2.46472662	3.1323E-06
216894_x_at	1028	CDKN1C	cyclin-dependent kinase inhibitor 1C (p57, Kip2)	-1.68540755	3.1435E-06
220134_x_at	55194	EVA1B	eva-1 homolog B (C. elegans)	-1.52423902	3.2353E-06
209686_at	6285	S100B	S100 calcium binding protein B	-3.29157509	3.2792E-06
211464_x_at	839	CASP6	caspase 6, apoptosis-related cysteine peptidase	-1.45052892	3.29E-06
202917_s_at	6279	S100A8	S100 calcium binding protein A8	5.17000548	3.3138E-06
230387_at	NA	NA	NA	-1.66826616	3.4266E-06
226817_at	1824	DSC2	desmocollin 2	-3.45172505	3.5007E-06
204960_at	5790	PTPRCAP	protein tyrosine phosphatase, receptor type, C-associated protein	-1.98295879	3.5768E-06
235647_at	11154	AP4S1	adaptor-related protein complex 4, sigma 1 subunit	-1.63216082	3.6093E-06
225685_at	10602	CDC42EP3	CDC42 effector protein (Rho GTPase binding) 3	3.80485455	3.8219E-06
223344_s_at	58475	MS4A7	membrane-spanning 4-domains, subfamily A, member 7	3.80986341	3.824E-06
211101_x_at	11027	LILRA2	leukocyte immunoglobulin-like receptor, subfamily A (with TM domain), member 2	2.61521863	3.8926E-06
212922_s_at	56950	SMYD2	SET and MYND domain containing 2	-1.39024715	3.9682E-06
203857_s_at	10954 | 10246	PDIA5 | MIR7110	protein disulfide isomerase family A, member 5 | microRNA 7110	-1.58107241	3.9765E-06
204994_at	4600	MX2	MX dynamin-like GTPase 2	1.88188206	4.1521E-06
204834_at	10875	FGL2	fibrinogen-like 2	3.2629497	4.2008E-06
235094_at	NA	NA	NA	-2.57488596	4.3246E-06
212599_at	26053	AUTS2	autism susceptibility candidate 2	-3.16646324	4.4883E-06
243904_at	134957	STXBP5	syntaxin binding protein 5 (tomosyn)	-1.9500042	4.6252E-06
212764_at	6935	ZEB1	zinc finger E-box binding homeobox 1	-3.22147025	4.6618E-06
209615_s_at	5058	PAK1	p21 protein (Cdc42/Rac)-activated kinase 1	2.14376286	4.7008E-06
201392_s_at	3482	IGF2R	insulin-like growth factor 2 receptor	2.53625359	4.724E-06
201393_s_at	3482	IGF2R	insulin-like growth factor 2 receptor	2.62057282	5.0439E-06
1556682_s_at	NA	NA	NA	-2.58689011	5.0468E-06
236474_at	NA	NA	NA	-1.88457116	5.2314E-06
212796_s_at	23102	TBC1D2B	TBC1 domain family, member 2B	-1.3876382	5.3101E-06
225959_s_at	84937	ZNRF1	zinc and ring finger 1, E3 ubiquitin protein ligase	-1.69213521	5.6427E-06
219607_s_at	51338	MS4A4A	membrane-spanning 4-domains, subfamily A, member 4A	4.85860449	5.6777E-06
219872_at	51313	FAM198B	family with sequence similarity 198, member B	2.4600561	5.7997E-06
204057_at	3394	IRF8	interferon regulatory factor 8	3.67889892	5.8196E-06
50221_at	7942	TFEB	transcription factor EB	1.64451397	5.9263E-06
223000_s_at	50848	F11R	F11 receptor	-2.24062328	6.0332E-06
225019_at	817	CAMK2D	calcium/calmodulin-dependent protein kinase II delta	1.998168	6.0457E-06
1557813_at	NA	NA	NA	-2.31267495	6.1087E-06
208792_s_at	1191	CLU	clusterin	-3.43943167	6.1748E-06
202855_s_at	9123 | 102465	SLC16A3 | MIR6787	solute carrier family 16 (monocarboxylate transporter), member 3 | microRNA 6787	2.02076041	6.1885E-06
213566_at	6039	RNASE6	ribonuclease, RNase A family, k6	4.37906609	6.3213E-06
201548_s_at	10765	KDM5B	lysine (K)-specific demethylase 5B	-1.86980719	6.4217E-06
227607_at	57559	STAMBPL1	STAM binding protein-like 1	-2.03242147	6.6327E-06
239361_at	NA	NA	NA	-1.75837304	6.8427E-06
237483_at	NA	NA	NA	-2.5133094	7.1118E-06
209099_x_at	182	JAG1	jagged 1	-3.79373131	7.1597E-06
203535_at	6280	S100A9	S100 calcium binding protein A9	4.99947338	7.1748E-06
208438_s_at	2268	FGR	FGR proto-oncogene, Src family tyrosine kinase	3.53164958	7.2778E-06

223599_at	117854	TRIM6	tripartite motif containing 6	-2.67390669	7.3168E-06
226651_at	9456	HOMER1	homer scaffolding protein 1	-1.86656978	7.4061E-06
212813_at	83700	JAM3	junctional adhesion molecule 3	-2.44058443	7.4715E-06
233031_at	9839	ZEB2	zinc finger E-box binding homeobox 2	1.9806465	7.6423E-06
234643_x_at	NA	NA	NA	-2.91526086	7.7174E-06
1561167_at	NA	NA	NA	-2.34405227	7.7411E-06
218149_s_at	55893	ZNF395	zinc finger protein 395	-1.92482186	7.7862E-06
212236_x_at	3728 | 3872	JUP | KRT17	junction plakoglobin | keratin 17, type I	-1.44036148	7.8004E-06
212642_s_at	3097	HIVEP2	human immunodeficiency virus type I enhancer binding protein 2	-1.78172909	7.8111E-06
220338_at	55103	RALGPS2	Ral GEF with PH domain and SH3 binding motif 2	-2.30644709	8.1636E-06
1554624_a_at	10326	SIRPB1	signal-regulatory protein beta 1	2.55290605	8.1742E-06
234987_at	25939	SAMHD1	SAM domain and HD domain 1	3.85581128	8.2982E-06
1566557_at	440465	BAIAP2-AS1	BAIAP2 antisense RNA 1 (head to head)	-2.65664293	8.526E-06
214450_at	1521	CTSW	cathepsin W	-3.07906913	8.82E-06
244889_at	400499	LOC400499	uncharacterized LOC400499	-2.38814324	8.8281E-06
213183_s_at	1028	CDKN1C	cyclin-dependent kinase inhibitor 1C (p57, Kip2)	-2.44543092	9.0285E-06
244743_x_at	7697	ZNF138	zinc finger protein 138	-1.28980225	9.0571E-06
227762_at	NA	NA	NA	-2.26530788	9.0697E-06
223627_at	84206	MEX3B	mex-3 RNA binding family member B	-2.43687972	9.1128E-06
210829_s_at	23635	SSBP2	single-stranded DNA binding protein 2	-2.3702419	9.147E-06
204044_at	23475	QPRT	quinolinate phosphoribosyltransferase	-1.86008956	9.2253E-06
201063_at	5954	RCN1	reticulocalbin 1, EF-hand calcium binding domain	-1.58725833	9.5957E-06
235964_x_at	25939	SAMHD1	SAM domain and HD domain 1	3.06852697	9.7555E-06
204081_at	4900	NRGN	neurogranin (protein kinase C substrate, RC3)	2.05073678	9.9945E-06
214623_at	26226	FBXW4P1	F-box and WD repeat domain containing 4 pseudogene 1	-1.44502149	1.0297E-05
229510_at	84689	MS4A14	membrane-spanning 4-domains, subfamily A, member 14	2.43132669	1.121E-05
223343_at	58475	MS4A7	membrane-spanning 4-domains, subfamily A, member 7	4.08297433	1.1438E-05
212688_at	5291	PIK3CB	phosphatidylinositol-4,5-bisphosphate 3-kinase, catalytic subunit beta	-1.66349201	1.1594E-05
235593_at	9839	ZEB2	zinc finger E-box binding homeobox 2	2.06732529	1.1847E-05
1554280_a_at	257169	C9orf43	chromosome 9 open reading frame 43	-2.0200088	1.188E-05
205076_s_at	10903	MTMR11	myotubularin related protein 11	3.56328467	1.1973E-05
239619_at	NA	NA	NA	-2.16231216	1.2119E-05
205789_at	912	CD1D	CD1d molecule	3.75480068	1.2406E-05
221123_x_at	55893 | 15757	ZNF395 | FBXO16	zinc finger protein 395 | F-box protein 16	-1.54988278	1.249E-05
214298_x_at	23157	SEPT6	septin 6	-2.14910197	1.2651E-05
203970_s_at	8504	PEX3	peroxisomal biogenesis factor 3	-1.69776064	1.2774E-05
224358_s_at	58475	MS4A7	membrane-spanning 4-domains, subfamily A, member 7	3.43611177	1.2954E-05
208791_at	1191	CLU	clusterin	-3.27307011	1.319E-05
214084_x_at	654816 | 6533	NCF1B | NCF1	neutrophil cytosolic factor 1B pseudogene | neutrophil cytosolic factor 1	2.61437001	1.3331E-05
232693_s_at	55893 | 15757	ZNF395 | FBXO16	zinc finger protein 395 | F-box protein 16	-1.66569553	1.3739E-05
1563453_at	NA	NA	NA	-2.4035004	1.3938E-05
228442_at	4773	NFATC2	nuclear factor of activated T-cells, cytoplasmic, calcineurin-dependent 2	-2.19145488	1.4005E-05
221656_s_at	55160	ARHGEF10L	Rho guanine nucleotide exchange factor (GEF) 10-like	1.48096223	1.4271E-05
211748_x_at	5730	PTGDS	prostaglandin D2 synthase 21kDa (brain)	-3.45625443	1.4513E-05
208991_at	6774	STAT3	signal transducer and activator of transcription 3 (acute-phase response factor)	-1.21137885	1.4542E-05
214228_x_at	7293	TNFRSF4	tumor necrosis factor receptor superfamily, member 4	-2.47888546	1.4543E-05
244043_at	7029	TFDP2	transcription factor Dp-2 (E2F dimerization partner 2)	-2.46013493	1.5074E-05
205051_s_at	3815	KIT	v-kit Hardy-Zuckerman 4 feline sarcoma viral oncogene homolog	-3.03685328	1.5165E-05
211100_x_at	11027	LILRA2	leukocyte immunoglobulin-like receptor, subfamily A (with TM domain), member 2	2.34171996	1.5316E-05
223952_x_at	10170	DHRS9	dehydrogenase/reductase (SDR family) member 9	3.78438967	1.5467E-05
217118_s_at	23313	KIAA0930	KIAA0930	1.36818695	1.5578E-05
219654_at	9200	HACD1	3-hydroxyacyl-CoA dehydratase 1	-1.64975252	1.5831E-05
204152_s_at	4242	MFNG	MFNG O-fucosylpeptide 3-beta-N-acetylglucosaminyltransferase	1.76907197	1.5842E-05
1555736_a_at	57085	AGTRAP	angiotensin II receptor-associated protein	2.13930459	1.6994E-05
232951_at	NA	NA	NA	-1.45607517	1.7187E-05
223216_x_at	55893 | 15757	ZNF395 | FBXO16	zinc finger protein 395 | F-box protein 16	-1.63164681	1.7517E-05
205898_at	1524	CX3CR1	chemokine (C-X3-C motif) receptor 1	3.6340374	1.7737E-05
235056_at	2120	ETV6	ets variant 6	-1.54411368	1.8046E-05
235529_x_at	25939	SAMHD1	SAM domain and HD domain 1	2.8302658	1.8132E-05
224009_x_at	10170	DHRS9	dehydrogenase/reductase (SDR family) member 9	3.99383412	1.8141E-05
209318_x_at	5325	PLAGL1	pleiomorphic adenoma gene-like 1	-1.72404666	1.8519E-05
211654_x_at	3119	HLA-DQB1	major histocompatibility complex, class II, DQ beta 1	2.71067291	1.8719E-05
221710_x_at	55194	EVA1B	eva-1 homolog B (C. elegans)	-1.57434967	1.8892E-05
225532_at	91768	CABLES1	Cdk5 and Abl enzyme substrate 1	-1.48447255	1.903E-05
203323_at	858	CAV2	caveolin 2	-2.0875614	1.9304E-05
208613_s_at	2317	FLNB	filamin B, beta	1.87166786	1.9953E-05
1566558_x_at	440465	BAIAP2-AS1	BAIAP2 antisense RNA 1 (head to head)	-2.18946678	2.0567E-05
206660_at	3543	IGLL1	immunoglobulin lambda-like polypeptide 1	-3.14501705	2.092E-05
212658_at	10184	LHFPL2	lipoma HMGIC fusion partner-like 2	-2.02807788	2.1004E-05
223162_s_at	57189	KIAA1147	KIAA1147	-1.649806	2.1054E-05
212414_s_at	23157 | 84656	SEPT6 | GLYR1	septin 6 | glyoxylate reductase 1 homolog (Arabidopsis)	-1.96142475	2.1568E-05
219615_s_at	8645	KCNK5	potassium channel, two pore domain subfamily K, member 5	-2.10928328	2.1578E-05
220266_s_at	9314	KLF4	Kruppel-like factor 4 (gut)	2.77125448	2.1766E-05
209288_s_at	10602	CDC42EP3	CDC42 effector protein (Rho GTPase binding) 3	3.56916605	2.1827E-05
1557814_a_at	NA	NA	NA	-1.72550032	2.1989E-05
204254_s_at	7421	VDR	vitamin D (1,25- dihydroxyvitamin D3) receptor	1.56167461	2.2106E-05
201826_s_at	51097	SCCPDH	saccharopine dehydrogenase (putative)	-1.46134812	2.23E-05
207002_s_at	5325	PLAGL1	pleiomorphic adenoma gene-like 1	-1.75611079	2.3501E-05
203324_s_at	858	CAV2	caveolin 2	-2.27743357	2.3707E-05
203146_s_at	2550	GABBR1	gamma-aminobutyric acid (GABA) B receptor, 1	1.84316143	2.3986E-05
225263_at	9394	HS6ST1	heparan sulfate 6-O-sulfotransferase 1	-1.11354115	2.4227E-05
213733_at	4542	MYO1F	myosin IF	1.78320166	2.5522E-05
226384_at	84513	PPAPDC1B	phosphatidic acid phosphatase type 2 domain containing 1B	-1.56477659	2.5531E-05
214177_s_at	57326	PBXIP1	pre-B-cell leukemia homeobox interacting protein 1	-1.67029769	2.5715E-05
221652_s_at	55726	ASUN	asunder spermatogenesis regulator	-1.2192709	2.5796E-05
232500_at	57186	RALGAPA2	Ral GTPase activating protein, alpha subunit 2 (catalytic)	-1.46654224	2.6351E-05
227558_at	8535	CBX4	chromobox homolog 4	-1.23314963	2.6853E-05
219366_at	57099	AVEN	apoptosis, caspase activation inhibitor	-1.50740282	2.6946E-05
215248_at	2887	GRB10	growth factor receptor-bound protein 10	-2.42030214	2.8149E-05
1564204_at	57234	LINC00869	long intergenic non-protein coding RNA 869	-2.20950652	2.8842E-05
1555349_a_at	3689	ITGB2	integrin, beta 2 (complement component 3 receptor 3 and 4 subunit)	3.20734087	2.9067E-05
205110_s_at	2258	FGF13	fibroblast growth factor 13	-3.73239742	2.9246E-05
219358_s_at	55803	ADAP2	ArfGAP with dual PH domains 2	1.70296904	2.9815E-05
213627_at	10916	MAGED2	melanoma antigen family D2	-1.3861605	3.0471E-05
221864_at	93129	ORAI3	ORAI calcium release-activated calcium modulator 3	-1.37810393	3.0496E-05
242471_at	NA	NA	NA	1.76563794	3.0536E-05
214743_at	1523	CUX1	cut-like homeobox 1	-1.3340707	3.0961E-05
215513_at	57061	HYMAI	hydatidiform mole associated and imprinted (non-protein coding)	-1.82862449	3.1014E-05
226478_at	51768	TM7SF3	transmembrane 7 superfamily member 3	-2.15413633	3.1129E-05
203162_s_at	10300	KATNB1	katanin p80 (WD repeat containing) subunit B 1	-1.32946932	3.1159E-05
231175_at	221336	BEND6	BEN domain containing 6	-3.00626632	3.1842E-05
212812_at	256987	SERINC5	serine incorporator 5	-2.3486705	3.1961E-05
212998_x_at	3119	HLA-DQB1	major histocompatibility complex, class II, DQ beta 1	3.37596808	3.2033E-05
238784_at	283417	DPY19L2	dpy-19-like 2 (C. elegans)	-2.99570671	3.284E-05

202609_at	2059	EPS8	epidermal growth factor receptor pathway substrate 8	3.05305076	3.328E-05
203972_s_at	8504	PEX3	peroxisomal biogenesis factor 3	-1.30607862	3.441E-05
228696_at	85414	SLC45A3	solute carrier family 45, member 3	-2.02436705	3.4841E-05
212415_at	23157	SEPT6	septin 6	-1.64156238	3.4993E-05
1559883_s_at	25939	SAMHD1	SAM domain and HD domain 1	3.40223644	3.5148E-05
217936_at	394	ARHGAP5	Rho GTPase activating protein 5	-2.45742719	3.5321E-05
202856_s_at	9123 | 102465	SLC16A3 | MIR6787	solute carrier family 16 (monocarboxylate transporter), member 3 | microRNA 6787	2.62437678	3.5339E-05
203624_at	8227	AKAP17A	A kinase (PRKA) anchor protein 17A	-0.84499362	3.5419E-05
231907_at	27	ABL2	ABL proto-oncogene 2, non-receptor tyrosine kinase	-0.98657232	3.544E-05
239383_at	NA	NA	NA	-1.28415621	3.7006E-05
235227_at	134957	STXBP5	syntaxin binding protein 5 (tomosyn)	-2.35487176	3.7452E-05
228058_at	124220	ZG16B	zymogen granule protein 16B	-1.84984012	3.8125E-05
202877_s_at	22918	CD93	CD93 molecule	2.26263782	3.8276E-05
222687_s_at	55331	ACER3	alkaline ceramidase 3	1.3578661	3.8438E-05
203760_s_at	6503	SLA	Src-like-adaptor	1.89297782	3.846E-05
201875_s_at	9019	MPZL1	myelin protein zero-like 1	-1.2151055	3.9476E-05
201427_s_at	6414	SEPP1	selenoprotein P, plasma, 1	-4.67891687	3.979E-05
232095_at	NA	NA	NA	2.13327764	4.0314E-05
234260_at	NA	NA	NA	-2.18168274	4.0744E-05
227055_at	196410	METTL7B	methyltransferase like 7B	2.10409154	4.0767E-05
212187_x_at	5730	PTGDS	prostaglandin D2 synthase 21kDa (brain)	-3.12240775	4.0797E-05
225330_at	3480	IGF1R	insulin-like growth factor 1 receptor	-2.93414833	4.1247E-05
202803_s_at	3689	ITGB2	integrin, beta 2 (complement component 3 receptor 3 and 4 subunit)	2.79390592	4.1298E-05
203467_at	5372	PMM1	phosphomannomutase 1	1.32038264	4.1325E-05
209824_s_at	406	ARNTL	aryl hydrocarbon receptor nuclear translocator-like	1.39703756	4.2238E-05
213266_at	7158	TP53BP1	tumor protein p53 binding protein 1	-1.21890815	4.2853E-05
232810_at	51390	AIG1	androgen-induced 1	-2.38516875	4.3549E-05
224847_at	1021	CDK6	cyclin-dependent kinase 6	-1.44251746	4.3644E-05
217286_s_at	57446	NDRG3	NDRG family member 3	-1.06415532	4.4117E-05
222043_at	1191	CLU	clusterin	-1.93801416	4.4348E-05
235661_at	5452	POU2F2	POU class 2 homeobox 2	1.32740396	4.4585E-05
227522_at	134147	CMBL	carboxymethylenebutenolidase homolog (Pseudomonas)	-2.53581881	4.4684E-05
210184_at	3687	ITGAX	integrin, alpha X (complement component 3 receptor 4 subunit)	1.4988144	4.492E-05
206978_at	729230	CCR2	chemokine (C-C motif) receptor 2	4.41679345	4.5369E-05
207104_x_at	10859	LILRB1	leukocyte immunoglobulin-like receptor, subfamily B (with TM and ITIM domains), me	2.00279597	4.5557E-05
50314_i_at	54976	C20orf27	chromosome 20 open reading frame 27	1.35152943	4.6909E-05
227547_at	NA	NA	NA	-1.08355345	4.7183E-05
236198_at	101929623	LINC01215	long intergenic non-protein coding RNA 1215	-2.34879979	4.7496E-05
219799_s_at	10170	DHRS9	dehydrogenase/reductase (SDR family) member 9	3.19195061	4.7953E-05
209934_s_at	27032	ATP2C1	ATPase, Ca++ transporting, type 2C, member 1	-1.28662891	4.8187E-05
1555961_a_at	3094	HINT1	histidine triad nucleotide binding protein 1	-1.33278363	4.8615E-05
214176_s_at	57326	PBXIP1	pre-B-cell leukemia homeobox interacting protein 1	-1.43381748	4.8811E-05
212501_at	1051	CEBPB	CCAAT/enhancer binding protein (C/EBP), beta	1.92029558	4.8947E-05
225391_at	93622	LOC93622	Morf4 family associated protein 1-like 1 pseudogene	-1.24348846	4.9248E-05
214054_at	9046	DOK2	docking protein 2, 56kDa	1.66586533	5.0648E-05
212509_s_at	439921	MXRA7	matrix-remodelling associated 7	-2.26305503	5.2112E-05
219908_at	27123	DKK2	dickkopf WNT signaling pathway inhibitor 2	2.1082283	5.2292E-05
209948_at	3779	KCNMB1	potassium channel subfamily M regulatory beta subunit 1	1.66869226	5.2441E-05
212413_at	23157	SEPT6	septin 6	-1.93111381	5.2624E-05
240008_at	NA	NA	NA	-1.72696429	5.2795E-05
227533_at	55103	RALGPS2	Ral GEF with PH domain and SH3 binding motif 2	-1.50788463	5.3337E-05
202662_s_at	3709	ITPR2	inositol 1,4,5-trisphosphate receptor, type 2	-2.34071189	5.3728E-05
205780_at	638	BIK	BCL2-interacting killer (apoptosis-inducing)	-2.61403798	5.4539E-05
233849_s_at	394	ARHGAP5	Rho GTPase activating protein 5	-2.98744986	5.5215E-05
225112_at	10152	ABI2	abl-interactor 2	-1.5505169	5.5289E-05
236295_s_at	197358	NLRC3	NLR family, CARD domain containing 3	-1.96509695	5.5337E-05
207721_x_at	3094	HINT1	histidine triad nucleotide binding protein 1	-1.33096378	5.6076E-05
241926_s_at	2078	ERG	v-ets avian erythroblastosis virus E26 oncogene homolog	-2.33723948	5.6308E-05
1553982_a_at	338382	RAB7B	RAB7B, member RAS oncogene family	-1.98194534	5.6537E-05
1563088_a_at	284837	AATBC	apoptosis associated transcript in bladder cancer	1.94399874	5.6597E-05
215659_at	55876	GSDMB	gasdermin B	-1.59526751	5.6778E-05
209287_s_at	10602	CDC42EP3	CDC42 effector protein (Rho GTPase binding) 3	1.93759134	5.8021E-05
208682_s_at	10916	MAGED2	melanoma antigen family D2	-0.95535827	5.9624E-05
215388_s_at	3078 | 3075	CFHR1 | CFH	complement factor H-related 1 | complement factor H	-2.89020258	5.9674E-05
240155_x_at	284443	ZNF493	zinc finger protein 493	-1.75823735	5.9719E-05
39248_at	360	AQP3	aquaporin 3 (Gill blood group)	-3.16214877	5.9726E-05
214316_x_at	NA	NA	NA	-2.28854495	5.9782E-05
204670_x_at	3127 | 3123 |	HLA-DRB5 | HLA-DRB:	major histocompatibility complex, class II, DR beta 5 | major histocompatibility comp	2.6871003	6.0066E-05
231205_at	NA	NA	NA	1.98423835	6.124E-05
225240_s_at	124540	MSI2	musashi RNA-binding protein 2	-3.52364237	6.1355E-05
211663_x_at	5730	PTGDS	prostaglandin D2 synthase 21kDa (brain)	-2.55918444	6.1637E-05
211102_s_at	11027	LILRA2	leukocyte immunoglobulin-like receptor, subfamily A (with TM domain), member 2	2.36101563	6.1745E-05
1559154_at	NA	NA	NA	-1.41266754	6.251E-05
233713_at	NA	NA	NA	-1.90691338	6.3298E-05
221814_at	25960	ADGRA2	adhesion G protein-coupled receptor A2	2.10694748	6.3812E-05
209312_x_at	3123 | 3119 |	HLA-DRB1 | HLA-DQB:	major histocompatibility complex, class II, DR beta 1 | major histocompatibility comp	2.85354644	6.382E-05
207857_at	11027	LILRA2	leukocyte immunoglobulin-like receptor, subfamily A (with TM domain), member 2	3.23547242	6.5043E-05
229199_at	6335	SCN9A	sodium channel, voltage gated, type IX alpha subunit	-2.47955233	6.5529E-05
242054_s_at	6496	SIX3	SIX homeobox 3	-3.29016458	6.6201E-05
203508_at	7133	TNFRSF1B	tumor necrosis factor receptor superfamily, member 1B	2.70955618	6.7624E-05
201655_s_at	3339	HSPG2	heparan sulfate proteoglycan 2	-1.21679535	6.8456E-05
220742_s_at	55768	NGLY1	N-glycanase 1	-0.9272464	6.9891E-05
212999_x_at	3119	HLA-DQB1	major histocompatibility complex, class II, DQ beta 1	2.69989308	7.0045E-05
219477_s_at	55901 | 10088	THSD1 | MRPS31P5	thrombospondin, type I, domain containing 1 | mitochondrial ribosomal protein S31 p	-1.83700131	7.0191E-05
218656_s_at	10186	LHFP	lipoma HMGIC fusion partner	-2.43567415	7.0663E-05
212070_at	9289	ADGRG1	adhesion G protein-coupled receptor G1	-2.93231252	7.0924E-05
232935_at	NA	NA	NA	-2.3270212	7.0957E-05
226794_at	134957	STXBP5	syntaxin binding protein 5 (tomosyn)	-1.81808396	7.1368E-05
209213_at	873	CBR1	carbonyl reductase 1	1.36660827	7.2193E-05
229560_at	51311	TLR8	toll-like receptor 8	4.18645014	7.3127E-05
213363_at	340591	CA5BP1	carbonic anhydrase VB pseudogene 1	0.93354905	7.3401E-05
229459_at	25817	FAM19A5	family with sequence similarity 19 (chemokine (C-C motif)-like), member A5	-2.68260501	7.3764E-05
226507_at	5058	PAK1	p21 protein (Cdc42/Rac)-activated kinase 1	1.58760648	7.4102E-05
204576_s_at	23059	CLUAP1	clusterin associated protein 1	-0.85182578	7.4373E-05
200093_s_at	3094	HINT1	histidine triad nucleotide binding protein 1	-1.4926241	7.5295E-05
235019_at	1368	CPM	carboxypeptidase M	2.68695826	7.611E-05
232365_at	6477	SIAH1	siah E3 ubiquitin protein ligase 1	-2.42929722	7.6118E-05
220668_s_at	1789	DNMT3B	DNA (cytosine-5-)-methyltransferase 3 beta	-1.93457729	7.7168E-05
205684_s_at	55667	DENND4C	DENN/MADD domain containing 4C	-2.16499139	7.7944E-05
200706_s_at	9516	LITAF	lipopolysaccharide-induced TNF factor	-1.42061215	7.8256E-05
225935_at	1523	CUX1	cut-like homeobox 1	-1.30355129	7.8388E-05
217388_s_at	8942	KYNU	kynureninase	4.21933257	7.8581E-05
225704_at	57666	FBRSL1	fibrosin-like 1	-1.4651064	7.9223E-05
202371_at	79921	TCEAL4	transcription elongation factor A (SII)-like 4	-1.66787403	8.01E-05

1554616_at	5271	SERPINB8	serpin peptidase inhibitor, clade B (ovalbumin), member 8	0.95752641	8.08E-05
235443_at	100131067	CKMT2-AS1	CKMT2 antisense RNA 1	-1.60133561	8.1017E-05
225484_at	95681	CEP41	centrosomal protein 41kDa	-1.34715394	8.1058E-05
209098_s_at	182	JAG1	jagged 1	-2.03378106	8.2477E-05
1563792_at	81693	AMN	amnion associated transmembrane protein	-1.38389954	8.3867E-05
221755_at	254102	EHBP1L1	EH domain binding protein 1-like 1	1.48876349	8.4421E-05
216015_s_at	114548	NLRP3	NLR family, pyrin domain containing 3	2.50702636	8.4619E-05
242974_at	961	CD47	CD47 molecule	-1.85044273	8.4703E-05
238022_at	643911	CRNDE	colorectal neoplasia differentially expressed (non-protein coding)	-3.04351525	8.4868E-05
229693_at	388335	TMEM220	transmembrane protein 220	-1.42865122	9.0152E-05
203299_s_at	8905	AP1S2	adaptor-related protein complex 1, sigma 2 subunit	1.57089952	9.0764E-05
238021_s_at	643911	CRNDE	colorectal neoplasia differentially expressed (non-protein coding)	-4.07778152	9.2265E-05
212611_at	23220	DTX4	deltex 4, E3 ubiquitin ligase	1.6733676	9.3097E-05
228988_at	7552	ZNF711	zinc finger protein 711	-4.07558607	9.3164E-05
227629_at	5618	PRLR	prolactin receptor	2.82783892	9.3479E-05
1552553_a_at	58484	NLRC4	NLR family, CARD domain containing 4	2.11323667	9.3793E-05
1559502_s_at	126364	LRRC25	leucine rich repeat containing 25	1.95064581	9.4671E-05
226646_at	10365	KLF2	Kruppel-like factor 2	1.22665184	9.4851E-05
205099_s_at	1230	CCR1	chemokine (C-C motif) receptor 1	2.63535159	9.5439E-05
241723_at	10788	IQGAP2	IQ motif containing GTPase activating protein 2	-1.25966306	9.5501E-05
226157_at	7029	TFDP2	transcription factor Dp-2 (E2F dimerization partner 2)	-2.04937212	9.5566E-05
1563621_at	NA	NA	NA	-1.66645097	9.5611E-05
1559987_at	NA	NA	NA	-2.30387136	9.6827E-05
1568955_at	23380 | 6471	SRGAP2 | SRGAP2B |	SLIT-ROBO Rho GTPase activating protein 2 | SLIT-ROBO Rho GTPase activating prot	1.5612634	9.845E-05
201056_at	2804	GOLGB1	golgin B1	-0.94746869	9.8812E-05
207943_x_at	5325	PLAGL1	pleiomorphic adenoma gene-like 1	-1.68153175	9.9389E-05
207559_s_at	9203	ZMYM3	zinc finger, MYM-type 3	-0.94345773	0.0001022
224049_at	89822	KCNK17	potassium channel, two pore domain subfamily K, member 17	-2.22660778	0.00010332
225059_at	57085	AGTRAP	angiotensin II receptor-associated protein	1.46001831	0.00010338
228343_at	5452	POU2F2	POU class 2 homeobox 2	2.14062539	0.00010433
228486_at	23446	SLC44A1	solute carrier family 44 (choline transporter), member 1	2.73059175	0.0001069
225373_at	64115	C10orf54	chromosome 10 open reading frame 54	1.73214475	0.00010989
204799_at	9889	ZBED4	zinc finger, BED-type containing 4	-1.31455094	0.00011113
226773_at	152926	PPM1K	protein phosphatase, Mg2+/Mn2+ dependent, 1K	-0.95067611	0.00011117
225646_at	1075	CTSC	cathepsin C	-1.38666415	0.00011139
203300_x_at	8905	AP1S2	adaptor-related protein complex 1, sigma 2 subunit	2.47830096	0.00011148
223305_at	51259	TMEM216	transmembrane protein 216	-0.9438131	0.00011163
1569091_at	NA	NA	NA	-1.46537565	0.00011178
234985_at	143458	LDLRAD3	low density lipoprotein receptor class A domain containing 3	2.6402031	0.00011295
204614_at	5055	SERPINB2	serpin peptidase inhibitor, clade B (ovalbumin), member 2	4.54168349	0.00011312
242110_at	NA	NA	NA	-3.34436496	0.00011368
226016_at	961	CD47	CD47 molecule	-1.3604667	0.00011368
210038_at	5588	PRKCQ	protein kinase C, theta	-1.77256744	0.00011419
227046_at	201266	SLC39A11	solute carrier family 39, member 11	-1.77758865	0.00011497
232724_at	64231	MS4A6A	membrane-spanning 4-domains, subfamily A, member 6A	2.56364123	0.00011514
222920_s_at	9840	TESPA1	thymocyte expressed, positive selection associated 1	-2.21686816	0.00011584
202304_at	22862	FNDC3A	fibronectin type III domain containing 3A	-1.26067535	0.00011623
208092_s_at	81553	FAM49A	family with sequence similarity 49, member A	1.78933421	0.00011698
239598_s_at	54947	LPCAT2	lysophosphatidylcholine acyltransferase 2	-1.59159367	0.00011703
212747_at	23294	ANKS1A	ankyrin repeat and sterile alpha motif domain containing 1A	-1.32795155	0.00011838
242669_at	51569	UFM1	ubiquitin-fold modifier 1	-1.24289356	0.00011846
232338_at	170959	ZNF431	zinc finger protein 431	-2.2020875	0.00011848
221841_s_at	9314	KLF4	Kruppel-like factor 4 (gut)	3.2521745	0.00011852
231234_at	1075	CTSC	cathepsin C	-1.30294405	0.00011878
219183_s_at	27128	CYTH4	cytohesin 4	1.57922835	0.00011988
230894_s_at	NA	NA	NA	-2.01788951	0.00012125
201825_s_at	51097	SCCPDH	saccharopine dehydrogenase (putative)	-1.42201499	0.00012181
225372_at	64115	C10orf54	chromosome 10 open reading frame 54	2.14565916	0.00012232
218880_at	2355	FOSL2	FOS-like antigen 2	2.01456513	0.00012241
1559282_at	NA	NA	NA	-1.80522669	0.00012384
215116_s_at	1759	DNM1	dynamin 1	-1.97562017	0.00012574
208407_s_at	1500 | 10052	CTNND1 | TMX2-CTNN	catenin (cadherin-associated protein), delta 1 | TMX2-CTNND1 readthrough (NMD ca	-1.99354314	0.0001264
243808_at	NA	NA	NA	-2.24216091	0.00012699
220694_at	29065	ASAP1-IT1	ASAP1 intronic transcript 1	1.95127065	0.00012743
232891_at	128646	SIRPD	signal-regulatory protein delta	1.01685816	0.00012948
230158_at	283417	DPY19L2	dpy-19-like 2 (C. elegans)	-2.70428942	0.00012961
205098_at	1230	CCR1	chemokine (C-C motif) receptor 1	2.59129318	0.00012963
1556352_at	NA	NA	NA	1.81855085	0.00013129
202862_at	2184	FAH	fumarylacetoacetate hydrolase (fumarylacetoacetase)	-1.61881685	0.00013158
223401_at	56985	ADPRM	ADP-ribose/CDP-alcohol diphosphatase, manganese-dependent	-0.87908434	0.00013406
215193_x_at	3123 | 3119 |	HLA-DRB1 | HLA-DQB	major histocompatibility complex, class II, DR beta 1 | major histocompatibility comp	2.85718803	0.00013418
203955_at	9858	PPP1R26	protein phosphatase 1, regulatory subunit 26	-1.27853979	0.0001343
203688_at	5311	PKD2	polycystic kidney disease 2 (autosomal dominant)	-1.61100518	0.00013459
207550_at	4352	MPL	MPL proto-oncogene, thrombopoietin receptor	-2.5960176	0.000136
36553_at	8623	ASMTL	acetylserotonin O-methyltransferase-like	-1.35119444	0.00013723
213261_at	9881	TRANK1	tetratricopeptide repeat and ankyrin repeat containing 1	-1.30645609	0.00013769
224574_at	124944 | 100	C17orf49 | RNASEK-C	chromosome 17 open reading frame 49 | RNASEK-C17orf49 readthrough	-1.07443575	0.00013872
217728_at	6277	S100A6	S100 calcium binding protein A6	1.77986344	0.00013926
205322_s_at	4520	MTF1	metal-regulatory transcription factor 1	0.93714535	0.00013987
239529_at	140947 | 4971	DCANP1 | TIFAB	dendritic cell-associated nuclear protein | TRAF-interacting protein with forkhead-ass	2.94060586	0.00014051
1565598_at	NA	NA	NA	2.24659428	0.00014252
224301_x_at	55766	H2AFJ	H2A histone family, member J	1.86249313	0.00014283
228628_at	653464	SRGAP2C	SLIT-ROBO Rho GTPase activating protein 2C	2.65703421	0.00014297
200953_s_at	894	CCND2	cyclin D2	-2.59898144	0.00014331
230381_at	440712 | 1019	C1orf186 | LOC10192	chromosome 1 open reading frame 186 | uncharacterized LOC101929219	-1.71939648	0.00014407
204150_at	23166	STAB1	stabilin 1	-3.71794622	0.00014461
202933_s_at	7525	YES1	YES proto-oncogene 1, Src family tyrosine kinase	-2.49538568	0.00014468
201564_s_at	6624	FSCN1	fascin actin-bundling protein 1	-1.95246318	0.00014526
204116_at	3561	IL2RG	interleukin 2 receptor, gamma	-1.64457103	0.00014759
227837_at	NA	NA	NA	-1.26785825	0.0001483
224796_at	50807	ASAP1	ArfGAP with SH3 domain, ankyrin repeat and PH domain 1	1.4382194	0.0001483
1570441_at	63908	NAPB	N-ethylmaleimide-sensitive factor attachment protein, beta	-1.8293415	0.0001488
218324_s_at	65244	SPATS2	spermatogenesis associated, serine-rich 2	-1.16544012	0.0001528
202053_s_at	224	ALDH3A2	aldehyde dehydrogenase 3 family, member A2	1.67195506	0.00015727
225207_at	5166	PDK4	pyruvate dehydrogenase kinase, isozyme 4	2.38993386	0.00016039
243579_at	124540	MSI2	musashi RNA-binding protein 2	-1.7841415	0.00016315
225245_x_at	55766	H2AFJ	H2A histone family, member J	1.80312023	0.00016317
206295_at	3606	IL18	interleukin 18	-1.92090537	0.00016342
91703_at	254102	EHBP1L1	EH domain binding protein 1-like 1	1.19409376	0.00016503
228285_at	122402	TDRD9	tudor domain containing 9	-3.22130819	0.00016518
205859_at	9450	LY86	lymphocyte antigen 86	2.95372919	0.00016632
213361_at	23424	TDRD7	tudor domain containing 7	1.77396873	0.00016812
210166_at	7100	TLR5	toll-like receptor 5	2.38686684	0.00016821
218081_at	54976	C20orf27	chromosome 20 open reading frame 27	1.15586494	0.00016986

236561_at	7046	TGFBR1	transforming growth factor, beta receptor 1	-1.34370528	0.00017036
211031_s_at	7461	CLIP2	CAP-GLY domain containing linker protein 2	-1.92972368	0.00017153
205398_s_at	4088	SMAD3	SMAD family member 3	1.0904246	0.00017167
228024_at	137492	VPS37A	vacuolar protein sorting 37 homolog A (S. cerevisiae)	-1.18965653	0.00017279
215767_at	91752	ZNF804A	zinc finger protein 804A	-2.42556309	0.00017419
209949_at	4688	NCF2	neutrophil cytosolic factor 2	3.52471486	0.00017593
208306_x_at	3126 | 3123	HLA-DRB4 | HLA-DRB1	major histocompatibility complex, class II, DR beta 4 | major histocompatibility comp	2.41295284	0.00017752
208826_x_at	3094	HINT1	histidine triad nucleotide binding protein 1	-1.30598143	0.00017764
201565_s_at	3398	ID2	inhibitor of DNA binding 2, dominant negative helix-loop-helix protein	2.97970942	0.00018151
225546_at	29904 | 10193	EEF2K | LOC10193012	eukaryotic elongation factor 2 kinase	-1.14128713	0.00018319
239879_at	284998	LINC01114	long intergenic non-protein coding RNA 1114	-1.96467267	0.00018413
216388_s_at	1241	LTB4R	leukotriene B4 receptor	1.21338962	0.00018417
202732_at	11142	PKIG	protein kinase (cAMP-dependent, catalytic) inhibitor gamma	-1.67918418	0.00018603
210210_at	9019	MPZL1	myelin protein zero-like 1	-1.12584461	0.00018609
211612_s_at	3597	IL13RA1	interleukin 13 receptor, alpha 1	2.56737337	0.00018701
221616_s_at	51616	TAF9B	TAF9B RNA polymerase II, TATA box binding protein (TBP)-associated factor, 31kDa	-1.03359658	0.00018711
203965_at	10868	USP20	ubiquitin specific peptidase 20	-1.4563869	0.00018717
205594_at	22834	ZNF652	zinc finger protein 652	-1.5452682	0.00018749
1554408_a_at	7083	TK1	thymidine kinase 1, soluble	1.14470964	0.00018907
221515_s_at	51451	LCMT1	leucine carboxyl methyltransferase 1	-1.11694542	0.00018923
218499_at	51765	STK26	serine/threonine protein kinase 26	-1.27161445	0.00018972
202932_at	7525	YES1	YES proto-oncogene 1, Src family tyrosine kinase	-2.42291328	0.00019065
213269_at	57209	ZNF248	zinc finger protein 248	-1.35771174	0.00019095
218667_at	64219	PJA1	praja ring finger 1, E3 ubiquitin protein ligase	-0.85299488	0.00019098
209409_at	2887	GRB10	growth factor receptor-bound protein 10	-2.44902055	0.0001912
203035_s_at	10401	PIAS3	protein inhibitor of activated STAT, 3	-1.10458575	0.00019143
202804_at	4363	ABCC1	ATP-binding cassette, sub-family C (CFTR/MRP), member 1	-1.70946869	0.00019379
229838_at	4925	NUCB2	nucleobindin 2	-1.83360643	0.00019451
225499_at	57186	RALGAPA2	Ral GTPase activating protein, alpha subunit 2 (catalytic)	-0.99157558	0.00019617
206480_at	4056	LTC4S	leukotriene C4 synthase	-1.3990741	0.00019933
204352_at	7188	TRAF5	TNF receptor-associated factor 5	-1.99652039	0.00020458
225032_at	64778	FNDC3B	fibronectin type III domain containing 3B	-1.48327441	0.00020474
212316_at	23225	NUP210	nucleoporin 210kDa	1.33461542	0.00020557
208749_x_at	10211	FLOT1	flotillin 1	1.29008	0.00020747
224959_at	1836	SLC26A2	solute carrier family 26 (anion exchanger), member 2	-1.39178629	0.00021191
217362_x_at	3128	HLA-DRB6	major histocompatibility complex, class II, DR beta 6 (pseudogene)	1.9251957	0.00021198
227134_at	84958	SYTL1	synaptotagmin-like 1	-2.18704981	0.00021206
231188_at	54993	ZSCAN2	zinc finger and SCAN domain containing 2	-1.33971032	0.00021248
233214_at	NA	NA	NA	-1.28668646	0.00021696
210962_s_at	10142	AKAP9	A kinase (PRKA) anchor protein 9	-0.93390145	0.00021812
228570_at	121551	BTBD11	BTB (POZ) domain containing 11	-2.14972981	0.00021822
206950_at	6335	SCN9A	sodium channel, voltage gated, type IX alpha subunit	-2.50070138	0.00022003
202957_at	3059	HCLS1	hematopoietic cell-specific Lyn substrate 1	1.03814446	0.00022009
1564031_a_at	285613	RELL2	RELT-like 2	1.00858712	0.00022013
226473_at	84733	CBX2	chromobox homolog 2	-1.46470384	0.00022043
210377_at	6296	ACSM3	acyl-CoA synthetase medium-chain family member 3	-2.00870966	0.00022259
206361_at	11251	PTGDR2	prostaglandin D2 receptor 2	-1.0967412	0.00022546
205142_x_at	215	ABCD1	ATP-binding cassette, sub-family D (ALD), member 1	0.79840214	0.00022818
219053_s_at	55048	VPS37C	vacuolar protein sorting 37 homolog C (S. cerevisiae)	1.14750654	0.00022956
208858_s_at	23344	ESYT1	extended synaptotagmin-like protein 1	-1.12541315	0.00022996
212956_at	23158	TBC1D9	TBC1 domain family, member 9 (with GRAM domain)	2.17282732	0.00023039
204184_s_at	157	ADRBK2	adrenergic, beta, receptor kinase 2	1.28839179	0.00023168
235500_at	3183	HNRNPC	heterogeneous nuclear ribonucleoprotein C (C1/C2)	-1.41192484	0.00023438
223477_s_at	91574	C12orf65	chromosome 12 open reading frame 65	-1.11822405	0.00023453
205801_s_at	25780	RASGRP3	RAS guanyl releasing protein 3 (calcium and DAG-regulated)	-2.23649145	0.00023484
1553906_s_at	221472	FGD2	FYVE, RhoGEF and PH domain containing 2	1.45149776	0.00023726
210784_x_at	11025	LILRB3	leukocyte immunoglobulin-like receptor, subfamily B (with TM and ITIM domains), me	1.73562815	0.00023915
1560060_s_at	55048	VPS37C	vacuolar protein sorting 37 homolog C (S. cerevisiae)	1.17158395	0.00023939
1555846_a_at	NA	NA	NA	-1.03113501	0.00024031
226912_at	254887	ZDHHC23	zinc finger, DHHC-type containing 23	-1.81228063	0.00024222
227564_at	138050	HGSNAT	heparan-alpha-glucosaminide N-acetyltransferase	-0.92228448	0.00024258
213375_s_at	90634	N4BP2L1	NEDD4 binding protein 2-like 1	-1.58912101	0.00024378
240798_at	NA	NA	NA	-1.40011838	0.00024462
232001_at	439949	PRKCQ-AS1	PRKCQ antisense RNA 1	-1.58145071	0.00024696
221039_s_at	50807	ASAP1	ArfGAP with SH3 domain, ankyrin repeat and PH domain 1	1.68532239	0.00024699
211776_s_at	23136	EPB41L3	erythrocyte membrane protein band 4.1-like 3	1.62226943	0.00025147
236829_at	NA	NA	NA	-1.07781151	0.00025159
201670_s_at	4082	MARCKS	myristoylated alanine-rich protein kinase C substrate	2.87738237	0.00025351
240094_at	NA	NA	NA	-1.71221558	0.00025442
219696_at	163486	DENND1B	DENN/MADD domain containing 1B	-1.1000923	0.00025499
239448_at	NA	NA	NA	1.71762265	0.00025514
204639_at	100	ADA	adenosine deaminase	-1.67784375	0.00025822
216813_at	NA	NA	NA	-1.90398957	0.00025921
222526_at	54815	GATAD2A	GATA zinc finger domain containing 2A	-0.76406215	0.00025975
225320_at	90550	MCU	mitochondrial calcium uniporter	1.10289317	0.00026132
203920_at	10062	NR1H3	nuclear receptor subfamily 1, group H, member 3	-1.40612002	0.00026147
236592_at	NA	NA	NA	2.01275604	0.00026412
221944_at	645644	FLJ42627	uncharacterized LOC645644	-1.11561634	0.00026831
230170_at	5008	OSM	oncostatin M	-1.46529897	0.00026832
1562948_at	NA	NA	NA	-2.03490115	0.00026941
202996_at	57804	POLD4	polymerase (DNA-directed), delta 4, accessory subunit	0.91665227	0.00026949
240499_at	NA	NA	NA	-1.50250655	0.00027095
209705_at	22823	MTF2	metal response element binding transcription factor 2	-1.01042466	0.00027122
222670_s_at	9935	MAFB	v-maf avian musculoaponeurotic fibrosarcoma oncogene homolog B	2.32052113	0.00027151
210663_s_at	8942	KYNU	kynureninase	2.8059985	0.00027255
213645_at	55556	ENOSF1	enolase superfamily member 1	-1.94904525	0.00027294
203140_at	604	BCL6	B-cell CLL/lymphoma 6	2.18040048	0.00027573
200670_at	7494	XBP1	X-box binding protein 1	-1.12485271	0.00027688
223382_s_at	84937	ZNRF1	zinc and ring finger 1, E3 ubiquitin protein ligase	-1.44433636	0.00028198
229937_x_at	NA	NA	NA	2.49842202	0.0002857
1566243_at	NA	NA	NA	-1.72026208	0.0002859
228056_s_at	256236	NAPSB	napsin B aspartic peptidase, pseudogene	2.32316591	0.00028988
213358_at	23255	MTCL1	microtubule crosslinking factor 1	-1.94515064	0.00029204
222451_s_at	51114	ZDHHC9	zinc finger, DHHC-type containing 9	-1.24108065	0.00029326
224576_at	57222	ERGIC1	endoplasmic reticulum-golgi intermediate compartment (ERGIC) 1	-1.18771354	0.00029351
211563_s_at	8725	URI1	URI1, prefoldin-like chaperone	-1.21918643	0.0002954
201486_at	5955	RCN2	reticulocalbin 2, EF-hand calcium binding domain	-1.07497504	0.00029982
219593_at	51296	SLC15A3	solute carrier family 15 (oligopeptide transporter), member 3	1.47268062	0.00030194
243016_at	NA	NA	NA	-2.56807547	0.00030409
1568597_at	646762	LOC646762	uncharacterized LOC646762	-1.76597706	0.0003041
228485_s_at	23446	SLC44A1	solute carrier family 44 (choline transporter), member 1	1.91610196	0.00030502
211336_x_at	10859	LILRB1	leukocyte immunoglobulin-like receptor, subfamily B (with TM and ITIM domains), me	1.85009796	0.00030658
223282_at	10194	TSHZ1	teashirt zinc finger homeobox 1	-1.29071813	0.0003069
221004_s_at	81618	ITM2C	integral membrane protein 2C	-1.96945772	0.00030713

1559131_a_at	101928047	LOC101928047	uncharacterized LOC101928047	-2.16475346	0.00030765
228055_at	256236	NAPSB	napsin B aspartic peptidase, pseudogene	2.96312687	0.00030944
212279_at	27346	TMEM97	transmembrane protein 97	1.60558922	0.00030962
211864_s_at	26509	MYOF	myoferlin	2.46979926	0.00030992
221042_s_at	79789	CLMN	calmin (calponin-like, transmembrane)	-1.84372482	0.00031055
64418_at	11276	SYNRG	synergin, gamma	-0.85958138	0.0003106
1555728_a_at	51338	MS4A4A	membrane-spanning 4-domains, subfamily A, member 4A	3.38653576	0.000313
219371_s_at	10365	KLF2	Kruppel-like factor 2	1.85187889	0.00031953
229686_at	286530	P2RY8	purinergic receptor P2Y, G-protein coupled, 8	-1.48609289	0.00032145
213329_at	23380 | 64713	SRGAP2 | SRGAP2B |	SLIT-ROBO Rho GTPase activating protein 2 | SLIT-ROBO Rho GTPase activating prote	1.15001542	0.00032192
209143_s_at	1207	CLNS1A	chloride channel, nucleotide-sensitive, 1A	-1.1272778	0.00032574
224674_at	80727	TTYH3	tweety family member 3	0.94662004	0.0003273
223136_at	51390	AIG1	androgen-induced 1	-3.19196053	0.00032902
238908_at	NA	NA	NA	-1.20468747	0.00033034
215435_at	NA	NA	NA	1.69039162	0.00033115
232406_at	NA	NA	NA	-1.52989624	0.00033332
210116_at	4068	SH2D1A	SH2 domain containing 1A	-2.50649587	0.00033622
213666_at	23157	SEPT6	septin 6	-1.64518856	0.00033639
209422_at	51230	PHF20	PHD finger protein 20	-1.31034681	0.00034105
217691_x_at	9123 | 102465	SLC16A3 | MIR6787	solute carrier family 16 (monocarboxylate transporter), member 3 | microRNA 6787	0.90156315	0.00034139
212831_at	1955	MEGF9	multiple EGF-like-domains 9	1.61991052	0.00034335
212686_at	57460	PPM1H	protein phosphatase, Mg2+/Mn2+ dependent, 1H	-2.00263254	0.00034367
202159_at	2193	FARSA	phenylalanyl-tRNA synthetase, alpha subunit	-0.85180762	0.00034391
230264_s_at	8905	AP1S2	adaptor-related protein complex 1, sigma 2 subunit	2.27451798	0.00034668
209865_at	23443	SLC35A3	solute carrier family 35 (UDP-N-acetylglucosamine (UDP-GlcNAc) transporter), memb	-1.33088723	0.00034685
202842_s_at	4189	DNAJB9	DnaJ (Hsp40) homolog, subfamily B, member 9	-1.23580254	0.00034907
222876_s_at	55803	ADAP2	ArfGAP with dual PH domains 2	1.66371519	0.0003491
202349_at	1861	TOR1A	torsin family 1, member A (torsin A)	-0.93437683	0.00034971
210645_s_at	7267 | 286495	TTC3 | TTC3P1	tetratricopeptide repeat domain 3 | tetratricopeptide repeat domain 3 pseudogene 1	-1.00584915	0.00035238
229766_at	353274	ZNF445	zinc finger protein 445	-1.01032881	0.00035544
226820_at	149076	ZNF362	zinc finger protein 362	-0.70903701	0.00036177
1556202_at	23380	SRGAP2	SLIT-ROBO Rho GTPase activating protein 2	2.65882685	0.00036644
215201_at	85021	REPS1	RALBP1 associated Eps domain containing 1	-1.33170259	0.00036997
243003_at	NA	NA	NA	-1.56814889	0.00037085
227224_at	55103	RALGPS2	Ral GEF with PH domain and SH3 binding motif 2	-1.27492725	0.00037207
221937_at	11276	SYNRG	synergin, gamma	-0.76480239	0.00037537
210225_x_at	11025	LILRB3	leukocyte immunoglobulin-like receptor, subfamily B (with TM and ITIM domains), me	1.5580442	0.0003758
203050_at	7158	TP53BP1	tumor protein p53 binding protein 1	-1.07224683	0.00037839
212200_at	23141	ANKLE2	ankyrin repeat and LEM domain containing 2	-1.24173329	0.00037951
225747_at	93058	COQ10A	coenzyme Q10 homolog A (S. cerevisiae)	-0.98326435	0.00038341
215577_at	NA	NA	NA	-0.99264894	0.00039163
204385_at	8942	KYNU	kynureninase	1.89509977	0.0003917
244697_at	NA	NA	NA	-1.22775834	0.00039276
225081_s_at	55536	CDCA7L	cell division cycle associated 7-like	-1.45218835	0.000394
207067_s_at	3067	HDC	histidine decarboxylase	-2.42761157	0.00039612
204858_s_at	1890	TYMP	thymidine phosphorylase	1.46292697	0.00039618
213779_at	129080	EMID1	EMI domain containing 1	-1.78212184	0.00039829
203685_at	596	BCL2	B-cell CLL/lymphoma 2	-1.67656725	0.0004048
237600_at	NA	NA	NA	-1.38658697	0.0004064
226888_at	53944	CSNK1G1	casein kinase 1, gamma 1	-0.93753971	0.00040707
242029_at	NA	NA	NA	-2.56381756	0.00041063
212202_s_at	25963	TMEM87A	transmembrane protein 87A	-1.19849673	0.00041249
201798_s_at	26509	MYOF	myoferlin	2.77954911	0.00041572
212360_at	271	AMPD2	adenosine monophosphate deaminase 2	-0.85405809	0.00041714
1559739_at	56994	CHPT1	choline phosphotransferase 1	-1.36056653	0.00041747
207292_s_at	5598	MAPK7	mitogen-activated protein kinase 7	0.85721742	0.00041896
222288_at	NA	NA	NA	-2.57955398	0.00042561
233037_at	NA	NA	NA	-1.55704208	0.00042708
1552733_at	122773	KLHDC1	kelch domain containing 1	-1.392531	0.00042728
239744_at	NA	NA	NA	-1.89962036	0.0004303
229861_at	117584 | 3980	RFFL | LIG3	ring finger and FYVE-like domain containing E3 ubiquitin protein ligase | ligase III, DN	-1.10910272	0.00043439
203188_at	11041	B4GAT1	beta-1,4-glucuronyltransferase 1	-1.08904386	0.00043451
212959_s_at	79158	GNPTAB	N-acetylglucosamine-1-phosphate transferase, alpha and beta subunits	-1.49886254	0.00043728
219202_at	79651	RHBDF2	rhomboid 5 homolog 2 (Drosophila)	1.34224598	0.00043963
1557738_at	NA	NA	NA	-1.02690788	0.00044279
211065_x_at	5211	PFKL	phosphofructokinase, liver	0.7968323	0.00044369
228280_at	92092	ZC3HAV1L	zinc finger CCCH-type, antiviral 1-like	-1.43585683	0.00044499
209975_at	1571	CYP2E1	cytochrome P450, family 2, subfamily E, polypeptide 1	-1.95229271	0.00044827
234269_at	NA	NA	NA	-2.62819664	0.00045079
214058_at	4610	MYCL	v-myc avian myelocytomatosis viral oncogene lung carcinoma derived homolog	1.45994035	0.00045097
206206_at	4064	CD180	CD180 molecule	2.12624902	0.00045375
225098_at	10152	ABI2	abl-interactor 2	-1.30355704	0.00045435
234041_at	NA	NA	NA	-1.04440982	0.00045731
213839_at	79789	CLMN	calmin (calponin-like, transmembrane)	-1.87162772	0.00045877
221591_s_at	54478	FAM64A	family with sequence similarity 64, member A	1.38417072	0.00045898
227802_at	22902	RUFY3	RUN and FYVE domain containing 3	-1.39640665	0.00045904
210140_at	8530	CST7	cystatin F (leukocystatin)	-3.06028886	0.00045928
218618_s_at	64778	FNDC3B	fibronectin type III domain containing 3B	-1.85758302	0.00046213
203163_at	10300	KATNB1	katanin p80 (WD repeat containing) subunit B 1	-1.15534164	0.00046272
1566901_at	7050	TGIF1	TGFB-induced factor homeobox 1	-1.80901781	0.00046306
204393_s_at	55	ACPP	acid phosphatase, prostate	1.44852429	0.00046416
223538_at	8293 | 728492	SERF1A | SERF1B	small EDRK-rich factor 1A (telomeric) | small EDRK-rich factor 1B (centromeric)	-1.77469882	0.00046508
225440_at	56894	AGPAT3	1-acylglycerol-3-phosphate O-acyltransferase 3	1.27870945	0.00046658
235985_at	NA	NA	NA	-1.13678021	0.0004666
210999_s_at	2887	GRB10	growth factor receptor-bound protein 10	-1.80816547	0.00047036
213939_s_at	22902	RUFY3	RUN and FYVE domain containing 3	-1.9973875	0.00047101
204751_x_at	1824	DSC2	desmocollin 2	-2.17081388	0.00047428
202943_s_at	4668	NAGA	N-acetylgalactosaminidase, alpha-	1.3145789	0.00047665
230713_at	NA	NA	NA	-1.54231046	0.00047677
212062_at	10079	ATP9A	ATPase, class II, type 9A	-2.38022595	0.00047829
1560271_at	NA	NA	NA	-1.20304144	0.00048242
241788_x_at	NA	NA	NA	-1.19014694	0.00048356
209480_at	3119	HLA-DQB1	major histocompatibility complex, class II, DQ beta 1	3.75430043	0.00048436
227908_at	57465	TBC1D24	TBC1 domain family, member 24	-1.18903516	0.00048633
1567628_at	972	CD74	CD74 molecule, major histocompatibility complex, class II invariant chain	1.62878222	0.00048983
244696_at	NA	NA	NA	2.03672298	0.00049095
212776_s_at	23363	OBSL1	obscurin-like 1	-1.91225979	0.00049152
211656_x_at	3119	HLA-DQB1	major histocompatibility complex, class II, DQ beta 1	1.98020777	0.00049259
212646_at	23180	RFTN1	raftlin, lipid raft linker 1	-1.41295901	0.00049287
1555841_at	91283	MSANTD3	Myb/SANT-like DNA-binding domain containing 3	-0.89759317	0.00049629
223424_s_at	7589	ZSCAN21	zinc finger and SCAN domain containing 21	-1.09680576	0.00050351
207270_x_at	10871	CD300C	CD300c molecule	1.24039551	0.00050372
223306_at	84650	EBPL	emopamil binding protein-like	-1.07522676	0.00050487
213947_s_at	23225	NUP210	nucleoporin 210kDa	1.18175853	0.00051328

214579_at	57185	NIPAL3	NIPA-like domain containing 3	-0.86700246	0.0005148
202878_s_at	22918	CD93	CD93 molecule	2.77668449	0.00051733
209795_at	969	CD69	CD69 molecule	-2.90432931	0.00051979
213549_at	118987	PDZD8	PDZ domain containing 8	-1.91149492	0.00052371
223383_at	84937	ZNRF1	zinc and ring finger 1, E3 ubiquitin protein ligase	-1.27153962	0.00052478
204951_at	399	RHOH	ras homolog family member H	-1.27369991	0.00053161
224097_s_at	50848	F11R	F11 receptor	-0.99089401	0.00053207
224790_at	50807	ASAP1	ArfGAP with SH3 domain, ankyrin repeat and PH domain 1	1.44092396	0.00053796
208702_x_at	334	APLP2	amyloid beta (A4) precursor-like protein 2	1.56166716	0.00053813
218301_at	57140	RNPEPL1	arginyl aminopeptidase (aminopeptidase B)-like 1	0.81831604	0.00053911
1558409_at	NA	NA	NA	-1.11200538	0.00053974
233271_at	NA	NA	NA	-2.81299927	0.00054078
236153_at	NA	NA	NA	-1.30501124	0.00054158
227265_at	10875	FGL2	fibrinogen-like 2	3.43984336	0.00054349
205863_at	6283	S100A12	S100 calcium binding protein A12	4.66103031	0.00054684
219221_at	253461	ZBTB38	zinc finger and BTB domain containing 38	1.67967488	0.00055328
1560754_at	112616	CMTM7	CKLF-like MARVEL transmembrane domain containing 7	-1.33077912	0.00055467
232472_at	NA	NA	NA	-2.3595673	0.00055567
226245_at	284252	KCTD1	potassium channel tetramerization domain containing 1	-1.70929746	0.00055969
202626_s_at	4067	LYN	LYN proto-oncogene, Src family tyrosine kinase	1.4559367	0.00056027
220952_s_at	54477	PLEKHA5	pleckstrin homology domain containing, family A member 5	-1.90018634	0.00056345
242414_at	23475	QPRT	quinolinate phosphoribosyltransferase	-1.2828883	0.000566
229021_at	55784	MCTP2	multiple C2 domains, transmembrane 2	-1.33132286	0.0005672
202800_at	6507	SLC1A3	solute carrier family 1 (glial high affinity glutamate transporter), member 3	1.90627955	0.00056919
226546_at	100506844	LOC100506844	uncharacterized LOC100506844	-1.09720574	0.00056952
239660_at	57186	RALGAPA2	Ral GTPase activating protein, alpha subunit 2 (catalytic)	-1.25759474	0.00056998
227749_at	5452	POU2F2	POU class 2 homeobox 2	1.37986932	0.00057671
223565_at	51237	MZB1	marginal zone B and B1 cell-specific protein	-1.71362342	0.00057902
206207_at	1178	CLC	Charcot-Leyden crystal galectin	-3.68701494	0.00058177
206335_at	2588	GALNS	galactosamine (N-acetyl)-6-sulfatase	-0.91578373	0.00058226
226455_at	148327	CREB3L4	cAMP responsive element binding protein 3-like 4	-1.1393423	0.00058265
208862_s_at	1500 | 10052	CTNND1 | TMX2-CTNN	catenin (cadherin-associated protein), delta 1 | TMX2-CTNND1 readthrough (NMD ca	-1.0890363	0.00058335
203897_at	57149	LYRM1	LYR motif containing 1	-1.86244413	0.00058421
244663_at	170959	ZNF431	zinc finger protein 431	-1.70634428	0.0005887
207130_at	23613	ZMYND8	zinc finger, MYND-type containing 8	-1.47394198	0.00058891
207474_at	54861	SNRK	SNF related kinase	-1.4396665	0.00059009
225703_at	57666	FBRSL1	fibrosin-like 1	-1.43674405	0.00059254
217897_at	53826	FXYD6	FXYD domain containing ion transport regulator 6	1.14917568	0.00059265
218641_at	65998	C11orf95	chromosome 11 open reading frame 95	-0.99700735	0.00059605
230653_at	102724356	LOC102724356	uncharacterized LOC102724356	-1.33249867	0.00059624
1555793_a_at	284406	ZFP82	ZFP82 zinc finger protein	-1.13159273	0.00059983
227353_at	147138	TMC8	transmembrane channel-like 8	1.10986288	0.00060123
205768_s_at	11001	SLC27A2	solute carrier family 27 (fatty acid transporter), member 2	-2.01188749	0.00060232
227279_at	85012	TCEAL3	transcription elongation factor A (SII)-like 3	-1.27190396	0.00060507
205505_at	2650	GCNT1	glucosaminyl (N-acetyl) transferase 1, core 2	-1.2773062	0.00060621
214173_x_at	8725	URI1	URI1, prefoldin-like chaperone	-0.78295064	0.00060697
239384_at	6426	SRSF1	serine/arginine-rich splicing factor 1	1.36775212	0.00060896
203271_s_at	9094	UNC119	unc-119 homolog (C. elegans)	0.76917434	0.00061036
234394_at	7678	ZNF124	zinc finger protein 124	-2.03507961	0.00061316
217788_s_at	2590	GALNT2	polypeptide N-acetylgalactosaminyltransferase 2	-1.02788074	0.00061717
203236_s_at	3965	LGALS9	lectin, galactoside-binding, soluble, 9	1.48407038	0.0006172
228032_s_at	163486	DENND1B	DENN/MADD domain containing 1B	-1.10559342	0.00061814
232527_at	100507062	PSMD6-AS2	PSMD6 antisense RNA 2	-0.97759642	0.00061979
209344_at	7171	TPM4	tropomyosin 4	-1.9574653	0.0006206
240446_at	NA	NA	NA	-1.10263028	0.00062608
227711_at	121355	GTSF1	gametocyte specific factor 1	-2.81652982	0.00062664
1558624_at	NA	NA	NA	-1.57702612	0.00063026
214203_s_at	5625 | 10272	PRODH | LOC1027247	proline dehydrogenase (oxidase) 1 | proline dehydrogenase 1, mitochondrial	-1.52796342	0.00063042
201724_s_at	2589	GALNT1	polypeptide N-acetylgalactosaminyltransferase 1	-1.0371055	0.00063192
225246_at	57620	STIM2	stromal interaction molecule 2	1.26925008	0.00063501
225722_at	7371 | 10050	UCK2 | MIR3658	uridine-cytidine kinase 2 | microRNA 3658	-0.90972316	0.00063505
218458_at	64396 | 6439	GMCL1P1 | GMCL1	germ cell-less, spermatogenesis associated 1 pseudogene 1 | germ cell-less, spermat	-1.26785443	0.00063534
217911_s_at	9531	BAG3	BCL2-associated athanogene 3	1.86833229	0.00063835
221286_s_at	51237	MZB1	marginal zone B and B1 cell-specific protein	-1.75084048	0.00064658
224722_at	57534	MIB1	mindbomb E3 ubiquitin protein ligase 1	-1.34214412	0.00065281
1552867_at	NA	NA	NA	-2.4328957	0.00065352
242725_at	NA	NA	NA	-1.29459675	0.0006541
215046_at	151050	KANSL1L	KAT8 regulatory NSL complex subunit 1-like	-1.61972732	0.0006584
224492_s_at	199692	ZNF627	zinc finger protein 627	-1.36982516	0.00065845
202948_at	3554	IL1R1	interleukin 1 receptor, type I	-2.56042682	0.00066055
243816_at	7621	ZNF70	zinc finger protein 70	-1.89214789	0.00066164
1556203_a_at	23380	SRGAP2	SLIT-ROBO Rho GTPase activating protein 2	2.74276811	0.00066431
232466_at	8451	CUL4A	cullin 4A	-1.52983166	0.00066835
213537_at	3113	HLA-DPA1	major histocompatibility complex, class II, DP alpha 1	2.56725827	0.00067473
201668_x_at	4082	MARCKS	myristoylated alanine-rich protein kinase C substrate	1.67911654	0.00067532
239735_at	NA	NA	NA	-1.42054566	0.00067534
209823_x_at	3119	HLA-DQB1	major histocompatibility complex, class II, DQ beta 1	2.3446932	0.00067575
213005_s_at	23189	KANK1	KN motif and ankyrin repeat domains 1	-1.48497695	0.00067775
201057_s_at	2804	GOLGB1	golgin B1	-0.64120223	0.00068239
213607_x_at	65220	NADK	NAD kinase	1.43456065	0.00068306
220097_s_at	54868	TMEM104	transmembrane protein 104	0.89161256	0.00068314
209757_s_at	4613	MYCN	v-myc avian myelocytomatosis viral oncogene neuroblastoma derived homolog	-1.86095751	0.00068735
233647_s_at	81602	CDADC1	cytidine and dCMP deaminase domain containing 1	-1.15526096	0.00069411
201566_x_at	3398	ID2	inhibitor of DNA binding 2, dominant negative helix-loop-helix protein	2.12952442	0.00069413
203704_s_at	6239	RREB1	ras responsive element binding protein 1	-1.01651435	0.00069984
233198_at	55592	GOLGA2P5	golgin A2 pseudogene 5	-1.76177485	0.00069999
222893_s_at	79871	RPAP2	RNA polymerase II associated protein 2	-0.74564591	0.00070225
206506_s_at	8464	SUPT3H	suppressor of Ty 3 homolog (S. cerevisiae)	-0.81381253	0.00070332
201531_at	7538	ZFP36	ZFP36 ring finger protein	1.37735482	0.00070465
207636_at	5276	SERPINI2	serpin peptidase inhibitor, clade I (pancpin), member 2	2.36312693	0.00070589
221802_s_at	57698	KIAA1598	KIAA1598	2.11877226	0.00070767
227716_at	91544	UBXN11	UBX domain protein 11	1.22537117	0.00071088
222688_at	55331	ACER3	alkaline ceramidase 3	1.3290823	0.00071563
214745_at	23007	PLCH1	phospholipase C, eta 1	-1.70374053	0.00071732
1555041_a_at	4668	NAGA	N-acetylgalactosaminidase, alpha-	1.23179015	0.00071779
203198_at	1025	CDK9	cyclin-dependent kinase 9	1.15745916	0.00071788
222693_at	64778	FNDC3B	fibronectin type III domain containing 3B	-1.76210843	0.00071985
212775_at	23363	OBSL1	obscurin-like 1	-2.58339068	0.00072342
212234_at	171023	ASXL1	additional sex combs like transcriptional regulator 1	-0.6608373	0.00072408
1562467_at	NA	NA	NA	-1.41649194	0.00072683
209297_at	6453	ITSN1	intersectin 1 (SH3 domain protein)	1.03960141	0.00072703
219251_s_at	55112	WDR60	WD repeat domain 60	-0.94099389	0.00073209
229655_at	25817	FAM19A5	family with sequence similarity 19 (chemokine (C-C motif)-like), member A5	-1.65052403	0.00073474
38487_at	23166	STAB1	stabilin 1	-3.03582999	0.00073705

225869_s_at	81622	UNC93B1	unc-93 homolog B1 (C. elegans)	1.48266799	0.0007389
201722_s_at	2589	GALNT1	polypeptide N-acetylgalactosaminyltransferase 1	-1.04273745	0.00074885
207734_at	54900	LAX1	lymphocyte transmembrane adaptor 1	-1.11546291	0.00074932
235706_at	1368	CPM	carboxypeptidase M	1.79767687	0.00075124
201792_at	165	AEBP1	AE binding protein 1	-1.23539065	0.00075814
202237_at	4837	NNMT	nicotinamide N-methyltransferase	-3.04552417	0.00075916
208594_x_at	79168	LILRA6	leukocyte immunoglobulin-like receptor, subfamily A (with TM domain), member 6	1.47389966	0.00076384
230033_at	352909	DNAAF3	dynein, axonemal, assembly factor 3	-1.18252698	0.00076483
201874_at	9019	MPZL1	myelin protein zero-like 1	-1.11628536	0.00076807
205771_s_at	9465	AKAP7	A kinase (PRKA) anchor protein 7	2.15852221	0.00076847
226032_at	835	CASP2	caspase 2, apoptosis-related cysteine peptidase	-0.82561257	0.00076854
202763_at	836	CASP3	caspase 3, apoptosis-related cysteine peptidase	-1.26914579	0.00076962
207224_s_at	27036	SIGLEC7	sialic acid binding Ig-like lectin 7	1.40815408	0.00077096
202972_s_at	10144	FAM13A	family with sequence similarity 13, member A	1.17518994	0.00077713
227502_at	57189	KIAA1147	KIAA1147	-1.14102733	0.00077772
201102_s_at	5211	PFKL	phosphofructokinase, liver	0.70694415	0.00078066
225441_x_at	84316	NAA38	N(alpha)-acetyltransferase 38, NatC auxiliary subunit	0.863373	0.0007809
224516_s_at	51523	CXXC5	CXXC finger protein 5	-2.11953776	0.00078445
213418_at	3310	HSPA6	heat shock 70kDa protein 6 (HSP70B')	1.45782708	0.00078647
202180_s_at	9961	MVP	major vault protein	1.33023213	0.00078765
225958_at	1911	PHC1	polyhomeotic homolog 1 (Drosophila)	-1.39640157	0.0007887
1557228_at	254102	EHBP1L1	EH domain binding protein 1-like 1	0.90995756	0.00079007
235061_at	152926	PPM1K	protein phosphatase, Mg2+/Mn2+ dependent, 1K	-1.59364876	0.0007913
212204_at	25963	TMEM87A	transmembrane protein 87A	-0.96387364	0.00079327
219952_s_at	57192	MCOLN1	mucolipin 1	1.21919694	0.00079409
212791_at	127703	C1orf216	chromosome 1 open reading frame 216	-0.88150427	0.0007997
212345_s_at	64764	CREB3L2	cAMP responsive element binding protein 3-like 2	-0.9152434	0.00080043
205627_at	978	CDA	cytidine deaminase	2.06896857	0.00080172
235670_at	8676	STX11	syntaxin 11	2.25215393	0.00080396
236957_at	157313	CDCA2	cell division cycle associated 2	1.14297547	0.00080586
226043_at	26086	GPSM1	G-protein signaling modulator 1	-1.57289478	0.00080601
222182_s_at	4848	CNOT2	CCR4-NOT transcription complex, subunit 2	-0.79212536	0.00080944
1557803_at	NA	NA	NA	2.20227862	0.00080961
221664_s_at	50848	F11R	F11 receptor	-1.53603741	0.0008112
228569_at	10914	PAPOLA	poly(A) polymerase alpha	-1.17135869	0.00082403
222116_s_at	125058	TBC1D16	TBC1 domain family, member 16	-1.71529394	0.00082923
220352_x_at	645644	FLJ42627	uncharacterized LOC645644	-1.11030201	0.00083046
224719_s_at	113246	C12orf57	chromosome 12 open reading frame 57	-1.16556018	0.00083983
207180_s_at	10553	HTATIP2	HIV-1 Tat interactive protein 2, 30kDa	1.50574235	0.00084093
1569540_at	NA	NA	NA	-1.75103739	0.00084231
220744_s_at	55764	IFT122	intraflagellar transport 122	-0.80156033	0.00084384
219412_at	23682	RAB38	RAB38, member RAS oncogene family	-1.91498022	0.00084463
212071_s_at	6711	SPTBN1	spectrin, beta, non-erythrocytic 1	-1.2010185	0.00084768
211474_s_at	5269	SERPINB6	serpin peptidase inhibitor, clade B (ovalbumin), member 6	2.43131401	0.0008554
209537_at	2135	EXTL2	exostosin-like glycosyltransferase 2	-1.18788047	0.00086138
227361_at	9953	HS3ST3B1	heparan sulfate (glucosamine) 3-O-sulfotransferase 3B1	-2.7076637	0.00086146
225764_at	2120	ETV6	ets variant 6	-1.2274051	0.00086234
208918_s_at	65220	NADK	NAD kinase	1.60073056	0.00086478
237561_x_at	NA	NA	NA	-1.07908301	0.00086739
213975_s_at	4069	LYZ	lysozyme	2.01264342	0.00086817
226017_at	112616	CMTM7	CKLF-like MARVEL transmembrane domain containing 7	-0.90916683	0.00087216
232889_at	NA	NA	NA	-1.27033752	0.0008756
223513_at	55835	CENPJ	centromere protein J	-1.48165633	0.00088391
240846_at	84105	PCBD2	pterin-4 alpha-carbinolamine dehydratase/dimerization cofactor of hepatocyte nuclea	-1.35853841	0.0008875
209448_at	10553	HTATIP2	HIV-1 Tat interactive protein 2, 30kDa	2.18707449	0.00088953
211135_x_at	11025	LILRB3	leukocyte immunoglobulin-like receptor, subfamily B (with TM and ITIM domains), me	1.38789974	0.00089198
235123_at	NA	NA	NA	-1.46673685	0.00089893
227611_at	123283	TARSL2	threonyl-tRNA synthetase-like 2	-1.16106766	0.00089947
226134_s_at	124540	MSI2	musashi RNA-binding protein 2	-2.86864884	0.00090103
225335_at	84838	ZNF496	zinc finger protein 496	-1.39186393	0.00090382
207075_at	114548	NLRP3	NLR family, pyrin domain containing 3	2.1008389	0.0009044
225060_at	84918	LRP11	low density lipoprotein receptor-related protein 11	1.52954248	0.00090606
227870_at	57722	IGDCC4	immunoglobulin superfamily, DCC subclass, member 4	-2.00132516	0.0009073
218437_s_at	54585	LZTFL1	leucine zipper transcription factor-like 1	-1.3762225	0.00091148
224162_s_at	79791	FBXO31	F-box protein 31	-0.89769277	0.0009149
207001_x_at	1831	TSC22D3	TSC22 domain family, member 3	1.34287961	0.00093171
1556034_s_at	10903	MTMR11	myotubularin related protein 11	1.93986264	0.00093288
204882_at	9938	ARHGAP25	Rho GTPase activating protein 25	-0.93536297	0.00093368
205748_s_at	55658	RNF126	ring finger protein 126	0.81025376	0.00093889
202365_at	84747	UNC119B	unc-119 homolog B (C. elegans)	-1.03258153	0.00093926
208704_x_at	334	APLP2	amyloid beta (A4) precursor-like protein 2	1.37645398	0.00094044
1560026_at	NA	NA	NA	1.93538739	0.00094378
225381_at	399959	MIR100HG	mir-100-let-7a-2 cluster host gene	-3.07263935	0.00094456
223259_at	94103	ORMDL3	ORMDL sphingolipid biosynthesis regulator 3	-0.93047996	0.00094721
221617_at	51616	TAF9B	TAF9B RNA polymerase II, TATA box binding protein (TBP)-associated factor, 31kDa	-0.7732444	0.00094977
207794_at	729230	CCR2	chemokine (C-C motif) receptor 2	3.2388098	0.00095312
221059_s_at	4166 | 23406	CHST6 | COTL1	carbohydrate (N-acetylglucosamine 6-O) sulfotransferase 6 | coactosin-like F-actin bi	1.68177706	0.00095319
235657_at	NA	NA	NA	-1.14108147	0.00095442
208810_at	65084 | 10049	TMEM135 | DNAJB6	transmembrane protein 135 | DnaJ (Hsp40) homolog, subfamily B, member 6	-1.13386287	0.00096072
244456_at	NA	NA	NA	-1.72421439	0.00096087
209360_s_at	861 | 1005064	RUNX1 | LOC1005064	runt-related transcription factor 1 | uncharacterized LOC100506403 | uncharacterized	-1.1494272	0.00096173
1558822_at	NA	NA	NA	-1.07400031	0.00096252
210190_at	8676	STX11	syntaxin 11	1.8351003	0.0009628
1559362_at	NA	NA	NA	-1.22780284	0.00096973
230276_at	81553	FAM49A	family with sequence similarity 49, member A	1.23487559	0.00097491
240175_at	NA	NA	NA	-1.12275936	0.0009786
222771_s_at	50804	MYEF2	myelin expression factor 2	2.41324813	0.00097904
204334_at	8609	KLF7	Kruppel-like factor 7 (ubiquitous)	1.29658681	0.00097966
223051_at	29101	SSU72	SSU72 RNA polymerase II CTD phosphatase homolog (S. cerevisiae)	-1.13142186	0.00099494
210142_x_at	10211	FLOT1	flotillin 1	1.16676055	0.00099715
236439_at	NA	NA	NA	1.87626023	0.00099912
34206_at	116985	ARAP1	ArfGAP with RhoGAP domain, ankyrin repeat and PH domain 1	1.05997188	0.00100404
223568_s_at	84513	PPAPDC1B	phosphatidic acid phosphatase type 2 domain containing 1B	-0.78103959	0.0010043
208807_s_at	1107	CHD3	chromodomain helicase DNA binding protein 3	0.90415825	0.00101488
1554240_a_at	3683	ITGAL	integrin, alpha L (antigen CD11A (p180), lymphocyte function-associated antigen 1; a	1.93671046	0.00101564
243454_at	NA	NA	NA	-1.62337977	0.00101588
203853_s_at	9846	GAB2	GRB2-associated binding protein 2	-1.19629109	0.0010193
202510_s_at	7127	TNFAIP2	tumor necrosis factor, alpha-induced protein 2	1.90732869	0.00102443
237387_at	NA	NA	NA	1.47624222	0.00102555
223268_at	28970	C11orf54	chromosome 11 open reading frame 54	-1.16450047	0.00102888
244716_x_at	126259	TMIGD2	transmembrane and immunoglobulin domain containing 2	-0.80720131	0.00103198
240652_at	NA	NA	NA	1.67896669	0.00103635
235199_at	54941	RNF125	ring finger protein 125, E3 ubiquitin protein ligase	-1.52631687	0.00103889
207719_x_at	9859 | 645455	CEP170 | CEP170P1	centrosomal protein 170kDa | centrosomal protein 170kDa pseudogene 1	-0.99569505	0.00104691

243158_at	NA	NA	NA	-1.60841297	0.00105115
1553992_s_at	10230	NBR2	neighbor of BRCA1 gene 2 (non-protein coding)	-0.9987371	0.00106164
208070_s_at	5980	REV3L	REV3-like, polymerase (DNA directed), zeta, catalytic subunit	-0.75487554	0.00107449
228287_at	84289	ING5	inhibitor of growth family, member 5	-1.18989181	0.00107695
208073_x_at	7267 | 28649!	TTC3 | TTC3P1	tetratricopeptide repeat domain 3 | tetratricopeptide repeat domain 3 pseudogene 1	-0.95723388	0.00108587
90265_at	11033	ADAP1	ArfGAP with dual PH domains 1	0.96689568	0.00109016
231183_s_at	NA	NA	NA	-2.5212433	0.00109018
206059_at	7644	ZNF91	zinc finger protein 91	-2.01778951	0.00110084
232441_at	11103	KRR1	KRR1, small subunit (SSU) processome component, homolog (yeast)	-0.95269986	0.00110496
215203_at	2803	GOLGA4	golgin A4	-1.11506261	0.00110646
210389_x_at	51174	TUBD1	tubulin, delta 1	-1.05702361	0.00110923
237881_at	NA	NA	NA	-1.28879526	0.00111
242399_at	NA	NA	NA	1.73797799	0.00111203
242827_x_at	NA	NA	NA	-1.23767755	0.00111514
225037_at	51006	SLC35C2	solute carrier family 35 (GDP-fucose transporter), member C2	-1.05018958	0.00111576
207697_x_at	10288	LILRB2	leukocyte immunoglobulin-like receptor, subfamily B (with TM and ITIM domains), me	1.91688822	0.00111605
211404_s_at	334	APLP2	amyloid beta (A4) precursor-like protein 2	1.42650804	0.00111737
222164_at	2260	FGFR1	fibroblast growth factor receptor 1	-1.46104872	0.00111828
1555845_at	NA	NA	NA	-1.27228066	0.00111922
224327_s_at	84649	DGAT2	diacylglycerol O-acyltransferase 2	1.08272599	0.00113656
1569527_at	NA	NA	NA	-1.51780534	0.00113719
215281_x_at	23126	POGZ	pogo transposable element with ZNF domain	-1.39631366	0.00113848
232239_at	643529	LINC00865	long intergenic non-protein coding RNA 865	1.5095949	0.00113915
212350_at	23216	TBC1D1	TBC1 (tre-2/USP6, BUB2, cdc16) domain family, member 1	1.14247189	0.00114204
201849_at	664	BNIP3	BCL2/adenovirus E1B 19kDa interacting protein 3	-1.63356043	0.00115421
225029_at	550643	LINC01420	long intergenic non-protein coding RNA 1420	-1.27741345	0.00115839
225656_at	114327	EFHC1	EF-hand domain (C-terminal) containing 1	-1.09824132	0.00116018
242751_at	NA	NA	NA	-1.27464712	0.00117197
219330_at	81839	VANGL1	VANGL planar cell polarity protein 1	-1.75000686	0.0011741
222761_at	54841	BIVM	basic, immunoglobulin-like variable motif containing	-1.79972067	0.0011747
201952_at	214	ALCAM	activated leukocyte cell adhesion molecule	-1.56750392	0.00117902
232113_at	NA	NA	NA	-3.28068973	0.00117984
225105_at	387882	C12orf75	chromosome 12 open reading frame 75	-2.39578506	0.00118013
225775_at	340348	TSPAN33	tetraspanin 33	-0.847309	0.00118113
59697_at	376267	RAB15	RAB15, member RAS oncogene family	-1.05817318	0.00118208
242440_at	NA	NA	NA	-1.04880116	0.00118292
227417_at	54996	MARC2	mitochondrial amidoxime reducing component 2	-1.9426742	0.00119266
229787_s_at	8473	OGT	O-linked N-acetylglucosamine (GlcNAc) transferase	-1.43641635	0.00119588
222858_s_at	27071	DAPP1	dual adaptor of phosphotyrosine and 3-phosphoinositides	2.15974169	0.00119795
208917_x_at	65220	NADK	NAD kinase	1.36060101	0.00120087
239296_at	NA	NA	NA	1.60437062	0.00120211
208723_at	8237	USP11	ubiquitin specific peptidase 11	-0.78958415	0.0012054
224357_s_at	51338	MS4A4A	membrane-spanning 4-domains, subfamily A, member 4A	1.36285252	0.00121273
216950_s_at	2209 | 2210 |	FCGR1A | FCGR1B | F(	Fc fragment of IgG, high affinity Ia, receptor (CD64) | Fc fragment of IgG, high affinit	2.07040502	0.00121517
213610_s_at	151230 | 1005	KLHL23 | PHOSPHO2-I	kelch-like family member 23 | PHOSPHO2-KLHL23 readthrough	-1.14635671	0.0012184
206656_s_at	57136	APMAP	adipocyte plasma membrane associated protein	-0.79870346	0.00122042
211990_at	3113	HLA-DPA1	major histocompatibility complex, class II, DP alpha 1	2.3329854	0.00122366
214670_at	7586	ZKSCAN1	zinc finger with KRAB and SCAN domains 1	-1.42953581	0.00122569
217974_at	51768	TM7SF3	transmembrane 7 superfamily member 3	-1.57752366	0.0012334
229615_at	128710	SLX4IP	SLX4 interacting protein	-1.07699013	0.00123484
228562_at	65986	ZBTB10	zinc finger and BTB domain containing 10	-1.19437574	0.00123745
221235_s_at	9392	TGFBRAP1	transforming growth factor, beta receptor associated protein 1	-0.96818905	0.00124001
227572_at	84749	USP30	ubiquitin specific peptidase 30	-0.9815604	0.00124132
233596_at	NA	NA	NA	-1.05936229	0.00124832
215239_x_at	10793	ZNF273	zinc finger protein 273	-1.24273276	0.00125565
212462_at	23522	KAT6B	K(lysine) acetyltransferase 6B	-0.87873592	0.00126171
238861_at	23635	SSBP2	single-stranded DNA binding protein 2	-1.55220086	0.00126236
210660_at	11024	LILRA1	leukocyte immunoglobulin-like receptor, subfamily A (with TM domain), member 1	1.95304381	0.00126465
206828_at	7294	TXK	TXK tyrosine kinase	-1.85753783	0.00126854
224675_at	23184	MESDC2	mesoderm development candidate 2	-0.89885288	0.00126856
201743_at	929	CD14	CD14 molecule	3.93132895	0.00127062
201422_at	10437 | 5296	IFI30 | PIK3R2	interferon, gamma-inducible protein 30 | phosphoinositide-3-kinase, regulatory subui	2.86115508	0.00127112
206042_x_at	8926 | 6638	SNURF | SNRPN	SNRPN upstream reading frame | small nuclear ribonucleoprotein polypeptide N	1.89931831	0.00127346
214875_x_at	334	APLP2	amyloid beta (A4) precursor-like protein 2	1.51417176	0.00127459
202843_at	4189	DNAJB9	DnaJ (Hsp40) homolog, subfamily B, member 9	-1.27726888	0.00129318
236465_at	285533	RNF175	ring finger protein 175	1.89121978	0.00129336
240297_at	NA	NA	NA	-1.44260557	0.00129385
224802_at	54602	NDFIP2	Nedd4 family interacting protein 2	-2.76294747	0.00129444
227410_at	131583	FAM43A	family with sequence similarity 43, member A	-1.30171785	0.00129698
201581_at	56255	TMX4	thioredoxin-related transmembrane protein 4	-0.95500187	0.00129761
200622_x_at	808 | 801 | 8C	CALM3 | CALM1 | CAL	calmodulin 3 (phosphorylase kinase, delta) | calmodulin 1 (phosphorylase kinase, del	0.83485553	0.00130197
39402_at	3553	IL1B	interleukin 1, beta	-1.50876876	0.00130776
220085_at	3070	HELLS	helicase, lymphoid-specific	1.7064156	0.00131632
213475_s_at	3683	ITGAL	integrin, alpha L (antigen CD11A (p180), lymphocyte function-associated antigen 1; a	1.48509822	0.00132264
231644_at	NA	NA	NA	2.71419153	0.00132657
212873_at	23526	HMHA1	histocompatibility (minor) HA-1	1.39386916	0.00132675
212803_at	4665	NAB2	NGFI-A binding protein 2 (EGR1 binding protein 2)	-0.85889679	0.00132802
210039_s_at	5588	PRKCQ	protein kinase C, theta	-1.2410712	0.00132845
207741_x_at	7177	TPSAB1	tryptase alpha/beta 1	-2.21776637	0.00133077
38149_at	9938	ARHGAP25	Rho GTPase activating protein 25	-0.99327667	0.00133083
244158_at	NA	NA	NA	1.31276093	0.00133157
227069_at	1523	CUX1	cut-like homeobox 1	-1.08972413	0.00133643
221771_s_at	54737	MPHOSPH8	M-phase phosphoprotein 8	-0.98330211	0.00134065
236610_at	NA	NA	NA	-1.62868563	0.00134643
209683_at	81553	FAM49A	family with sequence similarity 49, member A	1.9130465	0.00135079
211991_s_at	3113	HLA-DPA1	major histocompatibility complex, class II, DP alpha 1	2.53635161	0.00135653
219972_s_at	64430	PCNXL4	pecanex-like 4 (Drosophila)	-1.00415073	0.00135794
227603_at	NA	NA	NA	-1.13646904	0.00136276
215253_s_at	1827	RCAN1	regulator of calcineurin 1	1.0366078	0.00136594
203186_s_at	6275	S100A4	S100 calcium binding protein A4	1.60428268	0.00136655
221832_s_at	7798	LUZP1	leucine zipper protein 1	-0.83813322	0.00137168
1553219_a_at	9949	AMMECR1	Alport syndrome, mental retardation, midface hypoplasia and elliptocytosis chromoso	1.86270524	0.0013718
217807_s_at	29997 | 6920!	GLTSCR2 | SNORD23	glioma tumor suppressor candidate region gene 2 | small nucleolar RNA, C/D box 23	-0.88821427	0.0013728
208894_at	3122	HLA-DRA	major histocompatibility complex, class II, DR alpha	2.61964709	0.00137375
1557737_s_at	4820	NKTR	natural killer cell triggering receptor	-1.70439355	0.00137825
1557889_at	NA	NA	NA	1.57800067	0.00138393
210448_s_at	5026	P2RX5	purinergic receptor P2X, ligand gated ion channel, 5	-1.34601323	0.00138498
203375_s_at	7174	TPP2	tripeptidyl peptidase II	-0.8981327	0.00138604
210239_at	10265	IRX5	iroquois homeobox 5	-1.93334933	0.0013879
226295_at	55846 | 1005(	ITFG2 | LOC10050742	integrin alpha FG-GAP repeat containing 2 | uncharacterized LOC100507424	-0.68192671	0.00138917
226219_at	257106	ARHGAP30	Rho GTPase activating protein 30	0.99535496	0.00139022
218217_at	59342	SCPEP1	serine carboxypeptidase 1	1.99621216	0.00139706
225327_at	56204	FAM214A	family with sequence similarity 214, member A	-0.890523	0.00139958
226035_at	57478	USP31	ubiquitin specific peptidase 31	1.49326596	0.00140385

209619_at	972	CD74	CD74 molecule, major histocompatibility complex, class II invariant chain	1.94185524	0.00141715
220005_at	53829	P2RY13	purinergic receptor P2Y, G-protein coupled, 13	2.79685814	0.00141872
228264_at	84680	ACCS	1-aminocyclopropane-1-carboxylate synthase homolog (Arabidopsis)(non-functional)	-1.40853054	0.0014287
208127_s_at	9655	SOCS5	suppressor of cytokine signaling 5	-0.90211697	0.00143011
239442_at	23177	CEP68	centrosomal protein 68kDa	-1.10076511	0.00143848
227934_at	3841	KPNA5	karyopherin alpha 5 (importin alpha 6)	-1.21180763	0.00144354
213274_s_at	1508	CTSB	cathepsin B	1.48363438	0.00144477
231550_at	NA	NA	NA	-0.90444515	0.00144654
216103_at	26027	ACOT11	acyl-CoA thioesterase 11	-1.26680334	0.00145166
203761_at	6503	SLA	Src-like-adaptor	1.47121469	0.00145586
214051_at	286527 | 1101	TMSB15B | TMSB15A	thymosin beta 15B | thymosin beta 15a	-1.45603039	0.00146251
201681_s_at	9231	DLG5	discs, large homolog 5 (Drosophila)	-1.70875045	0.0014631
203004_s_at	4209	MEF2D	myocyte enhancer factor 2D	0.82200668	0.00146591
204183_s_at	157	ADRBK2	adrenergic, beta, receptor kinase 2	0.8613923	0.0014667
201140_s_at	5878	RAB5C	RAB5C, member RAS oncogene family	0.75252803	0.00146871
242550_at	8662	EIF3B	eukaryotic translation initiation factor 3, subunit B	-1.12135721	0.00146876
1565703_at	4089	SMAD4	SMAD family member 4	-1.25854336	0.00147821
204315_s_at	51512	GTSE1	G-2 and S-phase expressed 1	1.05869308	0.00149538
217223_s_at	613	BCR	breakpoint cluster region	-0.91896161	0.00149643
1557238_s_at	NA	NA	NA	-1.91298366	0.00150155
210530_s_at	7181	NR2C1	nuclear receptor subfamily 2, group C, member 1	-0.89659302	0.00150437
206554_x_at	6419	SETMAR	SET domain and mariner transposase fusion gene	-1.20042529	0.00153114
212945_s_at	23269	MGA	MGA, MAX dimerization protein	-0.8855245	0.00153182
217591_at	6498	SKIL	SKI-like proto-oncogene	-1.88523889	0.00153715
214627_at	8288	EPX	eosinophil peroxidase	-1.90896715	0.00155112
206995_x_at	8578	SCARF1	scavenger receptor class F, member 1	-1.40738905	0.00155202
205659_at	9734	HDAC9	histone deacetylase 9	2.33699784	0.00155441
217690_at	55556	ENOSF1	enolase superfamily member 1	-1.3012773	0.0015577
203949_at	4353	MPO	myeloperoxidase	-3.52094685	0.00156034
1569320_at	60313	GPBP1L1	GC-rich promoter binding protein 1-like 1	-0.88830283	0.00156497
224835_at	56261	GPCPD1	glycerophosphocholine phosphodiesterase GDE1 homolog (S. cerevisiae)	1.07166134	0.00156705
214523_at	1053	CEBPE	CCAAT/enhancer binding protein (C/EBP), epsilon	-1.38885628	0.00157049
34689_at	11277 | 8412€	TREX1 | ATRIP	three prime repair exonuclease 1 | ATR interacting protein	0.72538596	0.00157097
37152_at	5467	PPARD	peroxisome proliferator-activated receptor delta	0.90817502	0.0015777
222444_at	51566	ARMCX3	armadillo repeat containing, X-linked 3	-1.21057212	0.00158476
200762_at	1808	DPYSL2	dihydropyrimidinase-like 2	2.46958958	0.00158626
213196_at	23361	ZNF629	zinc finger protein 629	-0.71079896	0.00158975
208634_s_at	23499	MACF1	microtubule-actin crosslinking factor 1	-0.86951731	0.00159805
223006_s_at	23731	TMEM245	transmembrane protein 245	-0.84599385	0.00159981
203291_at	4850	CNOT4	CCR4-NOT transcription complex, subunit 4	-1.0650023	0.00160003
201282_at	4967	OGDH	oxoglutarate (alpha-ketoglutarate) dehydrogenase (lipoamide)	0.90439272	0.0016027
228929_at	1773	DNASE1	deoxyribonuclease I	-0.76966585	0.00160942
227162_at	57684	ZBTB26	zinc finger and BTB domain containing 26	-1.33034474	0.00161852
1560720_at	8569	MKNK1	MAP kinase interacting serine/threonine kinase 1	-1.0785908	0.00161922
1558487_a_at	222068	TMED4	transmembrane emp24 protein transport domain containing 4	-0.82214636	0.00162612
227737_at	58477	SRPRB	signal recognition particle receptor, B subunit	-0.75568466	0.00162772
224826_at	56261	GPCPD1	glycerophosphocholine phosphodiesterase GDE1 homolog (S. cerevisiae)	1.28092583	0.00162814
214795_at	23613	ZMYND8	zinc finger, MYND-type containing 8	-0.89208894	0.00163168
210658_s_at	23062	GGA2	golgi-associated, gamma adaptin ear containing, ARF binding protein 2	1.33271867	0.00163224
238005_s_at	25942	SIN3A	SIN3 transcription regulator family member A	-1.02880857	0.00163842
211133_x_at	11025	LILRB3	leukocyte immunoglobulin-like receptor, subfamily B (with TM and ITIM domains), me	1.2682913	0.00163995
225224_at	140688	NOL4L	nucleolar protein 4-like	-1.2541903	0.00164253
203759_at	6484	ST3GAL4	ST3 beta-galactoside alpha-2,3-sialyltransferase 4	-1.16448806	0.00166689
232676_x_at	50804	MYEF2	myelin expression factor 2	2.05789725	0.00167025
219279_at	55619	DOCK10	dedicator of cytokinesis 10	1.57448764	0.00167382
1559882_at	25939	SAMHD1	SAM domain and HD domain 1	2.44812842	0.00167936
203675_at	4925	NUCB2	nucleobindin 2	-1.60656772	0.00167941
202122_s_at	10226	PLIN3	perilipin 3	-0.88635133	0.00167994
226152_at	145567	TTC7B	tetratricopeptide repeat domain 7B	-1.52372158	0.00169216
205786_s_at	3684	ITGAM	integrin, alpha M (complement component 3 receptor 3 subunit)	2.06752812	0.00169313
224512_s_at	84316	NAA38	N(alpha)-acetyltransferase 38, NatC auxiliary subunit	0.88867546	0.00169402
219463_at	24141	LAMP5	lysosomal-associated membrane protein family, member 5	3.72116295	0.00169552
207106_s_at	4058	LTK	leukocyte receptor tyrosine kinase	-1.74053562	0.00169616
214370_at	6279	S100A8	S100 calcium binding protein A8	2.0468185	0.00169697
208370_s_at	1827	RCAN1	regulator of calcineurin 1	1.71327156	0.00169824
1555526_a_at	23157	SEPT6	septin 6	-1.31774533	0.00170477
220911_s_at	57523	NYNRIN	NYN domain and retroviral integrase containing	-1.03938511	0.0017049
228402_at	84327	ZBED3	zinc finger, BED-type containing 3	-1.0628194	0.00170655
208626_s_at	10493	VAT1	vesicle amine transport 1	-1.07300987	0.00172696
210904_s_at	3597	IL13RA1	interleukin 13 receptor, alpha 1	2.30077774	0.00172803
215855_s_at	7110	TMF1	TATA element modulatory factor 1	-0.85151645	0.00173038
222742_s_at	64792	IFT22	intraflagellar transport 22	-1.1667338	0.00173108
239761_at	2650	GCNT1	glucosaminyl (N-acetyl) transferase 1, core 2	-1.31996994	0.00173426
1556008_a_at	NA	NA	NA	-1.01920476	0.00173656
243745_at	NA	NA	NA	1.87580689	0.00174005
210358_x_at	2624	GATA2	GATA binding protein 2	-1.18526588	0.00174515
215159_s_at	65220	NADK	NAD kinase	1.37275538	0.0017493
212830_at	1955	MEGF9	multiple EGF-like-domains 9	2.05948551	0.00175442
227333_at	123879	DCUN1D3	DCN1, defective in cullin neddylation 1, domain containing 3	-0.85609775	0.00175998
228719_at	125150	ZSWIM7	zinc finger, SWIM-type containing 7	-1.04582492	0.00176028
209732_at	9976	CLEC2B	C-type lectin domain family 2, member B	2.51518337	0.00176137
215785_s_at	26999	CYFIP2	cytoplasmic FMR1 interacting protein 2	-1.30723527	0.00176586
210425_x_at	23015 | 4402?	GOLGA8A | GOLGA8B	golgin A8 family, member A | golgin A8 family, member B	-2.12879403	0.00176846
218454_at	79887	PLBD1	phospholipase B domain containing 1	3.42937094	0.00177666
1563364_at	NA	NA	NA	-1.6036929	0.00179463
211366_x_at	834	CASP1	caspase 1, apoptosis-related cysteine peptidase	1.7190956	0.00179523
223527_s_at	81602	CDADC1	cytidine and dCMP deaminase domain containing 1	-0.89308071	0.00179659
203659_s_at	10206	TRIM13	tripartite motif containing 13	-0.9162704	0.00180378
238161_at	NA	NA	NA	-0.93290226	0.00181649
1554600_s_at	4000	LMNA	lamin A/C	1.96710616	0.00182188
228826_at	100506779	BZRAP1-AS1	BZRAP1 antisense RNA 1	-1.20794278	0.00183169
1562265_at	NA	NA	NA	-1.21228467	0.00183705
1565597_at	NA	NA	NA	1.74167557	0.00184053
205685_at	942	CD86	CD86 molecule	1.235378	0.00184762
206634_at	6496	SIX3	SIX homeobox 3	-2.5563782	0.00185445
234068_s_at	160	AP2A1	adaptor-related protein complex 2, alpha 1 subunit	1.2192734	0.00187068
224791_at	50807	ASAP1	ArfGAP with SH3 domain, ankyrin repeat and PH domain 1	1.61745298	0.00187081
232306_at	60437	CDH26	cadherin 26	-1.77200772	0.00188443
202944_at	4668	NAGA	N-acetylgalactosaminidase, alpha-	1.37640131	0.00188806
218361_at	55204	GOLPH3L	golgi phosphoprotein 3-like	-0.83940205	0.001893
226722_at	56975	FAM20C	family with sequence similarity 20, member C	2.09054917	0.00189472
57532_at	1856	DVL2	dishevelled segment polarity protein 2	-0.68826909	0.00189765
1554638_at	9765	ZFYVE16	zinc finger, FYVE domain containing 16	-1.47730399	0.00189846
202630_at	10513	APPBP2	amyloid beta precursor protein (cytoplasmic tail) binding protein 2	-1.13557213	0.00189847

212423_at	219654	ZCCHC24	zinc finger, CCHC domain containing 24	1.08357146	0.00190003
213761_at	56890	MDM1	Mdm1 nuclear protein homolog (mouse)	-1.07839166	0.00190199
202655_at	7873	MANF	mesencephalic astrocyte-derived neurotrophic factor	-1.15187038	0.00190591
239682_at	151050	KANSL1L	KAT8 regulatory NSL complex subunit 1-like	-1.26340023	0.00191007
235222_x_at	331	XIAP	X-linked inhibitor of apoptosis, E3 ubiquitin protein ligase	-0.91513135	0.00191644
214511_x_at	2210	FCGR1B	Fc fragment of IgG, high affinity Ib, receptor (CD64)	2.20992604	0.00191679
235902_at	NA	NA	NA	-1.40267374	0.00192212
212285_s_at	375790	AGRN	agrin	-1.63129536	0.0019361
205769_at	11001	SLC27A2	solute carrier family 27 (fatty acid transporter), member 2	-1.79942713	0.00193682
225992_at	8028	MLLT10	myeloid/lymphoid or mixed-lineage leukemia (trithorax homolog, Drosophila); translo	-0.96748767	0.00193913
242449_at	NA	NA	NA	-1.01438679	0.00194228
224577_at	57222	ERGIC1	endoplasmic reticulum-golgi intermediate compartment (ERGIC) 1	-1.00988225	0.00194494
209386_at	4071	TM4SF1	transmembrane 4 L six family member 1	-2.40530325	0.00195614
201656_at	3655	ITGA6	integrin, alpha 6	-1.40406406	0.00195703
218363_at	55218	EXD2	exonuclease 3'-5' domain containing 2	-0.90793534	0.0019591
212255_s_at	27032	ATP2C1	ATPase, Ca++ transporting, type 2C, member 1	-1.11475417	0.00197685
218471_s_at	582	BBS1	Bardet-Biedl syndrome 1	-0.87232054	0.001986
208656_s_at	10983	CCNI	cyclin I	-0.63598673	0.00198692
242585_at	100131067	CKMT2-AS1	CKMT2 antisense RNA 1	-1.20845523	0.00199491
1560145_at	4289	MKLN1	muskelin 1, intracellular mediator containing kelch motifs	-1.09493606	0.00200552
210982_s_at	3122	HLA-DRA	major histocompatibility complex, class II, DR alpha	2.41366199	0.00200827
227144_at	23313	KIAA0930	KIAA0930	1.01168704	0.00201165
218756_s_at	79154	DHRS11	dehydrogenase/reductase (SDR family) member 11	-1.72432776	0.00201735
215599_at	11039 | 65318	SMA4 | GUSBP3 | GUS	glucuronidase, beta pseudogene | glucuronidase, beta pseudogene 3 | glucuronidase,	-1.90011757	0.00201939
221523_s_at	58528	RRAGD	Ras-related GTP binding D	-1.5980803	0.00202264
206695_x_at	7594	ZNF43	zinc finger protein 43	-1.2125851	0.00202306
219714_s_at	55799	CACNA2D3	calcium channel, voltage-dependent, alpha 2/delta subunit 3	2.57902234	0.00203391
201718_s_at	2037	EPB41L2	erythrocyte membrane protein band 4.1-like 2	1.98404846	0.00203458
239937_at	7756	ZNF207	zinc finger protein 207	-1.34108024	0.00203542
230131_x_at	414	ARSD	arylsulfatase D	-1.44368097	0.00204007
227334_at	159195	USP54	ubiquitin specific peptidase 54	-1.18347061	0.0020426
228099_at	162972	ZNF550	zinc finger protein 550	-0.99806938	0.0020509
224341_x_at	7099	TLR4	toll-like receptor 4	2.24013088	0.00205343
225831_at	7798	LUZP1	leucine zipper protein 1	-0.7651265	0.00205389
220059_at	26228	STAP1	signal transducing adaptor family member 1	-1.86384943	0.00205609
209574_s_at	753	LDLRAD4	low density lipoprotein receptor class A domain containing 4	-2.40261446	0.00205878
203814_s_at	4835	NQO2	NAD(P)H dehydrogenase, quinone 2	-1.12752645	0.00206218
202741_at	5567	PRKACB	protein kinase, cAMP-dependent, catalytic, beta	-1.14780214	0.00206328
217653_x_at	NA	NA	NA	-1.83407592	0.0020633
234192_s_at	80318	GKAP1	G kinase anchoring protein 1	-1.41325968	0.00207651
224583_at	23406	COTL1	coactosin-like F-actin binding protein 1	1.78631176	0.00207977
212125_at	5905	RANGAP1	Ran GTPase activating protein 1	1.09514606	0.00208634
225237_s_at	124540	MSI2	musashi RNA-binding protein 2	-2.41178093	0.00209281
209970_x_at	834	CASP1	caspase 1, apoptosis-related cysteine peptidase	1.778541	0.00209616
215633_x_at	7940	LST1	leukocyte specific transcript 1	1.71832797	0.00209919
203542_s_at	687	KLF9	Kruppel-like factor 9	1.61970194	0.00210115
209729_at	10634	GAS2L1	growth arrest-specific 2 like 1	-0.90241802	0.00210957
225329_at	348262	FAM195B	family with sequence similarity 195, member B	-0.74330055	0.00211327
239784_at	NA	NA	NA	-1.50294107	0.00212522
206572_x_at	7639	ZNF85	zinc finger protein 85	-1.22566172	0.00213344
205213_at	9744	ACAP1	ArfGAP with coiled-coil, ankyrin repeat and PH domains 1	-0.91774134	0.00214077
209340_at	6675	UAP1	UDP-N-acetylglucosamine pyrophosphorylase 1	-1.67975546	0.00214084
232504_at	285628 | 4069	LOC285628 | MIR146A	uncharacterized LOC285628 | microRNA 146a	-2.07139153	0.002141
218854_at	29940	DSE	dermatan sulfate epimerase	-2.29701396	0.00214124
210629_x_at	7940	LST1	leukocyte specific transcript 1	1.6812692	0.00215161
226991_at	4773	NFATC2	nuclear factor of activated T-cells, cytoplasmic, calcineurin-dependent 2	-1.45143186	0.00215352
200766_at	1509	CTSD	cathepsin D	1.31304692	0.00215475
225026_at	84181	CHD6	chromodomain helicase DNA binding protein 6	-0.94096217	0.00216037
222218_s_at	29992	PILRA	paired immunoglobin-like type 2 receptor alpha	1.46008225	0.00217009
222483_at	79180	EFHD2	EF-hand domain family, member D2	0.89373067	0.00217158
1552329_at	5930	RBBP6	retinoblastoma binding protein 6	-1.31988723	0.00217331
212573_at	23052	ENDOD1	endonuclease domain containing 1	1.85089057	0.00218068
1553183_at	89766	UMODL1	uromodulin-like 1	-2.88988543	0.00218102
224927_at	170954	PPP1R18	protein phosphatase 1, regulatory subunit 18	1.18141205	0.00218406
204153_s_at	4242	MFNG	MFNG O-fucosylpeptide 3-beta-N-acetylglucosaminyltransferase	1.12606188	0.00218543
64432_at	51275	MAPKAPK5-AS1	MAPKAPK5 antisense RNA 1	-0.78471576	0.00218783
1552330_at	92806	CENPBD1	CENPB DNA-binding domains containing 1	-1.37215882	0.00223305
209734_at	3071	NCKAP1L	NCK-associated protein 1-like	0.91582181	0.00223511
54037_at	89781	HPS4	Hermansky-Pudlak syndrome 4	-0.93487763	0.00224142
1567107_s_at	7171	TPM4	tropomyosin 4	-1.79913007	0.00224321
1558410_s_at	NA	NA	NA	-1.21909471	0.00224723
218005_at	7570	ZNF22	zinc finger protein 22	-0.94959026	0.00224876
218071_s_at	23609	MKRN2	makorin ring finger protein 2	-0.71129634	0.00225318
35776_at	6453	ITSN1	intersectin 1 (SH3 domain protein)	0.79693124	0.00225834
213309_at	23228	PLCL2	phospholipase C-like 2	1.26269754	0.00227117
239893_at	NA	NA	NA	-2.34069828	0.00227334
215385_at	NA	NA	NA	-1.14335648	0.00227379
227234_at	100132815	IPO5P1	importin 5 pseudogene 1	-1.07137739	0.00228246
211582_x_at	7940	LST1	leukocyte specific transcript 1	1.82851137	0.00228988
1556728_at	NA	NA	NA	-1.56973935	0.00229413
243568_at	NA	NA	NA	-0.89636961	0.00230318
228424_at	10004	NAALADL1	N-acetylated alpha-linked acidic dipeptidase-like 1	-1.2776789	0.00230418
235125_x_at	374986	FAM73A	family with sequence similarity 73, member A	-0.88730856	0.00231005
234199_at	NA	NA	NA	-2.16775474	0.00231123
209407_s_at	10522	DEAF1	DEAF1 transcription factor	-0.78724356	0.00232112
209155_s_at	22978	NT5C2	5'-nucleotidase, cytosolic II	-1.22860836	0.00232277
217763_s_at	11031	RAB31	RAB31, member RAS oncogene family	2.36654646	0.0023228
219759_at	64167	ERAP2	endoplasmic reticulum aminopeptidase 2	-1.48799216	0.00232761
209331_s_at	4149	MAX	MYC associated factor X	-1.04027522	0.00233579
223264_at	59274	MESDC1	mesoderm development candidate 1	-0.67073398	0.00233816
208798_x_at	23015	GOLGA8A	golgin A8 family, member A	-2.61032561	0.00234178
1554827_a_at	113	ADCY7	adenylate cyclase 7	0.85790403	0.00235619
1557478_at	NA	NA	NA	-1.08955271	0.00236107
206420_at	10261	IGSF6	immunoglobulin superfamily, member 6	2.43032744	0.00236271
227198_at	3899	AFF3	AF4/FMR2 family, member 3	2.60020112	0.00236435
203411_s_at	4000	LMNA	lamin A/C	1.92016013	0.00236664
227523_s_at	51105	PHF20L1	PHD finger protein 20-like 1	0.60439514	0.00236813
201029_s_at	4267	CD99	CD99 molecule	-1.31766882	0.0023688
221213_s_at	54816 | 14578	ZNF280D | LOC14578	zinc finger protein 280D | uncharacterized LOC145783	-1.13903013	0.00237014
203111_s_at	2185	PTK2B	protein tyrosine kinase 2 beta	0.81977822	0.00237288
208906_at	26580 | 10053	BSCL2 | HNRNPUL2-BS	Berardinelli-Seip congenital lipodystrophy 2 (seipin) | HNRNPUL2-BSCL2 readthrough	-0.86372465	0.0023789
204143_s_at	55556	ENOSF1	enolase superfamily member 1	-1.58568329	0.00238039
203975_s_at	10036	CHAF1A	chromatin assembly factor 1, subunit A (p150)	0.87512034	0.00238874
239432_at	379025	PSMA3-AS1	PSMA3 antisense RNA 1	-1.00354112	0.00238985

209298_s_at	6453	ITSN1	intersectin 1 (SH3 domain protein)	1.27203812	0.00239378
226127_at	221120	ALKBH3	alkB, alkylation repair homolog 3 (E. coli)	-0.71412503	0.0023951
201938_at	8099	CDK2AP1	cyclin-dependent kinase 2 associated protein 1	-1.01029474	0.00241559
220999_s_at	26999	CYFIP2	cytoplasmic FMR1 interacting protein 2	-1.1441719	0.00241731
201074_at	6599	SMARCC1	SWI/SNF related, matrix associated, actin dependent regulator of chromatin, subfami	-0.69023034	0.00242742
238412_at	100131998 | 1	RRN3P3 | LOC10106(	RNA polymerase I transcription factor homolog (S. cerevisiae) pseudogene 3 | putati\	-0.95948427	0.00242998
55093_at	54480	CHPF2	chondroitin polymerizing factor 2	-0.69258087	0.00244024
212146_at	23207	PLEKHM2	pleckstrin homology domain containing, family M (with RUN domain) member 2	0.72649366	0.0024437
205634_x_at	79143 | 25435	MBOAT7 | ZDHHC24	membrane bound O-acyltransferase domain containing 7 | zinc finger, DHHC-type cor	-0.61943255	0.00244387
212149_at	23167	EFR3A	EFR3 homolog A (S. cerevisiae)	0.96062116	0.00244478
239083_at	136051	ZNF786	zinc finger protein 786	-1.02832025	0.00245534
202934_at	3099	HK2	hexokinase 2	-1.3606926	0.00246147
228788_at	29799	YPEL1	yippee-like 1 (Drosophila)	-1.46578224	0.00246472
208248_x_at	334	APLP2	amyloid beta (A4) precursor-like protein 2	1.29258999	0.00246765
228275_at	100505687	LINC00888	long intergenic non-protein coding RNA 888	-1.09485124	0.00247173
209702_at	79068	FTO	fat mass and obesity associated	-1.06588257	0.00247696
212197_x_at	23164	MPRIP	myosin phosphatase Rho interacting protein	-0.58940131	0.00248745
207621_s_at	10400	PEMT	phosphatidylethanolamine N-methyltransferase	-0.86536223	0.00249037
242121_at	100302692	FTX	FTX transcript, XIST regulator (non-protein coding)	-1.09403721	0.00249247
220176_at	80224	NUBPL	nucleotide binding protein-like	-1.17235518	0.00249424
208949_s_at	3958	LGALS3	lectin, galactoside-binding, soluble, 3	2.37338305	0.00249479
226771_at	57198	ATP8B2	ATPase, aminophospholipid transporter, class I, type 8B, member 2	-1.02615633	0.00251439
222816_s_at	54877	ZCCHC2	zinc finger, CCHC domain containing 2	1.43084278	0.00252116
216071_x_at	9968	MED12	mediator complex subunit 12	-0.69625859	0.00253333
220917_s_at	57728	WDR19	WD repeat domain 19	-1.16637765	0.00255225
223519_at	51776	ZAK	sterile alpha motif and leucine zipper containing kinase AZK	2.16016798	0.00255841
1558299_at	NA	NA	NA	1.19133893	0.00256439
227489_at	64750	SMURF2	SMAD specific E3 ubiquitin protein ligase 2	-1.0704431	0.00256504
228897_at	91319	DERL3	derlin 3	-1.09194447	0.00256612
230174_at	127018	LYPLAL1	lysophospholipase-like 1	-0.9295904	0.00256917
1555960_at	3094	HINT1	histidine triad nucleotide binding protein 1	-1.29158654	0.00257182
244357_at	NA	NA	NA	1.81929359	0.00257286
226317_at	151987	PPP4R2	protein phosphatase 4, regulatory subunit 2	-1.02200659	0.00257778
221193_s_at	54819	ZCCHC10	zinc finger, CCHC domain containing 10	-1.03160626	0.00258433
218700_s_at	8934	RAB29	RAB29, member RAS oncogene family	1.26698089	0.00259702
227062_at	283131 | 6931	NEAT1 | MIR612	nuclear paraspeckle assembly transcript 1 (non-protein coding) | microRNA 612	2.61108489	0.00260262
1559025_at	10801	SEPT9	septin 9	-1.16162389	0.00261061
209791_at	11240	PADI2	peptidyl arginine deiminase, type II	1.14511442	0.00261586
215034_s_at	4071	TM4SF1	transmembrane 4 L six family member 1	-1.94115183	0.00261769
1570571_at	55297	CCDC91	coiled-coil domain containing 91	-1.42635125	0.00261912
222266_at	NA	NA	NA	-1.25328407	0.00261992
223253_at	54749	EPDR1	ependymin related 1	-1.50492118	0.00262139
228594_at	133686	NADK2	NAD kinase 2, mitochondrial	-1.061523	0.00263993
207134_x_at	64499	TPSB2	tryptase beta 2 (gene/pseudogene)	-2.50362735	0.00264209
204158_s_at	10312	TCIRG1	T-cell, immune regulator 1, ATPase, H+ transporting, lysosomal V0 subunit A3	1.27836219	0.0026608
207275_s_at	2180	ACSL1	acyl-CoA synthetase long-chain family member 1	1.35196817	0.00266098
225876_at	57185	NIPAL3	NIPA-like domain containing 3	-0.94186436	0.00266435
216264_s_at	3913	LAMB2	laminin, beta 2 (laminin S)	-1.41951729	0.00266773
225378_at	137492	VPS37A	vacuolar protein sorting 37 homolog A (S. cerevisiae)	-1.01923552	0.00267009
225457_s_at	25845 | 90271	PP7080 | OLMALINC	uncharacterized LOC25845 | oligodendrocyte maturation-associated long intergenic n	-0.87296416	0.00267094
233473_x_at	NA	NA	NA	-1.00433447	0.00267558
222258_s_at	23677	SH3BP4	SH3-domain binding protein 4	-1.84072962	0.00269507
229665_at	1479	CSTF3	cleavage stimulation factor, 3' pre-RNA, subunit 3, 77kDa	-1.23456456	0.00270254
238449_at	595101	SMG1P5	SMG1 pseudogene 5	-1.04754109	0.0027031
204000_at	10681	GNB5	guanine nucleotide binding protein (G protein), beta 5	-0.99881169	0.00270512
211342_x_at	9968	MED12	mediator complex subunit 12	-0.76210678	0.00271097
238076_at	57459	GATAD2B	GATA zinc finger domain containing 2B	-0.76545745	0.00271878
223464_at	114879	OSBPL5	oxysterol binding protein-like 5	-1.16074519	0.00272565
242673_at	NA	NA	NA	-1.10309721	0.00272957
1566501_at	NA	NA	NA	-1.56947687	0.00273687
226495_at	57506	MAVS	mitochondrial antiviral signaling protein	-0.75017672	0.00274193
228389_at	NA	NA	NA	-0.95508725	0.00274601
220832_at	51311	TLR8	toll-like receptor 8	2.36908142	0.0027467
230472_at	79192	IRX1	iroquois homeobox 1	-3.15313701	0.00275478
202548_s_at	8874	ARHGEF7	Rho guanine nucleotide exchange factor (GEF) 7	-0.70606485	0.00277009
238631_at	NA	NA	NA	-1.16396995	0.00277325
209321_s_at	109	ADCY3	adenylate cyclase 3	1.18603905	0.00279845
209930_s_at	4778	NFE2	nuclear factor, erythroid 2	-1.36583258	0.00279962
218503_at	54914	FOCAD	focadhesin	-0.95166947	0.0028069
242051_at	NA	NA	NA	-2.01911296	0.00281019
203030_s_at	5799	PTPRN2	protein tyrosine phosphatase, receptor type, N polypeptide 2	1.41417449	0.00281397
218792_s_at	54836	BSPRY	B-box and SPRY domain containing	-1.42382771	0.00282119
204164_at	6494	SIPA1	signal-induced proliferation-associated 1	0.71060691	0.0028319
218559_s_at	9935	MAFB	v-maf avian musculoaponeurotic fibrosarcoma oncogene homolog B	2.66873786	0.00284486
244022_at	NA	NA	NA	-2.0552058	0.00284515
205239_at	374	AREG	amphiregulin	2.81999193	0.00285047
205582_s_at	2687	GGT5	gamma-glutamyltransferase 5	-1.11612229	0.0028756
233224_at	NA	NA	NA	-0.81672799	0.00288361
227145_at	84171	LOXL4	lysyl oxidase-like 4	-1.73522296	0.00288832
202950_at	1429	CRYZ	crystallin, zeta (quinone reductase)	-1.13066382	0.00289124
222734_at	10352	WARS2	tryptophanyl tRNA synthetase 2, mitochondrial	-0.83230364	0.00289283
202013_s_at	2132	EXT2	exostosin glycosyltransferase 2	-0.91589573	0.00289473
226629_at	124935	SLC43A2	solute carrier family 43 (amino acid system L transporter), member 2	1.04643072	0.00289536
221216_s_at	22955	SCMH1	sex comb on midleg homolog 1 (Drosophila)	-0.9059974	0.00289981
1568865_at	2342	FNTB	farnesyltransferase, CAAX box, beta	-1.1600724	0.00290422
228758_at	604	BCL6	B-cell CLL/lymphoma 6	1.08850614	0.00290751
223248_at	83693	HSDL1	hydroxysteroid dehydrogenase like 1	-0.90451	0.00291273
227951_s_at	147965	FAM98C	family with sequence similarity 98, member C	1.09141012	0.00291277
213800_at	3075	CFH	complement factor H	-2.2814949	0.00292861
212188_at	115207	KCTD12	potassium channel tetramerization domain containing 12	3.09896047	0.00292962
243249_at	NA	NA	NA	-0.74881791	0.00293271
212421_at	23313	KIAA0930	KIAA0930	0.97044032	0.00293446
210701_at	10428	CFDP1	craniofacial development protein 1	-1.51746941	0.00293708
238043_at	57492	ARID1B	AT rich interactive domain 1B (SWI1-like)	-1.09441558	0.00294099
210548_at	6368	CCL23	chemokine (C-C motif) ligand 23	2.15571174	0.00294138
231851_at	55225	RAVER2	ribonucleoprotein, PTB-binding 2	-1.3415534	0.00294282
225767_at	100507412 | 1	LOC100507412 | RNA	uncharacterized LOC100507412 | RNA, 45S pre-ribosomal 5	1.10673725	0.00294448
224794_s_at	51148	CERCAM	cerebral endothelial cell adhesion molecule	-1.49573912	0.00294654
219431_at	79658	ARHGAP10	Rho GTPase activating protein 10	-1.03192549	0.00295981
208981_at	5175	PECAM1	platelet/endothelial cell adhesion molecule 1	1.55383079	0.00296097
235028_at	NA	NA	NA	-1.67282526	0.00296142
221596_s_at	84060	RBM48	RNA binding motif protein 48	-0.96419605	0.00296696
220617_s_at	55205	ZNF532	zinc finger protein 532	1.29764942	0.00297467
204142_at	55556	ENOSF1	enolase superfamily member 1	-1.63736807	0.00297471

222996_s_at	51523	CXXC5	CXXC finger protein 5	-1.29518145	0.00297797
207522_s_at	489	ATP2A3	ATPase, Ca++ transporting, ubiquitous	-1.08710216	0.0029915
230110_at	255231	MCOLN2	mucolipin 2	1.54742977	0.002997
243010_at	124540	MSI2	musashi RNA-binding protein 2	-1.68830674	0.0030006
208806_at	1107	CHD3	chromodomain helicase DNA binding protein 3	0.86620816	0.00301305
223283_s_at	10194	TSHZ1	teashirt zinc finger homeobox 1	-1.02047785	0.00301323
219724_s_at	9840	TESPA1	thymocyte expressed, positive selection associated 1	-1.08962542	0.00301872
211136_s_at	1209	CLPTM1	cleft lip and palate associated transmembrane protein 1	0.85195925	0.00302845
227948_at	121512	FGD4	FYVE, RhoGEF and PH domain containing 4	2.2202532	0.00305906
202845_s_at	10928	RALBP1	ralA binding protein 1	-0.7500297	0.00306004
215398_at	NA	NA	NA	-0.95098271	0.00306092
218050_at	51569	UFM1	ubiquitin-fold modifier 1	-0.7825205	0.00307156
204174_at	241	ALOX5AP	arachidonate 5-lipoxygenase-activating protein	1.9096943	0.00307929
1556338_at	NA	NA	NA	-0.9969999	0.00309081
225065_x_at	125144 | 2680	LRRC75A-AS1 | SNORI	LRRC75A antisense RNA 1 | small nucleolar RNA, C/D box 49A | small nucleolar RNA,	-0.96471285	0.00309713
219574_at	55016	MARCH1	membrane-associated ring finger (C3HC4) 1, E3 ubiquitin protein ligase	2.51323568	0.00310933
227461_at	85439	STON2	stonin 2	-2.75371825	0.00310937
205068_s_at	23092	ARHGAP26	Rho GTPase activating protein 26	0.95154455	0.00311195
207945_s_at	1453	CSNK1D	casein kinase 1, delta	0.89643129	0.00311994
225851_at	2342 | 100529	FNTB | CHURC1-FNTB	farnesyltransferase, CAAX box, beta | CHURC1-FNTB readthrough	-0.59411262	0.00312306
203026_at	9925	ZBTB5	zinc finger and BTB domain containing 5	-0.74615548	0.00312577
239451_at	NA	NA	NA	-1.46484315	0.00312996
1565743_at	10443	N4BP2L2	NEDD4 binding protein 2-like 2	-1.5595075	0.00313724
201054_at	10949	HNRNPA0	heterogeneous nuclear ribonucleoprotein A0	-0.84890887	0.00313892
228532_at	128346	C1orf162	chromosome 1 open reading frame 162	1.26677199	0.00314059
220643_s_at	55179	FAIM	Fas apoptotic inhibitory molecule	-1.3945602	0.00314276
212750_at	26051	PPP1R16B	protein phosphatase 1, regulatory subunit 16B	-0.87393917	0.00314384
225743_at	285367	RPUSD3	RNA pseudouridylate synthase domain containing 3	-0.96231363	0.00314505
222482_at	23648	SSBP3	single stranded DNA binding protein 3	-1.48222482	0.00315394
218694_at	51309	ARMCX1	armadillo repeat containing, X-linked 1	-1.41385995	0.00316088
212481_s_at	7171	TPM4	tropomyosin 4	-1.52901089	0.00316268
217734_s_at	11180	WDR6	WD repeat domain 6	-0.65759711	0.00316347
1568834_s_at	60492	CCDC90B	coiled-coil domain containing 90B	-0.87359297	0.00316891
202778_s_at	7750	ZMYM2	zinc finger, MYM-type 2	-0.7024065	0.00317799
209774_x_at	2920	CXCL2	chemokine (C-X-C motif) ligand 2	-1.91203564	0.00319942
219383_at	79899	PRR5L	proline rich 5 like	-1.41566936	0.00319964
239835_at	84541	KBTBD8	kelch repeat and BTB (POZ) domain containing 8	1.13412656	0.0032031
203388_at	409	ARRB2	arrestin, beta 2	1.2786829	0.00320607
200859_x_at	2316	FLNA	filamin A, alpha	1.09411017	0.00320833
215050_x_at	9261	MAPKAPK2	mitogen-activated protein kinase-activated protein kinase 2	0.77742487	0.00321166
222757_s_at	51776	ZAK	sterile alpha motif and leucine zipper containing kinase AZK	1.44290443	0.00322563
202565_s_at	6840	SVIL	supervillin	-1.52257512	0.00322611
1557987_at	NA	NA	NA	-1.33560845	0.00324877
213346_at	93081	TEX30	testis expressed 30	-1.27531574	0.00325617
209622_at	8576	STK16	serine/threonine kinase 16	0.73904511	0.00325764
209431_s_at	23598	PATZ1	POZ (BTB) and AT hook containing zinc finger 1	-0.90950271	0.00325781
229582_at	125476	INO80C	INO80 complex subunit C	-0.86629627	0.00325949
239731_at	NA	NA	NA	-1.18056597	0.00326025
219360_s_at	54795	TRPM4	transient receptor potential cation channel, subfamily M, member 4	1.88574805	0.00327353
208843_s_at	26003	GORASP2	golgi reassembly stacking protein 2, 55kDa	-0.87065743	0.00327573
206829_x_at	80264	ZNF430	zinc finger protein 430	-1.10108138	0.00329038
208051_s_at	10605	PAIP1	poly(A) binding protein interacting protein 1	-1.03256116	0.00329287
205469_s_at	3663	IRF5	interferon regulatory factor 5	0.96046389	0.00329392
213385_at	1124	CHN2	chimerin 2	-1.37436219	0.00330128
214574_x_at	7940	LST1	leukocyte specific transcript 1	1.59210399	0.00330497
200721_s_at	10121	ACTR1A	ARP1 actin-related protein 1 homolog A, centractin alpha (yeast)	0.59147168	0.0033179
210754_s_at	4067	LYN	LYN proto-oncogene, Src family tyrosine kinase	1.46478177	0.00332219
221858_at	23232	TBC1D12	TBC1 domain family, member 12	1.3503328	0.00332963
239740_at	2120	ETV6	ets variant 6	-1.82046789	0.00333918
200617_at	9761	MLEC	malectin	-0.81873509	0.00334654
242932_at	NA	NA	NA	1.10707671	0.00334882
201391_at	10131	TRAP1	TNF receptor-associated protein 1	0.89701294	0.00336168
213864_s_at	4673	NAP1L1	nucleosome assembly protein 1-like 1	-0.85681266	0.00336818
226150_at	84513	PPAPDC1B	phosphatidic acid phosphatase type 2 domain containing 1B	-1.04402942	0.00337298
202060_at	9646	CTR9	CTR9, Paf1/RNA polymerase II complex component	-0.87069804	0.00338338
208908_s_at	831	CAST	calpastatin	1.58075433	0.00339629
1566480_x_at	284071	C17orf104	chromosome 17 open reading frame 104	-1.19255131	0.00339932
223062_s_at	29968	PSAT1	phosphoserine aminotransferase 1	2.19529141	0.0033996
231844_at	157247 | 5513	MGC27345 | RBM28	uncharacterized protein MGC27345 | RNA binding motif protein 28	-0.87241491	0.00340023
212226_s_at	8613	PPAP2B	phosphatidic acid phosphatase type 2B	1.30847188	0.0034004
200972_at	10099	TSPAN3	tetraspanin 3	-0.93388492	0.00340312
202027_at	25829	TMEM184B	transmembrane protein 184B	-0.8119772	0.00340475
201137_s_at	3115	HLA-DPB1	major histocompatibility complex, class II, DP beta 1	1.95983237	0.00341048
213943_at	7291	TWIST1	twist family bHLH transcription factor 1	-2.20191765	0.00341105
227923_at	85358	SHANK3	SH3 and multiple ankyrin repeat domains 3	-1.6813131	0.00341262
223347_at	84939	MUM1	melanoma associated antigen (mutated) 1	-0.98887791	0.0034127
232599_at	54536	EXOC6	exocyst complex component 6	-1.12944309	0.0034147
235432_at	27031	NPHP3	nephronophthisis 3 (adolescent)	-1.42916433	0.00341714
200931_s_at	7414	VCL	vinculin	1.57962421	0.00342253
200976_s_at	8887	TAX1BP1	Tax1 (human T-cell leukemia virus type I) binding protein 1	-0.81010104	0.00342884
242357_x_at	NA	NA	NA	-1.13829996	0.00343825
244474_at	NA	NA	NA	-0.80887764	0.00344724
213447_at	6638 | 3653 |	SNRPN | IPW | SNORD	small nuclear ribonucleoprotein polypeptide N | imprinted in Prader-Willi syndrome (n	1.32899356	0.00345813
215123_at	23117 | 44034	NPIPB3 | NPIPB4 | LO	nuclear pore complex interacting protein family, member B3 | nuclear pore complex i	-1.76583891	0.00347048
207167_at	9398	CD101	CD101 molecule	1.50867486	0.00347767
225376_at	54994	GID8	GID complex subunit 8	-0.72649225	0.00347803
226204_at	54584 | 79680	GNB1L | C22orf29	guanine nucleotide binding protein (G protein), beta polypeptide 1-like | chromosome	-0.87768399	0.00348064
201417_at	6659	SOX4	SRY (sex determining region Y)-box 4	-1.47016165	0.00348307
209648_x_at	9655	SOCS5	suppressor of cytokine signaling 5	-0.92233748	0.00349062
239709_at	NA	NA	NA	-1.39029493	0.00349347
232168_x_at	23499	MACF1	microtubule-actin crosslinking factor 1	-1.02452609	0.00350219
219217_at	79731	NARS2	asparaginyl-tRNA synthetase 2, mitochondrial (putative)	-0.84390731	0.00350584
221804_s_at	404636 | 5585	FAM45A | FAM45B	family with sequence similarity 45, member A | family with sequence similarity 45, m	-1.33314379	0.0035185
212704_at	23318	ZCCHC11	zinc finger, CCHC domain containing 11	-0.91982922	0.00352258
202351_at	3685	ITGAV	integrin, alpha V	-2.05080091	0.00352628
1552798_a_at	7099	TLR4	toll-like receptor 4	1.64804614	0.00353278
214864_s_at	9380	GRHPR	glyoxylate reductase/hydroxypyruvate reductase	-0.74042632	0.00353567
216032_s_at	51614	ERGIC3	ERGIC and golgi 3	-0.58593352	0.00353765
236554_x_at	147138	TMC8	transmembrane channel-like 8	1.09621921	0.00355866
204181_s_at	23099	ZBTB43	zinc finger and BTB domain containing 43	1.2927555	0.00356686
202085_at	9414	TJP2	tight junction protein 2	1.86874644	0.00356788
225733_at	126792	B3GALT6	UDP-Gal:betaGal beta 1,3-galactosyltransferase polypeptide 6	-0.71233253	0.00357476
235693_at	NA	NA	NA	-1.02216436	0.00357989
205875_s_at	11277 | 84126	TREX1 | ATRIP	three prime repair exonuclease 1 | ATR interacting protein	0.74870677	0.0036044

218043_s_at	64343	AZI2	5-azacytidine induced 2	-0.85521625	0.003607
208703_s_at	334	APLP2	amyloid beta (A4) precursor-like protein 2	1.60950332	0.00361772
223663_at	283234	CCDC88B	coiled-coil domain containing 88B	0.72803745	0.00361934
221850_x_at	119016 | 1193	AGAP4 | AGAP11 | AG	ArfGAP with GTPase domain, ankyrin repeat and PH domain 4 | ankyrin repeat and G	-0.87419358	0.0036201
241577_at	NA	NA	NA	0.7755061	0.00362642
209711_at	23169	SLC35D1	solute carrier family 35 (UDP-GlcA/UDP-GalNAc transporter), member D1	-0.78136393	0.00362828
202592_at	2647	BLOC1S1	biogenesis of lysosomal organelles complex-1, subunit 1	0.68829066	0.00363403
215990_s_at	604	BCL6	B-cell CLL/lymphoma 6	1.44147632	0.00364861
218971_s_at	29062	WDR91	WD repeat domain 91	0.74791285	0.00365411
232068_s_at	7099	TLR4	toll-like receptor 4	1.84070083	0.0036585
223182_s_at	56894	AGPAT3	1-acylglycerol-3-phosphate O-acyltransferase 3	1.06157545	0.00366442
224558_s_at	378938	MALAT1	metastasis associated lung adenocarcinoma transcript 1 (non-protein coding)	-0.89352579	0.00366476
224909_s_at	57580	PREX1	phosphatidylinositol-3,4,5-trisphosphate-dependent Rac exchange factor 1	1.13781822	0.00366508
238736_at	5980	REV3L	REV3-like, polymerase (DNA directed), zeta, catalytic subunit	-0.88477471	0.00366855
209049_s_at	23613	ZMYND8	zinc finger, MYND-type containing 8	-1.0156402	0.00366883
212594_at	27250 | 10061	PDCD4 | MIR4680	programmed cell death 4 (neoplastic transformation inhibitor) | microRNA 4680	-0.84110469	0.00366986
207821_s_at	5747	PTK2	protein tyrosine kinase 2	1.54530416	0.00367027
200077_s_at	4946	OAZ1	ornithine decarboxylase antizyme 1	0.53846318	0.00367879
222859_s_at	27071	DAPP1	dual adaptor of phosphotyrosine and 3-phosphoinositides	1.52656883	0.003692
227663_at	NA	NA	NA	-1.23065156	0.00369433
224982_at	84335	AKT1S1	AKT1 substrate 1 (proline-rich)	0.87093876	0.00369775
226143_at	10743	RAI1	retinoic acid induced 1	-0.63313967	0.00369882
238800_s_at	79670	ZCCHC6	zinc finger, CCHC domain containing 6	1.18025956	0.00369965
227791_at	285195	SLC9A9	solute carrier family 9, subfamily A (NHE9, cation proton antiporter 9), member 9	0.78450714	0.00370172
1554241_at	1690	COCH	cochlin	-0.93630472	0.00370359
202659_at	5699	PSMB10	proteasome (prosome, macropain) subunit, beta type, 10	1.11397559	0.00370992
212855_at	23142	DCUN1D4	DCN1, defective in cullin neddylation 1, domain containing 4	-0.95558653	0.00371141
204972_at	4939	OAS2	2'-5'-oligoadenylate synthetase 2, 69/71kDa	1.68678279	0.00372716
213852_at	114814 | 9939	GNRHR2 | RBM8A	gonadotropin-releasing hormone (type 2) receptor 2, pseudogene | RNA binding moti	-0.71526752	0.00372931
229638_at	79191	IRX3	iroquois homeobox 3	-3.42401753	0.00373013
240128_at	NA	NA	NA	-1.04961482	0.00373246
206100_at	1368	CPM	carboxypeptidase M	2.23344578	0.00373454
226419_s_at	6426	SRSF1	serine/arginine-rich splicing factor 1	1.41368307	0.00373463
242111_at	56339	METTL3	methyltransferase like 3	-0.96492914	0.00373704
201888_s_at	3597	IL13RA1	interleukin 13 receptor, alpha 1	1.81565327	0.00374598
229168_at	91522	COL23A1	collagen, type XXIII, alpha 1	-1.65835227	0.00374879
228145_s_at	57541	ZNF398	zinc finger protein 398	-0.7519924	0.00375227
212041_at	9114	ATP6V0D1	ATPase, H+ transporting, lysosomal 38kDa, V0 subunit d1	1.01024282	0.00375607
229026_at	56990	CDC42SE2	CDC42 small effector 2	-1.73248957	0.00376215
239289_x_at	22909	FAN1	FANCD2/FANCI-associated nuclease 1	-1.02658745	0.00376863
206034_at	5271	SERPINB8	serpin peptidase inhibitor, clade B (ovalbumin), member 8	1.806198	0.00376871
203948_s_at	4353	MPO	myeloperoxidase	-3.60909853	0.0037809
223165_s_at	51447	IP6K2	inositol hexakisphosphate kinase 2	-1.17532609	0.00378557
200878_at	2034	EPAS1	endothelial PAS domain protein 1	-1.52382755	0.0037905
209394_at	8623	ASMTL	acetylserotonin O-methyltransferase-like	-0.897156	0.00379087
228258_at	374403	TBC1D10C	TBC1 domain family, member 10C	-1.05731776	0.00380015
207812_s_at	26003	GORASP2	golgi reassembly stacking protein 2, 55kDa	-0.73776464	0.00381351
232277_at	64078	SLC28A3	solute carrier family 28 (concentrative nucleoside transporter), member 3	-2.04466781	0.00381624
212498_at	10299	MARCH6	membrane-associated ring finger (C3HC4) 6, E3 ubiquitin protein ligase	-0.84280347	0.00382329
205047_s_at	440	ASNS	asparagine synthetase (glutamine-hydrolyzing)	-1.70378506	0.00382555
1557996_at	NA	NA	NA	-1.44442741	0.00383763
205899_at	8900	CCNA1	cyclin A1	-3.06772362	0.00384897
210874_s_at	24142	NAT6	N-acetyltransferase 6 (GCN5-related)	-0.83076874	0.00385047
230192_at	10206	TRIM13	tripartite motif containing 13	-1.2039637	0.00385346
204363_at	2152	F3	coagulation factor III (thromboplastin, tissue factor)	-1.70629763	0.00386465
221768_at	6421	SFPQ	splicing factor proline/glutamine-rich	-0.79520187	0.00387515
201721_s_at	7805	LAPTM5	lysosomal protein transmembrane 5	0.75053335	0.00387616
202431_s_at	4609	MYC	v-myc avian myelocytomatosis viral oncogene homolog	-0.98046162	0.00388461
1553407_at	23499	MACF1	microtubule-actin crosslinking factor 1	-1.39955105	0.00388467
224949_at	81555	YIPF5	Yip1 domain family, member 5	-0.76819607	0.00389175
211521_s_at	27128	CYTH4	cytohesin 4	1.04387604	0.00389909
229296_at	100506119	LINC01503	long intergenic non-protein coding RNA 1503	1.00802896	0.00390089
217741_s_at	7763	ZFAND5	zinc finger, AN1-type domain 5	0.94291086	0.00390753
201487_at	1075	CTSC	cathepsin C	-1.09668713	0.00390786
210423_s_at	6556	SLC11A1	solute carrier family 11 (proton-coupled divalent metal ion transporter), member 1	1.75277352	0.00390957
206314_at	55888	ZKSCAN7	zinc finger with KRAB and SCAN domains 7	-1.48828019	0.00391096
236292_at	55819	RNF130	ring finger protein 130	-1.29449936	0.00393218
232535_at	222194	RSBN1L	round spermatid basic protein 1-like	-1.68776083	0.00394745
206770_s_at	23443	SLC35A3	solute carrier family 35 (UDP-N-acetylglucosamine (UDP-GlcNAc) transporter), memb	-0.88961059	0.00396463
209864_at	23401	FRAT2	frequently rearranged in advanced T-cell lymphomas 2	1.11244	0.00396971
203320_at	10019	SH2B3	SH2B adaptor protein 3	0.9272161	0.00397937
213988_s_at	6303	SAT1	spermidine/spermine N1-acetyltransferase 1	1.64797065	0.00398025
217798_at	4848	CNOT2	CCR4-NOT transcription complex, subunit 2	-0.61994374	0.00398957
210549_s_at	6368	CCL23	chemokine (C-C motif) ligand 23	2.56596152	0.00400105
212841_s_at	8495	PPFIBP2	PTPRF interacting protein, binding protein 2 (liprin beta 2)	1.23892442	0.00400107
211794_at	2533	FYB	FYN binding protein	1.51867634	0.00400191
1560332_at	NA	NA	NA	-1.44434818	0.00400862
223018_at	28987	NOB1	NIN1/RPN12 binding protein 1 homolog (S. cerevisiae)	-0.66993072	0.00401697
206105_at	2334	AFF2	AF4/FMR2 family, member 2	-1.38974468	0.00401741
222763_s_at	55339 | 84826	WDR33 | SFT2D3	WD repeat domain 33 | SFT2 domain containing 3	-0.76028108	0.00402092
235479_at	132864	CPEB2	cytoplasmic polyadenylation element binding protein 2	0.87957425	0.00404282
210334_x_at	332	BIRC5	baculoviral IAP repeat containing 5	0.93174114	0.00407954
64899_at	64748	LPPR2	lipid phosphate phosphatase-related protein type 2	0.63857815	0.0040811
208024_s_at	8214 | 85359	DGCR6 | DGCR6L	DiGeorge syndrome critical region gene 6 | DiGeorge syndrome critical region gene 6	-0.80068641	0.00408743
206991_s_at	1234	CCR5	chemokine (C-C motif) receptor 5 (gene/pseudogene)	1.64635719	0.00410088
201637_s_at	8087	FXR1	fragile X mental retardation, autosomal homolog 1	-0.75148506	0.00410873
233364_s_at	NA	NA	NA	-2.78773833	0.00411049
206130_s_at	433	ASGR2	asialoglycoprotein receptor 2	1.1278068	0.00411324
235266_at	29028	ATAD2	ATPase family, AAA domain containing 2	1.28232059	0.00413302
216474_x_at	7177 | 64499	TPSAB1 | TPSB2	tryptase alpha/beta 1 | tryptase beta 2 (gene/pseudogene)	-2.55143607	0.00413618
1566142_at	NA	NA	NA	-1.1665521	0.00413934
230820_at	64750	SMURF2	SMAD specific E3 ubiquitin protein ligase 2	-0.79025073	0.00414304
214752_x_at	2316	FLNA	filamin A, alpha	1.06288802	0.00414873
233898_s_at	26127	FGFR1OP2	FGFR1 oncogene partner 2	-1.13969765	0.00415123
1554597_at	3980	LIG3	ligase III, DNA, ATP-dependent	-0.83994826	0.00415443
228181_at	7779	SLC30A1	solute carrier family 30 (zinc transporter), member 1	1.31638386	0.00417489
204699_s_at	27042	DIEXF	digestive organ expansion factor homolog (zebrafish)	-0.66609877	0.00419613
210933_s_at	6624	FSCN1	fascin actin-bundling protein 1	-1.65808941	0.00420241
227776_at	55331	ACER3	alkaline ceramidase 3	1.51856206	0.00420272
228702_at	378805	LINC-PINT	long intergenic non-protein coding RNA, p53 induced transcript	-1.50807029	0.00420476
226392_at	5922	RASA2	RAS p21 protein activator 2	-0.74317649	0.00421928
205683_x_at	7177	TPSAB1	tryptase alpha/beta 1	-2.26525917	0.00422273
219583_s_at	55812	SPATA7	spermatogenesis associated 7	-1.14771596	0.00422454
210347_s_at	53335	BCL11A	B-cell CLL/lymphoma 11A (zinc finger protein)	-1.19405088	0.00424237

1560275_at	93109	TMEM44	transmembrane protein 44	-1.13835129	0.00424481
226748_at	256586	LYSMD2	LysM, putative peptidoglycan-binding, domain containing 2	-0.839481	0.00424929
1554447_at	554203	JPX	JPX transcript, XIST activator (non-protein coding)	-1.34850033	0.00425089
221492_s_at	64422	ATG3	autophagy related 3	1.07522881	0.00425349
212593_s_at	27250 | 10061	PDCD4 | MIR4680	programmed cell death 4 (neoplastic transformation inhibitor) | microRNA 4680	-0.70427922	0.00425704
230413_s_at	NA	NA	NA	1.70192371	0.00426714
225630_at	80820	EEPD1	endonuclease/exonuclease/phosphatase family domain containing 1	-0.70904778	0.00427863
226784_at	221830	TWISTNB	TWIST neighbor	-1.33907937	0.00428608
236172_at	1241	LTB4R	leukotriene B4 receptor	1.58872234	0.00428715
224686_x_at	474170	LRRC37A2	leucine rich repeat containing 37, member A2	-0.89007546	0.00429085
213492_at	1280	COL2A1	collagen, type II, alpha 1	-1.88108917	0.00429474
204619_s_at	1462	VCAN	versican	3.06541037	0.004306
219889_at	10023	FRAT1	frequently rearranged in advanced T-cell lymphomas 1	1.17922293	0.00430721
225723_at	154467	CCDC167	coiled-coil domain containing 167	0.72341558	0.00432169
202739_s_at	5257	PHKB	phosphorylase kinase, beta	-0.97654976	0.00433165
225351_at	404636 | 5585	FAM45A | FAM45B	family with sequence similarity 45, member A | family with sequence similarity 45, m	-1.31233998	0.00433512
226412_at	25957	PNISR	PNN-interacting serine/arginine-rich protein	-0.99360814	0.0043375
205708_s_at	7226	TRPM2	transient receptor potential cation channel, subfamily M, member 2	0.9375764	0.00434098
201276_at	5869	RAB5B	RAB5B, member RAS oncogene family	-1.03687178	0.00434365
203397_s_at	2591	GALNT3	polypeptide N-acetylgalactosaminyltransferase 3	-1.85478275	0.00436542
219332_at	79778	MICALL2	MICAL-like 2	-1.18400806	0.00437011
205081_at	1396	CRIP1	cysteine-rich protein 1 (intestinal)	2.32242914	0.00437094
204115_at	2791	GNG11	guanine nucleotide binding protein (G protein), gamma 11	1.36423506	0.00438687
206871_at	1991	ELANE	elastase, neutrophil expressed	-3.21246009	0.00439696
221561_at	6646	SOAT1	sterol O-acyltransferase 1	1.14042982	0.00440201
204391_x_at	8805	TRIM24	tripartite motif containing 24	-0.92043722	0.00441491
222533_at	51185	CRBN	cereblon	-0.9455491	0.00441992
217967_s_at	116496	FAM129A	family with sequence similarity 129, member A	-1.847586	0.0044261
1555948_s_at	23196	FAM120A	family with sequence similarity 120A	1.00612669	0.00443077
204703_at	8100	IFT88	intraflagellar transport 88	-0.73718472	0.00443895
204341_at	10626	TRIM16	tripartite motif containing 16	-1.38109432	0.00443896
201152_s_at	4154	MBNL1	muscleblind-like splicing regulator 1	0.93215969	0.00444008
209240_at	8473	OGT	O-linked N-acetylglucosamine (GlcNAc) transferase	-0.88462298	0.00444126
230541_at	149134	LINC01341	long intergenic non-protein coding RNA 1341	-1.22974417	0.00444452
234989_at	283131 | 6931	NEAT1 | MIR612	nuclear paraspeckle assembly transcript 1 (non-protein coding) | microRNA 612	1.47104747	0.00446107
213848_at	1849	DUSP7	dual specificity phosphatase 7	1.04426136	0.004478
222603_at	79956	ERMP1	endoplasmic reticulum metallopeptidase 1	-1.32187291	0.00449164
222622_at	283871	PGP	phosphoglycolate phosphatase	0.97848727	0.00449477
1552736_a_at	81832	NETO1	neuropilin (NRP) and tolloid (TLL)-like 1	-1.71632674	0.00449511
215952_s_at	4946	OAZ1	ornithine decarboxylase antizyme 1	0.79765015	0.00450038
210788_s_at	51635	DHRS7	dehydrogenase/reductase (SDR family) member 7	-0.99393975	0.00450677
212018_s_at	26156	RSL1D1	ribosomal L1 domain containing 1	-1.12989535	0.00452056
235033_at	79716	NPEPL1	aminopeptidase-like 1	1.26349338	0.00453133
203932_at	3109	HLA-DMB	major histocompatibility complex, class II, DM beta	1.69800271	0.00453507
235052_at	126375	ZNF792	zinc finger protein 792	-1.07696678	0.00454081
220404_at	222487	ADGRG3	adhesion G protein-coupled receptor G3	-1.39281206	0.00454261
239376_at	64421	DCLRE1C	DNA cross-link repair 1C	-0.83848133	0.00456865
226480_at	NA	NA	NA	-0.80000453	0.00457303
217122_s_at	9906 | 728661	SLC35E2 | SLC35E2B	solute carrier family 35, member E2 | solute carrier family 35, member E2B	-0.83392223	0.00457425
215806_x_at	6967 | 6983 |	TRGC2 | TRGV9 | TARI	T cell receptor gamma constant 2 | T cell receptor gamma variable 9 | TCR gamma a	-2.01526333	0.00457976
204718_at	2051	EPHB6	EPH receptor B6	-0.75477517	0.0045841
204916_at	10267	RAMP1	receptor (G protein-coupled) activity modifying protein 1	-0.68591041	0.00461788
205382_s_at	1675	CFD	complement factor D (adipsin)	-2.26780668	0.00461865
1570165_at	NA	NA	NA	-1.70678919	0.0046261
212927_at	23137	SMC5	structural maintenance of chromosomes 5	-0.93609959	0.00463416
1554173_at	124599	CD300LB	CD300 molecule-like family member b	1.01644831	0.00463462
221731_x_at	1462	VCAN	versican	3.43232688	0.00463915
200785_s_at	4035	LRP1	low density lipoprotein receptor-related protein 1	0.98598262	0.00464286
221918_at	5128	CDK17	cyclin-dependent kinase 17	-0.92098458	0.0046445
239151_at	399761	BMS1P5	BMS1 pseudogene 5	-2.05863183	0.0046647
207968_s_at	4208	MEF2C	myocyte enhancer factor 2C	1.36349627	0.00467228
203573_s_at	5875	RABGGTA	Rab geranylgeranyltransferase, alpha subunit	0.60854381	0.00467381
1554555_a_at	79918	SETD6	SET domain containing 6	-0.59619627	0.00467418
221740_x_at	474170	LRRC37A2	leucine rich repeat containing 37, member A2	-1.06302742	0.00469067
222692_s_at	64778	FNDC3B	fibronectin type III domain containing 3B	-1.41708809	0.00469978
213996_at	29799	YPEL1	yippee-like 1 (Drosophila)	-1.18746975	0.00470075
214181_x_at	7940	LST1	leukocyte specific transcript 1	1.79997349	0.004703
232307_at	NA	NA	NA	-0.9446951	0.00470354
219036_at	80321	CEP70	centrosomal protein 70kDa	-1.67631459	0.00471381
235508_at	5371	PML	promyelocytic leukemia	-0.94932919	0.00471599
209371_s_at	6452	SH3BP2	SH3-domain binding protein 2	1.07037707	0.00472689
209014_at	9500	MAGED1	melanoma antigen family D1	-1.52932147	0.0047398
222786_at	55501	CHST12	carbohydrate (chondroitin 4) sulfotransferase 12	-1.44381815	0.00474227
218699_at	8934	RAB29	RAB29, member RAS oncogene family	1.17698891	0.00474836
228605_at	165324	UBXN2A	UBX domain protein 2A	-0.77970477	0.00475669
217586_x_at	NA	NA	NA	-0.75221877	0.00476512
211571_s_at	1462	VCAN	versican	3.5109664	0.00477541
235371_at	727936	GXYLT2	glucoside xylosyltransferase 2	-2.29195243	0.00477681
213394_at	23005	MAPKBP1	mitogen-activated protein kinase binding protein 1	-0.76408552	0.00478004
208763_s_at	1831	TSC22D3	TSC22 domain family, member 3	1.08937721	0.00478757
203588_s_at	7029	TFDP2	transcription factor Dp-2 (E2F dimerization partner 2)	-1.42751273	0.00478793
230848_s_at	23269	MGA	MGA, MAX dimerization protein	-0.90996827	0.00480104
208368_s_at	675	BRCA2	breast cancer 2, early onset	0.88210715	0.00483016
215786_at	NA	NA	NA	-1.0837234	0.00483076
225927_at	4214	MAP3K1	mitogen-activated protein kinase kinase kinase 1, E3 ubiquitin protein ligase	-0.95120062	0.00483334
209393_s_at	9470	EIF4E2	eukaryotic translation initiation factor 4E family member 2	0.75254521	0.00483752
208405_s_at	8763	CD164	CD164 molecule, sialomucin	-0.9417399	0.00484635
233690_at	NA	NA	NA	-1.2338428	0.00485266
227576_at	NA	NA	NA	-0.89971255	0.00485738
242797_x_at	NA	NA	NA	-1.55499192	0.00486891
218517_at	79960	JADE1	jade family PHD finger 1	-0.91144077	0.00487604
227038_at	166929	SGMS2	sphingomyelin synthase 2	2.59583419	0.00488033
233955_x_at	51523	CXXC5	CXXC finger protein 5	-1.47292463	0.00488035
235281_x_at	79026	AHNAK	AHNAK nucleoprotein	1.60638735	0.00488489
212671_s_at	3117 | 3118	HLA-DQA1 | HLA-DQA	major histocompatibility complex, class II, DQ alpha 1 | major histocompatibility com	2.46560066	0.00488618
233138_at	753	LDLRAD4	low density lipoprotein receptor class A domain containing 4	-1.29899389	0.00489041
243827_at	NA	NA	NA	-1.20197897	0.00489808
235900_at	201305	SPNS3	spinster homolog 3 (Drosophila)	-1.51917688	0.00490488
200704_at	9516	LITAF	lipopolysaccharide-induced TNF factor	-1.10365183	0.00492407
227889_at	54947	LPCAT2	lysophosphatidylcholine acyltransferase 2	-2.18238554	0.00492529
1557270_at	NA	NA	NA	-1.99189382	0.00493241
203801_at	63931	MRPS14	mitochondrial ribosomal protein S14	-1.04104268	0.0049384
203665_at	3162	HMOX1	heme oxygenase (decycling) 1	1.66442056	0.00494695
224679_at	23184	MESDC2	mesoderm development candidate 2	-0.59170431	0.00495303

224759_s_at	90488	TMEM263	transmembrane protein 263	-1.0862517	0.00495672
242241_x_at	NA	NA	NA	-0.95951773	0.0049664
201389_at	3678	ITGA5	integrin, alpha 5 (fibronectin receptor, alpha polypeptide)	-0.89376954	0.00497874
213233_s_at	55958	KLHL9	kelch-like family member 9	-0.83883874	0.00498107
201845_s_at	23429	RYBP	RING1 and YY1 binding protein	-1.03602595	0.00498482
219010_at	55765	C1orf106	chromosome 1 open reading frame 106	1.99920931	0.00498824
220081_x_at	51478	HSD17B7	hydroxysteroid (17-beta) dehydrogenase 7	-0.93184797	0.0049938
223059_s_at	83641	FAM107B	family with sequence similarity 107, member B	1.72270081	0.00499698
229865_at	64778	FNDC3B	fibronectin type III domain containing 3B	-1.42280267	0.00501495
227049_at	284273	ZADH2	zinc binding alcohol dehydrogenase domain containing 2	-1.1195628	0.00502721
220079_s_at	84196	USP48	ubiquitin specific peptidase 48	-0.75233339	0.00503946
227551_at	51104	ABHD17B	abhydrolase domain containing 17B	-0.96236673	0.00504615
213867_x_at	60	ACTB	actin, beta	0.49008892	0.0050488
224987_at	221477	C6orf89	chromosome 6 open reading frame 89	0.6701045	0.005071
225921_at	51199	NIN	ninein (GSK3B interacting protein)	-1.01306894	0.00507124
226663_at	100505494	ANKRD10-IT1	ANKRD10 intronic transcript 1	-1.57742163	0.00507838
224594_x_at	60	ACTB	actin, beta	0.5018602	0.00510345
1570151_at	NA	NA	NA	-1.0035169	0.00512649
204319_s_at	6001	RGS10	regulator of G-protein signaling 10	1.30992088	0.00512932
239001_at	4257	MGST1	microsomal glutathione S-transferase 1	-1.67808595	0.00513539
219235_s_at	65979	PHACTR4	phosphatase and actin regulator 4	-1.28364791	0.00514254
208636_at	87	ACTN1	actinin, alpha 1	-1.19270842	0.00514576
207076_s_at	445	ASS1	argininosuccinate synthase 1	-1.45682133	0.00515044
1553043_a_at	146722	CD300LF	CD300 molecule-like family member f	1.96963379	0.00516546
218508_at	55802	DCP1A	decapping mRNA 1A	-0.59428131	0.00519663
229390_at	441168	FAM26F	family with sequence similarity 26, member F	-2.5960134	0.0052137
233296_x_at	NA	NA	NA	-0.93216879	0.00522093
226125_at	100288152	LOC100288152	uncharacterized LOC100288152	-1.14557303	0.00523449
205119_s_at	2357	FPR1	formyl peptide receptor 1	1.94565773	0.00523604
204624_at	540	ATP7B	ATPase, Cu++ transporting, beta polypeptide	-1.66161816	0.00523781
238418_at	84912	SLC35B4	solute carrier family 35 (UDP-xylose/UDP-N-acetylglucosamine transporter), member	-1.31887229	0.00523904
209831_x_at	1777	DNASE2	deoxyribonuclease II, lysosomal	-0.6719343	0.00524206
238912_x_at	138241	C9orf85	chromosome 9 open reading frame 85	-0.68474246	0.00524292
218967_s_at	9317	PTER	phosphotriesterase related	0.71623055	0.00525682
200628_s_at	7453	WARS	tryptophanyl-tRNA synthetase	1.2050735	0.00526222
208820_at	5747	PTK2	protein tyrosine kinase 2	1.9210617	0.00527836
206687_s_at	5777	PTPN6	protein tyrosine phosphatase, non-receptor type 6	1.06551679	0.00528548
241133_at	28560	TRBV27	T cell receptor beta variable 27	-2.37201864	0.00529496
231836_at	284459	HKR1	HKR1, GLI-Kruppel zinc finger family member	-0.90931248	0.00530006
212259_s_at	57326	PBXIP1	pre-B-cell leukemia homeobox interacting protein 1	-0.94047074	0.00530371
232356_at	NA	NA	NA	1.80276695	0.00531243
242652_at	NA	NA	NA	1.32982462	0.0053516
244623_at	56479	KCNQ5	potassium channel, voltage gated KQT-like subfamily Q, member 5	-1.95517947	0.00536143
229700_at	148203	ZNF738	zinc finger protein 738	-1.35887596	0.00537873
213212_x_at	374650 | 4402	GOLGA6L5P | GOLGA6	golgin A6 family-like 5, pseudogene | golgin A6 family-like 9 | golgin A6 family-like 4	-1.41476446	0.0053802
224848_at	1021	CDK6	cyclin-dependent kinase 6	-1.139031	0.00538164
242337_at	NA	NA	NA	-0.75500943	0.0053824
41577_at	26051	PPP1R16B	protein phosphatase 1, regulatory subunit 16B	-1.13225345	0.00538666
221060_s_at	7099	TLR4	toll-like receptor 4	2.24218722	0.005387
226889_at	57539	WDR35	WD repeat domain 35	-1.23735959	0.00539698
202086_at	4599	MX1	MX dynamin-like GTPase 1	1.49205441	0.00541003
1557751_at	NA	NA	NA	-1.00104658	0.00542978
1556339_a_at	NA	NA	NA	-1.04769944	0.00543817
221641_s_at	23597	ACOT9	acyl-CoA thioesterase 9	1.72329432	0.00543833
222357_at	26137	ZBTB20	zinc finger and BTB domain containing 20	-0.96552385	0.0054481
216705_s_at	100	ADA	adenosine deaminase	-1.30109185	0.00544815
208982_at	5175	PECAM1	platelet/endothelial cell adhesion molecule 1	1.34019372	0.00545047
1553974_at	128977	C22orf39	chromosome 22 open reading frame 39	-0.71165441	0.00546414
225091_at	85364	ZCCHC3	zinc finger, CCHC domain containing 3	-0.53065121	0.00548353
226175_at	283237	TTC9C	tetratricopeptide repeat domain 9C	-0.68451877	0.00549634
235408_x_at	51351	ZNF117	zinc finger protein 117	-1.15526786	0.00549981
229492_at	81839	VANGL1	VANGL planar cell polarity protein 1	-1.58782976	0.00551132
229513_at	55342	STRBP	spermatid perinuclear RNA binding protein	-1.15975248	0.0055155
232617_at	1520	CTSS	cathepsin S	1.70573651	0.0055351
224709_s_at	56990	CDC42SE2	CDC42 small effector 2	-0.76026081	0.00554401
202054_s_at	224	ALDH3A2	aldehyde dehydrogenase 3 family, member A2	1.2741571	0.00555895
219116_s_at	55208	DCUN1D2	DCN1, defective in cullin neddylation 1, domain containing 2	-0.87763094	0.00557617
214909_s_at	23564	DDAH2	dimethylarginine dimethylaminohydrolase 2	-1.01270506	0.00559518
203347_s_at	22823	MTF2	metal response element binding transcription factor 2	-1.01299911	0.00559849
201739_at	6446	SGK1	serum/glucocorticoid regulated kinase 1	2.08552055	0.00559879
205552_s_at	4938	OAS1	2'-5'-oligoadenylate synthetase 1, 40/46kDa	1.96153137	0.00560157
1560443_at	NA	NA	NA	-0.83452017	0.00562153
225715_at	57521	RPTOR	regulatory associated protein of MTOR, complex 1	-0.8197301	0.00564733
201620_at	8720	MBTPS1	membrane-bound transcription factor peptidase, site 1	-0.67527128	0.00565392
229514_at	55668	GPATCH2L	G patch domain containing 2-like	-1.13383642	0.00565711
218826_at	54733	SLC35F2	solute carrier family 35, member F2	-1.26238215	0.00566048
212086_x_at	4000	LMNA	lamin A/C	1.2337989	0.00567234
212068_s_at	84726	PRRC2B	proline-rich coiled-coil 2B	-0.62594924	0.00567571
203282_at	2632	GBE1	glucan (1,4-alpha-), branching enzyme 1	-0.9615396	0.00568478
213942_at	1953	MEGF6	multiple EGF-like-domains 6	-0.86983311	0.00569979
222143_s_at	64419	MTMR14	myotubularin related protein 14	0.6004402	0.0057053
203823_at	5998	RGS3	regulator of G-protein signaling 3	-0.76858879	0.00570653
236738_at	401097	C3orf80	chromosome 3 open reading frame 80	-2.45302186	0.00570653
200629_at	7453	WARS	tryptophanyl-tRNA synthetase	0.88661848	0.00571502
215235_at	6709	SPTAN1	spectrin, alpha, non-erythrocytic 1	-0.76909961	0.00571919
210785_s_at	9473	THEMIS2	thymocyte selection associated family member 2	1.45247531	0.00572379
215049_x_at	9332	CD163	CD163 molecule	2.5791679	0.00572746
243037_at	NA	NA	NA	-1.43700675	0.00573167
56919_at	57599	WDR48	WD repeat domain 48	-0.6256792	0.00573463
224928_at	80854	SETD7	SET domain containing (lysine methyltransferase) 7	1.11265541	0.00574387
219582_at	79627	OGFRL1	opioid growth factor receptor-like 1	0.88959843	0.00575193
202146_at	3475	IFRD1	interferon-related developmental regulator 1	-0.94764171	0.00575534
223025_s_at	8907	AP1M1	adaptor-related protein complex 1, mu 1 subunit	0.72486524	0.00576226
213589_s_at	146712	B3GNTL1	UDP-GlcNAc:betaGal beta-1,3-N-acetylglucosaminyltransferase-like 1	-1.26086125	0.00577927
213746_s_at	2316	FLNA	filamin A, alpha	1.22261122	0.00580381
201622_at	27044	SND1	staphylococcal nuclease and tudor domain containing 1	-0.66912278	0.00580788
209558_s_at	9026	HIP1R	huntingtin interacting protein 1 related	-0.97638578	0.00581156
212920_at	5978	REST	RE1-silencing transcription factor	-0.80528869	0.00582016
242403_at	NA	NA	NA	-1.14683708	0.0058278
1553639_a_at	133522	PPARGC1B	peroxisome proliferator-activated receptor gamma, coactivator 1 beta	0.8381423	0.00582969
204163_at	11117	EMILIN1	elastin microfibril interfacer 1	-1.87041887	0.00583059
215359_x_at	51710 | 10106	ZNF44 | LOC1010601	zinc finger protein 44 | zinc finger protein ZnFP12	-1.34180269	0.00583927
210517_s_at	9590	AKAP12	A kinase (PRKA) anchor protein 12	-1.47714324	0.00584417
212566_at	4134	MAP4	microtubule-associated protein 4	-0.72441725	0.0058483

209367_at	6813	STXBP2	syntaxin binding protein 2	1.0453633	0.0058505
211743_s_at	5553	PRG2	proteoglycan 2, bone marrow (natural killer cell activator, eosinophil granule major ba	-2.63256394	0.00585637
211581_x_at	7940	LST1	leukocyte specific transcript 1	1.59271356	0.005864
201363_s_at	10625	IVNS1ABP	influenza virus NS1A binding protein	1.13113775	0.00586902
235818_at	284415	VSTM1	V-set and transmembrane domain containing 1	-2.14990486	0.00590528
211368_s_at	834	CASP1	caspase 1, apoptosis-related cysteine peptidase	1.90872117	0.00591778
236379_at	NA	NA	NA	-1.21341482	0.00593374
213887_s_at	5434	POLR2E	polymerase (RNA) II (DNA directed) polypeptide E, 25kDa	0.98280946	0.00594321
201636_at	8087	FXR1	fragile X mental retardation, autosomal homolog 1	-0.6406177	0.00595104
65718_at	25960	ADGRA2	adhesion G protein-coupled receptor A2	1.09150787	0.00596252
221971_x_at	653268 | 119(	AGAP7P | AGAP4 | AG	ArfGAP with GTPase domain, ankyrin repeat and PH domain 7, pseudogene | ArfGAP	-0.85937726	0.00597091
224764_at	57584	ARHGAP21	Rho GTPase activating protein 21	-1.59839443	0.00597342
212846_at	23076	RRP1B	ribosomal RNA processing 1B	-0.62491697	0.00597364
209379_s_at	54462	CCSER2	coiled-coil serine-rich protein 2	-0.88614532	0.00598154
207467_x_at	831	CAST	calpastatin	1.65312436	0.00598479
226103_at	91624	NEXN	nexilin (F actin binding protein)	1.55948996	0.00598756
201280_s_at	1601	DAB2	Dab, mitogen-responsive phosphoprotein, homolog 2 (Drosophila)	-1.25608088	0.00600339
229366_at	NA	NA	NA	-1.68327357	0.0060206
239171_at	NA	NA	NA	1.57237399	0.00603516
208614_s_at	2317	FLNB	filamin B, beta	1.59480215	0.00603658
233003_at	NA	NA	NA	0.83676346	0.00606005
212090_at	2907	GRINA	glutamate receptor, ionotropic, N-methyl D-aspartate-associated protein 1 (glutamate	0.91526792	0.00607146
212205_at	94239	H2AFV	H2A histone family, member V	-0.69342294	0.00608103
205229_s_at	1690	COCH	cochlin	-1.9005941	0.0060859
239930_at	2590	GALNT2	polypeptide N-acetylgalactosaminyltransferase 2	-1.31188008	0.00608655
203120_at	7159	TP53BP2	tumor protein p53 binding protein 2	-0.88360177	0.00608701
209238_at	6809	STX3	syntaxin 3	1.03847982	0.00611588
211144_x_at	445347 | 6967	TARP | TRGC2 | TRGV	TCR gamma alternate reading frame protein | T cell receptor gamma constant 2 | T c	-1.99690634	0.00611641
211367_s_at	834	CASP1	caspase 1, apoptosis-related cysteine peptidase	1.95833776	0.00612722
203021_at	6590	SLPI	secretory leukocyte peptidase inhibitor	-1.81184575	0.00612836
228974_at	342926	ZNF677	zinc finger protein 677	-1.23745169	0.00612918
203257_s_at	79096	C11orf49	chromosome 11 open reading frame 49	-0.68044519	0.00614403
214196_s_at	1200	TPP1	tripeptidyl peptidase I	1.18188668	0.0061472
1563473_at	NA	NA	NA	-2.24874963	0.00614928
227184_at	5724	PTAFR	platelet-activating factor receptor	1.24215823	0.00616484
208940_at	22929	SEPHS1	selenophosphate synthetase 1	-0.71686868	0.00620545
215646_s_at	1462	VCAN	versican	3.84041635	0.00621595
213625_at	387032	ZKSCAN4	zinc finger with KRAB and SCAN domains 4	-0.86001947	0.0062165
200831_s_at	6319	SCD	stearoyl-CoA desaturase (delta-9-desaturase)	0.7724779	0.00622771
218626_at	56478	EIF4ENIF1	eukaryotic translation initiation factor 4E nuclear import factor 1	-0.64271026	0.00623091
239179_at	NA	NA	NA	-1.3624311	0.00624777
223184_s_at	56894	AGPAT3	1-acylglycerol-3-phosphate O-acyltransferase 3	0.79326078	0.00625431
1562307_at	NA	NA	NA	-1.70409705	0.00625598
244778_x_at	NA	NA	NA	-0.82682909	0.00627895
205964_at	79088	ZNF426	zinc finger protein 426	-1.06548786	0.0062825
200803_s_at	7009	TMBIM6	transmembrane BAX inhibitor motif containing 6	0.69087535	0.00628963
200801_x_at	60	ACTB	actin, beta	0.47815127	0.00630033
221998_s_at	51231	VRK3	vaccinia related kinase 3	0.75054115	0.00630459
205052_at	549	AUH	AU RNA binding protein/enoyl-CoA hydratase	-0.83685691	0.00631557
209199_s_at	4208	MEF2C	myocyte enhancer factor 2C	1.54330129	0.00636711
212419_at	219654	ZCCHC24	zinc finger, CCHC domain containing 24	0.84507158	0.00636757
202443_x_at	4853	NOTCH2	notch 2	1.32716117	0.00637632
204849_at	10732	TCFL5	transcription factor-like 5 (basic helix-loop-helix)	-1.23328368	0.00642811
204491_at	5144	PDE4D	phosphodiesterase 4D, cAMP-specific	-1.06112528	0.00645691
244015_at	NA	NA	NA	-1.06471011	0.00645889
228009_x_at	30834	ZNRD1	zinc ribbon domain containing 1	-0.75230699	0.00646021
202012_s_at	2132	EXT2	exostosin glycosyltransferase 2	-0.66599008	0.00648371
208997_s_at	7351	UCP2	uncoupling protein 2 (mitochondrial, proton carrier)	1.40279487	0.00654083
206245_s_at	10625	IVNS1ABP	influenza virus NS1A binding protein	1.1571392	0.00656622
205035_at	9150	CTDP1	CTD (carboxy-terminal domain, RNA polymerase II, polypeptide A) phosphatase, sub	-0.62909978	0.00656758
1555756_a_at	64581	CLEC7A	C-type lectin domain family 7, member A	3.01856994	0.00657637
202238_s_at	4837	NNMT	nicotinamide N-methyltransferase	-1.51861376	0.00657726
229391_s_at	441168	FAM26F	family with sequence similarity 26, member F	-2.15507458	0.00658936
203985_at	7988	ZNF212	zinc finger protein 212	-0.59677522	0.00659791
203298_s_at	3720	JARID2	jumonji, AT rich interactive domain 2	-0.77098085	0.00660602
205568_at	366	AQP9	aquaporin 9	2.00759568	0.00661062
212223_at	3423	IDS	iduronate 2-sulfatase	-1.09868796	0.00661578
219947_at	50856	CLEC4A	C-type lectin domain family 4, member A	2.42404029	0.00663447
203074_at	728113 | 6531	ANXA8L1 | ANXA8	annexin A8-like 1 | annexin A8	-2.26487816	0.00664018
204620_s_at	1462	VCAN	versican	3.39284458	0.00664848
221538_s_at	5361	PLXNA1	plexin A1	1.30592974	0.00665493
208893_s_at	1848	DUSP6	dual specificity phosphatase 6	1.98429106	0.00666226
220307_at	51744	CD244	CD244 molecule, natural killer cell receptor 2B4	-1.19041686	0.00666252
213218_at	7741	ZSCAN26	zinc finger and SCAN domain containing 26	-0.79646695	0.00667539
213772_s_at	23062	GGA2	golgi-associated, gamma adaptin ear containing, ARF binding protein 2	0.84847456	0.00667573
217969_at	738	VPS51	vacuolar protein sorting 51 homolog (S. cerevisiae)	-0.60431474	0.00670678
240655_at	NA	NA	NA	-1.26812076	0.00671835
235568_at	199675	MCEMP1	mast cell-expressed membrane protein 1	1.84192084	0.00671893
218709_s_at	51098	IFT52	intraflagellar transport 52	-0.66332972	0.00671955
202736_s_at	25804	LSM4	LSM4 homolog, U6 small nuclear RNA associated (S. cerevisiae)	0.9358791	0.0067472
204106_at	7016	TESK1	testis-specific kinase 1	-0.80074027	0.00675216
1555730_a_at	1072	CFL1	cofilin 1 (non-muscle)	1.204918	0.00676474
204045_at	9338	TCEAL1	transcription elongation factor A (SII)-like 1	-0.94324127	0.00676482
212547_at	8019	BRD3	bromodomain containing 3	-0.54397687	0.00676914
205928_at	10224	ZNF443	zinc finger protein 443	-0.84077297	0.00677062
225469_at	144363	LYRM5	LYR motif containing 5	-0.84963065	0.00677281
212921_at	56950	SMYD2	SET and MYND domain containing 2	-1.12217119	0.00678015
208655_at	10983	CCNI	cyclin I	-0.55816542	0.00678569
218259_at	57496	MKL2	MKL/myocardin-like 2	-1.07213099	0.00681337
213036_x_at	489	ATP2A3	ATPase, Ca++ transporting, ubiquitous	-0.93839815	0.00681934
232614_at	NA	NA	NA	-1.27409408	0.00682782
205016_at	7039	TGFA	transforming growth factor, alpha	-1.22925015	0.00683674
239193_at	8939	FUBP3	far upstream element (FUSE) binding protein 3	-0.89538965	0.00685264
222632_s_at	54585	LZTFL1	leucine zipper transcription factor-like 1	-0.8843181	0.00686358
230795_at	NA	NA	NA	-1.23891419	0.0068816
228105_at	NA	NA	NA	-0.74511027	0.00689471
238924_at	399761	BMS1P5	BMS1 pseudogene 5	-2.12649726	0.00690632
241093_at	NA	NA	NA	-1.03272061	0.0069192
200852_x_at	2783	GNB2	guanine nucleotide binding protein (G protein), beta polypeptide 2	0.80078341	0.00692277
238058_at	150381	PRR34-AS1	PRR34 antisense RNA 1	1.12785375	0.00693086
225343_at	283578	TMED8	transmembrane emp24 protein transport domain containing 8	-0.7403288	0.00697389
232286_at	NA	NA	NA	2.05876335	0.00697497
219644_at	51134	CEP83	centrosomal protein 83kDa	-1.02839681	0.00698681
210794_s_at	55384	MEG3	maternally expressed 3 (non-protein coding)	-1.69148836	0.00699401

205480_s_at	7360	UGP2	UDP-glucose pyrophosphorylase 2	-0.99719635	0.00701068
202924_s_at	5326	PLAGL2	pleiomorphic adenoma gene-like 2	1.01331831	0.00702017
221799_at	54480	CHPF2	chondroitin polymerizing factor 2	-0.64580807	0.00705197
236338_at	NA	NA	NA	1.74379136	0.00705698
229748_x_at	100132288 | 7	TEKT4P2 | MAFIP | LO	tektin 4 pseudogene 2 | MAFF interacting protein (pseudogene) | tektin 4 pseudogen	-1.18349056	0.0070594
226840_at	9555	H2AFY	H2A histone family, member Y	-1.21232049	0.00706127
32402_s_at	8189	SYMPK	symplekin	0.86275779	0.0070763
220774_at	54808	DYM	dymeclin	-0.74548625	0.00710087
210405_x_at	8795	TNFRSF10B	tumor necrosis factor receptor superfamily, member 10b	0.95206538	0.0071151
236814_at	4194	MDM4	MDM4, p53 regulator	-0.83243912	0.00711764
202806_at	1627	DBN1	drebrin 1	-1.17043095	0.00712418
204735_at	5141	PDE4A	phosphodiesterase 4A, cAMP-specific	1.1697537	0.00712464
221483_s_at	10776	ARPP19	cAMP-regulated phosphoprotein, 19kDa	-0.61998101	0.00712536
201490_s_at	10105	PPIF	peptidylprolyl isomerase F	1.07514904	0.00714256
221899_at	10443	N4BP2L2	NEDD4 binding protein 2-like 2	-0.86075244	0.00716786
209813_x_at	6983 | 6967 |	TRGV9 | TRGC2 | TAR	T cell receptor gamma variable 9 | T cell receptor gamma constant 2 | TCR gamma a	-1.90473055	0.00717358
213915_at	4818	NKG7	natural killer cell granule protein 7	1.35972665	0.00717633
209346_s_at	55361	PI4K2A	phosphatidylinositol 4-kinase type 2 alpha	0.68827923	0.00718384
229530_at	2982	GUCY1A3	guanylate cyclase 1, soluble, alpha 3	-2.43436944	0.00719062
232520_s_at	55968	NSFL1C	NSFL1 (p97) cofactor (p47)	0.56139752	0.0071925
212119_at	23433	RHOQ	ras homolog family member Q	0.98260145	0.00719251
222623_s_at	51193	ZNF639	zinc finger protein 639	-0.86623294	0.00719873
224865_at	84188	FAR1	fatty acyl CoA reductase 1	0.80838318	0.00722084
235786_at	NA	NA	NA	-0.95296101	0.00724481
220998_s_at	81622	UNC93B1	unc-93 homolog B1 (C. elegans)	0.78628198	0.00725218
233995_at	NA	NA	NA	-1.05445254	0.0072532
1555896_a_at	8751	ADAM15	ADAM metallopeptidase domain 15	0.54794842	0.00726688
208670_s_at	23741	EID1	EP300 interacting inhibitor of differentiation 1	1.03099475	0.00726911
230389_at	23048	FNBP1	formin binding protein 1	-1.23497331	0.00727007
206298_at	58504	ARHGAP22	Rho GTPase activating protein 22	-2.11788491	0.00727829
239228_at	NA	NA	NA	-1.07689204	0.00729436
214964_at	NA	NA	NA	-1.56677438	0.00729536
216920_s_at	445347 | 6967	TARP | TRGC2 | TRGV	TCR gamma alternate reading frame protein | T cell receptor gamma constant 2 | T c	-1.87022906	0.00729633
212192_at	115207	KCTD12	potassium channel tetramerization domain containing 12	3.15160443	0.0073007
224813_at	8976	WASL	Wiskott-Aldrich syndrome-like	-0.82259414	0.00730862
226372_at	50515	CHST11	carbohydrate (chondroitin 4) sulfotransferase 11	-0.92547213	0.00730903
239762_at	286437	LOC286437	uncharacterized LOC286437	-1.02590438	0.00731031
238896_at	6483	ST3GAL2	ST3 beta-galactoside alpha-2,3-sialyltransferase 2	-0.51067147	0.00731176
219225_at	79605	PGBD5	piggyBac transposable element derived 5	-1.34345125	0.00731944
218086_at	56654	NPDC1	neural proliferation, differentiation and control, 1	-1.17383775	0.0073207
227284_at	90321	ZNF766	zinc finger protein 766	-0.89442608	0.00732321
237945_at	NA	NA	NA	-2.11125384	0.00732887
1557797_a_at	NA	NA	NA	1.23847132	0.00733419
224998_at	146223	CMTM4	CKLF-like MARVEL transmembrane domain containing 4	1.34531785	0.00734032
210269_s_at	8227	AKAP17A	A kinase (PRKA) anchor protein 17A	-0.67886557	0.00734326
205067_at	3553	IL1B	interleukin 1, beta	-1.28464222	0.00735749
206011_at	834	CASP1	caspase 1, apoptosis-related cysteine peptidase	1.80445192	0.00736474
227239_at	84668	FAM126A	family with sequence similarity 126, member A	1.02233599	0.00736754
223413_s_at	55646	LYAR	Ly1 antibody reactive	1.08869401	0.00742043
217234_s_at	7430	EZR	ezrin	1.74151409	0.00743932
214672_at	23093	TTLL5	tubulin tyrosine ligase-like family member 5	-0.74172529	0.00746448
1554599_x_at	3980	LIG3	ligase III, DNA, ATP-dependent	-0.66451091	0.00747687
239886_at	NA	NA	NA	-1.11241959	0.00751572
212952_at	811	CALR	calreticulin	-1.38023633	0.00752478
222364_at	23446	SLC44A1	solute carrier family 44 (choline transporter), member 1	1.14792592	0.00753469
235309_at	6210	RPS15A	ribosomal protein S15a	-0.88343883	0.00753927
207996_s_at	753	LDLRAD4	low density lipoprotein receptor class A domain containing 4	-1.7468125	0.00754489
226867_at	55667	DENND4C	DENN/MADD domain containing 4C	-1.70139912	0.00754491
1553535_a_at	5905	RANGAP1	Ran GTPase activating protein 1	1.00937146	0.00754825
31826_at	23307	FKBP15	FK506 binding protein 15, 133kDa	0.66033741	0.00754835
201242_s_at	481	ATP1B1	ATPase, Na+/K+ transporting, beta 1 polypeptide	-2.35657947	0.00755408
227235_at	2982	GUCY1A3	guanylate cyclase 1, soluble, alpha 3	-2.53995086	0.00755451
211979_at	57720	GPR107	G protein-coupled receptor 107	-0.64675995	0.00755475
230408_at	10336	PCGF3	polycomb group ring finger 3	-1.03762572	0.00756963
221893_s_at	90956	ADCK2	aarF domain containing kinase 2	0.92366473	0.00757348
228176_at	1903	S1PR3	sphingosine-1-phosphate receptor 3	1.54958913	0.00757737
222760_at	80139	ZNF703	zinc finger protein 703	1.14265673	0.00757984
231406_at	80228	ORAI2	ORAI calcium release-activated calcium modulator 2	-0.8845634	0.00758265
1555759_a_at	6352	CCL5	chemokine (C-C motif) ligand 5	1.37096984	0.00758463
202902_s_at	1520	CTSS	cathepsin S	2.14407739	0.00758689
235513_at	NA	NA	NA	-1.16888071	0.00758985
209294_x_at	8795	TNFRSF10B	tumor necrosis factor receptor superfamily, member 10b	1.02245756	0.00759062
1555916_at	285367	RPUSD3	RNA pseudouridylate synthase domain containing 3	-0.68745918	0.00760834
225461_at	79813	EHMT1	euchromatic histone-lysine N-methyltransferase 1	-0.60953563	0.0076123
222431_at	10927	SPIN1	spindlin 1	-0.94816009	0.0076205
215707_s_at	5621	PRNP	prion protein	1.24259641	0.00765853
235493_at	NA	NA	NA	-0.74144994	0.00767072
205249_at	1959	EGR2	early growth response 2	1.1969915	0.00767222
225129_at	221184	CPNE2	copine II	-1.43026432	0.00768525
227084_at	1837	DTNA	dystrobrevin, alpha	1.36244642	0.00768547
1568964_x_at	6693	SPN	sialophorin	-1.7023852	0.00769035
202909_at	9852	EPM2AIP1	EPM2A (laforin) interacting protein 1	-0.59917909	0.0077188
204453_at	7637	ZNF84	zinc finger protein 84	-0.85453441	0.00772532
220760_x_at	79788	ZNF665	zinc finger protein 665	-0.77650117	0.0077414
201968_s_at	5236	PGM1	phosphoglucomutase 1	-0.95612704	0.00774236
208983_s_at	5175	PECAM1	platelet/endothelial cell adhesion molecule 1	1.70913421	0.0077428
1565881_at	NA	NA	NA	-1.08379779	0.00775179
200711_s_at	6500	SKP1	S-phase kinase-associated protein 1	-0.56731459	0.00775829
208246_x_at	NA	NA	NA	-0.89918934	0.00777597
201039_s_at	5886	RAD23A	RAD23 homolog A (S. cerevisiae)	1.18846558	0.00777959
203645_s_at	9332	CD163	CD163 molecule	2.51732174	0.0077804
242506_at	NA	NA	NA	-0.79447194	0.00779217
243296_at	10135	NAMPT	nicotinamide phosphoribosyltransferase	1.76179536	0.00779453
205745_x_at	6868	ADAM17	ADAM metallopeptidase domain 17	-0.6771212	0.0078257
235112_at	NA	NA	NA	-1.62520767	0.00783481
202447_at	1666	DECR1	2,4-dienoyl CoA reductase 1, mitochondrial	0.80688983	0.00786008
1569490_at	64778	FNDC3B	fibronectin type III domain containing 3B	-1.34541099	0.00786636
234341_x_at	91548	LOC91548	uncharacterized LOC91548	-0.58752967	0.00787685
223912_s_at	2055	CLN8	ceroid-lipofuscinosis, neuronal 8 (epilepsy, progressive with mental retardation)	1.01106556	0.00791985
234981_x_at	134147	CMBL	carboxymethylenebutenolidase homolog (Pseudomonas)	-0.83172177	0.0079341
222988_s_at	252839	TMEM9	transmembrane protein 9	-0.76447135	0.00793708
202656_s_at	9792	SERTAD2	SERTA domain containing 2	-0.80567157	0.00794128
226039_at	11320	MGAT4A	mannosyl (alpha-1,3-)-glycoprotein beta-1,4-N-acetylglucosaminyltransferase, isozyr	-0.88151224	0.00796091
213577_at	6713	SQLE	squalene epoxidase	0.76446265	0.00797714

1557626_at	NA	NA	NA	-1.06814362	0.00799974
206015_s_at	22887	FOXJ3	forkhead box J3	-0.63805896	0.00801952
230416_at	118987	PDZD8	PDZ domain containing 8	-1.49734652	0.00802366
230618_s_at	NA	NA	NA	-1.19595746	0.00803344
200757_s_at	813	CALU	calumenin	-0.66908659	0.00803824
221073_s_at	10392	NOD1	nucleotide-binding oligomerization domain containing 1	-0.55534788	0.00805744
201791_s_at	1717	DHCR7	7-dehydrocholesterol reductase	-0.89622984	0.00806231
202687_s_at	8743	TNFSF10	tumor necrosis factor (ligand) superfamily, member 10	1.39760581	0.00806947
239516_at	NA	NA	NA	-0.92897808	0.0080779
220467_at	NA	NA	NA	-1.55988533	0.00808341
1557145_at	11329	STK38	serine/threonine kinase 38	-1.0917942	0.00810497
207992_s_at	272	AMPD3	adenosine monophosphate deaminase 3	-0.87530693	0.00811976
215146_s_at	23331	TTC28	tetratricopeptide repeat domain 28	-1.46093113	0.00813204
227158_at	112487	DTD2	D-tyrosyl-tRNA deacylase 2 (putative)	-0.90259251	0.00814833
223032_x_at	27166	PRELID1	PRELI domain containing 1	0.70384931	0.00819705
1557804_at	NA	NA	NA	-0.72175234	0.00820826
225317_at	84320	ACBD6	acyl-CoA binding domain containing 6	-0.79331564	0.0082114
213587_s_at	155066	ATP6V0E2	ATPase, H+ transporting V0 subunit e2	1.06938183	0.00821584
207341_at	5657	PRTN3	proteinase 3	-2.59841692	0.00822357
238042_at	NA	NA	NA	-0.68950634	0.00822845
213056_at	23150	FRMD4B	FERM domain containing 4B	-1.13243645	0.00822975
201418_s_at	6659	SOX4	SRY (sex determining region Y)-box 4	-0.98157524	0.0082337
233017_x_at	NA	NA	NA	-0.81415199	0.00823994
205002_at	27245	AHDC1	AT hook, DNA binding motif, containing 1	-0.81066979	0.00824639
226352_at	133746	JMY	junction mediating and regulatory protein, p53 cofactor	-2.18576067	0.00825766
221253_s_at	81567 | 10052	TXNDC5 | BLOC1S5-T	thioredoxin domain containing 5 (endoplasmic reticulum) | BLOC1S5-TXNDC5 readthr	-0.87780294	0.00826357
219233_s_at	55876	GSDMB	gasdermin B	-0.95913333	0.00826803
219151_s_at	11158 | 1115	RABL2B | RABL2A	RAB, member of RAS oncogene family-like 2B | RAB, member of RAS oncogene famil	-0.94929169	0.00827156
226232_at	10220	GDF11	growth differentiation factor 11	-0.79819597	0.00828591
218099_at	55852	TEX2	testis expressed 2	0.88747254	0.00833192
208621_s_at	7430	EZR	ezrin	1.77712319	0.00835064
208752_x_at	4673	NAP1L1	nucleosome assembly protein 1-like 1	-0.75113028	0.00836544
239651_at	51433	ANAPC5	anaphase promoting complex subunit 5	-1.09914202	0.00836778
226957_x_at	10928	RALBP1	ralA binding protein 1	-0.77684207	0.00837488
218996_at	29844	TFPT	TCF3 (E2A) fusion partner (in childhood Leukemia)	-0.48320101	0.0083802
244234_at	NA	NA	NA	-0.69448279	0.00840255
202814_s_at	10614	HEXIM1	hexamethylene bis-acetamide inducible 1	0.85305766	0.00840657
202211_at	26286	ARFGAP3	ADP-ribosylation factor GTPase activating protein 3	-0.6389989	0.00840836
223814_at	51095	TRNT1	tRNA nucleotidyl transferase, CCA-adding, 1	-1.22566266	0.0084097
208920_at	6717	SRI	sorcin	-1.17611889	0.0084195
206081_at	9187	SLC24A1	solute carrier family 24 (sodium/potassium/calcium exchanger), member 1	-1.16970811	0.00842125
200866_s_at	5660	PSAP	prosaposin	1.39435018	0.00844
208622_s_at	7430	EZR	ezrin	1.57366829	0.00844368
225262_at	2355	FOSL2	FOS-like antigen 2	1.56360947	0.00844768
202241_at	10221	TRIB1	tribbles pseudokinase 1	1.58799514	0.00845306
209362_at	9412 | 10192	MED21 | LOC1019286	mediator complex subunit 21 | uncharacterized LOC101928625	-1.11994354	0.00846658
203490_at	2000	ELF4	E74-like factor 4 (ets domain transcription factor)	0.79318178	0.00847216
233399_x_at	286101	ZNF252P	zinc finger protein 252, pseudogene	-0.78015966	0.00847396
1554343_a_at	26228	STAP1	signal transducing adaptor family member 1	-1.2016346	0.00849707
204425_at	393	ARHGAP4	Rho GTPase activating protein 4	1.49338265	0.00850217
200897_s_at	23022	PALLD	palladin, cytoskeletal associated protein	1.47783207	0.0085135
207551_s_at	10943	MSL3	male-specific lethal 3 homolog (Drosophila)	0.68510915	0.00851841
204328_at	11322	TMC6	transmembrane channel-like 6	0.79184746	0.00853329
226833_at	124637	CYB5D1	cytochrome b5 domain containing 1	-0.66316796	0.00853446
226111_s_at	25946	ZNF385A	zinc finger protein 385A	0.74990775	0.0085671
218473_s_at	79709	COLGALT1	collagen beta(1-O)galactosyltransferase 1	0.64731908	0.00858612
203315_at	8440	NCK2	NCK adaptor protein 2	-0.64169773	0.00859736
218421_at	64781	CERK	ceramide kinase	-0.79566558	0.00860312
238553_at	399761 | 4141	BMS1P5 | AGAP6 | AG	BMS1 pseudogene 5 | ArfGAP with GTPase domain, ankyrin repeat and PH domain 6	-1.81944241	0.00860677
203591_s_at	1441	CSF3R	colony stimulating factor 3 receptor (granulocyte)	-1.33073287	0.00862294
216903_s_at	10367	MICU1	mitochondrial calcium uptake 1	0.58284296	0.00863468
232180_at	7360	UGP2	UDP-glucose pyrophosphorylase 2	-1.50768275	0.00863775
203988_s_at	2530	FUT8	fucosyltransferase 8 (alpha (1,6) fucosyltransferase)	-0.82164827	0.00864465
1553133_at	203228	C9orf72	chromosome 9 open reading frame 72	1.35350593	0.00865447
228582_x_at	378938	MALAT1	metastasis associated lung adenocarcinoma transcript 1 (non-protein coding)	1.32696053	0.00867212
234488_s_at	64396 | 6439	GMCL1P1 | GMCL1	germ cell-less, spermatogenesis associated 1 pseudogene 1 | germ cell-less, spermat	-1.01384416	0.00870262
216109_at	23389	MED13L	mediator complex subunit 13-like	-1.27848649	0.00870895
240344_x_at	90624	LYRM7	LYR motif containing 7	-0.91132904	0.00871595
228993_s_at	92482	BBIP1	BBSome interacting protein 1	-0.88929318	0.00874089
227860_at	56265	CPXM1	carboxypeptidase X (M14 family), member 1	-1.49827042	0.00875808
204441_s_at	23649	POLA2	polymerase (DNA directed), alpha 2, accessory subunit	0.66183737	0.00876134
212616_at	80205	CHD9	chromodomain helicase DNA binding protein 9	-0.69002605	0.00877587
202826_at	6692	SPINT1	serine peptidase inhibitor, Kunitz type 1	0.91747917	0.00877706
232759_at	NA	NA	NA	-0.91128038	0.00877903
228999_at	1106	CHD2	chromodomain helicase DNA binding protein 2	-1.22564207	0.00879752
203627_at	3480	IGF1R	insulin-like growth factor 1 receptor	-1.89025178	0.00880014
204713_s_at	2153	F5	coagulation factor V (proaccelerin, labile factor)	1.09556458	0.00880338
224471_s_at	8945	BTRC	beta-transducin repeat containing E3 ubiquitin protein ligase	-0.68798626	0.00880348
203805_s_at	2175	FANCA	Fanconi anemia, complementation group A	0.70644414	0.00881156
230759_at	57231	SNX14	sorting nexin 14	-0.8600338	0.00883035
212967_x_at	4673	NAP1L1	nucleosome assembly protein 1-like 1	-0.77154369	0.00884884
201156_s_at	5878	RAB5C	RAB5C, member RAS oncogene family	0.86277813	0.00885545
236703_at	NA	NA	NA	-1.13982613	0.00886634
242297_at	6239	RREB1	ras responsive element binding protein 1	-0.91502623	0.00887184
204537_s_at	2564 | 40700	GABRE | MIR224 | MI	gamma-aminobutyric acid (GABA) A receptor, epsilon | microRNA 224 | microRNA 45	-2.42060283	0.00887303
243524_at	NA	NA	NA	-0.72738417	0.00887426
204896_s_at	5734	PTGER4	prostaglandin E receptor 4 (subtype EP4)	0.86293711	0.00890104
38671_at	23129	PLXND1	plexin D1	-0.76257026	0.00893031
215268_at	643314	KIAA0754	KIAA0754	-0.96517987	0.00894083
1560156_at	NA	NA	NA	-1.00093401	0.00895753
236045_x_at	NA	NA	NA	-1.78626483	0.0089613
226390_at	134429	STARD4	StAR-related lipid transfer (START) domain containing 4	1.13006131	0.00896565
201919_at	55186	SLC25A36	solute carrier family 25 (pyrimidine nucleotide carrier), member 36	-0.68319515	0.00896816
215640_at	23102	TBC1D2B	TBC1 domain family, member 2B	-0.7728047	0.00897783
222416_at	5832	ALDH18A1	aldehyde dehydrogenase 18 family, member A1	-0.8867215	0.00898962
203042_at	3920	LAMP2	lysosomal-associated membrane protein 2	-0.99659592	0.00899283
222640_at	1788	DNMT3A	DNA (cytosine-5-)-methyltransferase 3 alpha	-0.95980443	0.00901125
203817_at	2983	GUCY1B3	guanylate cyclase 1, soluble, beta 3	-1.43877924	0.00902145
223058_at	83641	FAM107B	family with sequence similarity 107, member B	1.34300149	0.00904318
201522_x_at	6638 | 8926	SNRPN | SNURF	small nuclear ribonucleoprotein polypeptide N | SNRPN upstream reading frame	1.67755613	0.00904564
225316_at	84879	MFSD2A	major facilitator superfamily domain containing 2A	0.9533082	0.00907778
201416_at	6659	SOX4	SRY (sex determining region Y)-box 4	-1.42596479	0.00908468
207624_s_at	6103	RPGR	retinitis pigmentosa GTPase regulator	-1.13101397	0.0091193
229312_s_at	80318	GKAP1	G kinase anchoring protein 1	-1.0868508	0.00911955

201184_s_at	1108	CHD4	chromodomain helicase DNA binding protein 4	-0.48404056	0.00913013
203313_s_at	7050	TGIF1	TGFB-induced factor homeobox 1	-1.15507712	0.00917253
203420_at	51439	FAM8A1	family with sequence similarity 8, member A1	-0.70986268	0.00920762
223220_s_at	83666	PARP9	poly (ADP-ribose) polymerase family, member 9	0.96289748	0.00920947
212675_s_at	23177	CEP68	centrosomal protein 68kDa	-1.00286801	0.00922008
208944_at	7048	TGFBR2	transforming growth factor, beta receptor II (70/80kDa)	-1.05767744	0.00922919
204011_at	10253	SPRY2	sprouty homolog 2 (Drosophila)	-1.86943456	0.00923839
202105_at	3476	IGBP1	immunoglobulin (CD79A) binding protein 1	-0.57311446	0.00925268
220711_at	NA	NA	NA	-0.80145332	0.00925631
226055_at	27106	ARRDC2	arrestin domain containing 2	0.8601301	0.0092572
235109_at	84327	ZBED3	zinc finger, BED-type containing 3	-1.20056055	0.00925837
218825_at	51162	EGFL7	EGF-like-domain, multiple 7	-0.82679111	0.00930233
238595_at	NA	NA	NA	1.53749729	0.00931076
212663_at	23307	FKBP15	FK506 binding protein 15, 133kDa	0.66317133	0.00931333
226893_at	27	ABL2	ABL proto-oncogene 2, non-receptor tyrosine kinase	-0.74632139	0.00933577
217719_at	51386	EIF3L	eukaryotic translation initiation factor 3, subunit L	-0.62984453	0.00933637
229097_at	81624	DIAPH3	diaphanous-related formin 3	0.9608003	0.00936771
223828_s_at	85329	LGALS12	lectin, galactoside-binding, soluble, 12	-1.90089684	0.00937698
1556613_s_at	286148	DPY19L4	dpy-19-like 4 (C. elegans)	-1.11190747	0.00937939
215898_at	23093	TTLL5	tubulin tyrosine ligase-like family member 5	-0.9051163	0.00938054
230528_s_at	65996	CENPBD1P1	CENPBD1 pseudogene 1	-1.03884738	0.00938054
221588_x_at	4329	ALDH6A1	aldehyde dehydrogenase 6 family, member A1	-0.60279145	0.00938173
244872_at	5928	RBBP4	retinoblastoma binding protein 4	-1.04917418	0.00939154
243046_at	NA	NA	NA	-0.91992596	0.00939725
219906_at	55096	EBLN2	endogenous Bornavirus-like nucleoprotein 2	-0.67064653	0.00940318
210146_x_at	10288	LILRB2	leukocyte immunoglobulin-like receptor, subfamily B (with TM and ITIM domains), me	2.23955093	0.00940955
1552388_at	80274 | 15037	SCUBE1 | FLJ30901	signal peptide, CUB domain, EGF-like 1 | uncharacterized protein FLJ30901	0.99808255	0.00940957
1554442_at	7439	BEST1	bestrophin 1	0.64969692	0.00940995
201461_s_at	9261	MAPKAPK2	mitogen-activated protein kinase-activated protein kinase 2	1.35828783	0.00940998
208450_at	3957	LGALS2	lectin, galactoside-binding, soluble, 2	2.12421849	0.0094106
218338_at	1911	PHC1	polyhomeotic homolog 1 (Drosophila)	-0.69936024	0.00941676
226811_at	54855	FAM46C	family with sequence similarity 46, member C	-2.49294995	0.00944546
211048_s_at	9601	PDIA4	protein disulfide isomerase family A, member 4	-0.8998919	0.00945378
223639_s_at	30834	ZNRD1	zinc ribbon domain containing 1	-0.77969181	0.00945485
208018_s_at	3055	HCK	HCK proto-oncogene, Src family tyrosine kinase	1.97494066	0.00947936
202426_s_at	6256	RXRA	retinoid X receptor, alpha	0.9751378	0.0094868
241154_x_at	NA	NA	NA	1.03402695	0.00949675
236924_at	NA	NA	NA	-1.28528204	0.0095043
204446_s_at	240	ALOX5	arachidonate 5-lipoxygenase	-2.32563312	0.00951033
227645_at	23533	PIK3R5	phosphoinositide-3-kinase, regulatory subunit 5	0.80019818	0.00951271
235925_at	NA	NA	NA	-0.7949527	0.00953168
213035_at	23243	ANKRD28	ankyrin repeat domain 28	-1.49590319	0.00953948
228415_at	8905	AP1S2	adaptor-related protein complex 1, sigma 2 subunit	1.30376847	0.00955466
239577_at	NA	NA	NA	-1.05096972	0.00957696
238653_at	9860	LRIG2	leucine-rich repeats and immunoglobulin-like domains 2	-1.20534332	0.00957867
215537_x_at	23564	DDAH2	dimethylarginine dimethylaminohydrolase 2	-0.84339273	0.00959789
214113_s_at	9939	RBM8A	RNA binding motif protein 8A	-0.59549665	0.00962857
229040_at	100505746	ITGB2-AS1	ITGB2 antisense RNA 1	1.17920866	0.0096382
1554406_a_at	64581	CLEC7A	C-type lectin domain family 7, member A	1.75282323	0.00964017
212772_s_at	20	ABCA2	ATP-binding cassette, sub-family A (ABC1), member 2	-0.66659639	0.00964237
224807_at	57655	GRAMD1A	GRAM domain containing 1A	-0.74891142	0.00964759
224800_at	57590	WDFY1	WD repeat and FYVE domain containing 1	0.94330511	0.00966248
213539_at	915	CD3D	CD3d molecule, delta (CD3-TCR complex)	-1.83077814	0.00969556
202375_at	9871	SEC24D	SEC24 family member D	-1.01045404	0.00971478
235093_at	5194	PEX13	peroxisomal biogenesis factor 13	-0.83154145	0.00973678
219033_at	79668	PARP8	poly (ADP-ribose) polymerase family, member 8	1.57683655	0.00975346
230244_at	389084	C2orf82	chromosome 2 open reading frame 82	-1.9084951	0.00975642
200833_s_at	5908	RAP1B	RAP1B, member of RAS oncogene family	-0.64996805	0.00975722
1569401_at	160364	CLEC12A	C-type lectin domain family 12, member A	1.06981164	0.00975954
231323_at	5690	PSMB2	proteasome (prosome, macropain) subunit, beta type, 2	-1.2821581	0.00976795
240238_at	NA	NA	NA	-0.59615132	0.00980143
212242_at	7277	TUBA4A	tubulin, alpha 4a	1.1843053	0.00985698
223168_at	58480	RHOU	ras homolog family member U	2.04622783	0.00986363
213213_at	11083	DIDO1	death inducer-obliterator 1	-0.81129032	0.00988289
215612_at	NA	NA	NA	-0.69533873	0.00988883
239453_at	NA	NA	NA	-1.38047881	0.00990299
242920_at	NA	NA	NA	-0.73226065	0.00990855
215382_x_at	7177	TPSAB1	tryptase alpha/beta 1	-2.108838	0.00991209
235385_at	55016	MARCH1	membrane-associated ring finger (C3HC4) 1, E3 ubiquitin protein ligase	2.40381814	0.00992543
229519_at	8087	FXR1	fragile X mental retardation, autosomal homolog 1	-0.60847501	0.00995832
223245_at	55342	STRBP	spermatid perinuclear RNA binding protein	-0.81759719	0.00996105
244803_at	NA	NA	NA	-0.87249097	0.00997048
204496_at	29966	STRN3	striatin, calmodulin binding protein 3	-0.68379913	0.00998988
227567_at	100499466	LINC00674	long intergenic non-protein coding RNA 674	-1.35496482	0.00999133
221619_s_at	23787	MTCH1	mitochondrial carrier 1	0.80986528	0.00999501
