# Supplementary material for: SGK1 repression by WT1 may confer a survival advantage to leukemic cells under stress conditions
Source: Ann Hematol. 2025 Jul 4;104(7):3655–67. doi: 10.1007/s00277-025-06458-z (PMC12334445; doi:10.1007/s00277-025-06458-z)
Supplement: Supplementary file 6 — Supplementary file6 (PDF 30 KB) [file 277_2025_6458_MOESM6_ESM.pdf]

Supplementary Table 4: Clinical and biological characteristics of the AML patients used in the RT-qPCR assays.

Patient #	WT1 status	Sex	Age (years)	[WT1/ABL1] x 10^4 WT1 copies	SGK1 $\Delta\Delta C_T$	Cytogenetics	Molecular
1	WT1 low	M	69	30	5.85	46,XY 5q-/+der(1)5q-6p-16	FLT3-NPM-CEBPA-MLL-
2	WT1 low	M		187	0.16	46,XY	FLT3-NPM-CEBPA-MLL-
3	WT1 low	M		9.2	0.46	47,XY,+8/46,XY	FLT3-NPM-CEBPA-MLL-
4	WT1 low	F	61	3.7	0.56	47,XX,+19 / 48,XX,+8,+19 / 46,XX	FLT3-NPM-CEBPA-MLL-
5	WT1 low	M	67	20	4.46	46,XY	FLT3-NPM-CEBPA-MLL-
6	WT1 low	F		67	0.07	50,XX,+2,3p+,+6,+8,+19/46,XX	FLT3-NPM-CEBPA-MLL-
7	WT1 low	M		153	1.59	46,XY	FLT3-NPM-CEBPA-MLL-
8	WT1 low	M		138	21.89	46,XY,del(5)(q31),+mar/46,XY	FLT3-NPM-CEBPA-MLL-
9	WT1 low	F	34	0.1	1.62	46,XX	FLT3-NPM-CEBPA-MLL-
10	WT1 low	M		0.1	2.36	46,XY	nd
11	WT1 low	M		32	5.38	46,XY,7q-/46,XY	FLT3-NPM-CEBPA-MLL-
12	WT1 low	M		18	0.44	46,XY	FLT3-NPM-CEBPA-MLL-
13	WT1 low	M		12	0.68	46,XY	FLT3-NPM-CEBPA-MLL-
14	WT1 low	M		42	0.62	46,XY	FLT3-NPM-CEBPA-MLL-
15	WT1 low	F		191	7.63	46,XX	FLT3-NPM-CEBPA+MLL-
16	WT1 low	M	59	13	3.40	46,XY,+1,der(1;7)(q10;p10) / 46,XY	FLT3-NPM-CEBPA-MLL-
17	WT1 low	F	76	43	0.47	46,XX	nd
18	WT1 low	F		20	1.83	46,X,idic(X)(q13)/46,XX,t(2;3)/46,XX	FLT3-NPM-CEBPA-MLL-
19	WT1 low	M		38	53.60	47,XY,-2,5q-,-6,+der(8),20q-,-21,-22,+3mar / 46,XY	FLT3-NPM-CEBPA-MLL-
20	WT1 low	F		7.5	1.62	45,XX,-12,t(17;18)(q10;q10),19p+,-21,+4	FLT3-NPM-CEBPA-MLL-
21	WT1 low	F	79	83	32.70	NA	FLT3-NPM-CEBPA-
22	WT1 low	F	60	100	1.23	46,XY,7q- / 46,XY	FLT3-NPM-MLL-

Patient #	WT1 status	Sex	Age (years)	[WT1/ABL1] x 10^4 WT1 copies	SGK1 $\Delta\Delta C_T$	Cytogenetics	Molecular
1	WT1 high	F	64	705	0.82	46,XX	NPM+
2	WT1 high	M	52	44090	0.02	NA	FLT3+ NPM- CEBPA- MLL- PMLRARA+
3	WT1 high	F		7500	0.03	NA	FLT3- NPM- CEBPA- MLL+
4	WT1 high	F	82	2227	1.07	NA	FLT3- NPM+ CEBPA- MLL-
5	WT1 high	F	67	24782	0.08	45,XX,-3,5q-,+8,16q+,-18,-21,+mar,dmin / 47,-3,5q-,+8,16q+,-1	FLT3- NPM- CEBPA- MLL-
6	WT1 high	M	79	4842	0.39	46,XY	FLT3- NPM+ CEBPA- MLL-
7	WT1 high	M	52	3962	0.06	43-46,X,der(Y)t(Y;17)(q10;q10),2q+,-3,5q-,t(7;?)(q32;?),7q-,+2r	FLT3- NPM- CEBPA- MLL-
8	WT1 high	M	68	1727	0.03	47,XY,+8 / 46,XY	FLT3- NPM- CEBPA- MLL-
9	WT1 high	M	66	882	0.15	46,XY	FLT3- NPM- CEBPA- MLL-
10	WT1 high	M		1642	4.29	46,XY	FLT3- NPM+ CEBPA- MLL-
11	WT1 high	F		7872	0.86	NA	FLT3- NPM- CEBPA- MLL-
12	WT1 high	M		960	0.21	47,XY,+8/46,XY	FLT3- NPM- CEBPA- MLL-
13	WT1 high	F		339	0.17	46,X,t(X;21)(p11;q22)/46,XX	FLT3-
14	WT1 high	M	42	3000	0.07	46,XY	FLT3- NPM- CEBPA- MLL-
15	WT1 high	M	52	4500	0.12	46,XY	FLT3- NPM- CEBPA- MLL-
16	WT1 high	F	83	5714	0.14	46,XX,5q- / 46,XX	FLT3- NPM- CEBPA- MLL-
17	WT1 high	M		2968	0.03	NA	FLT3- NPM-
18	WT1 high	M	81	625	1.67	48,XY,+8,+13/46,XY	FLT3- NPM- CEBPA- MLL-
19	WT1 high	F	45	1625	2.62	46,XX	FLT3- NPM+ CEBPA- MLL-
20	WT1 high	M	41	3207	0.12	46,XY	FLT3+ NPM- CEBPA- MLL-
21	WT1 high	M	66	833	0.27	46,XY	FLT3- NPM+ CEBPA- MLL-
22	WT1 high	M	54	666	0.18	56,XY,1p+,+3,+4,+6,+8,der(9),+12,+16,+18,+19,+20,+21/46,,X	FLT3- NPM- CEBPA- MLL-
23	WT1 high	F	53	5625	0.34	46,XX	FLT3- NPM+ CEBPA- MLL-
